# Neoantigens and shared MICB α3 antigen dual-targeted vaccine generates potent antitumor immunity

**DOI:** 10.1038/s44321-026-00424-6

**Published:** 2026-04-17

**Authors:** Ruijing Tang, Honghao Ye, Geng Chen, Xiuqing Dong, Zhenli Li, Fangzhou Lin, Tingfeng Huang, Liman Qiu, Gengping Lin, Ming Wu, Haijun Yu, Jianhua Zou, Xiaolong Liu, Zhixiong Cai

**Affiliations:** 1 The United Innovation of Mengchao Hepatobiliary Technology Key Laboratory of Fujian Province & Mengchao Hepatobiliary Hospital of Fujian Medical University, Fuzhou, China; 2 The Liver Disease Research Center of Fujian Province, Fuzhou, China; 3 College of Pharmacy, Taizhou University, Taizhou, China; 4 State Key Laboratory of Drug Research & Center of Pharmaceutics, Shanghai Institute of Materia Medica, Chinese Academy of Sciences, Shanghai, China; 5 Department of Diagnostic Radiology, Yong Loo Lin School of Medicine, National University of Singapore, Singapore, Singapore

**Keywords:** Cancer, Digestive System, Immunology

## Abstract

Immune suppression is one of the primary obstacles in neoantigen immunotherapy because tumors can rapidly adapt by reducing MHC-I expression or antigen presentation. Here, we developed a novel immunotherapy strategy that combined vaccination of neoantigens with MICB α3 antigen, by using bacterial outer membrane vesicles (OMVs) as a versatile vector and adjuvant. This approach aims to simultaneously induce a neoantigen-specific cellular immune response and an anti-MICB α3 humoral immune response, to enhance the recognition and killing of tumor cells by immune cells. This strategy significantly improves the infiltration of neoantigen-specific T cells and NK cells, and reverses immunosuppression across various preclinical models. Mechanistically, ILC1s characterized by high GZMA/GZMB expression represent the primary subset accumulating within tumors and are responsible for enhancing antitumor immunity, which can induce Gasdermin D cleavage in tumor cells to initiate tumor pyroptosis for a cascade of cancer-immunity cycle. Overall, this study demonstrated that combined neoantigens and shared MICB α3 antigen for tumor vaccination enhances immune efficacy by eliciting ILC1s-mediated tumor pyroptosis and support the rationale and clinical translation for cancer immunotherapy.

## Synopsis

A bacterial outer membrane vesicle (OMV)-based dual-targeted vaccine was developed for combined delivery of personalized neoantigens and the shared MICB α3 domain to elicit synergistic antitumor immunity in solid tumors.

- The OMV vaccine platform simultaneously induced neoantigen-specific cellular immunity and anti-MICB α3 humoral immunity, overcoming tumor immune evasion mechanisms such as MICA/B shedding.
- The combined treatment activated a population of IL-15-armed ILC1s, which mediated GSDMD-dependent tumor pyroptosis and amplified the antitumor immune response.
- The therapeutic strategy demonstrated broad applicability and safety across multiple preclinical models, supporting its potential for clinical translation in various cancer types.

## Abstract (web summary)

A bacterial outer membrane vesicle (OMV)-based dual-targeted vaccine was developed for combined delivery of personalized neoantigens and the shared MICB α3 domain to elicit synergistic antitumor immunity in solid tumors.

## Graphical abstract

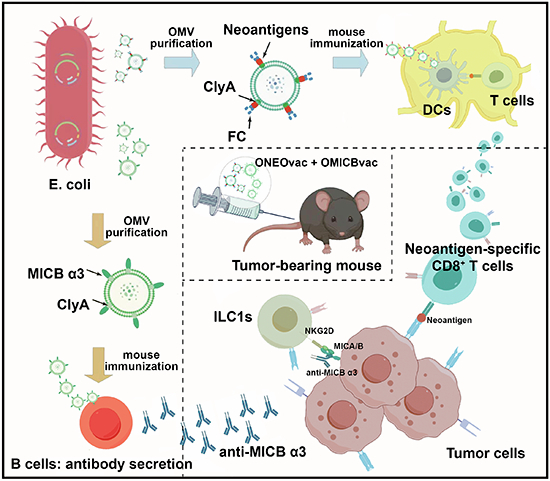

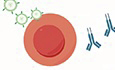

The paper explained **Problem**

Neoantigen-based vaccines hold great promise for personalized cancer immunotherapy, yet their efficacy is often compromised by tumor immune evasion mechanisms. Moreover, even when neoantigen-specific T cells are successfully primed, they often become exhausted in the immunosuppressive tumor microenvironment. Therefore, there is an urgent need for integrated vaccine platforms that can simultaneously enhance antigen presentation, prevent immune escape, and sustain potent antitumor immunity across diverse tumor types.

**Results**

We developed a novel bacterial outer membrane vesicle (OMV)-based vaccine platform that co-delivers personalized neoantigens and the shared MICB α3 domain. This dual-targeted approach simultaneously activates neoantigen-specific CD8⁺ T cells and induces high-titer antibodies that block MICB shedding, thereby preserving NKG2D-mediated tumor recognition. In multiple orthotopic and metastatic mouse models of hepatocellular and pancreatic cancers, the combined vaccine elicited superior antitumor efficacy and prolonged survival compared to either component alone. Mechanistically, single-cell RNA sequencing revealed that the therapy recruited and activated a population of IL-15-armed ILC1s, which mediated tumor pyroptosis via GZMA/GZMB-induced cleavage of GSDMD. This pyroptotic cell death amplified the inflammatory tumor microenvironment and enhanced systemic antitumor immunity without inducing significant toxicity.

**Impact**

This study introduces a versatile and synergistic vaccination strategy that merges personalized and shared antigen targeting to overcome key limitations of current neoantigen immunotherapies. Importantly, the OMV-based delivery system is scalable, clinically tractable, and adaptable to various cancer types, offering a translational pathway toward more effective and broadly applicable cancer vaccines.

## Introduction

Recent studies have demonstrated that immunogenic peptides generated by specific gene variations (such as mutations, RNA editing, insertions, deletions, etc.) in tumor cells can be presented on the cell surface by MHC complexes and subsequently recognized by TCR receptors on T cells, inducing specific tumor cell killing (Lang et al, 2022; Xie et al, 2023). These immunogenic peptides, known as tumor neoantigens, are highly attractive targets for tumor immunity due to their exclusive presence in tumors, their capacity to elicit a robust antitumor immune response with minimal off-target effects, and the lack of central tolerance. Precision immunotherapy targeting tumor neoantigens, such as peptide or mRNA vaccines, has been shown to induce the generation and infiltration of tumor neoantigen-specific T cells into various solid tumors, including hepatocellular carcinoma (HCC) and pancreatic cancer, leading to potent antitumor effects (Baretti et al, 2025; Castle et al, 2012; Linnemann et al, 2015; Robbins et al, 2013; Yarchoan et al, 2024). However, for tumors with rapid progression and strong heterogeneity, such as HCC, neoantigen vaccines alone often do not achieve expected efficacy (Cai et al, 2021). In clinical trials, combining neoantigen vaccines with immune checkpoint inhibitors is a common strategy. This combination can relieve the immunosuppressive microenvironment and prevent the exhaustion of tumor-specific T cells. Nevertheless, preliminary study has shown that the overall response rate (ORR) of such immunotherapy in HCC is only 30.4%, indicating the presence of numerous other immune suppression mechanisms (Peng et al, 2019).

The rapid adaptation and evolution within a patient’s tumor are key factors for tumor immune suppression. Tumor cells can evolve various branches during development, leading to antigen loss or low major histocompatibility complex (MHC) expression in some tumor cells, making them unrecognizable and unkillable to tumor-specific T cells. Meanwhile, NK cells can recognize MHC class I chain-related polypeptide A/B (MIC A/B) highly expressed on tumor cells through NKG2D, which in turn eliminate tumor cells. However, MICA/B are frequently proteolytically shed in the α3 domain by tumor cells to evade NK cell-mediated immunity (Boutet et al, 2009; Groh et al, 2002; Kaiser et al, 2007; Waldhauer et al, 2008). Addressing these shortcomings in immune cells’ ability to recognize tumor cells will be essential to improve the applicability of neoantigen-based therapies. The research group led by Kai W. Wucherpfennig demonstrated that MICA/B shedding can be blocked by antibodies that bind to the α3 domains, and developed a vaccine consisting of the α3 domain of MICA or MICB fused to the N-terminus of ferritin from Helicobacter pylori for multivalent antigen display, to induce high-titer antibodies derived from MICB α3 domain for inhibiting the shedding of MICB protein from the surface of tumor cells, thereby preventing immune suppression and inhibiting tumor cell growth (Ferrari de Andrade et al, 2018). Here, we propose a novel tumor vaccine treatment strategy that targets tumor immune suppression mechanisms by simultaneously immunizing the host with tumor neoantigens and shared MICB α3 antigen, thus utilizes MICB α3 antibodies to enhance the tumor cell recognition of immune cells induced by the neoantigen vaccine.

Moreover, to reduce potential side effects, one of the key factors is how to apply the clinically accessible delivery strategy with easy manipulation and rapid preparation to efficiently deliver tumor neoantigens and shared MICB α3 antigen for inducing the efficient generation of tumor neoantigen-specific immune cells and MICB α3 antibodies in vivo. Outer membrane vesicles (OMVs) are nanoscale vesicles naturally released from the outer membrane of Gram-negative bacteria with a size of 30–250 nm (Schwechheimer and Kuehn, 2015). They have garnered significant attention as antigen delivery carriers and adjuvants due to their intrinsic immunostimulatory properties and ability to present a diverse array of antigens. By harnessing their capability to deliver antigens to antigen-presenting cells, OMVs can effectively activate dendritic cells (DCs) and promote their maturation, increasing the expression of MHC class II (MHC-II) and CD86, and producing pro-inflammatory cytokines such as tumor necrosis factor α (TNF-α) and interleukin-12 (IL-12) (Gnopo et al, 2017; Kaparakis-Liaskos and Ferrero, 2015; Kim et al, 2017). Regarding humoral immunity, OMVs can bind to B cell receptors (BCRs) or Toll-like receptors (such as TLR2 and TLR9) on the surface of B cells, inducing their activation and proliferation. This leads to the production of polyclonal IgG and IgM antibodies (Hayashi et al, 2001; Kuzmich et al, 2017; Oliveira-Nascimento et al, 2012; Russo et al, 2018). Therefore, OMVs could serve as an excellent carrier for delivering personalized tumor neoantigens and shared MICB α3 antigen. However, how to synergistically immunize these two antigens in vivo to achieve desired anti-tumor efficacy remains to be explored.

Here, based on the OMVs platform, we designed a novel synergistic therapeutic vaccine strategy that combines neoantigens and MICB α3 antigen to induce cellular-specific immunity and antibody humoral immunity. This approach aims to enhance the recognition and killing of tumor cells by immune cells and reduce the risk of potential immune suppression in fast-progressing and highly heterogeneous cancers like hepatocellular carcinoma. Using multiple orthotopic tumor models and multi-omics techniques, we validated that this synergistic vaccine strategy could elicit stronger immunogenicity and induce tumor cell pyroptosis through ILC1s. Overall, this study provides a new perspective and therapeutic strategy to reduce the potential immune suppression of tumors, laying a solid foundation for its potential clinical application.

## Results

### Design and characterization of the OMV-based neoantigen vaccines

**Figure 1:**
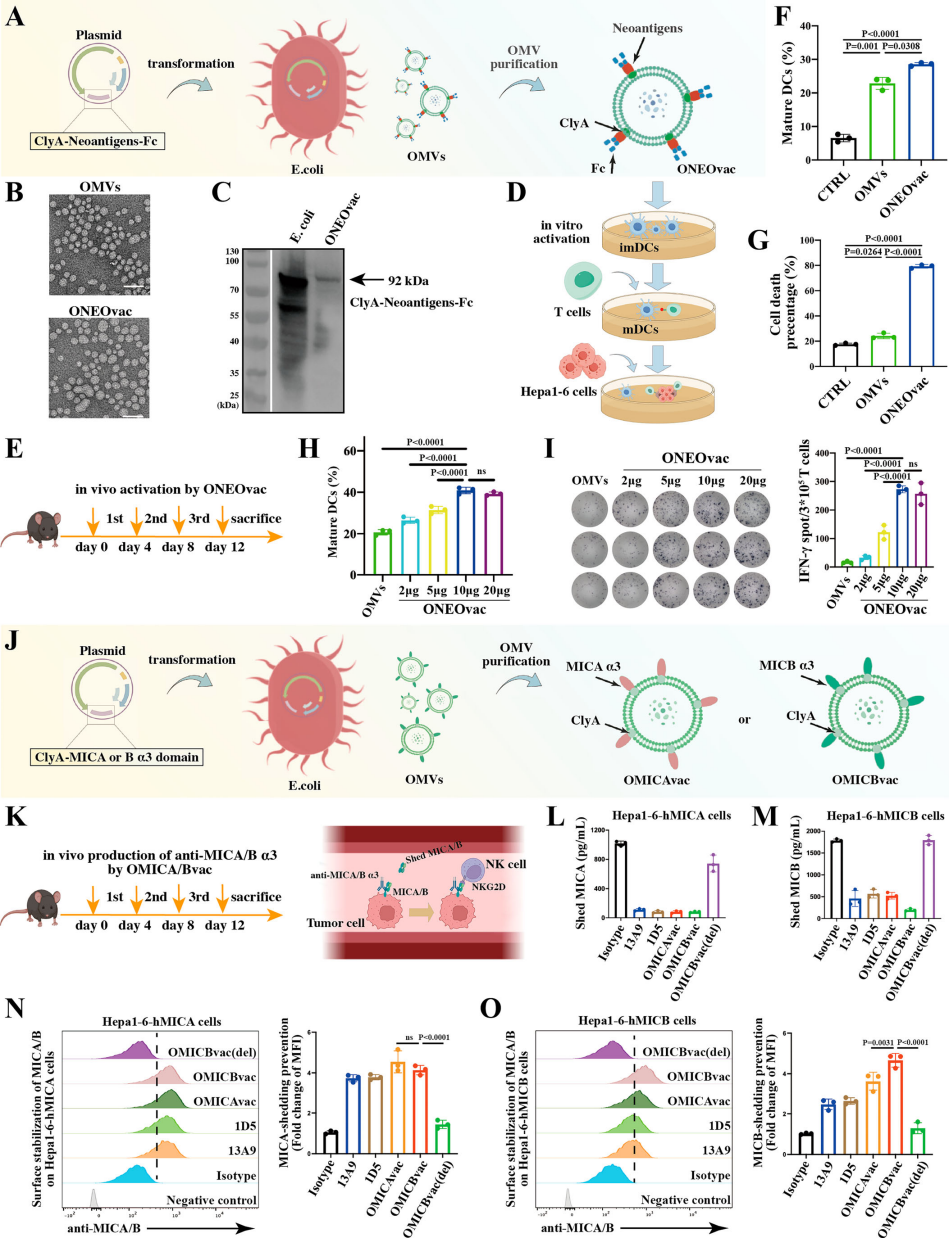
The preparation and functional analysis of OMICBvac and ONEOvac. (**A**) Schematic diagram illustrating the preparation of ONEOvac. (**B**) Transmission electron microscopy (TEM) analysis of OMVs (ClyA-none) and ONEOvac (ClyA-Neoantigens-Fc). Scale bar, 50 nm. (**C**) Western blot analysis showing the presence of ClyA-Neoantigens-Fc (92 kDa) in OMVs produced by *E. coli* Rosetta (DE3). Protein expression was induced using 0.5 mM IPTG and 20 μg of each sample was finally loaded for electrophoresis. (**D**–**G**) Schematic diagram of the in vitro cytotoxicity evaluation of ONEOvac. First, bone marrow-derived immature dendritic cells (BMDCs, 1×10^6^) were stimulated with ONEOvac (2 μg/mL) for 24 h, and the percentage of mature BMDCs (CD80^+^ and CD86^+^ within CD11c^+^ cells) was measured by flow cytometry (**F**) (*n* = 3 independent replicates; one-way ANOVA). Next, mouse splenic T cells (1 × 10^6^), separated from the mice immunized by ONEOvac (**E**), were cocultured with mature BMDCs for 48 h. Subsequently, Hepa1-6 cells (5 × 10^5^) were cocultured with BMDC-activated T cells, and T cell-mediated killing efficiency was assessed by flow cytometry using PI and Annexin V staining (**G**) (*n* = 3 independent replicates; one-way ANOVA). (**H**) Statistical analysis of flow cytometry data showing the percentage of DC maturation in axillary lymph nodes stimulated by OMVs (2 μg) and different amounts of ONEOvac (*n* = 3 independent replicates; one-way ANOVA). The lymph nodes were harvested from (**G**) after the mice were sacrificed on day 12. (**I**) ELISPOT assay evaluation of the neoantigen-specific immune response induced by OMVs (2 μg) and different amounts of ONEOvac (*n* = 3 independent replicates; one-way ANOVA). The splenic T cells for ELISPOT assay were harvested from (**G**) after the mice were sacrificed on day 12 (*n* = 3 independent replicates; one-way ANOVA). (**J**) Schematic diagram of the OMICA/Bvac preparation. (**K**) Schematic representation of in vivo induction of anti-MICA/B α3 by OMICBvac (10 μg). (**L**, **M**) MICA or MICB-overexpressing Hepa1-6 cells (5 × 10^5^) were treated for 24 h with serum (diluted 100-fold) from (**K**). Shed MICA (**L**) and MICB (**M**) were quantified in the supernatant by ELISA (*n* = 3). (**N**) Flow cytometry analysis of the stabilization of MICA/B on MICA-overexpressing Hepa1-6 cells after 24 h treatment by serum from (**K**) (*n* = 3 independent replicates; one-way ANOVA). (**O**) Flow cytometry analysis of the stabilization of MICA/B on MICB-overexpressing Hepa1-6 cells after 24 h treatment by serum from (**K**) (*n* = 3 independent replicates; one-way ANOVA). Data are presented as the mean ± SEM. *P < 0.05, **P < 0.01, ***P < 0.001, ****P < 0.0001; ns, no significance. Source data are available online for this figure.

To improve the delivery efficiency and immune response, OMVs from *E. coli* were selected and genetically engineered as the carrier and adjuvant for neoantigens. The sequence of ClyA (one of the most abundant proteins on the OMVs surface), 3×hemagglutinin tags (HA)-tag, 7 highly immunogenic neoantigens derived from HCC cell line Hepa1-6 cells which has been identified for developing tumor vaccines in our previous study (Appendix Table S1) (Chen et al, 2022), and the Fc domain of mouse immunoglobulin G (IgG; Fc: protein for target to antigen presenting cells) was fused in the plasmid pET28a. Then this plasmid was transformed in *E. coli* Rosetta (DE3) for protein expression (Fig. 1A). During the culture of *E. coli* induced by IPTG, OMVs loaded with recombinant ClyA-3×HA-Neoantigens-Fc protein (Clya-Neo-Fc) in the membrane were budded and secreted into supernatant, and then were collected by ultracentrifugation. Subsequently, the OMVs were characterized by transmission electron microscopy (TEM) and dynamic light scattering (DLS), and the results showed a uniform circular morphology with a diameter of ~35 nm (Fig. 1B; Appendix Fig. S1A). Western blot analysis by HA-tag antibody indicated that ClyA-3×HA-Neoantigens-Fc protein (93kD) can be obviously detected in these OMVs, suggesting that OMVs containing neoantigen peptides (termed as ONEOvac) were successfully prepared (Fig. 1C). To further confirm whether the ONEOvac could stimulate an effective immune response by activating dendritic cells (DCs), ONEOvac were firstly added to immature bone marrow-derived DCs (BMDCs) for inducing maturation and neoantigen processing (Fig. 1D). Then, the DCs were co-cultured with splenic T cells from ONEOvac-vaccined mice to generate neoantigen specific T cells by neoantigen presenting. Here, the mice were subcutaneously immunized 5 μg ONEOvac at the lateral flank of C57BL/6 mice on day 0, day 4 and day 8 (Fig. 1E). Since OMVs themselves included pathogen-associated molecular patterns (PAMPs) and lipopolysaccharide (LPS) to promote the activation of DC cells, the proportion of CD80^+^ and CD86^+^ cells from CD11c^+^ DCs (mature DCs) with OMVs (without neoantigens loading) stimulation was already upregulated compared with the PBS-treated group (*P* = 0.001, Fig. 1F; Appendix Fig. S1B). More significantly, ONEOvac stimulation could induce even higher proportions of DC maturation comparing to OMVs treatment (*P *= 0.0308) or PBS treatment (*P* < 0.0001), respectively. Furthermore, when co-incubated with Hepa1-6 cells, the activated T cells collected from ONEOvac treatment consistently showed the highest proportion of tumor cell death comparing with the PBS-treated group and OMVs-treated group (*P* < 0.0001 and *P *< 0.0001, Fig. 1G; Appendix Fig. S1C). To further test whether ONEOvac could induce neoantigen-specific immune responses in vivo, different dosage of ONEOvac (2 μg, 5 μg, 10 μg and 20 μg) was subcutaneously immunized at the lateral flank of C57BL/6 mice on day 0, day 4 and day 8, respectively (Fig. 1D). Then, these immunized mice were sacrificed at day 12, and DC in lymph nodes and splenic T cells were collected for flow cytometry analysis and ex vivo interferon (IFN)-γ assay by ELISPOT, respectively. As shown in Fig. 1H,I, ONEOvac with 10 μg dosage vaccination could induce higher proportion of mature DCs (*P* < 0.0001) and stronger neoantigen-specific immune responses (*P* = 0.0006) in vivo when compared with 5 μg dosage vaccination; however, when the dosage was increased to 20 μg, there was no significant further enhancement in the immune response. Taken together, these results clearly demonstrated that ONEOvac with 10 μg dosage vaccination could effectively promote well neoantigen presenting by DCs and induce more neoantigen-specific immune responses.

### Immunization of shared MICB α3 domain-loading OMVs in vivo inhibits the shedding of MICA/B from tumor cells

The α3 domain of MICA/B has been identified as a critical site on the surface of tumor cells where MICA/B is proteolyzed by proteases (Li et al, 2001; Wang et al, 2009). Anti-MICA/B α3 domain antibody could specifically block the recognition site of protease, thereby preventing the proteolytic shedding of MICA/B (Ferrari de Andrade et al, 2018). MICA and MICB share highly homologous amino acid sequences, particularly in their α3 domains where sequence similarity exceeds 90%. Previous report has demonstrated that the epitopes in MICA (SHDTQQ) and MICB (SHNTQQ) that activate B cells to produce antibodies inhibiting MICA and MICB shedding are remarkably similar (Wang et al, 2025). To determine whether OMV-displayed MICA α3 domain or MICB α3 domain more effectively inhibits the shedding of both MICA and MICB, we genetically fused human-derived MICA α3 domain, MICB α3 domain, or the SHNTQQ-deleted MICB mutant domain to ClyA in the plasmid pET28a. These constructs were then transformed into *E. coli* Rosetta (DE3) to produce OMVs carrying the respective domains: MICA α3 domain (termed as OMICAvac), MICB α3 domain (termed as OMICBvac), and the SHNTQQ-deleted MICB mutant domain (termed as OMICBvac(del)) (Fig. 1J). Subsequently, OMICAvac, OMICBvac, and OMICBvac(del) were subcutaneously administered to C57BL/6 mice three times to generate antibodies (the human-derived MICA/B α3 domain-specific IgG) against MICA/B shedding in vivo (Fig. 1K). To verify whether the specific IgG produced in the serum could inhibit the cleavage of MICA/B proteins (specifically the α1 and α2 domains) on tumor cell surfaces, the serum was directly co-cultured with Hepa1-6 cells overexpressing either human MICA or MICB. As positive controls, we synthesized two previously characterized monoclonal antibodies (13A9 and 1D5) targeting MICA α3 (Appendix Fig. S2), known to suppress MICA/B shedding (Lombana et al, 2019). Since MICA/B α1 and α2 domains are continuously proteolyzed and released into culture supernatants, ELISA was used to quantify these domains in the supernatant as a measure of MICA/B proteolytic shedding from the tumor surface. After 24 h of incubation, compared to the monoclonal antibodies (13A9 and 1D5) directly co-cultured with Hepa1-6 cells in vitro, antibodies induced by both OMICAvac and OMICBvac exhibited similar inhibitory effects on the shedding of both MICA and MICB (Fig. 1L,M). As expected, upon deletion of the “SHNTQQ” epitope, OMICBvac(del) lost its ability to induce antibodies targeting MICA/B for shedding inhibition (Fig. 1L,M). Furthermore, antibody-mediated stabilization of surface MICA/B on Hepa1-6 cells was assessed by flow cytometry. Except for the isotype and OMICBvac(del) control groups, both OMICAvac and OMICBvac treatment groups demonstrated comparable capacity to stabilize surface MICA/MICB expression on Hepa1-6 cells relative to the monoclonal antibody groups (Fig. 1N,O). Notably, the OMICBvac treatment group achieved higher level inhibition of both MICA and MICB shedding, superior to OMICAvac.

To further compare the antitumor efficacy between OMICAvac and OMICBvac, we established a hepatocellular carcinoma subcutaneous tumor model using Hepa1-6-hMICB cells (recorded as day −10). Mice were randomly divided into five groups and subsequently vaccinated with PBS, 13A9, 1D5, OMICAvac, or OMICBvac on days 0, 4, and 8, respectively (Appendix Fig. S3A). After 16 days of treatment, the antitumor effects of OMICAvac and OMICBvac were significantly stronger than those of 13A9 and 1D5, primarily manifested by tumor growth inhibition, enhanced infiltration of CD8^+^ T cells, and sustained in vivo generation of anti-MICA/B antibodies (Appendix Fig. S3B–G). These results suggest that the humoral immunity induced by OMICAvac and OMICBvac, which maintains higher and more persistent antibody levels in vivo, confers a greater advantage for antitumor effects. Based on these findings, we selected OMICBvac carrying a uniform spherical shape with MICB α3 domain loading (56 kDa) for subsequent antitumor analysis (Appendix Fig. S4A–C). Then, varying dosages of OMICBvac (2 μg, 5 μg, 10 μg, and 20 μg) were subcutaneously administered to C57BL/6 mice. As shown in Appendix Fig. S4D,E, 10 μg OMICBvac induced the highest levels of specific antibodies (human-derived MICB α3 domain-specific IgG) compared to other dosages. Collectively, these results demonstrate that exogenous MICB α3 domain displayed by OMVs effectively mobilizes the immune system to generate antibodies that inhibit MICA/B shedding from tumor cells, thereby exerting significant antitumor potential.

### Antitumor immune response evaluation of ONEOvac and OMICBvac in vivo

**Figure 2:**
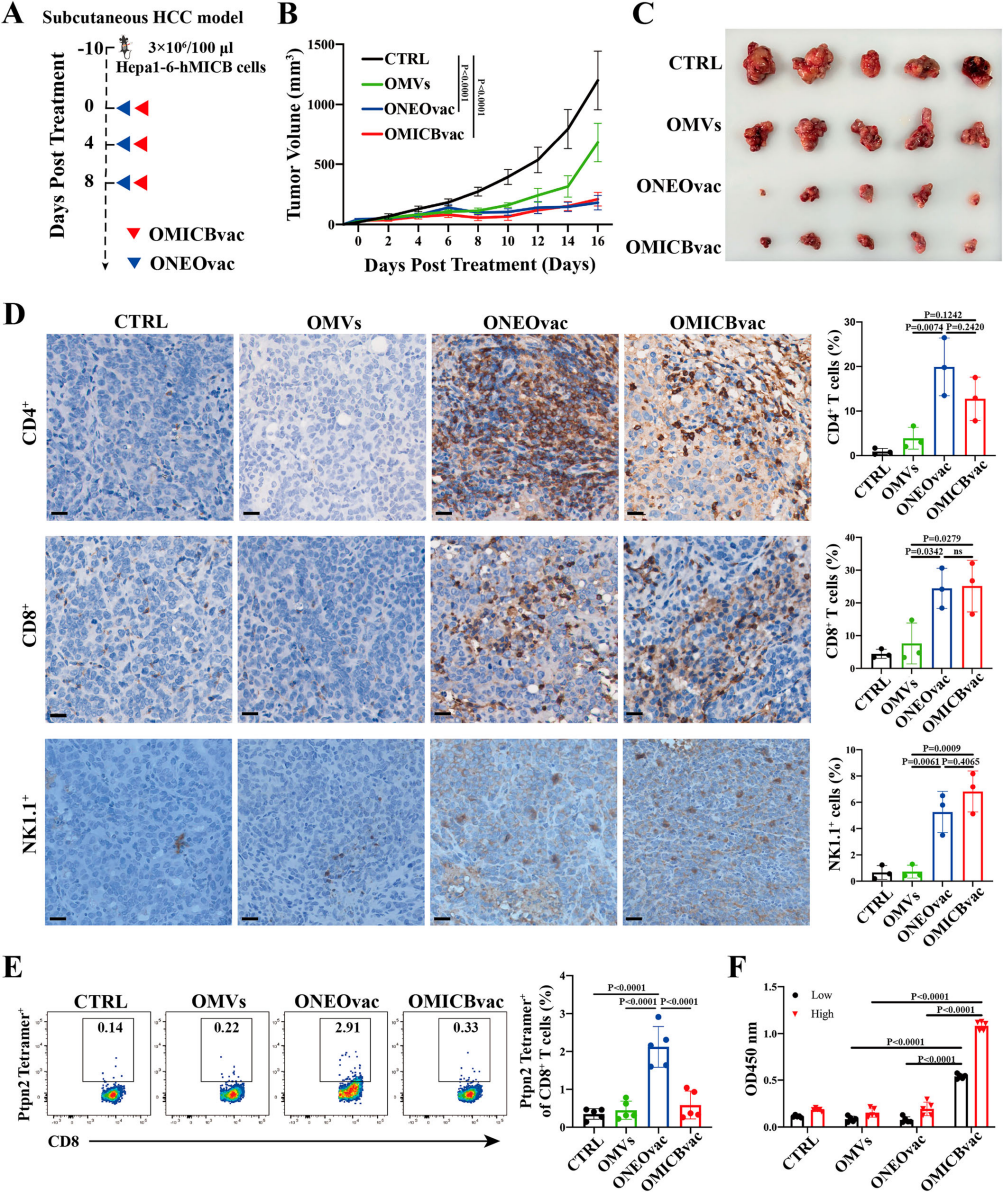
The antitumor efficacy evaluation of ONEOvac and OMICBvac in vivo. (**A**) Schematic representation of the Hepa1-6-hMICB subcutaneous HCC model and the treatment timeline. After 10 days of tumor cell inoculation, the mice received different treatments, including PBS, OMVs (10 μg), OMICBvac (10 μg) and ONEOvac (10 μg), on days 0, 4, and 8, respectively. After 16 days of treatment, tumors and serum were collected from the mice for subsequent analysis. (**B**, **C**) Tumor volume monitoring of mice (*n* = 5) treated with PBS, OMVs, ONEOvac and OMICBvac. The tumor size was recorded every 2 days (*n* = 6 mice per group; two-way ANOVA). (**D**) IHC images show CD4^+^ T cells, CD8^+^ T cells, and NK1.1^+^ NK cells infiltrating into tumor tissues (*n* = 3 independent replicates; one-way ANOVA) in each treatment group as indicated. The samples were harvested from (**C**), and the statistical results were analyzed using QuPath software. Scale bars, 20 μm. (**E**) Flow cytometry analysis of the percentage of Ptpn2 tetramer^+^ specific CD8^+^ T cells within CD8^+^ tumor-infiltrating lymphocytes (TILs) (*n* = 5 independent replicates; one-way ANOVA). The samples used for T cell detection were from the single-cell suspension of tumor tissue prepared from (**C**). (**F**) ELISA quantification of mouse anti-MICB α3 IgG levels in serum (*n* = 5 independent replicates; two-way ANOVA). The absorbance was read at 450 nm. Low: serum was diluted 10000-fold. High: serum was diluted 100-fold. Data are presented as the mean ± SEM. *P < 0.05, **P < 0.01, ***P < 0.001, ****P < 0.0001; ns, no significance. Source data are available online for this figure.

To verify the antitumor immune responses induced by ONEOvac and OMICBvac, an HCC subcutaneous tumor model based on Hepa1-6-hMICB cells were established (recorded as day −10). The mice were randomly divided into four groups and further vaccinated by PBS, OMVs (10 μg), ONEOvac (10 μg) or OMICBvac (10 μg) on days 0, 4 and 8, respectively (Fig. 2A). As shown in Fig. 2B,C, after 16 days of treatment, the tumor grew rapidly in mice treated with PBS and OMVs, while a significant tumor suppression was observed in both the mice treated by ONEOvac and OMICBvac (*P* = 0.0278 and *P* = 0.0049). To uncover the potential immune response and anti-tumor effects induced by ONEOvac and OMICBvac, the infiltration of T cells (CD4 and CD8) and NK cells (NK1.1) were assessed by IHC staining. The infiltration of CD3^+^CD4^+^ T cells (ONEOvac vs OMVs, *P* = 0.0074, OMICBvac vs OMVs, *P* = 0.1242), CD3^+^CD8^+^ T cells (ONEOvac vs OMVs, *P* = 0.00342, OMICBvac vs OMVs, *P *= 0.0279) and NK cells (ONEOvac vs OMVs, *P* = 0.0061, OMICBvac vs OMVs, *P* = 0.0009) in tumor tissues were all significantly increased after immunization with ONEOvac and OMICBvac (Fig. 2D). Furthermore, tumors were harvested and digested into single-cell suspensions to detect the neoantigen-specific T cells that expressing Ptpn2-specific T-cell receptors (TCRs) in the TILs. Peptide-MHC tetramer staining revealed only ONEOvac vaccination but not OMICBvac vaccination can induce a threefold increase of intra-tumoral neoantigen-specific CD8^+^ T cells (vs PBS, OMVs and OMICBvac, all *P* < 0.0001, Fig. 2E). Different from the T cell-based anti-tumor cellular immunity activated by the ONEOvac, after OMICBvac vaccination, the immune cells involved in humoral immunity (such as B cells, macrophages) could phagocytose and process the MICB α3 domain antigen information and produce a large amount of corresponding IgG antibodies. These antibodies could reach the tumor site through peripheral blood circulation, and further bind to the α3 domain of the MICB protein on the tumor surface, thereby inhibiting the cleavage and shedding of MICB protein. As expected, OMICBvac treatment could induce higher level of anti-human MICB α3 antibodies in the serum than OMVs and ONEOvac treatment (all *P* < 0.0001, Fig. 2F), suggesting more antibodies were produced against the MICB α3 domain by OMICBvac vaccination. Taken together, these data indicated that both ONEOvac and OMICBvac could achieve significant anti-tumor efficacy by tumor-specific cellular immunity and humoral immunity in vivo, respectively.

### Combining ONEOvac and OMICBvac elicited stronger antitumor immune response in mouse orthotopic tumor model

**Figure 3:**
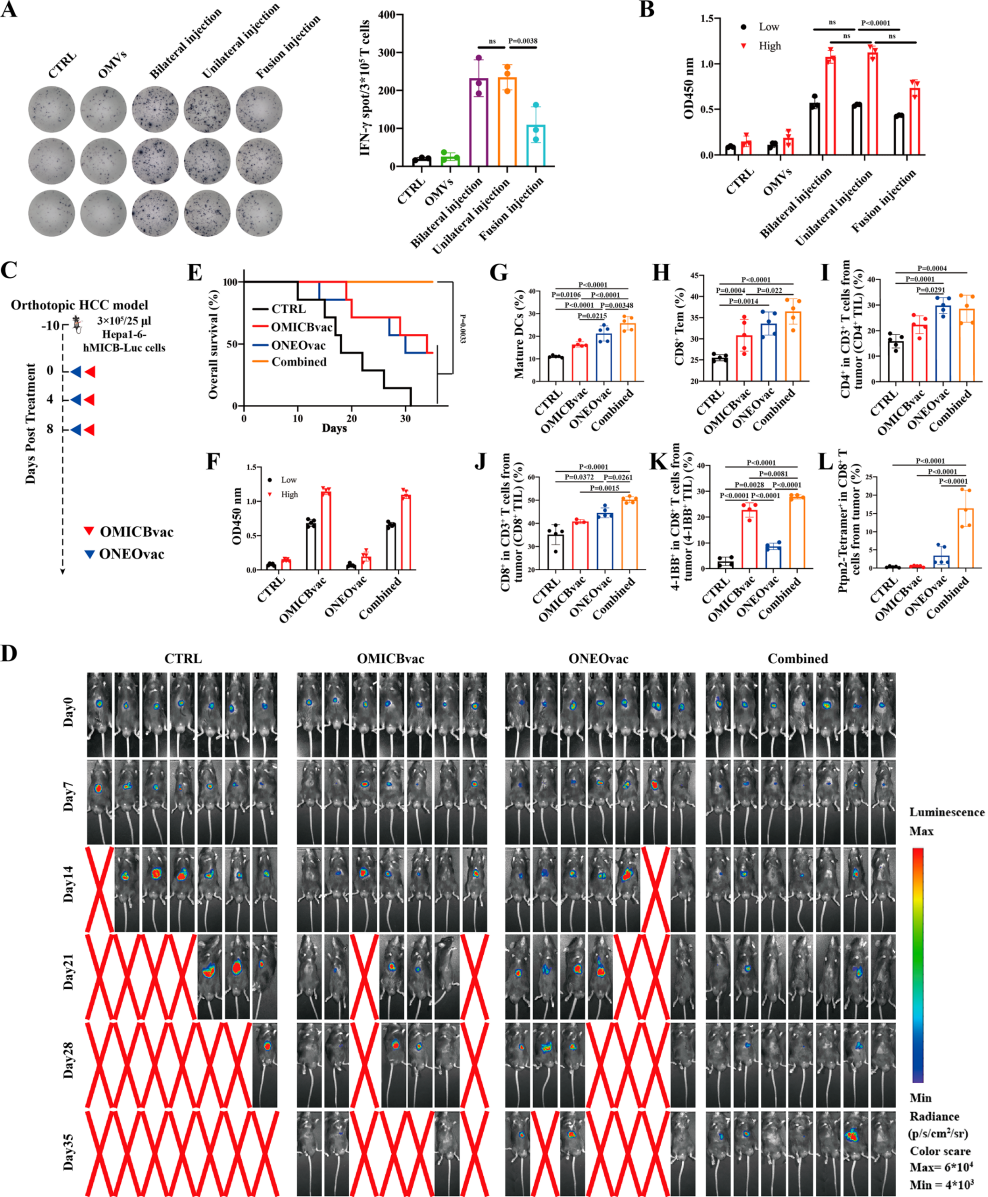
The antitumor efficacy of ONEOvac and OMICBvac combined treatment in the orthotopic HCC tumor model. (**A**, **B**) Evaluation of neoantigen-specific immune response (**A**) (*n* = 3 independent replicates; one-way ANOVA) and anti-MICB α3 IgG levels (**B**) (*n* = 3 independent replicates; two-way ANOVA) induced by different co-administration methods. Three mice in each group were immunized three times on days 0, 4, and 8 with one of the following treatments: PBS, OMVs (10 μg), bilateral injection (10 μg ONEOvac + 10 μg OMICBvac in separate sites), unilateral injection (10 μg ONEOvac + 10 μg OMICBvac at the same site), or fusion injection (10 μg ONEO/MICBvac). After 16 days of treatment, splenic T cells and serum were collected from the mice for analysis. Low: serum was diluted 10000-fold. High: serum was diluted 100-fold. (**C**) Schematic diagram illustrates the Hepa1-6-hMICB orthotopic HCC model and the treatment timeline. (**D**) Tumor burden monitoring in mice treated with PBS, OMICBvac, ONEOvac, and the combination of ONEOvac plus OMICBvac, as assessed by bioluminescence imaging (*n* = 7 mice per group). (**E**) Kaplan–Meier survival curves for each treatment group (*n* = 7 mice per group, Statistical analysis was performed with Log-Rank test). (**F**) ELISA measurement of mouse anti-MICB α3 IgG levels in serum. The same models and treatments as in (**C**) were repeated, and the samples were collected and analyzed on day 12 (*n* = 5 mice per group). (**G**) Statistical analysis of flow cytometry data to show the percentage of DC maturation in axillary lymph nodes (*n* = 5 mice per group; one-way ANOVA). (**H**) Percentage of effector memory T cells (CD8^+^ Tem) in the spleen (*n* = 5 mice per group; one-way ANOVA). (**I**–**L**) Flow cytometry analysis of CD4^+^ tumor-infiltrating lymphocytes (TILs) (**I**), CD8^+^ TILs (**J**), 4-1BB^+^CD8^+^ TILs (**K**), and Ptpn2 tetramer^+^ specific CD8^+^ T cells within CD8^+^ TILs (**L**) from each group as indicated (*n* = 5 mice per group; one-way ANOVA). The samples used for T cell detection were from the single-cell suspension of tumor tissues. Data are presented as the mean ± SEM. *P < 0.05, **P < 0.01, ***P < 0.001, ****P < 0.0001; ns, no significance. Source data are available online for this figure.

Tumor immune microenvironment is composed of many cells such as tumor-infiltrating lymphocytes, macrophages, NK cells, and tumor cells, and plays an important role in the development, immune suppression, and treatment resistance of solid tumors (de Visser and Joyce, 2023). The efficacy of a single immunotherapy strategy in treating tumors is often limited and can easily lead to immune suppression and treatment resistance (Zhu et al, 2021). Therefore, we tried to enhance the effect of anti-tumor immunotherapy by combining ONEOvac and OMICBvac. Since different co-administration methods may lead to different immune responses, we tested three combination strategies: (1) bilateral injection (ONEOvac and OMICBvac were subcutaneously injected separately into the bilateral inguinal regions), (2) unilateral injection (ONEOvac and OMICBvac were mixed at a 1:1 ratio and subcutaneously injected into the inguinal region on one side), and (3) fusion injection (the neoantigen and MICB α3 antigen were co-expressed in the same OMVs, which were then subcutaneously injected into the inguinal region on one side). As shown in Fig. 3A,B, surprisingly, the neoantigen-specific cellular immune responses and anti-human MICB α3 antibody titers induced by both the bilateral and unilateral injections were similar but significantly better than those induced by the fusion injection. Therefore, we selected the unilateral injection method for our subsequent studies.

Furthermore, orthotopic HCC mouse model based on the Hepa1-6-hMICB-Luc cell line was constructed to truly simulate the tumor microenvironment (Fig. 3C). Then the orthotopic HCC mice were randomly divided into 4 groups received PBS, OMICBvac alone, ONEOvac alone and OMICBvac plus ONEOvac treatment (the combined group) on day 0, 4 and 8, respectively. As shown in Fig. 3D,E, compared to PBS group, the mice receiving OMICBvac alone or ONEOvac alone showed good tumor growth inhibition, but still 4/7 mice died within 35 days. Impressively, the tumor burden in mice with the combined treatment (unilateral injection) almost completely inhibited in most of the mice, and the 35-day survival rate (100%) was consistently much higher comparing to the other three groups (*P *= 0.0033). In light of these remarkable treatment results, the ability of combined treatment to prevent recurrence of orthotopic HCC was further analyzed. Four mice cured in the combined treatment of Hepa1-6-hMICB orthotopic HCC model were re-injected with Hepa1-6-hMICB cells at liver subcapsular for recurrence re-challenge, and five untreated naïve mice were served as the control (Appendix Fig. S5A). As expected, no tumor growth was observed in all 4 cured mice after reimplantation of the tumor cells, whereas tumors continued to grow in the control group (Appendix Fig. S5B). These results suggested that neoantigen-specific T cells induced by the combined treatment can provide long-term anti-tumor immune protection and effectively prevent recurrence of liver metastases.

To further evaluate the immune response under different treatments, the same models and treatment were re-performed, and the samples were harvested and analyzed on day 12. The concentration of anti-human MICB α3 antibody in the serum of the combined treatment group was comparable in the OMICBvac group (Fig. 3F). These results suggested that the additional injection of ONEOvac does not affect the production of anti-human MICB α3 antibody. Meanwhile, flow cytometry results showed that the proportion of CD11c^+^CD80^+^CD86^+^ DCs (mature DCs) in axillary lymph nodes and CD3^+^CD8^+^CD44^+^CD62L^-^ T cells (T_em_) in spleen were both significantly increased in the combined treatment group when compared with other three groups (Fig. 3G,H; Appendix Fig. S5C,D). Moreover, ELISPOT assays for neoantigen-specific IFN-γ spot detection also indicated that the combined treatment could induce a stronger neoantigen-specific immune response in vivo than the other three groups (Appendix Fig. S5E).

**Figure EV1:**
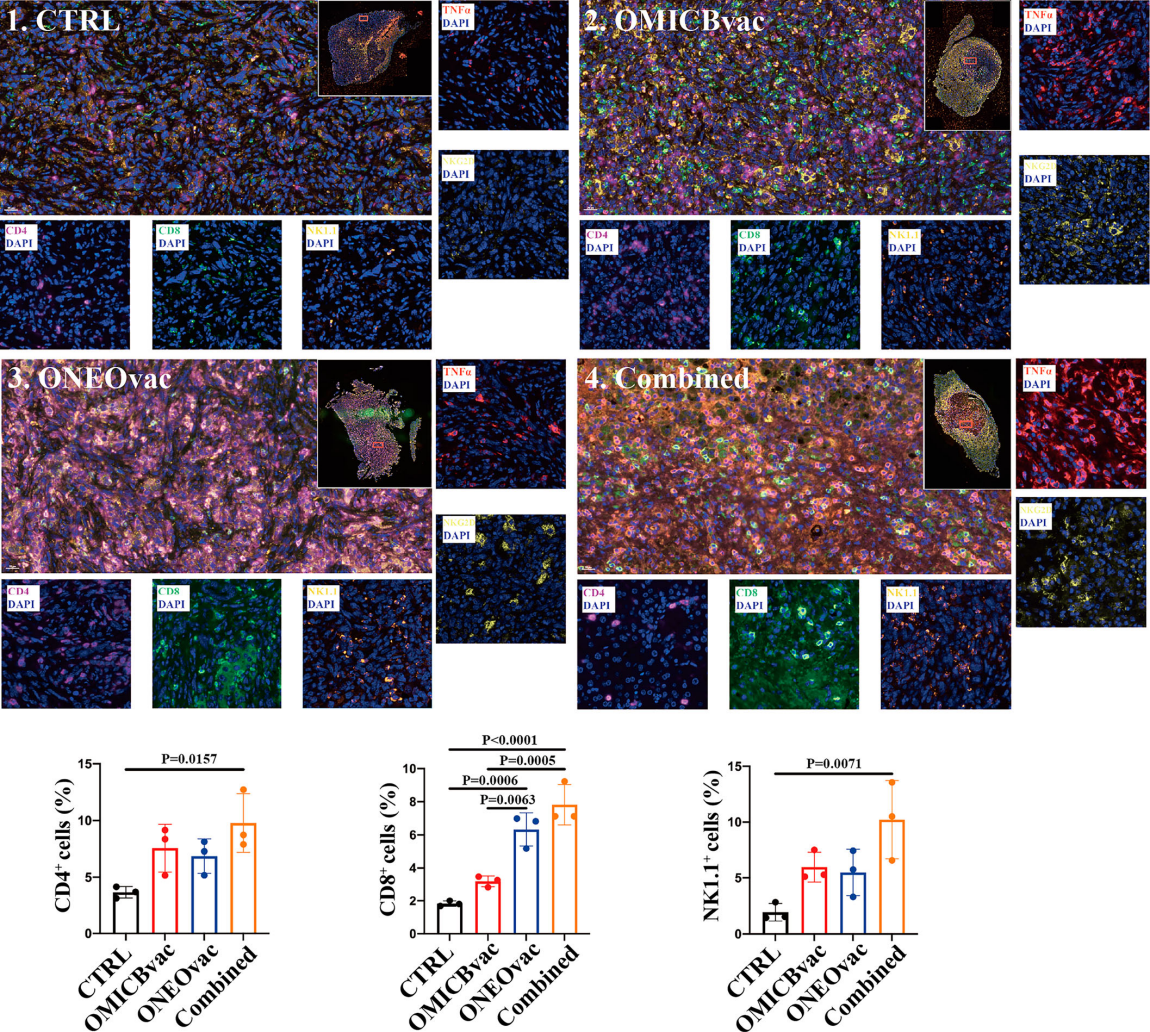
Immune cell infiltration and effector molecule expression in tumors. Representative multi-color immunofluorescence images of CD4^+^ T cells, CD8^+^ T cells, and NK1.1^+^ NK cells, as well as the NKG2D and TNF-α expression in tumors across different treatment groups. Statistical scatter plots illustrate the cell density of CD4^+^ T cells, CD8^+^ T cells, and NK1.1^+^ NK cells across entire tumor sections (*n*  =  3 slides per group, from different mice; one-way ANOVA). Scale bars, 20 μm. Data are presented as the mean ± SEM. *P < 0.05, **P < 0.01, ***P < 0.001, ****P < 0.0001; ns, no significance.

To further characterize the potential anti-tumor immune responses, the number of tumor-infiltrating immune cells were firstly evaluated by using multicolor immunofluorescence staining. As shown in Fig. EV1, the proportions of CD4^+^ T cells, CD8^+^ T cells, and NK1.1^+^ cells were significantly increased in combined treatment, and this phenomenon was further confirmed by flow cytometry (Fig. 3I,J; Appendix Fig. S5F). Furthermore, the proportion of cells expressing NKG2D (MICA/B serve as ligands for activating the NKG2D receptor on T cells and NK cells) were significantly increased in OMICBvac (vs ONEOvac, *P* = 0.1612) and the combined treatment group (vs OMICBvac, *P* < 0.0001) than that in PBS/ONEOvac treated group, which illustrates that under the action of MICB antibodies, the proteolytic shedding of MICB in the tumor cell membrane is inhibited. Moreover, higher proportion of NKG2D positive NK cells/T cells were observed in mice immunized with combined treatment relative to those receiving other three treatment (all *P* < 0.0001, Appendix Fig. S5G). Correspondingly, highest expression of TNF-α in NK cells/T cells were observed in the combined treatment group, showing a strong killing effect of immune cells to tumor cells (vs OMICBvac, *P* = 0.0033, vs ONEOvac, *P* = 0.0024, Appendix Fig. S5H). Moreover, the characterization of tumor-infiltrating T cell activation by flow cytometry also revealed that the expression of 4-1BB (vs OMICBvac, *P *= 0.0081, vs ONEOvac, *P* < 0.0001) on CD8^+^ T cells in the combined treatment were obviously higher than other three groups (Fig. 3K; Appendix Fig. S5I). Meanwhile, flow cytometry analysis using the neoantigen Ptpn2 tetramer also validated that the combined treatment showed three times higher of the infiltration of tumor-specific T cells than the ONEOvac treatment only (vs ONEOvac, *P* < 0.0001, Fig. 3L; Appendix Fig. S5J). Additionally, we employed antibodies to selectively deplete CD8^+^ T cells or NK cells in the orthotopic hepatocellular carcinoma (HCC) model. Our results demonstrated that depletion of either CD8^+^ T cells or NK cells significantly attenuated the combined antitumor efficacy of OMICBvac and ONEOvac, resulting in accelerated tumor progression (Appendix Fig. S6). These findings establish that the synergistic antitumor activity of OMICBvac and ONEOvac is critically dependent on both CD8^+^ T cell and NK cell-mediated immune responses. Moreover, to further demonstrate whether this combination strategy is applicable to other tumors, we established a pancreatic cancer liver metastasis (PCLM) based on the pancreatic cancer cell line Panc02-hMICB cells, and prepared personalized ONEOvac for Panc02 (Cai et al, 2025) and original OMICBvac for combined treatment (Appendix Fig. S7A). As expected, consistent with the findings in the orthotopic HCC model, the combined treatment group induced better anti-tumor efficacy, blood antibody production, and infiltration and activation of neoantigen-specific T cells in the PCLM model compared with the single treatment alone (Appendix Fig. S7B–G). These results strongly demonstrated the anti-tumor effect of OMICBvac combined with ONEOvac treatment.

Adverse events constitute the primary impediment to the clinical implementation of immune strategies. Thus, we evaluated the biosafety of the combination strategy in the Hepa1-6-hMICB orthotopic model by using H&E staining of vital organs and blood biochemical assays. Our findings indicate that our strategy did not induce significant biochemical abnormalities or damage to the heart, liver, spleen, lung, and kidney, suggesting that this strategy represents a potentially safe, feasible, and effective clinical approach for personalized tumor immunotherapy (Appendix Fig. S8A,B).

### Combined therapy enhances antitumor immune response by inducing tumor pyroptosis

**Figure 4:**
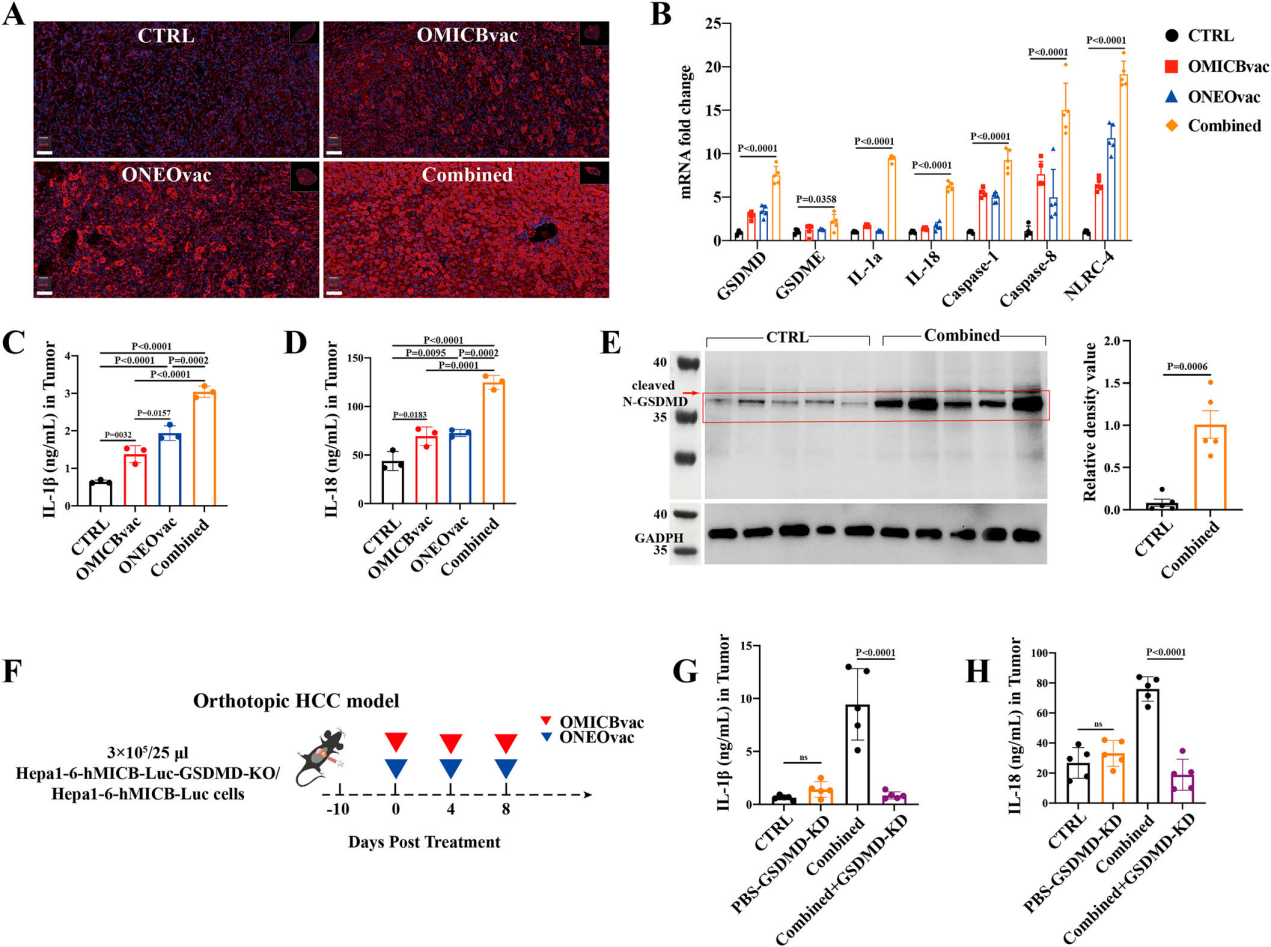
The combined therapy-induced pyroptosis enhances antitumor immune response. (**A**) Immunofluorescence images of calreticulin expression in tumor cells. Scale bar, 50 nm. (**B**) RT-qPCR analysis of relative mRNA expression levels of ICD-related inflammatory genes in tumors (*n* = 5 independent replicates; one-way ANOVA). (**C**, **D**) ELISA measurement of the IL-1β (**C**) and IL-18 (**D**) in tumor (*n* = 3 independent replicates; one-way ANOVA). (**E**) Western blot analysis of GSDMD cleavage in tumors following combined treatment. 50 μg of each sample was loaded for electrophoresis and quantified by Image Lab 5.0 (*n* = 5 independent replicates; one-way ANOVA). (**F**) Schematic representation of the Hepa1-6-hMICB (or GSDMD-KO) orthotopic HCC model and the treatment timeline. After 10 days of tumor cell inoculation, the mice received different treatments, including PBS and the combination of OMICBvac (10 μg) and ONEOvac (10 μg), on days 0, 4, and 8, respectively. After 21 days of treatment, tumors were collected from the mice for subsequent analysis. (**G**, **H**) ELISA measurements of IL-1β and IL-18 levels in tumors from the indicated groups (*n* = 5 mice per group; one-way ANOVA). Each group consisted of 10 mice for reconstructing the tumor model, and after 21 days of treatment, 5 mice were randomly selected for tumor sampling and analysis. Data are presented as the mean ± SEM. *P < 0.05, **P < 0.01, ***P < 0.001, ****P < 0.0001; ns, no significance. Source data are available online for this figure.

The occurrence of immunogenic cell death (ICD) during tumor treatment can increase the immune system’s recognition and attack on tumors, thereby improving the success rate of treatment. When tumor cells undergo ICD, they release a large number of inflammatory cytokines and damage-associated molecular patterns (DAMPs), which could amplify the immune response (Chen and Mellman, 2013, 2017; Li et al, 2020; Pio et al, 2019). To determine whether OMICBvac plus ONEOvac treatment can induce potent ICD in tumor cells, we evaluated the expression of calreticulin (CRT, a marker of ICD) in tumor tissues by immunofluorescence. As shown in Fig. 4A, ONEOvac or OMICBvac alone can induce the expression of CRT protein on the tumor cell membrane; meaningfully, the combined treatment group can induce significant more tumor cell expressing CRT in membrane, suggesting that more tumor cells undergo stronger ICD. ICD manifests in various forms, including apoptosis, ferroptosis, autophagy and pyroptosis, accompanied by inflammatory response. Therefore, we further used Q-PCR to detect the expression of 7 potential pro-inflammatory genes (GSDMD, GSDME, IL1β, IL18, Caspase-1, Caspase-8 and NLRC-4) in the tumor tissues of different treatment groups. As shown in Fig. 4B, almost all inflammatory factors were significantly upregulated in the combined treatment group (GSDME *P* = 0.0358, other all *P* < 0.0001). The main biochemical features of tumor cell pyroptosis include the formation of inflammasomes and the activation of caspases and gasdermin, which lead to the release of a large number of pro-inflammatory cytokines (such as IL-1β and IL-18), thereby activating a strong inflammatory response. Thus, we hypothesized that the combined therapy might induce pyroptosis for positive feedback loop, which created a favorable immunogenic hot tumor microenvironment that sensitizes cancer cells to immunotherapy. The high level of IL-1β and IL-18 in tumor tissues were considered as the indicators of pyroptosis occurrence. As shown in Fig. 4C,D, the level of IL-1β and IL-18 in combined treatment were significantly higher than other three groups (all *P* < 0.0001). More importantly, the cleavage of Gasdermin D (GSDMD), a key event in the occurrence of cell pyroptosis, in the tumor tissues of the combined treatment was clearer observed by western blot when compared with PBS-treated group (*P* = 0.0254, Fig. 4E). Taken together, the combined treatment could ignite the tumor microenvironment by inducing pyroptosis to enhance the efficacy of antitumor immunotherapy.

**Figure EV2:**
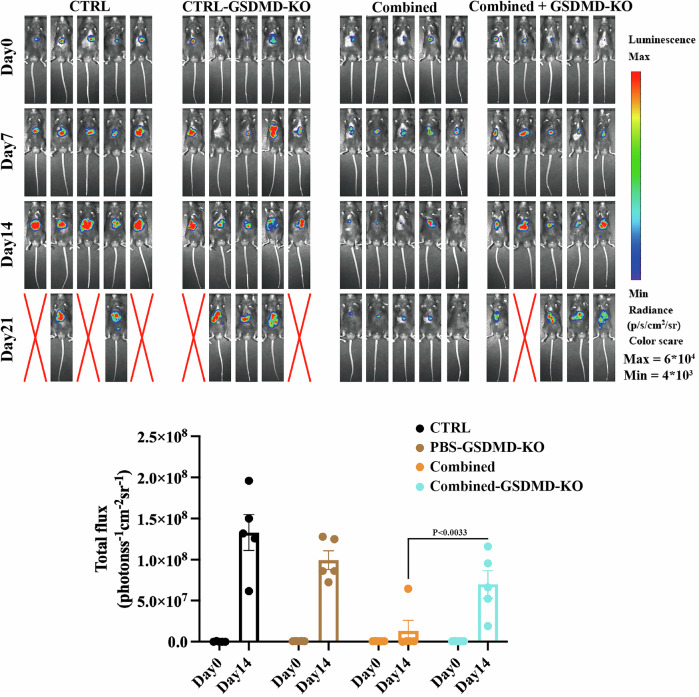
GSDMD-dependent antitumor efficacy of combined vaccination monitored by bioluminescence imaging. Tumor burden monitoring by bioluminescence imaging of mice inoculated with Hepa1-6 or Hepa1-6 GSDMD-KO cells and treated with PBS or the combination of ONEOvac and OMICBvac. Each group consisted of 5 mice, which were used to establish tumor models and monitor tumor growth (*n* = 5 mice per group; two-way ANOVA). Data are presented as the mean ± SEM. *P < 0.05, **P < 0.01, ***P < 0.001, ****P < 0.0001; ns, no significance.

Based on these findings, we speculated that pyroptosis plays an important role during the immunotherapy by the treatment of OMICBvac plus ONEOvac. To confirm this, we knockout the gene of *GSDMD* but not *GSDME* (the expression level is significantly less than that of *GSDMD*) in Hepa1-6-hMICB cells by using CRISPR/Cas9 to restrict the initiation of pyroptosis (Appendix Fig. S9A). In vitro and in vivo experiments demonstrated that GSDMD deletion had no impact on the proliferative capacity of Hepa1-6 cells (Appendix Fig. S9B–D). Then, the Hepa1-6-hMICB cells with or without GSDMD knockout (GSDMD-KO) were used to construct a mouse HCC orthotopic tumor model, and further received the combined therapy (Fig. 4F). As shown in Fig. EV2, the efficacy of orthotopic tumors based on Hepa1-6-hMICB cells with GSDMD-KO showed significantly worse therapeutic efficacy comparing to the tumors based on Hepa1-6-hMICB cells without GSDMD-KO after receiving OMICBvac plus ONEOvac treatment (*p* < 0.0033). Correspondingly, the expression level of IL-1β and IL-18 from the GSDMD-KO tumor tissue in the combined treatment group also both decreased significantly (*P* < 0.0001) when compared with the control tumor tissue and was even not significantly different from the PBS-treated group (Fig. 4G,H). These data indicated that OMICBvac plus ONEOvac treatment may exert its anti-tumor effect mainly by inducing GSDMD-mediated tumor cell pyroptosis.

### Combined therapy improves the immunogenicity of tumor cells

**Figure 5:**
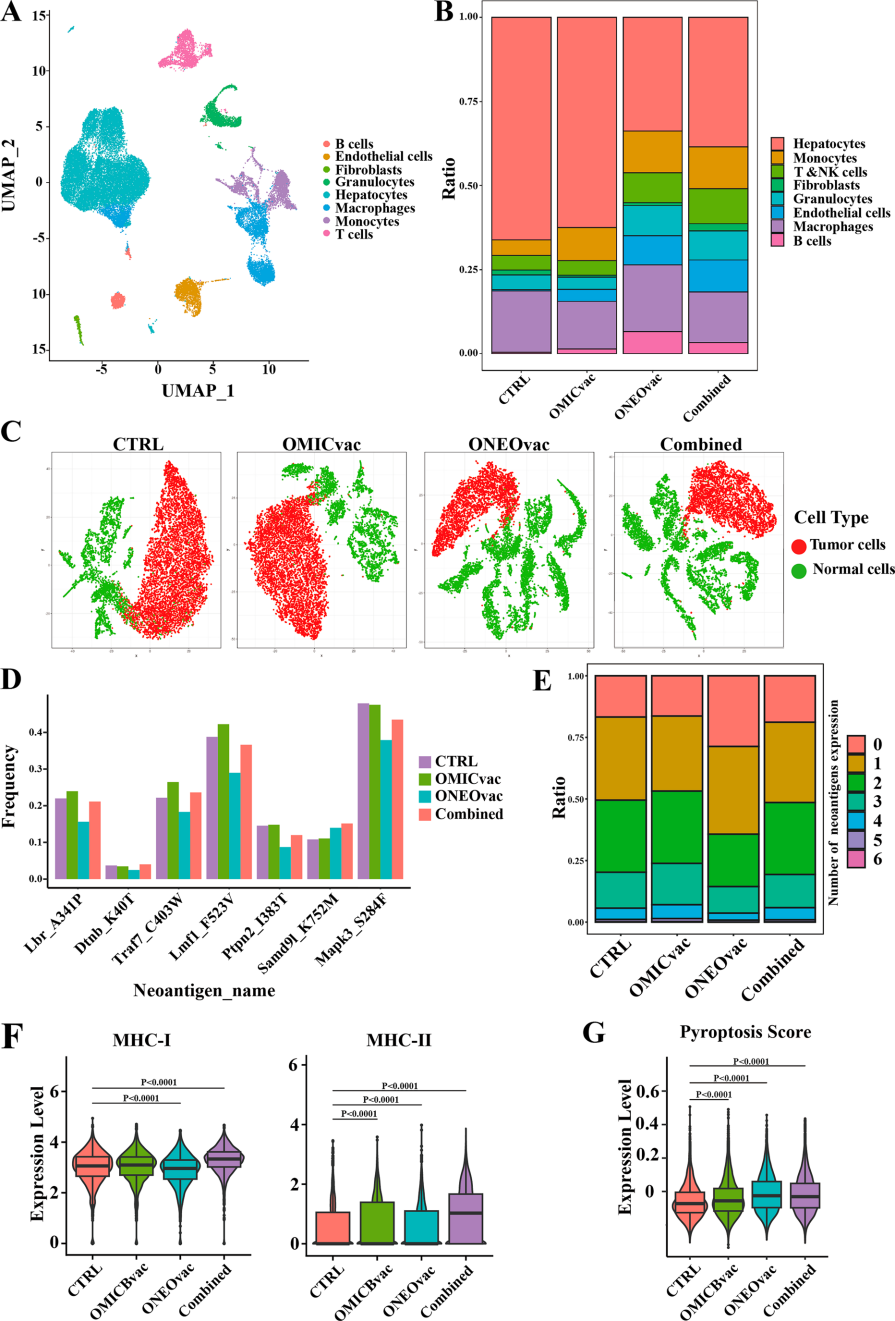
Single-cell RNA sequencing characterizes immune microenvironment changes in the orthotopic HCC tumor mice. (**A**) t-SNE plot showing cells merged from all groups. Data integration and cell clustering were performed with Seurat. (**B**) Histogram indicates the proportion of clusters within each group as indicated. (**C**) Tumor cells identified by copy number analysis and their distribution in each group as indicated. Copy number analysis was conducted using SCEVAN to identify cells with abnormal copy number profiles. (**D**) The difference in expression frequency of different neoantigens in each group as indicated. Cells with genotype of 0/1 or 1/1 identified by target sequencing of corresponding neoantigen derived mutation were classified as expressing this neoantigen. (**E**) The proportion of tumor cells co-expressing different numbers of neoantigen sequences evaluated by target sequencing. (**F**) Expression levels of MHC-I and MHC-II in tumor cells of each group. (**G**) Scoring of pyroptosis-related gene expression by AddmoduleScore in each group of tumor cells. The statistical details (minima, maxima, centre, box bounds, and whiskers from 8000 cells of each sample) for box plots in (**F**, **G**) were added in Appendix Table S4. Data are presented as the mean ± SEM. *P < 0.05, **P < 0.01, ***P < 0.001, ****P < 0.0001; ns, no significance.

To comprehensively explore the changes of immune microenvironment under OMICBvac plus ONEOvac treatment, single cell RNA sequencing together with target sequencing for seven neoantigen mutations (Lbr_A341P, Dtnb_K40T, Traf7_C403W, Lmf1_F523V, Ptpn2_I383T, Samd9l_K752M, Makp3_S284F) was performed for the tumor tissues collected from mice vaccinated by PBS, OMICBvac, ONEOvac and OMICBvac plus ONEOvac on days 0, 4 and 8. After data processing, 8000 cells were randomly selected from the sequencing data and cell clustering was performed. As shown in Fig. 5A, a total of 8 different types of cells were identified with known markers including B cells (CD19, CD79a, CD79b), Endothelial cells (Kdr, Pecam1, Oit3), Fibroblasts (CD34), Granulocytes (Camp, Csf3r), Hepatocytes (Alb, Afp), Macrophages (Adgre1, CD68, Csf1r), Monocytes (Ccr2, Chil3, CD209a), T cells (Klrb1c, CD3e, CD4, CD8a). The cell ratio analysis chart shows that the number of immune cells such as T & NK cells were significantly upregulated in the treatment group, especially in the combined treatment group (Fig. 5B). This finding at the single-cell transcriptome level independently corroborates the enhanced T and NK cell infiltration observed in our experimental validation. To further identify the tumor cell components in hepatocytes, we used SCEVAN by evaluating cells’ copy number profiles. As shown in Fig. 5C, the copy number profiles could discriminate tumor cells from normal cells. Since the ONEOvac vaccine includes seven neoantigen targets, we want to observe the dynamic changes in the mutation frequency of these seven targets in tumor cells in different treatment groups at the single cell level. As shown in Fig. 5D, the seven neoantigen mutation spectrum of OMICBvac was basically consistent with that of the PBS-treated group, suggesting that it induced non-specific tumor killing; the mutation frequency of seven neoantigens in the tumor cells of the ONEOvac treatment group was significantly downregulated compared with the control group and the OMICBvac group, suggesting that ONEOvac can induce T cells to specifically kill tumor cells expressing neoantigen mutation sites. By analyzing the number of neoantigens expressed in one tumor cell, it was found that when tumor cells expressed 2 or more neoantigens, they were more easily killed by ONEOvac-induced T cells, which was consistent with expectations (Fig. 5E). As we expected, the characteristics of the neoantigen mutation expression spectrum in the combined treatment group were intermediate between those of OMICBvac and ONEOvac alone treatment, suggesting that the combined treatment can simultaneously achieve both tumor-specific and non-specific killing. Further characterization of the changes in the expression levels of MHC- and MHC-II in tumor cells in different treatment groups found that the combined treatment can significantly increase the expression of MHC-I and MHC-II in tumor cells, thus significantly enhancing the immunogenicity of the tumor (all *P* < 0.0001, Fig. 5F). Such phenomenon was further confirmed in tumor tissues by flow cytometry (Appendix Fig. S10). The pyroptosis feature scoring of tumor cells also proved that the combined treatment could induce stronger tumor cell pyroptosis (Fig. 5G). These results suggest that the combined treatment can induce tumor cells to exhibit stronger immunogenicity, making them easier to be recognized by immune cells and to perform more extensive tumor killing thereby preventing immune suppression.

### The combined treatment induces significant ILC1 cell infiltration

**Figure 6:**
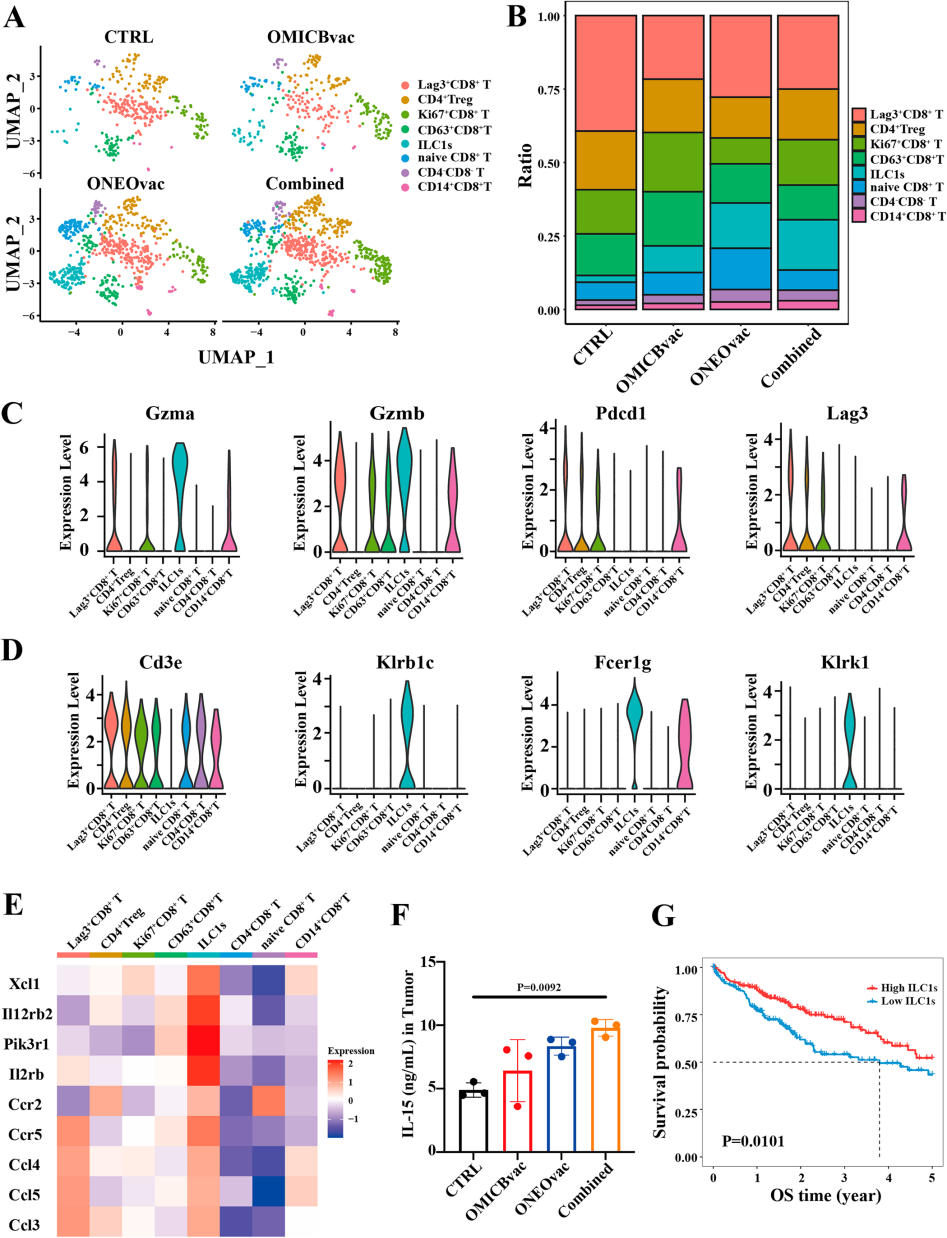
The combined treatment induces infiltration of IL15 armed ILC1 cells. (**A**, **B**) t-SNE plots of the enrichment of different T cell clusters in each group, histogram indicates the proportion of clusters within each group. Cell clustering was performed by Seurat. (**C**) Violin plot shows the expression of T cell function markers. (**D**) Violin plot shows the expression of ILC1s-specific markers. (**E**) Heatmap shows the average expression levels of IL-15 signaling pathway-related marker genes for all T cell subtypes. The average expression matrix was obtained with AverageExpression in Seurat. (**F**) ELISA measurement of the IL-15 in tumor of each group (*n* = 3 mice per group; one-way ANOVA). (**G**) Kaplan–Meier curves of 5-year overall survival for patients with HCC from TCGA database stratified by median expression level of three-gene signature (Klrb1c, Fcer1g and Klrk1). The data analysis was performed with survival packages. Data are presented as the mean ± SEM. *P < 0.05, **P < 0.01, ***P < 0.001, ****P < 0.0001; ns, no significance. Source data are available online for this figure.

Since T cells and NK cells are the main effector cells for killing tumors, T&NK cells were extracted from the sequencing data and re-clustered into 8 clusters, including 5 clusters of CD8 T cells (Lag3^+^CD8^+^ T cells, Ki67^+^CD8^+^ T cells, CD63^+^CD8^+^ T cells, naïve CD8^+^ T cells, CD14^+^CD8^+^ T cells), CD4^+^ Treg, CD3^+^CD4^-^CD8^-^ T cells and ILC1s. It is noteworthy that a higher enrichment of CD8^+^ T cells and ILC1s was observed in the tumor tissue of the combined treatment (Fig. 6A,B; Appendix Fig. S11). Subsequent analyses revealed that the CD8^+^ T cells in this group exhibited elevated expression levels of cytotoxic factors, including GZMA and GZMB, as well as increased expression of cell exhaustion markers such as PD-1 and LAG-3 (Fig. 6C). This suggests that the tumoricidal capacity of CD8^+^ T cells was compromised. Surprisingly, we found that ILC1s cells highly express GZMA and GZMB, and specifically express NK cell-related killing factors such as Fcer1g, Klrblc and Klrk1, while not express exhaustion genes such as PD-1 and Lag-3, suggesting that this group of cells has strong anti-tumor ability and may be the main effector cells of this combination strategy (Fig. 6C,D). ILC1s cells are an important cell group of the innate immune system and participate in the body’s immune surveillance and anti-tumor immunity. So why does combination treatment induce a significant increase in ILC1s in tumor tissues? Several research studies show that IL-15 is an essential cytokine for the development and survival of ILC1s and promotes the secretion of cytokines by ILC1s to exert antiviral and anti-tumor activities (Fuchs et al, 2013; Kansler et al, 2022; Waldmann et al, 2020). To further clarify whether IL-15 is related to the activation of ILC1s, key genes (Xcl1, Il12rb2, Pik3r1, Il2rb, Ccr2, Ccr5, Ccl4, Ccl5 and Ccl3) in IL-15 related pathways were extracted from KEGG pathways (mmu04060: Cytokine-cytokine receptor interaction; mmu04630: JAK-STAT signaling pathway) and the expression of them were evaluated in different T & NK cell clusters. As shown in Fig. 6E, IL-15 signaling pathway-related genes are only highly expressed in the ILC1s group, suggesting that ILC1s are mainly affected by IL-15 regulation. Therefore, we further detected the IL-15 concentration in tumor tissues after 4 different treatments as indicated in Fig. 3A and found that the IL-15 content was significantly increased in the combination treatment comparing with the PBS-treated group (*P* = 0.0092, Fig. 6F). Finally, Kaplan–Meier analysis in 370 cases of TCGA liver cancer data set also revealed that the signature of ILC1s built with NK1.1, CD49a and CD103 expression in liver cancer tissues was significantly related with patients’ over-all survival (OS), and patients with higher expression of the signature of ILC1s showed better OS (*P* = 0.0101, Fig. 6G). These results suggested that the combined treatment can enhance the infiltration and anti-tumor function of IL15-induced ILC1 in tumor tissues.

### IL-15 enhances ILC-mediated pyroptosis for antitumor effects

**Figure EV3:**
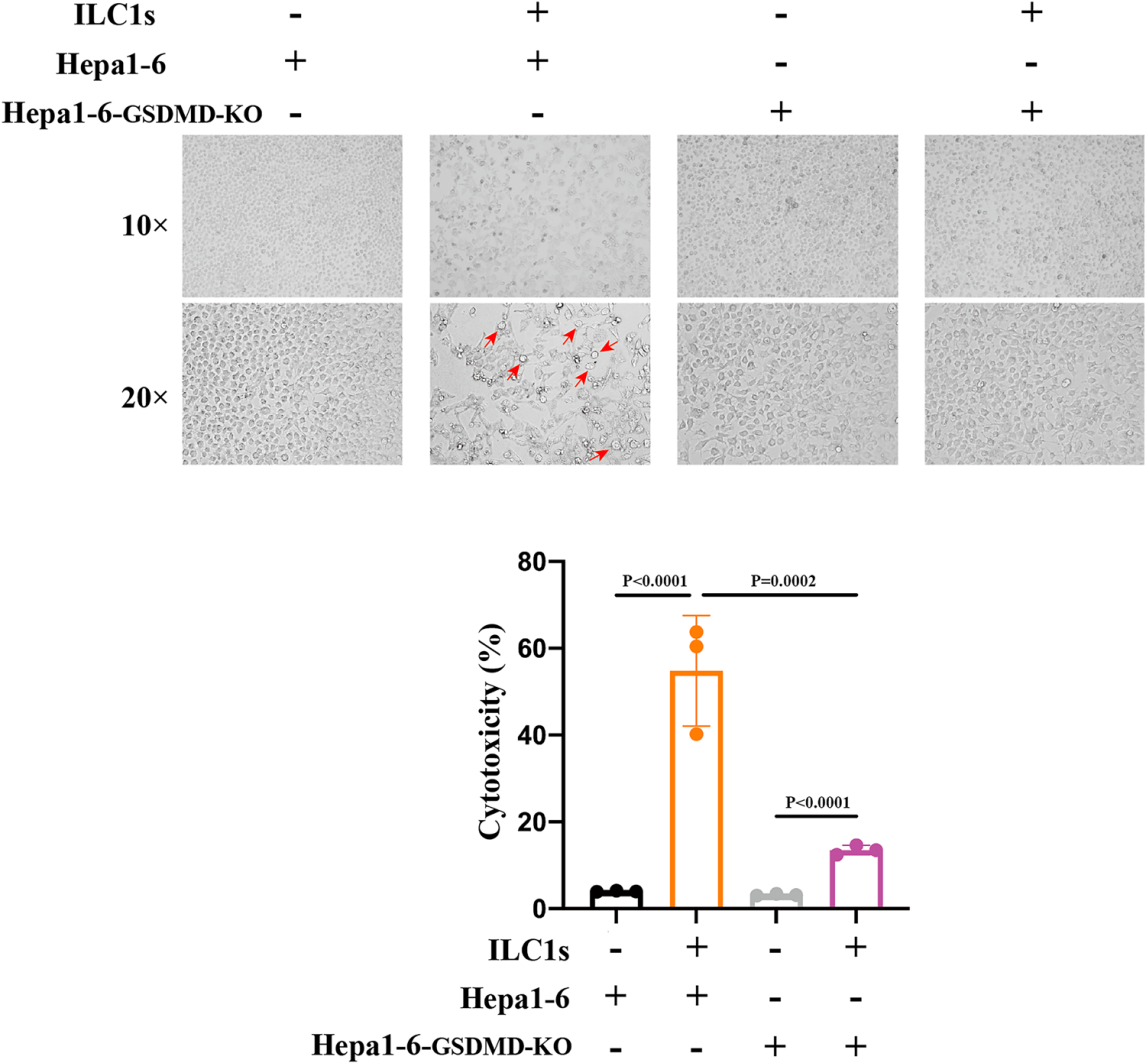
ILC1-mediated cytotoxicity against tumor cells in vitro. Microscopic images of ILC1s co-cultured with Hepa1-6-hMICB (or GSDMD KO) cells after 48 h. 12 days after the combined treatment, fresh tumor tissues from mice were collected to prepare single-cell suspensions and stained with CD49a and CD103 for sorting of the ILC1s by flow cytometry. Then ILC1s were co-cultured with Hepa1-6 cells as indicated. LDH analysis of the cell death percentage induced by ILC1s (*n* = 3 independent replicates; one-way ANOVA). 50 μl of the co-culture supernatant from each group was collected for LDH analysis. Data are presented as the mean ± SEM. *P < 0.05, **P < 0.01, ***P < 0.001, ****P < 0.0001; ns, no significance.

Based on these findings, we further questioned whether ILC1s cells that highly express pyroptosis-inducing factors GZMA and GZMB are effector cells for inducing pyroptosis of tumor cells. To verify this question, we sorted ILC1 cells from orthotopic HCC tumors in mice treated with combined therapy and co-incubated them with Hepa1-6-hMICB or Hepa1-6-hMICB-GSDMD-KO cells in vitro for 48 h, then took pictures and collected the supernatant for LDH detection to analyze the tumor killing effect. As shown in Fig. EV3, characteristic pyroptotic vesicles were obviously observed in Hepa1-6-hMICB cells but not in Hepa1-6-hMICB-GSDMD-KO cells after the co-culturing with ILC1s. Moreover, the LDH analysis revealed that ILC1s possessed the potent tumoricidal activity in vitro, inducing cell death for over 50% from Hepa1-6-hMICB cells. However, when the *GSDMD* gene in Hepa1-6-hMICB cells was knockout, the tumoricidal activity of ILC1s against Hepa1-6-hMICB cells was significantly reduced (*P* = 0.002), indicating that ILC1s primarily induced pyroptosis for tumor cell killing. In addition, this cytotoxicity partially depends on NKG2D but shows high tumor specificity, as ILC1s exhibited minimal activity against normal AML12 hepatocytes (Appendix Fig. S12). This selectivity aligns with the absence of liver damage in our in vivo safety assessment (Appendix Fig. S8), confirming the therapeutic safety and efficiency of vaccine-induced ILC1s.

**Figure 7:**
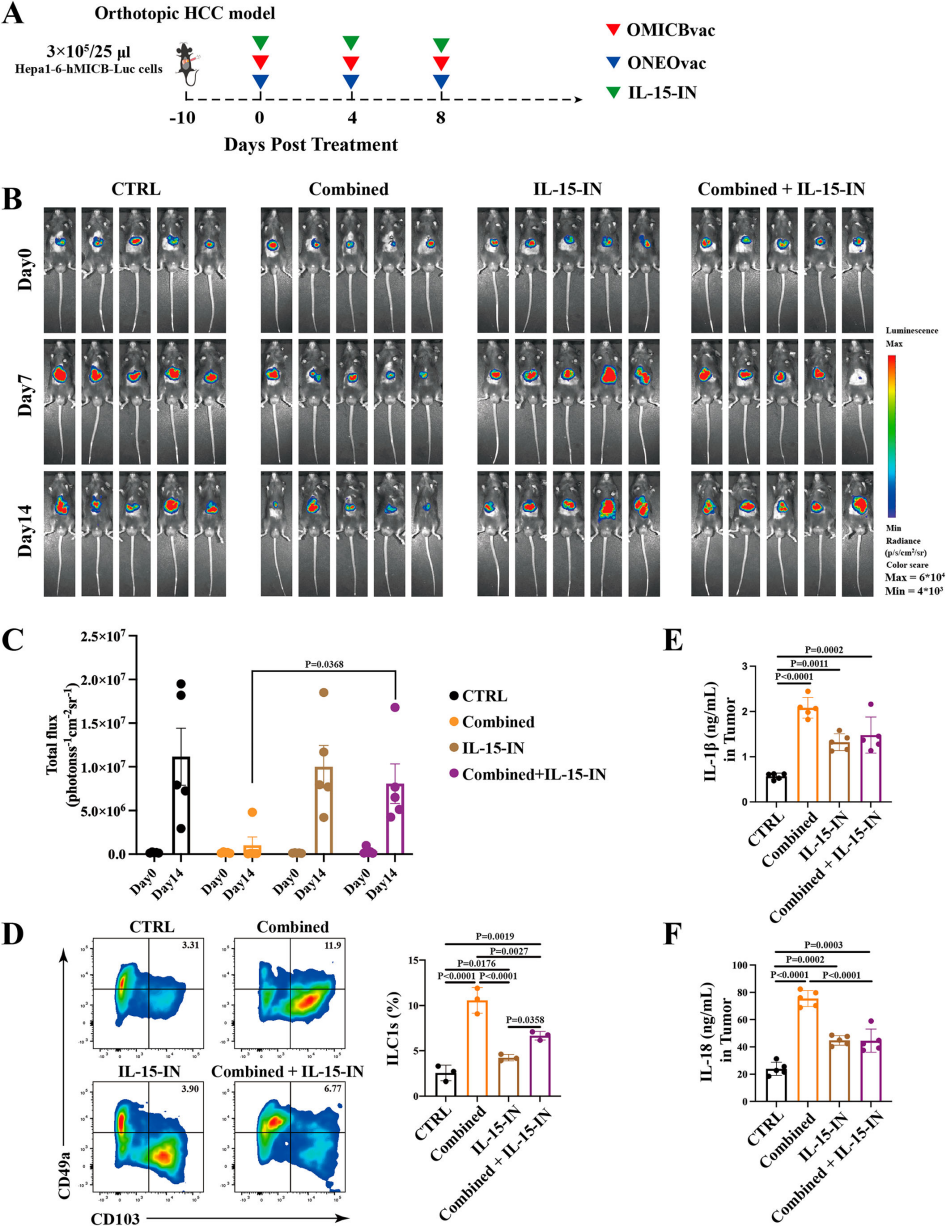
IL-15 enhances ILC-mediated pyroptosis for antitumor effects. (**A**) Schematic representation of the Hepa1-6-hMICB orthotopic HCC model and the treatment timeline. (**B**, **C**) Tumor burden monitoring of mice treated with PBS, the combined therapy (ONEOvac + OMICBvac), IL-15 inhibitor, and the combined therapy plus IL-15 inhibitor, as assessed by bioluminescence imaging (*n *= 5 mice per group; two-way ANOVA). After 14 days of treatment, tumors were collected from the mice for subsequent analysis. (**D**) Flow cytometry analysis of the proportion of ILC1s in mouse tumor tissues (*n* = 3 mice per group; one-way ANOVA). (**E**, **F**) ELISA measurements of IL-1β (**E**) and IL-18 (**F**) levels in tumors (*n* = 5 mice per group; one-way ANOVA). Data are presented as the mean ± SEM. *P < 0.05, **P < 0.01, ***P < 0.001, ****P < 0.0001; ns, no significance. Source data are available online for this figure.

Several studies have demonstrated that generation of ILC1s is dependent on the cytokine IL-15, and single-cell data in this study also showed ILC1s significantly overexpressed IL-15 signal pathway associated genes (Bank et al, 2020; Rodriguez-Rodriguez et al, 2022). Therefore, to further confirm the results in vivo by the orthotopic HCC mouse model, the IL-15 inhibitor was used to reduce the activation level of ILC1s in mice (Fig. 7A). The results showed that after the IL-15 inhibition, tumors became less sensitive to the combined treatment, resulting in decreased antitumor efficacy (vs the combined treatment, *P* = 0.0368, Fig. 7B,C). Flow cytometry results revealed a decrease in the proportion of ILC1s in mouse tumor tissues after treatment with the IL-15 inhibitor (vs the combined treatment, *P *= 0.0027, Fig. 7D), confirming that IL-15 could regulate the activation and infiltration of ILC1s in tumor. Additionally, the expression levels of IL-18 (vs the combined treatment, *P* = 0.0005) in tumor tissues was significantly downregulated, accompanied by a decrease of IL-1β (Fig. 7E,F). This result showed that IL15 armed ILC1s could induce tumor cell pyroptosis and enhance anti-tumor efficacy of OMICBvac plus ONEOvac treatment. Overall, these findings strongly demonstrated that the synergistic therapeutic strategy of ONEOvac and OMICBvac induced the activation of IL15 armed ILC1s population in tumors, leading to tumor cell pyroptosis and thereby enhancing the antitumor immune response.

## Discussion

Tumor-specific immunotherapy targeting unique antigens present on cancer cells is fundamental to the effectiveness of clinical immunotherapy and has significantly advanced the field of clinical cancer treatment. These responses are finely tuned to distinguish cancer cells from normal cells, minimizing damage to healthy tissue while focusing the immune attack on the tumor. Traditionally, tumor-specific immunotherapy has focused on the role of T cells in anti-tumor immunity, with cellular immunity considered dominant. However, recent studies have linked B cells and plasma cell-based humoral immunity to better clinical outcomes in various human cancers, including ovarian, colorectal, and liver cancers (Biswas et al, 2021; Meshcheryakova et al, 2014; Richards et al, 2012; Zhang et al, 2019). Tumor IgG produced by B cells and plasma cells redirects immunosuppressive myeloid cells (such as NK cells) to tumor cells to exert anti-tumor functions (Mazor et al, 2022). Here, we firstly developed a novel combination strategy that combines tumor-specific humoral and cellular immunity in tumor immunotherapy based on the bacterial OMV-based vaccine platform. On the one hand, the tumor personalized neoantigen vaccine (ONEOvac) can generate a large number of neoantigen-specific T cells that infiltrate into the tumor, to induce tumor-specific killing. However, when tumors face specific immune pressure, they can develop immune suppression mechanisms such as downregulating MHC-I and MHC-II expression. These tumor cells gain a survival advantage and become resistant. On the other hand, we employed the shared MICB α3 antigen to induce the production of antibodies against MICB α3 in vivo, which prevents the shedding of MICB from tumor cells, enhancing the broad-spectrum killing sensitivity of NK cells and others to tumor cells, thus preventing immune suppression. Single-cell level data on the antigen expression profiles of tumor cells further confirmed that this strategy can induce more extensive tumor killing and prevent immune suppression.

Furthermore, consistent with our previous neoantigen vaccine studies (Chen et al, 2022; Lin et al, 2024; Zhao et al, 2022), we found that the ONEOvac and combination therapy groups indeed induced a substantial generation and infiltration of T cells in vivo. However, we also observed that these T cells, while expressing effector factors such as GZMA and GZMB, also had high expression of exhaustion-related markers such as PD-1 and LAG-3, suggesting that they are susceptible to becoming exhausted or dysfunctional in the immunosuppressive microenvironment. Additionally, we identified a population of innate lymphoid cells (ILC1s) that showed significantly enhanced infiltration in the combined therapy. ILC1s demonstrated the capability to eliminate tumor cells through the recognition of MICA/B on the tumor cell surface. ILC1s can respond rapidly to precancerous lesions and expand under the pressures of tumor growth and immunotherapy, thus being regarded as unique sentinels of transformed epithelium (Sharma et al, 2017; Topalian et al, 2015). Our single-cell data show that this population of cells co-expresses high levels of the killing effectors GZMA, GZMB, KLRB1, FCER1G, and KIRK1, while exhibiting virtually no expression of Lag-3 and PD-1. This suggests that ILC1s possess potent and sustained antitumor capabilities, which we validated through both in vitro and in vivo experiments. Interestingly, we discovered for the first time that ILC1s could induce the GSDMD cleavage in tumor cells via GZMA and GZMB, eliciting pyroptosis in tumor cells. GSDM-mediated pyroptosis, a form of cell death accompanied by the secretion of multiple inflammatory cytokines, has recently attracted considerable attention as a unique mechanism in cancer immunotherapy (Guerra et al, 2008; Le Floc’h et al, 2007; Raulet et al, 2013). Triggering inflammatory cell pyroptosis via adenoviral or liposome delivery encoding the N-terminal construct of GSDM has been shown to achieve potent antitumor immunity, significantly improve the TME, and increase its sensitivity to other immunotherapies (Li et al, 2023). Shao et al found that, in addition to the common caspase family proteins, cleavage of GSDM family proteins by GZMA/GZMB can also induce pyroptosis (Zhou et al, 2020). This is consistent with our findings that ILC1s induce pyroptosis. These interesting observations suggest that through the combined action of ONEOvac and OMICBvac, despite the exhaustion of effector CD8^+^ T cells in the tumor microenvironment, the activity and tumor recognition capability of ILC1s are significantly enhanced by the MICB α3 antibody blocking. By inducing pyroptosis in tumor cells by ILC1s, the antitumor immune response is amplified to relieve tumor immune suppression, thus achieving better therapeutic outcomes.

Concurrently, this study also has several limitations: (1) The orthotopic tumor model employed primarily utilizes murine hepatocellular carcinoma cell lines engineered to overexpress human MICA/B proteins, which serves to simulate MICA/B shedding and investigate the efficacy and mechanisms of combinatorial immunotherapy. In future validation phases, further validation of the therapeutic efficacy and underlying mechanisms will be necessary through studies utilizing human patient-derived organoids (PDOs) or properly designed clinical trials; (2) Although no autoimmunity phenomena were observed with our combined immunotherapeutic strategy in this study, this potential risk warrants careful consideration in future clinical investigations. This concern arises primarily because neoantigen vaccine-based immunotherapy cannot completely eliminate the risk of autoimmunity, as neoantigen-specific T cells may exhibit cross-reactivity with wild-type (non-mutated) self-antigens. (3) Despite achieving considerable efficacy, our combined immunotherapeutic strategy did not completely eliminate all tumors, primarily due to persistent immunosuppressive mechanisms within the tumor microenvironment, such as PD-1 pathway activation. Therefore, further strategic combinations incorporating tyrosine kinase inhibitors (TKIs) and immune checkpoint inhibitors represent promising approaches to enhance vaccine efficacy without significantly increasing adverse effects. (4) Our biosafety assessment was limited to measuring ALT and AST levels at the peak of immune activation (day 12), without longitudinal sampling in long-term, tumor-free survivors. While normal liver histology at euthanasia (Appendix Fig. S8A) strongly suggests that the early enzyme elevations were transient and non-pathological, future studies should include longitudinal serum sampling to formally confirm reversibility.

In summary, personalized neoantigen vaccines combined with shared MICB α3 antigen based on OMVs can simultaneously activate tumor-specific cellular and humoral immunity to improve anti-tumor efficacy. Specifically, CD8^+^ T cells and NK cells are crucial for initial antigen-specific recognition and tumor cell killing, potentially through classical cytotoxic pathways, while the recruited ILC1s act as specialized effectors that amplify the immune response by initiating the pyroptotic cascade. Overall, our proposed therapeutic strategy, integrating cellular and humoral immunity, offers a new perspective for cancer immunotherapy in clinical.

## Methods

**Table:** Reagents and tools table

Reagent/resource	Reference or source	Identifier or catalog number
**Experimental models**		
Hepa1-6 (*M. sapiens*)	ATCC	Cat #CRL-1830
Panc02 (*M. sapiens*)	IMMOCELL	Cat # IM-M184
C57BL/6 mice (*M. musculus*)	Shanghai Silaike Experimental Animal Co., Ltd.	N/A
**Recombinant DNA**		
pET28a-ClyA-Hepa1-6(Neoantigens)-Fc	Chen et al, 2022; Cheng et al, 2021	N/A
pET28a-ClyA-Panc02(Neoantigens)-Fc	Cheng et al, 2021	N/A
pET28a-ClyA-MICA (α3)	Ferrari de Andrade et al, 2018	N/A
pET28a-ClyB-MICB (α3)	Ferrari de Andrade et al, 2018	N/A
pET28a-ClyA-MICA (a3)-SHNTQQ(del)	Wang et al, 2025	N/A
**Antibodies**		
Anti-HA antibody	Abcam	Cat #ab314237
Anti-GSDMD antibody	Abcam	Cat #ab219800
Anti-GAPDH antibody	Abcam	Cat #ab8245
Anti-Human IgG Fab fragment antibody	Abcam	Cat #ab771
Anti-CD3-FITC	BioLegend	Cat #100204
Anti-CD8-APC	BioLegend	Cat #140410
Anti-CD4-PE	BioLegend	Cat #100408
Anti-CD11c-APC	BioLegend	Cat #117310
Anti-CD80-PE	BioLegend	Cat #104708
Anti-CD86-PE-Cyanine7	BioLegend	Cat #105014
Anti-CD44-PE-Cyanine7	BioLegend	Cat #103030
Anti-CD62L-PerCP/Cy5.5	BioLegend	Cat #104430
Anti-MICA/B	BioLegend	Cat #320906
Anti-MHC-I	BioLegend	Cat #2102629
Anti-CD49a	Biolegend	Cat #142605
Anti-CD103	Biolegend	Cat #110903
Anti-mouse CD8a	Leinco Technologies, Inc.	Cat #C380
Anti-mouse NK1.1	Leinco Technologies, Inc.	Cat #N123
Ptpn2-specific [Ptpn2_376-384_(RWLYWQPTL):H-2Kb] tetramer-PE	Cancer Research Center of Xiamen University	N/A
Slc16a13-specific [Slc16a132_234-241_ (YVHLVANL):H-2Kb] tetramer-PE	Cancer Research Center of Xiamen University	N/A
Anti-mouse CD4 (IHC)	Abcam	Cat #ab217344
Anti-mouse CD8 (IHC)	Abcam	Cat #ab183685
Anti-mouse NK1.1 (IHC)	Abclonal	Cat #A25457
Anti-Calreticulin	Abcam	Cat #ab315210
Anti-mouse CD4 (IF)	Abcam	Cat #ab183685
Anti-mouse CD8 (IF)	Abcam	Cat # ab217344
Anti-mouse-NK1.1 (IF)	Abclonal	Cat #A25457
Anti-mouse NKG2D (IF)	GeneTex	Cat #GTX50988
Anti-mouse TNF-α (IF)	CST	Cat #11948
**Oligonucleotides and other sequence-based reagents**		
qPCR primer	This study	“Methods”
GSDMD-sgRNA1/2	This study	“Methods”
**Chemicals, enzymes and other reagents**		
FastDigest restriction enzymes	Thermo Fisher Scientific	N/A
T4 ligase	Thermo Fisher Scientific	Cat #EL0014
DMEM	Thermo Fisher Scientific	Cat #C11995500BT
RPMI-1640	Thermo Fisher Scientific	Cat #C11875500BT
Puromycin	Shanghai Genechem Co., Ltd	N/A
G418	Shanghai Genechem Co., Ltd	N/A
IL-15 inhibitor	MedChemExpress	Cat #HY-102049
Human MICA/MICB ELISA kit	Boster Biological Technology	Cat #EK0812/EK0963
Mouse IL-1β ELISA kit	Boster Biological Technology	Cat #EK0394
Mouse IL-18 ELISA kit	Boster Biological Technology	Cat #EK0433
Mouse IL-15 ELISA kit	R&D Systems	Cat #NBP3-06797
Taq Pro Universal SYBR qPCR Master Mix	Vazyme Biotech	Cat #Q712-02
Bovine serum albumin	Sigma-Aldrich	Cat #V900933-1KG
DAPI	Beyotime Biotechnology	Cat #C1002
Matrigel	Corning Life Sciences	Cat #3330624
**Software**		
Image Lab 5.0	https://www.bio-rad.com/zh-cn/product/image-lab-software?ID=KRE6P5E8Z	N/A
GraphPad Prism software V 9.0.0	https://www.graphpad.com/	N/A
Snapgene 6.1.1	https://www.snapgene.com/updates/snapgene-6-1-1-release-notes	N/A
Flowjo v.10	https://www.flowjo.com/	N/A
**Other**		
Cell Counting Kit (CCK-8)	Yeasen Biotechnology	Cat #40203ES76
TransZol Up Plus RNA Kit	TransGen Biotech	Cat #ER501-01-V2
Hifair® Ⅱ 1st Strand cDNA Synthesis Kit	Yeasen Biotechnology	Cat #11121ES60
LDH cytotoxicity assay kit	Beyotime Biotechnology	Cat #C0017
ELISpot Plus: Mouse IFN-γ (ALP)	Mabtech	Cat #3321-4APT-2
Applied Biosystems 7500 Real-Time PCR System	Thermo Fisher Scientific	N/A
ELISpot Analysis System	Antai Yongxin Medical Technology	Cat #AT-Spot2200
IVIS® Spectrum in Vivo Imaging System	PerkinElmer	N/A
6-Plex Detection Kit	AiFang Biological	Cat #AFIHC027

### Mice and cell lines

Male C57BL/6 mice aged at 6-8 weeks were purchased from Shanghai SLAC Laboratory Animal Co., Ltd. (China) and raised under specific pathogen-free (SPF) conditions. The mouse Hepa1-6 and Panc02 cell lines were purchased from ATCC and IMMOCELL authenticated by STR profiling, and both of the cell lines were tested negative for mycoplasma (MycoSEQ Mycoplasma Detection Kit, Thermo Fisher Scientific). Both of the cell lines were transfected with lentivirus (Shanghai Genechem Co., Ltd) to express the human MICA/B gene and luciferase reporter gene: Hepa1-6-hMICA/B-Luc and Panc02-hMICB-Luc. The positive cells were selected by puromycin (Shanghai Genechem Co., Ltd) and G418 (Shanghai Genechem Co., Ltd). All these cells were cultured in DMEM containing 10% FBS and 1% penicillin/streptomycin at 37 °C in a humidified environment with 5% CO_2_. All the cells were confirmed mycoplasma free by using a MycoAlert mycoplasma detection kit (Transgene). The cell lines used in this study were passaged less than 10 times after thawing to ensure their optimal growth and stability.

### Plasmid construction

The gene sequence of *E. coli*-derived ClyA, HA tag, neoantigens (from Hepa1-6 or Panc02 cell line), and mouse immunoglobulin G (IgG; Fc) were cloned together at the downstream of the T7 promoter in pET28a vector to get pET28a-ClyA-3×HA-Neoantigens-Fc plasmid. The sequence of Neoantigens-Fc in pET28a-ClyA-3×HA-Neoantigens-Fc was replaced by the human-derived MICA/B α3 to get pET28a-ClyA-3×HA-MICA/B α3. All the sequences were listed in Appendix Tables S1 and  S2. These plasmids were transformed into *E. coli* (strain Rosetta) for OMV generation. The expression of ClyA-3×HA-Neoantigens-Fc (ONEOvac) and ClyA-3×HA-MICA/B α3 (OMICAvac and OMICBvac) were confirmed using anti-HA tag (ab314237, Abcam, 1:1000 dilution) by western blot.

### ONEOvac and OMICBvac preparation

ONEOvac and OMICBvac were prepared from transformed *E. coli* with pET28a-ClyA-3×HA-Neoantigens-Fc or pET28a-ClyA-3×HA-MICB α3. Briefly, positive transformants were picked and pre-cultured in 5 ml LB medium with 50 μg/mL kanamycin overnight at 37 °C. Then 1 mL LB medium was inoculated into 100 mL fresh LB medium for ∼4 h at 37 °C with shaking (220 rpm) to reach an OD_600_ value of ∼0.6. A total of 0.5 mM IPTG was added into LB medium to induce protein expression, and the temperature was changed to 16 °C for overnight culture with shaking (160 rpm). After culture, the bacteria were pelleted by centrifugation at 8000 × *g* for 10 min at 4 °C. The supernatant was filtered by a 0.45 μm polyvinylidene fluoride filter (Millipore, R8SA47939) and another 0.22-μm pore size filter was used for second time filtration. The ONEOvac or OMICBvac were further collected by ultracentrifugation at 150,000 × *g* at 4 °C for 3 h from the supernatant. The products after ultracentrifugation were finally resuspended in 1 mL PBS and stored at −20 °C until use. The total protein concentration was analyzed by quantitative analysis using BCA analysis kit following the manufacturer’s instructions (Tiangen, China). After reaction, the absorbance was measured at 562 nm using Spectramax m5e (Molecular Devices, USA). Protein concentrations were calculated based on a standard curve generated using bovine serum albumin (BSA) standards (Appendix Fig. S13).

### Characterization of ONEOvac and OMICBvac

The morphology of ONEOvac and OMICBvac were characterized using transmission electron microscopy (TEM, Tecnai G2 F20 U-TWIN, FEI, USA). Briefly, 10 μl of ONEOvac or OMICBvac were dropped onto carbon-coated copper grids for drying. After removing the residual fluid using a piece of filter paper, the samples were negatively stained with 2% uranyl acetate for 5 min and examined by TEM. The hydrodynamic size of ONEOvac or OMICBvac were measured by dynamic light scattering (Zetasizer Nano ZS90, Malvern Instruments, UK).

### In vivo tumor models and evaluation of anti-tumor efficacy

The HCC subcutaneous tumor model was constructed by subcutaneously injecting 3 × 10^6^ Hepa1-6-hMICB cells into the right axilla of mice. The tumor burden was monitored every 2 days with a vernier caliper. The tumor volume was calculated using the formula: V = AB^2^/2 (A is the long diameter and B is the short diameter). When the tumor volume reached 50 mm^3^ (recorded day 0), the mice were randomly divided into 4 groups (*n *= 5) and treated with PBS, OMVs, ONEOvac and OMICBvac in day 0, 4, 8, respectively. The amount of ONEOvac or OMICBvac injected into each mouse was 10 μg total protein. The orthotopic HCC model was constructed by injecting 3 × 10^5^ Hepa1-6-hMICB-Luc cells mixed with Matrigel plugs at the liver subcapsular of mice. The liver metastasis model was constructed by injecting 3 × 10^5^ Panc02-hMICB-Luc cells mixed with the Matrigel plugs by portal vein injection. Tumor burdens of the models were monitored once a week by using the IVIS Spectrum Animal Imaging System (PerkinElmer, USA). The antibodies, anti-Mouse CD8a (Ly 2.2, Leinco Technologies, USA, RRID: AB_2737483) and anti-Mouse NK1.1 (Clone PK136, Leinco Technologies, USA, RRID: AB_2737553) were used to deplete CD8^+^ T cells and NK cells in the orthotopic hepatocellular carcinoma (HCC) mode. The IL-15 inhibitor (IL-15-IN-1, MCE) was administered on days 0, 4, and 8, with each mouse receiving a dose of 50 µg. After 7 days of injecting tumor cells, the mice were randomly divided into 4 groups and received corresponding treatment regimens, and the tumor size was monitored weekly.

### Recurrence rechallenges

The mice completely cured in the above-mentioned combined treatment group were used to construct the HCC recurrence model. At the end of the monitoring, 1 × 10^5^ Hepa1-6-hMICB-Luc cells were injected at liver subcapsular of the completely cured mice with combined treatment or naïve mice, and the tumor size was monitored weekly using the IVIS Spectrum Animal Imaging System (PerkinElmer, USA).

### Safety evaluation

To evaluate the biosafety of ONEOvac and OMICBvac, serum of subcutaneous HCC bearing mice treated with PBS, OMVs, ONEOvac and OMICBvac were collected at the day 12 after treatment and measured by an automated analyzer for biochemical indices (AU5800, Beckman Coulter, USA). Heart, liver, spleen, lung and kidney samples were fixated, and H&E stained to assess pathological changes.

### Generation of knockout cell lines using CRISPR-Cas9 technique

GSDMD knockout Hepa1-6 cells were generated by CRISPR-Cas9, followed by monoclonal selection. Briefly, at room temperature, Cas9 protein (10 µg) was incubated with GSDMD-sgRNA1/2 (total 9 µg) for 15 min. The GSDMD-sgRNA sequence by CRISPR-Cas9 was listed in the following: (1) CTGCAACAGCTTCGGAGTCG; (2) GGAGTTGAGACAACAGATAC. Hepa1-6 cells (1 × 10⁶) were then resuspended in 20 µl of electroporation buffer, mixed with the Cas9/sgRNA complex, and transferred into the electroporation cuvette. The cuvette was immediately placed in the Celetrix electroporator (Celetrix Biotechnologies, Manassas, USA) for electroporation under voltage of 540 V. The cells were slowly extracted and placed into pre-warmed culture medium at 37 °C. After monoclonal selection, western blots were used to validate the expression of GSDMD.

### Single-cell RNA library preparation and sequencing

Fresh tumor tissues were obtained from mice with HCC orthotopic tumors that received different treatments as indicated (PBS, OMICBvac, ONEOvac and Combined) and digested into single-cell suspensions with 2 mL of sCelLiveTM Tissue Digestion Solution (Singleron, China). The cell viability was assessed with trypan blue and was ensured to be above 80%. The cell concentration was adjusted to 3 × 10^5^ cells/mL with phosphate-buffered saline (PBS, HyClone, Marlborough, MA, USA), then single cells were isolated, and mRNA was captured in the Singleron Matrix single-cell processing system. Subsequently, the FocuSCOPE Single Cell Multiomics mRNA x Mouse Liver Mutation Detection Kit (Singleron, China) was used to construct scRNA-seq libraries and single cell target RNA sequencing simultaneously, and the libraries that met the quality standards were sequenced on the Illumina HiSeq 6000 platform (150 bp paired-end reads). The target sequencing panel included the seven neoantigen derived mutations (Appendix Table S1).

### Single-cell RNA sequencing data analysis and corresponding target sequencing

Raw reads obtained from single-cell RNA sequencing was first processed by Celescope (https://github.com/singleron-RD/CeleScope) and single-cell expression matrices were generated using GRCm38 as the reference genome. R package Seurat was used to process single cell data. In total, 8000 cells of each sample were randomly selected for following analysis and R package Harmony was deployed to perform single cell data integration as well as batch effect removal. After cells were clustered using Seurat, the cell type annotation was conducted by R package SingleR and then manually modified by evaluating genes specifically expressed in different clusters of cells.

To identify tumor cells from normal cells, copy number analysis was first conducted using SCEVAN (https://github.com/AntonioDeFalco/SCEVAN). Then, cells that were identified as tumor cells in SCEVAN and as Hepatocytes in cell annotation were finally identified as tumor cells for downstream analysis. Corresponding target sequencing data containing genotype information of 7 neoantigen-derived mutations were added to the metadata of single cell data, and the cellular frequency of each neoantigen was identified as potion of cells with mutated allele (https://github.com/singleron-RD/CeleScope). Seurat’s AddModuleScore function was used to evaluate the expression level of selected pathways in tumor cells in different samples. T cells identified by cell type annotation were further extracted and subjected to similar analysis pipeline to identify different subsets of T cells with each subset annotated based on their specifically expressed genes. AverageExpression function of Seurat was used to get the overall expression of selected genes in different subset of T cells.

Single cell target RNA sequencing data was also processed with Celescope to obtain single-cell level mutation distribution information. The cell barcodes of Single cell target RNA sequencing data were intersected with cell barcodes from single-cell RNA sequencing data to remove cells without expression information before downstream analysis. Genotypes of seven neoantigen derived mutations were identified and the cells with genotype 0/1 or 1/1 was considered as expressing corresponding mutations.

### mRNA isolation and quantitative PCR

Total RNA was extracted from tumor samples using TRIzol Reagent (Invitrogen, Carlsbad, CA, USA) according to the manufacturer’s instructions. A total of 1 μg of RNA was used as a template for single-strand cDNA synthesis. Quantitative PCR (Q-PCR) for GSDMD (For: CCATCGGCCTTTGAGAAAGTG, Rev: ACACATGAATAACGGGGTTTCC), GSDME (For: TGCAACTTCTAAGTCTGGTGACC, Rev: CTCCACAACCACTGGACTGAG), IL1β (For: GCAACTGTTCCTGAACTCAACT, Rev: ATCTTTTGGGGTCCGTCAACT), IL18 (For: GACTCTTGCGTCAACTTCAAGG, Rev: CAGGCTGTCTTTTGTCAACGA), Cacpase-1 (For: ACAAGGCACGGGACCTATG, Rev: TCCCAGTCAGTCCTGGAAATG), Caspase-8 (For: TGCTTGGACTACATCCCACAC, Rev: TGCAGTCTAGGAAGTTGACCA) and NLRC-4 (For: ATCGTCATCACCGTGTGGAG, Rev: GCCAGACTCGCCTTCAATCA) was performed on an Applied Biosystems 7300 Sequence Detection System (Applied Biosystems, Foster City, CA, USA) using SYBR (Vazyme Biotech, China). The PCR program consisted of an initial denaturation at 95 °C for 10 min, followed by 40 cycles of 95 °C for 15 s, 60 °C for 30 s, and 72 °C for 30 s. Q-PCR data were analyzed using the 2^-ΔΔCt^ method. All reactions were performed in triplicate.

### Western blot analysis

The protein expression of OMVs was analyzed by Western blot analysis. All samples were lysed in UREA buffer (8 M Urea, 1 M Thiourea, 0.5% CHAPS, 50 mM DTT and 24 mM Spermine) for 15 min. After centrifugation at 12,000 × *g* for 15 min, the supernatant with protein was collected. The protein samples were loaded onto a 10% SDS/PAGE for electrophoresis and transferred into PVDF membranes (Roche, Mannheim, Germany). Membranes were blocked in Tris-buffered saline with Tween-20 (TBST: 1 mM Tris-HCl, 150 mM NaCl, 0.05% Tween-20, pH 7.4) containing 5% BSA for 0.5 h and subsequently incubated overnight at 4 °C with diluted primary antibody against HA-tag (ab314237, Abcam, 1:1000 dilution), GSDMD (ab219800, Abcam, 1:600 dilution) and GADPH (ab8245, Abcam, 1:10,000 dilution). After washing three times using TBST, the membrane was incubated with HRP-conjugated goat anti-rabbit IgG or HRP-conjugated goat anti-mouse IgG for 1 h at room temperature. The bands were visualized using the FluorChem FC2 system (Alpha Innotech Corporation, St. Leonardo, CA, USA). The data was quantified using ImageJ software.

The anti-MICA-α3 antibodies (1D5 and 13A9, detected by Anti-Human IgG Fab fragment antibody, ab771, Abcam, 1:1000 dilution) were synthesized by GenScript (Jiangsu, China). The sequence of the antibodies was listed in Appendix Table S3, and the purification of the antibodies was analyzed by SDS-PAGE. Briefly, the purified proteins were loaded on to a 10% native PAGE for electrophoresis, then the gel was stained by EZBlue for 30 min and washed by water according to its protocol.

### Enzyme-linked immunospot (ELISPOT) assay

The IFN-γ secretion of mouse splenic T cells were detected by Mouse Interferon-γ ELISPOT Kit (Mabtech, 4APT-10) as described previously (22). Briefly, bone narrow cells were flushed from the femurs and tibias of 8 weeks C57BL/6 mice, and cultured in RPMI 1640 supplemented with 10% FBS, 10 ng/mL IL-4, 20 ng/mL GM-CSF at 37 °C with 5% CO_2_. After 6 days, 3 × 10^4^ bone narrow-derived dendritic cells (BMDCs) were pulsed with neoantigen peptide pool (4 µg in total, 0.55 µg per peptide) or each peptide (4 µg) for 48 h in a multiscreen 96-well filtration plate for 48 h. After three times immunization by neoantigen peptides, 3 × 10^5^ splenic T cells from mice were isolated and added to the 96-well plate for 24 h co-culture with BMDCs. Meanwhile, the BMDCs pulsed with PBS served as the negative control, and splenic T cells co-incubated with CD3 antibody for T cell activation served as the positive control. Then, the plates were washed and subsequently incubated with detection antibody (R4-6A2-biotin, 1 µg/mL, 100 µl/well) for 2 h at room temperature. Afterwards, the plates were washed again and then incubated with Streptavidin ALP (1:1000 dilution, 100 µl per well) for 1 h at room temperature. Subsequently, 3, 3’, 5, 5’-Tetramethylbenzidine (TMB) substrate solution was added to each well and incubated for 4–8 min at room temperature before adding deionized water to stop the reaction. Finally, IFN-γ spot-forming cells were imaged and analyzed by ELISPOT Analysis System (AT-Spot-2200, Beijing Antai Yongxin Medical Technology, China).

### Antigen-specific IgG analysis and ELISA

The antigen-specific IgG titers in mouse serum induced by OMICA/Bvac were assessed using a semi-quantitative ELISA. 96-well plates (Nunc) were coated with 100 µl per well of 0.5 µg recombinant MICA/MICB protein (HY-P70157/P73294, MedChemExpress, USA) prepared in PBS. After overnight incubation at 4 °C, the plates were washed three times with PBS-Tween-20 (0.05% v/v) and blocked for 1 h at 37 °C with 200 µl per well of blocking buffer (1% BSA w/v in PBS-Tween-20, 0.05% v/v). The diluted mouse serum was then added in the plates and incubated for 30 min. After another three-time washing by PBS, 1:1000 dilution of goat anti-mouse IgG/HRP (SE31, Solarbio, China) were incubated for 1 h at 37 °C. After final washing, plates were developed using 50 µl per well of TMB (3, 3′, 5, 5′-tetramethylbenzidine) substrate, and the reaction was stopped after 5 min with 50 µl per well of stop solution (Boster Biological Technology, Wuhan, China). The absorbance was read at 450 nm using Spectramax m5e (Molecular Devices, USA).

To detect the effect of anti-human MICA/B α3 antibody in inhibiting the shedding of MICB protein on the surface of tumor cells, 8 weeks C57BL/6 mice were pre-immunized three times with OMICA/Bvac, and the mouse serum was collected and added to the medium for the coculture of Hepa1-6-hMICA/B cells. Then, the cell culture supernatant was collected at 24 h, 48 h and 72 h, and the shed MICA/B protein content in the supernatant was detected by Human MICA/MICB ELISA kit (EK0812/EK0963, Boster Biological Technology, Wuhan, China) following manual.

Additionally, to assess cytokine levels in the tumors, the collected tumors of equal weight were lysed in the same volume of RIPA lysis buffer containing 1% phenylmethylsulfonyl fluoride (PMSF) and 2% protease inhibitor cocktail. The samples were then homogenized with grinding beads using a tissue homogenizer at low temperature for 6 min. The resulting suspension was centrifuged at 12,000 × *g* at 4 °C for 5 min. The supernatants of equal volume were subsequently analyzed using ELISA kits to measure IL-1β, IL-18 and IL-15, following the standard protocols.

### Flow cytometry

For flow cytometry analysis, single cell suspensions from tumor, spleen and lymph nodes were stained with different combinations of antibodies for 30 min under dark, including anti-mouse CD3-FITC (#100204, Biolegend, RRID: AB_312661, 1:100 dilution), anti-mouse CD8-APC (#140410, Biolegend, RRID: AB_10641696, 1:100 dilution), anti-mouse CD4-PE (#100408, Biolegend, RRID: AB_312693, 1:100 dilution), anti-mouse CD11c-APC (#117310, Biolegend, RRID: AB_313779, 1:200 dilution), anti-mouse CD80-PE mAb (#104708, Biolegend, RRID: AB_313129, 1:100 dilution), anti-mouse CD86-PE-Cyanine7 (#105014, Biolegend, RRID: AB_439783, 1:100 dilution), anti-mouse CD44-PE-Cyanine7 mAb (#103030, Biolegend, RRID: AB_830787, 1:100 dilution), anti-mouse CD62L-PerCP/Cy5.5 mAb (#104430, Biolegend, RRID: AB_2187124, 1:100 dilution), MICA/B (#320906, Biolegend, RRID: AB_493193, 1:200 dilution), and anti-mouse-MHC-I mAB (#2102629, eBioscience, RRID: AB_465358, 1:100 dilution). After staining, the samples were washed two times by PBS and finally resuspended by 300 µl PBS for detection. The anti-mouse Slc16a13 (p. 234-241: YVHLVANL, 1:50 dilution) and anti-mouse Ptpn2 (p. 376-384: RWLYWQPTL, 1:50 dilution)-specific tetramer-PE were purchased from BioReagent Unit of Cancer Research Center of Xiamen University (Xiamen, China). Tumor single cell suspensions were stained with 2 μl tetramer for 20 min at room temperature, then anti-mouse CD8 antibody was added and incubated for another 20 min at room temperature. All samples were analyzed on a flow cytometer (BD FACSVerseTM, USA), and data were analyzed by FlowJo v.10.

### Immunohistochemistry

For immunohistochemistry, standardized procedures were followed. Tumor tissues from mice were fixed, paraffin-embedded, sectioned, dewaxed, and blocked. They were then incubated overnight with primary antibodies, including anti-mouse CD4 (ab217344, Abcam, 1:2000 dilution), anti-mouse CD8 (ab183685, Abcam, 1:1000 dilution), and anti-mouse NK1.1 (A25457, Abclonal, 1:1000 dilution). Afterwards, the tissues were incubated with secondary antibodies, stained with DAB, and mounted. QuPath software was used to analyze the positive cell rate in the IHC images.

### Immunofluorescence and multi-color immunofluorescence

For Immunofluorescence analysis, tumor tissue slides were stained according to the IHC procedures, using anti-Calreticulin (ab315210, Abcam, 1:250 dilution) as the primary antibody, the secondary antibody was labeled by Alexa Fluor 690, and the nucleuses were counterstained with DAPI. For Multi-color Immunofluorescence analysis, the Multi-color immunofluorescence is performed according to 6-Plex Detection Kit (AFIHC027, AiFang Biological). Briefly, slides were stained with anti-mouse CD4 (ab183685, Abcam, 1:100 dilution), anti-mouse CD8 (ab217344, Abcam, 1:100 dilution), anti-mouse-NK1.1 (A25457, Abclonal, 1:50 dilution), anti-mouse NKG2D (GTX50988, GeneTex, 1:50 dilution) and anti-mouse TNF-α (11948, CST, 1:100 dilution). The slides were scanned and analyzed by PhenoCycle-Fusion 2.0 (Akoya Bioscience). The phenoptrReports R Package was downloaded from the official Akoya website to analyze the obtained data.

### Cell viability assay

In order to observe the effects of GSDMD-KO on tumor cell proliferation, Hepa1-6 cells (including WT and GSDMD-KO) were cultured in 96-well plates at a density of 5 × 10^3^ cells/well with 150 µl complete medium, at 37 °C in an atmosphere containing 5% CO_2_. Following the different time incubation at 37 °C, cell viability was determined using the cell counting kit-8 (CCK-8, Yeasen, China) according to the manufacturer’s protocol. A microplate reader (Spectramax m5e, Molecular Devices, USA) was used to measure the absorbance of the solution in each well at 450 nm. All experiments were performed in triplicate.

### Flow cytometric sorting and cytotoxicity assay

Twelve days after combined treatment, fresh tumor tissues of mice in the combined treatment group were obtained to prepare single-cell suspensions, which were stained with CD49a (#142605, Biolegend, RRID: AB_2562252, 1:100 dilution) and CD103 (#110903, Biolegend, RRID: AB_2927994, 1:100 dilution) and sorted using a Fusion cell sorter (BS FACSAria^TM^, USA). The sorted cells were co-incubated with Hepa1-6-hMICB cells (or GSDMD-KO), and the cytotoxicity was detected by LDH assay kit after 48 h.

### Ethics approval

All animal procedures in this study were conducted according to the “National animal management regulations of China” and approved by the Animal Ethics Committee of Mengchao Hepatobiliary Hospital of Fujian Medical University (MCHH-AEC-2023-12).

### Sex as a biological variable

Our study exclusively examined male mice. It is unknown whether the findings are relevant for female mice.

### Statistical analysis

The experiments were performed as non‑blinded tests. All cell lines used in vitro were treated identically without any preselection. For in vivo studies, animals were randomly assigned to groups by stratifying tumor volume or body weight, and sample sizes were determined based on prior experience with significance. The statistical analyses were performed using GraphPad Prism (version 9.0.0) software. Survival curves were estimated using the Kaplan–Meier method and compared with the log-rank test. Unless otherwise stated, the results were presented as mean ± SEM from at least three biologically independent samples. Differences between the two groups were analyzed by a two-tailed unpaired *t* test. Differences among multiple groups were analyzed using a two-tailed one-way analysis of variance (ANOVA). Statistical significance was set at *P* < 0.05.

## Supplementary information

Appendix

Peer Review File

Source data Fig. 1

Source data Fig. 2

Source data Fig. 3

Source data Fig. 4

Source data Fig. 6

Source data Fig. 7

Expanded View Figures

## Data Availability

The single-cell transcriptome sequencing data from this publication have been deposited to the Genome Sequencing Achieve (GSA) database (GSA number: CRA020188, UTR: https://ngdc.cncb.ac.cn/gsa/browse/CRA020188). The source data of this paper are collected in the following database record: biostudies:S-SCDT-10_1038-S44321-026-00424-6.
