# Supplementary material for: Neoantigens and shared MICB α3 antigen dual-targeted vaccine generates potent antitumor immunity
Source: EMBO Mol Med. 2026 Apr 17;18(6):2098–123. doi: 10.1038/s44321-026-00424-6 (PMC13269783; doi:10.1038/s44321-026-00424-6)
Supplement: Supplementary file 1 — Appendix [file 44321_2026_424_MOESM1_ESM.pdf]

Appendix Data

Table of contents:

APPENDIX	PAGE
Appendix Figure S1	1
Appendix Figure S2	2
Appendix Figure S3	3
Appendix Figure S4	4
Appendix Figure S5	5
Appendix Figure S6	7
Appendix Figure S7	8
Appendix Figure S8	10
Appendix Figure S9	12
Appendix Figure S10	13
Appendix Figure S11	14
Appendix Figure S12	15
Appendix Figure S13	16
Appendix Table S1	17
Appendix Table S2	18
Appendix Table S3	21
Appendix Table S4	22

Appendix figures

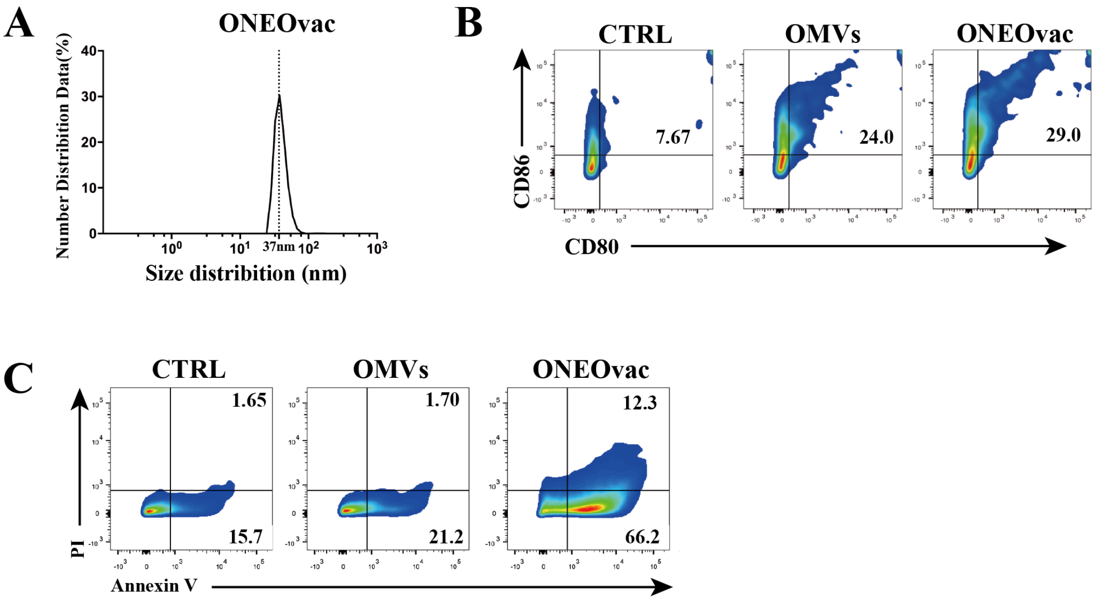

Appendix Figure S1. (A) Dynamic light scattering (DLS) analysis of ONEOvac. **(B)** Flow cytometry analysis of $CD80^+$ and $CD86^+$ cells in $CD11c^+$ cells following treatment with PBS, OMVs and ONEOvac. **(C)** Flow cytometry showing the percentage of apoptotic cells.

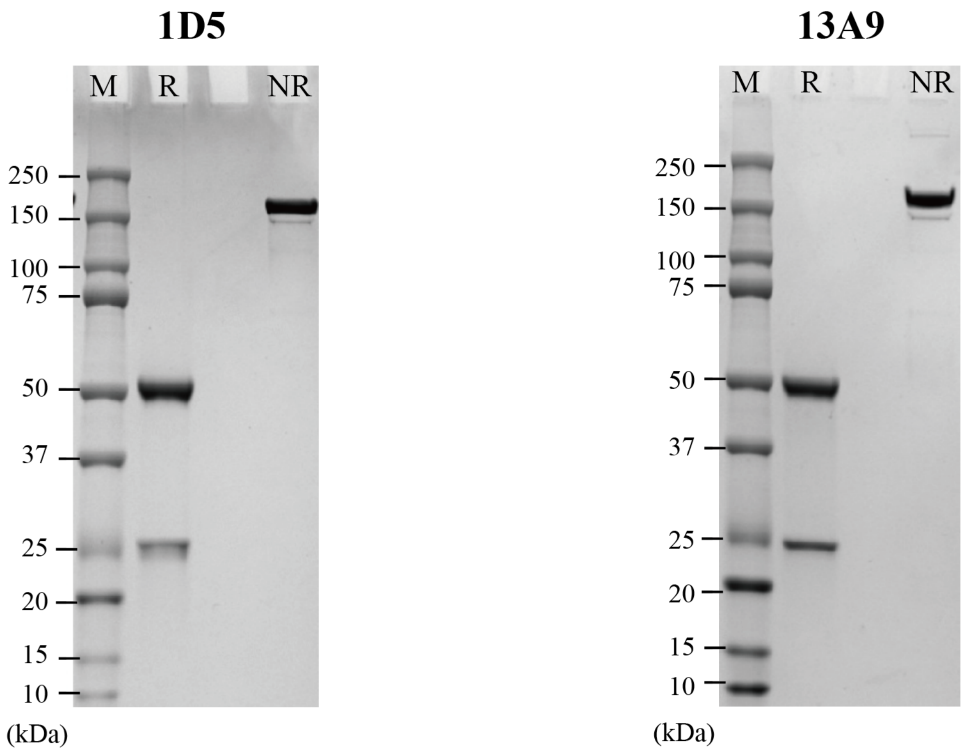

Lane M: Protein Marker; Lane R: Reducing condition; Lane NR: Non-reducing condition.

Appendix Figure S2. The purified anti-MICA α3 antibodies (1D5 and 13A9) were checked in a native PAGE gel. A total of 500 ng of each sample was loaded. 1D5: light chain 234 aa (~25 kDa), heavy chain 466 aa (~50 kDa); 13A9: light chain 233 aa (~25 kDa), heavy chain 466 aa (~50 kDa).

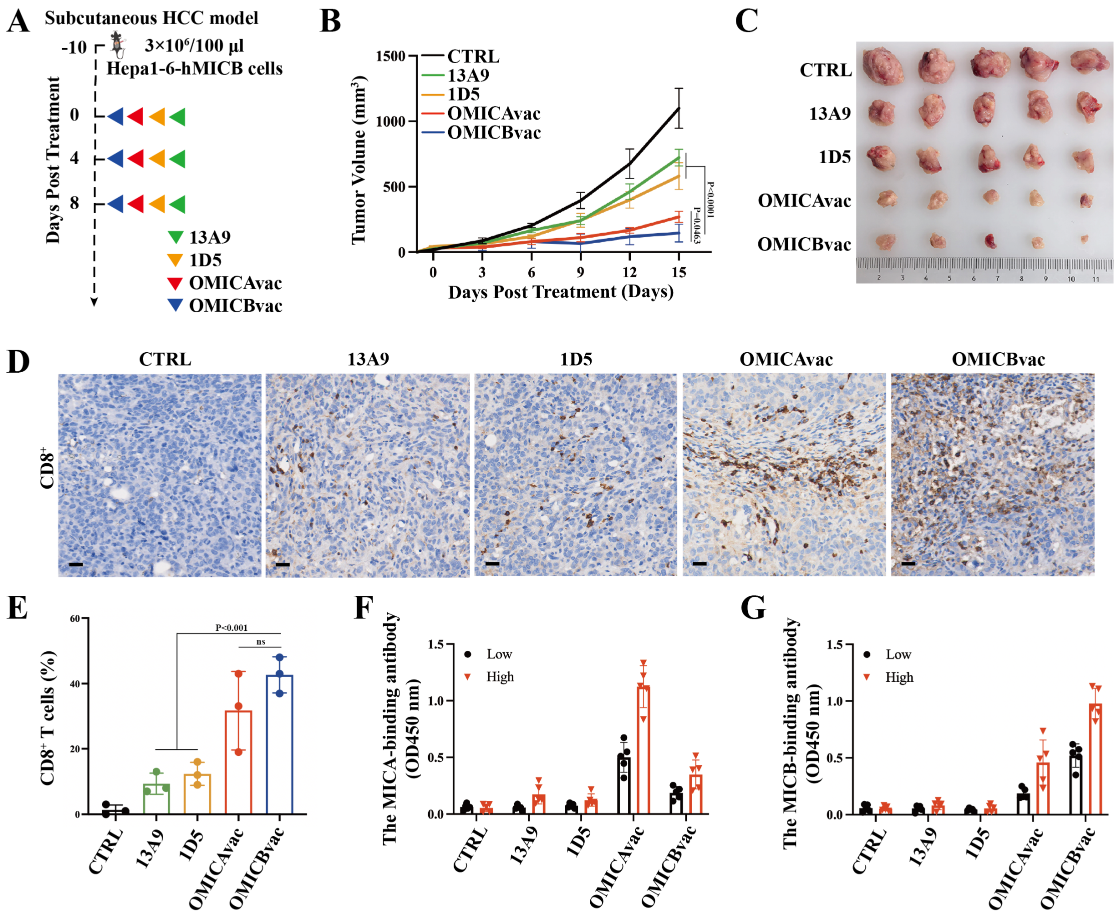

Appendix Figure S3. (A) Schematic representation of the Hepa1-6-hMICB subcutaneous HCC model and the treatment timeline. The mice were randomly divided into five groups and subsequently vaccinated with PBS, 13A9 (10 µg), 1D5 (10 µg), OMICAvac (10 µg), or OMICBvac (10 µg) on days 0, 4, and 8, respectively. **(B and C)** The mouse tumor volume was monitored for every 3 days (n=5). **(D and E)** IHC images show $CD8^+$ T cells infiltrating into tumor tissues (n=3) in each treatment group as indicated. Scale bars, 20 µm. **(F and G)** ELISA quantification of the induction of anti-MICB α3 IgG levels in serum by two different substrates: MICA (F) and MICB (G).

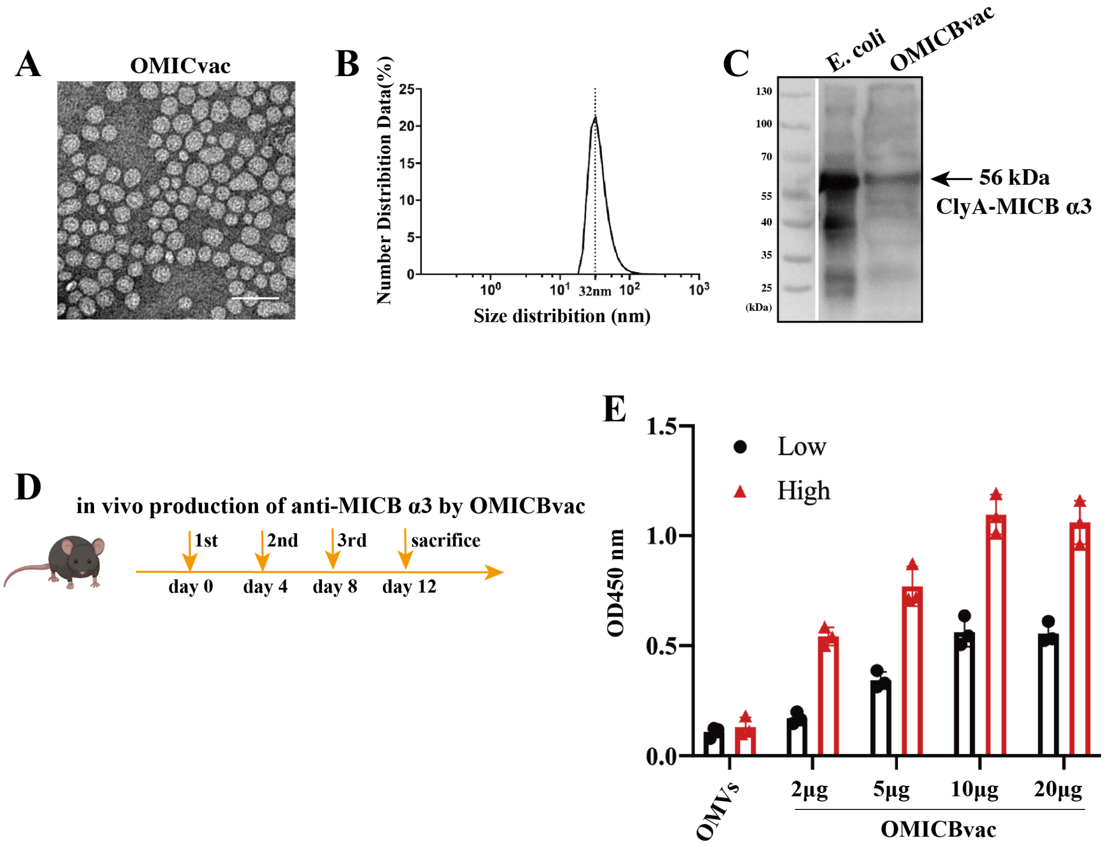

Appendix Figure S4. (A) TEM analysis of OMICBvac (ClyA-MICB α3 domain). Scale bar, 50 nm. **(B)** Dynamic light scattering (DLS) analysis of OMICBvac. **(C)** Western blot analysis of ClyA-MICB α3 domain (56 kDa) in OMVs produced by *E. coli* Rosetta (DE3). **(D)** Schematic representation of in vivo induction of anti-MICB α3 by OMICBvac. **(E)** ELISA quantification of mouse anti-MICB α3 IgG levels in serum after stimulation with different amounts of OMICBvac (n=3). Low: serum was diluted 10000-fold. High: serum was diluted 100-fold. The absorbance was read at 450 nm.

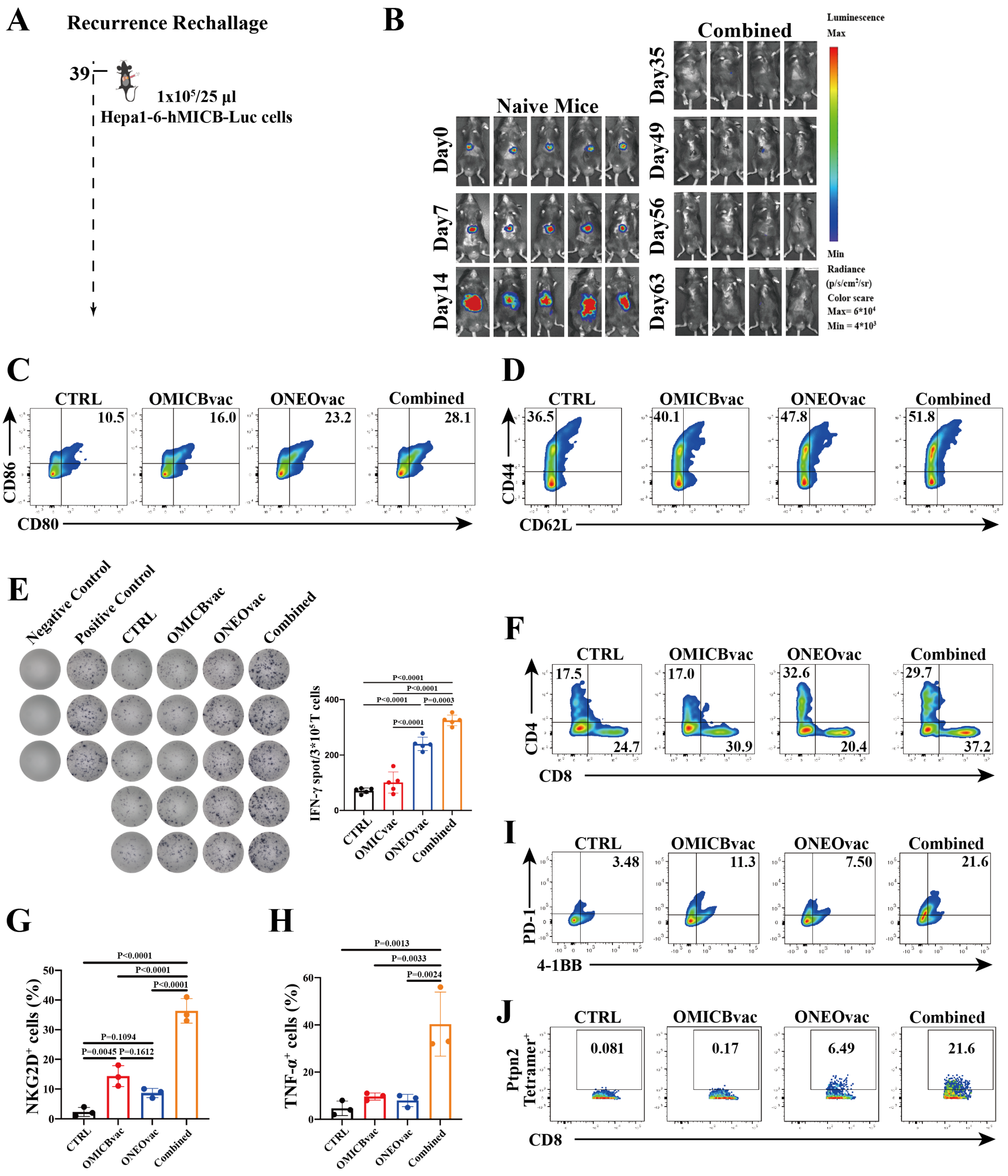

Appendix Figure S5. (A) Schematic view of the Hepa1-6-hMICB orthotopic HCC recurrence rechallenge model. **(B)** Tumor burden monitoring of recurrence rechallenge in mice by bioluminescence imaging. The mouse shown in Row 1 (Day 35), Combined group, Columns 1, 2, 3, and 4 corresponds to the mouse shown in Figure 3D, Row 6 (Day 35), Combined group, Columns 3, 4, 5, and 7. These images represent the same tumor-bearing mouse at the same time point, imaged and presented across two different figures. **(C and D)** Flow cytometry analysis of DC maturation in axillary lymph nodes (C), effector memory T cells ($CD8^+$ Tem) in spleen (D). **(E)** ELISPOT assay for

neoantigen-specific immune response induced by different treatment strategy in the Hepa1-6-hMICB orthotopic HCC mouse model (n=5). **(F)** Flow cytometry analysis of $CD4^+$ TIL and $CD8^+$ TIL. **(G** and **H)** The cell density of $NKG2D^+$ cells (G), TNF-α^+ cells (H) of entire tumor section was shown in statistical scatter plots (n = 3 slides from different mouse per group). **(I** and **J)** Flow cytometry analysis of 4-1BB$^+$CD8$^+$ TIL (I), and Ptpn2 tetramer$^+$ specific $CD8^+$ T cells in $CD8^+$ TILs (J).

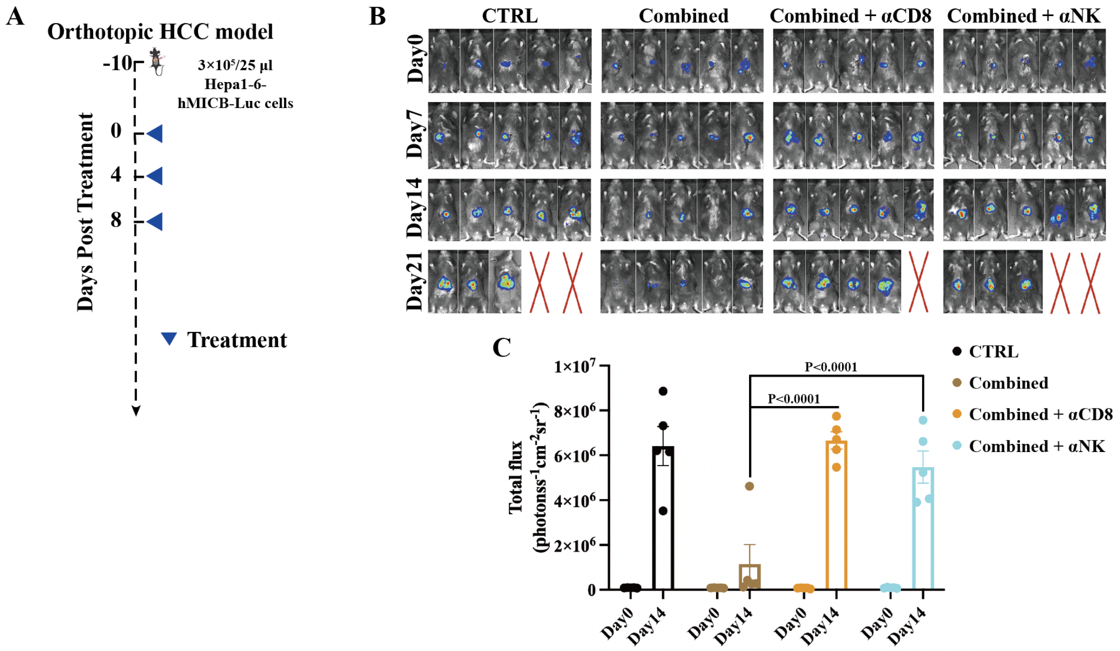

Appendix Figure S6. (A) Schematic illustration of the Hepa1-6-hMICB orthotopic HCC model and the treatment timeline. CTRL: PBS; Combined: 10 µg OMICBvac + 10 µg ONEOvac; αCD8: 20 µg anti-Mouse CD8a; αNK: 20 µg anti-Mouse NK1.1. **(B** and **C)** Tumor burden monitoring in mice treated with CTRL, Combined, Combined + αCD8, and Combined + αNK, as assessed by bioluminescence imaging (n=5).

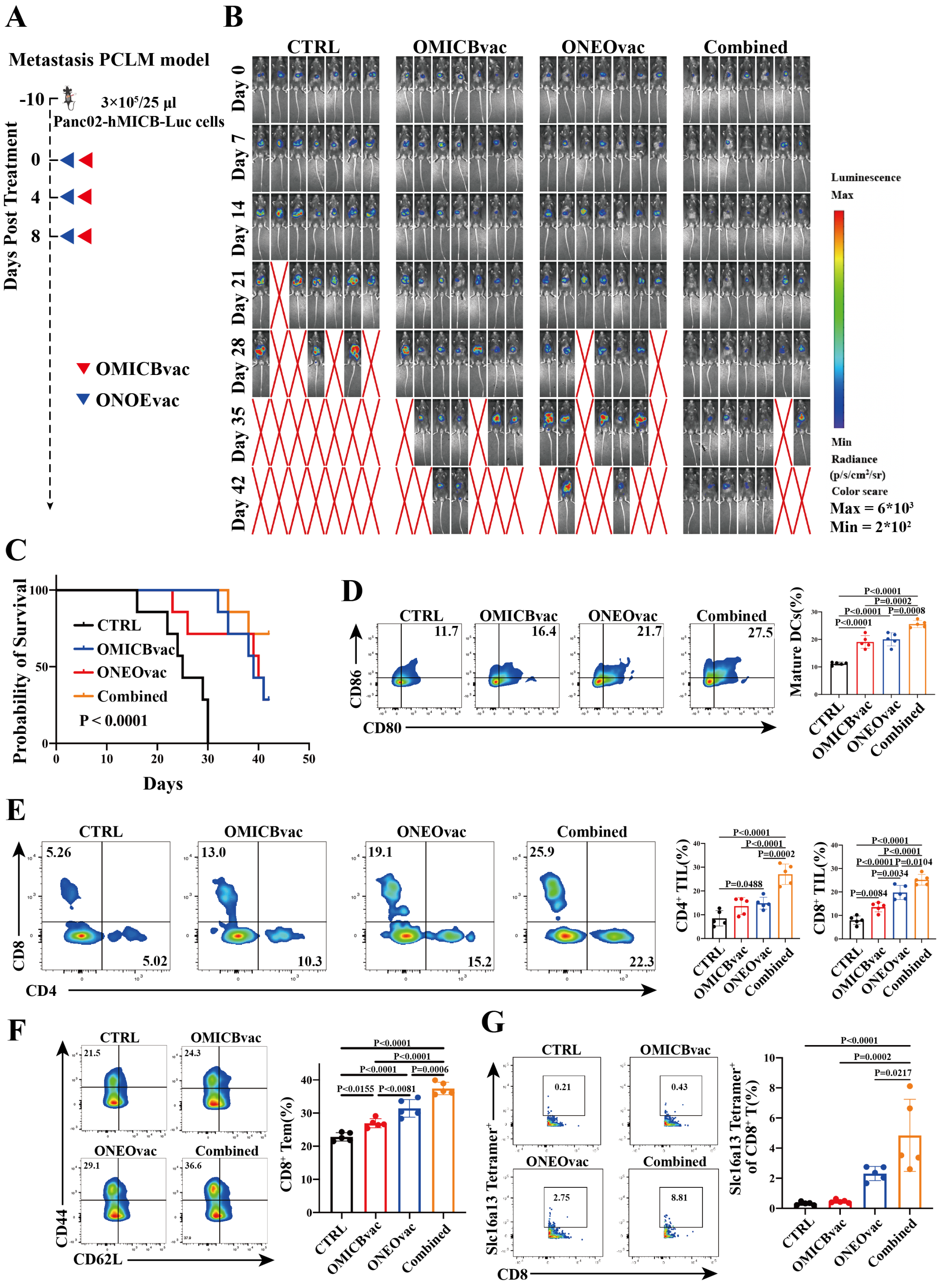

Appendix Figure S7. The antitumor effects by the combined treatment of ONEOvac plus OMICBvac in metastasis PCLM model. **(A)** Schematic view of the metastasis PCLM model and the treatment procedure timeline. **(B)** Tumor burden monitoring of PBS, OMICBvac, ONEOvac and Combination (ONEOvac plus OMICBvac) treated mice by bioluminescence imaging (n=7). **(C)** Kaplan-Meier survival curves of each

group (n=7). **(D-G)** Flow cytometry analysis of the percentage of DC maturation in axillary lymph nodes, effector memory T cells ($CD8^+$ Tem) in spleen (D), $CD4^+$ TIL (E), $CD8^+$ TIL (F) and Slc16a13 $tetramer^+$ specific $CD8^+$ in $CD8^+$ TILs (G) (n = 5).

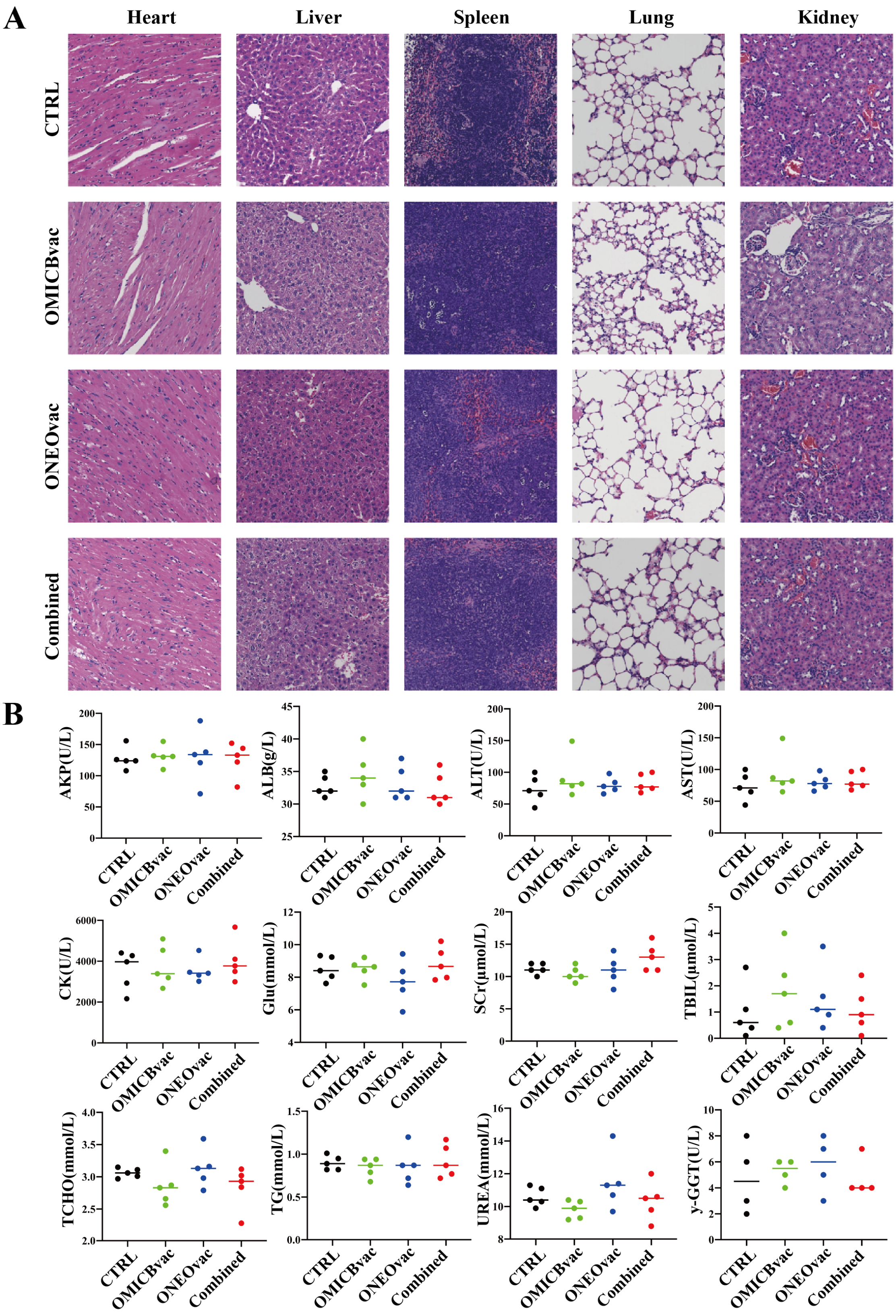

Appendix Figure S8. Safety evaluation of the combined treatment strategy. **(A)** H&E staining of major organs (heart, liver, spleen, lung and kidney) in the different treatment groups of mice in Hepa1-6-hMICB orthotopic HCC models. **(B)** Changes in serum

biochemical indices including alanine aminotransferase (ALT), aspartate aminotransferase (AST), albumin (ALB), total bilirubin (TBil), total protein (TP), Globulin (GLB), urea (Urea) and creatinine (Creatine). The samples were harvested and analyzed on day 12 from Figure 3C.

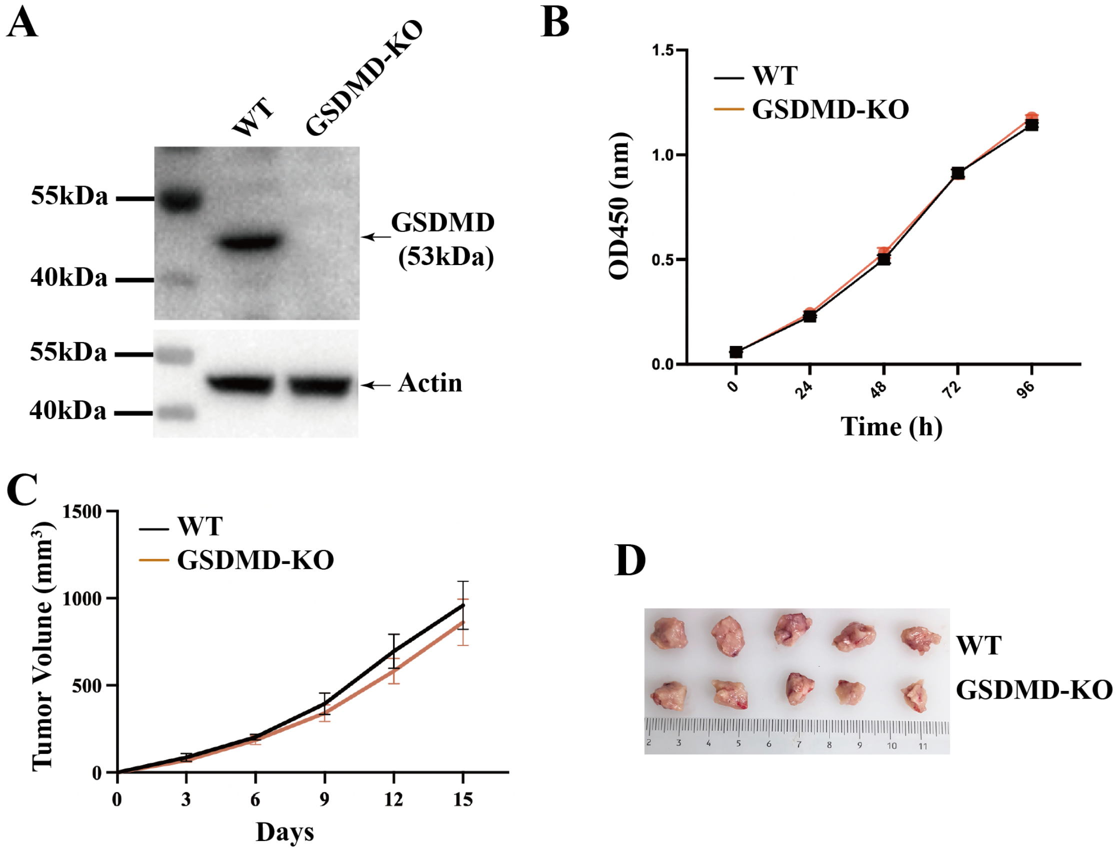

Appendix Figure S9. (**A)** Western blot shows the knockout efficiency of *GSDMD* in Hepa1-6 cells. **(B)** CCK-8 analysis of cell activity under different time points. **(C** and **D)** Comparison of tumor growth efficiency between WT and GSDMD-KO Hepa1-6 cells in mice.

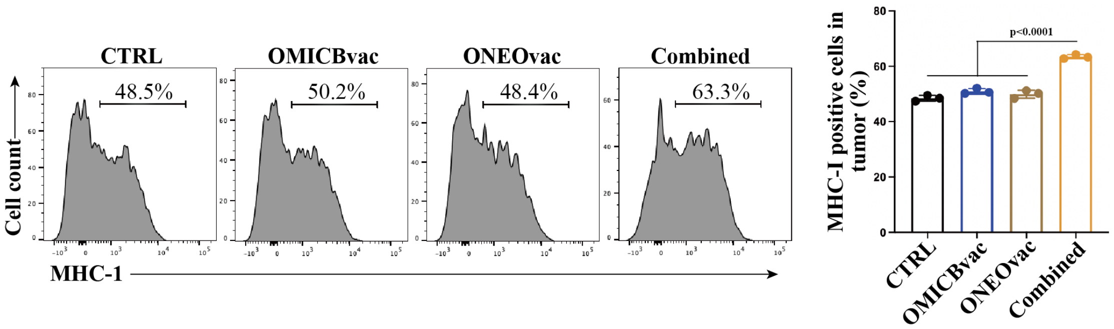

Appendix Figure S10. Flow cytometry analysis of the expression level of MHC-I on tumor cells.

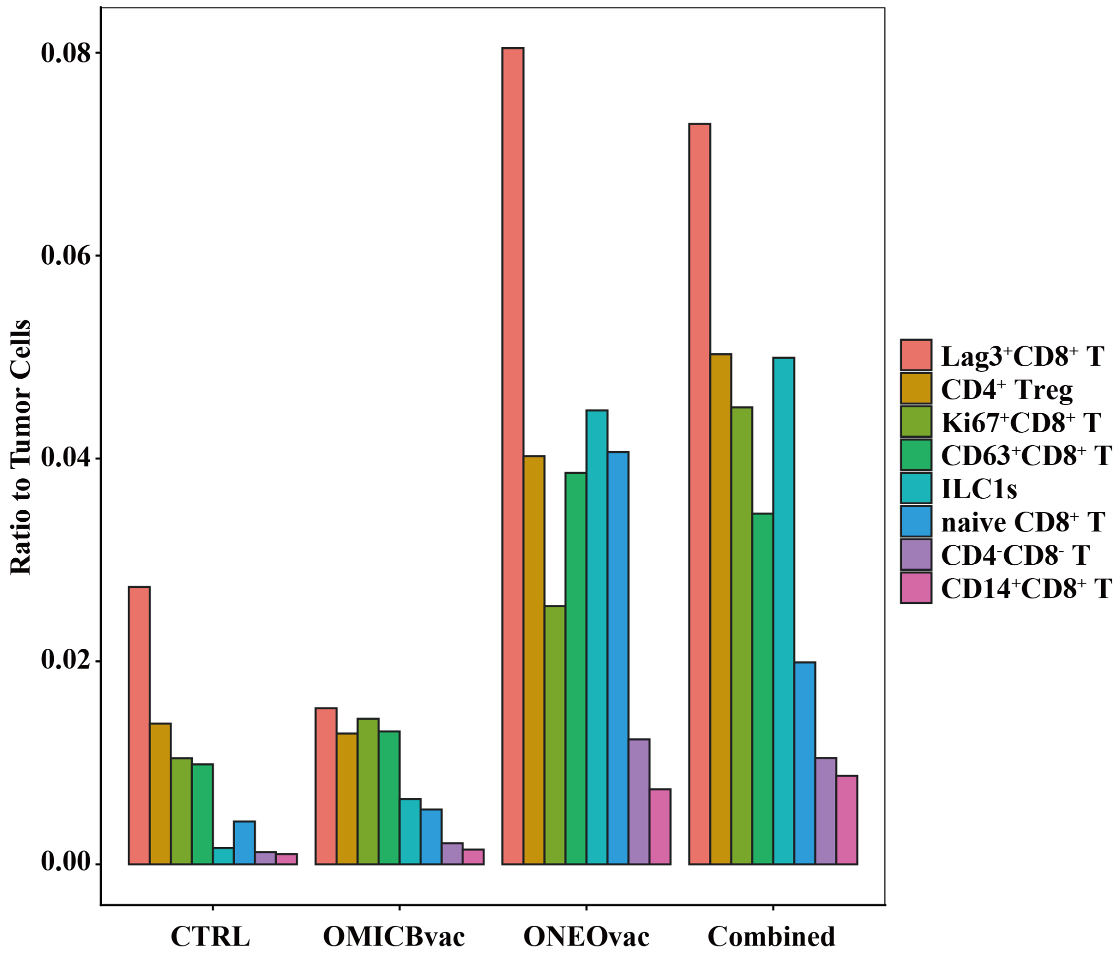

Appendix Figure S11. Proportions of various immune cell populations relative to tumor cells across different treatment conditions from Figure 6B. Cell subsets analyzed include Lag3$^+$CD8$^+$ T cells, CD4$^+$ Tregs, Ki67$^+$CD8$^+$ T cells, CD63$^+$CD8$^+$ T cells, ILC1s, naive CD8$^+$ T cells, CD4$^-$CD8$^-$ T cells, and CD14$^+$CD8$^+$ T cells.

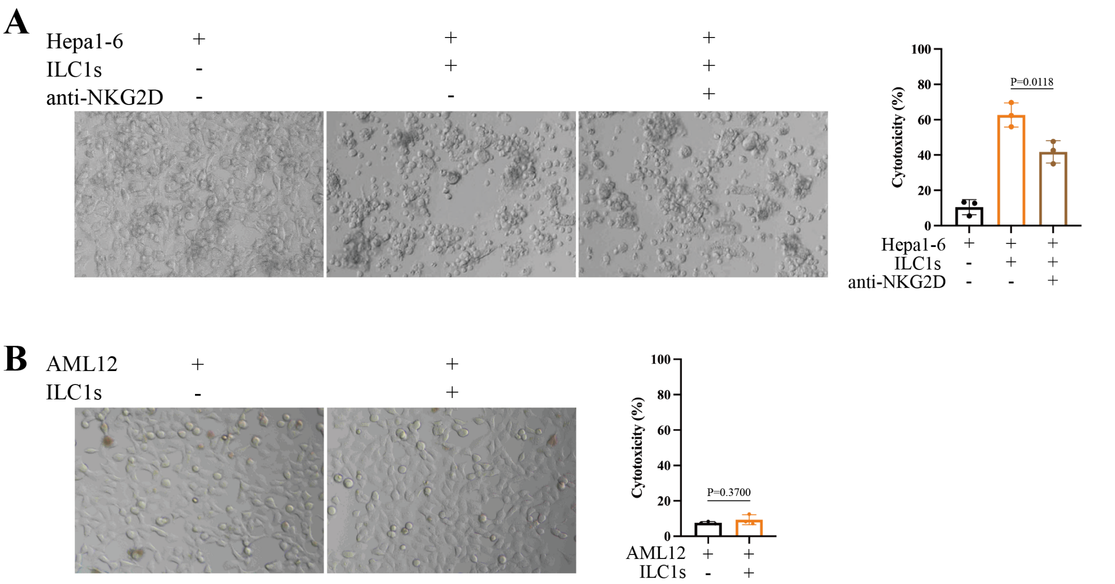

Appendix Figure S12. Microscopic images of ILC1s co-cultured with Hepa1-6 (A) and AML12 (B) cells after 48 hours. The cell death percentage induced by ILC1s was analyzed by LDH detection (n=3).

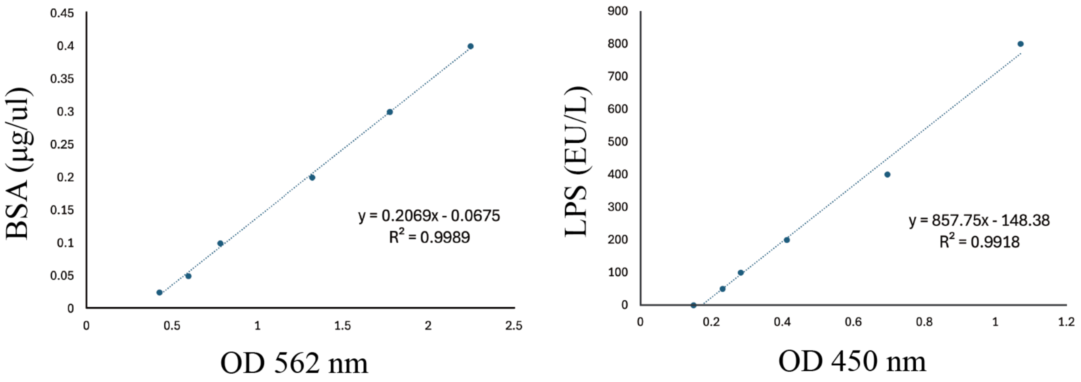

Sample/100mL *E.coli*	Protein amount (ug)	LPS (EU/ug)
CTRL (empty plasmid)	388.5 ± 7.647	86.22 ± 1.285
ONEOvac	523.4 ± 4.930	94.79 ± 15.87
OMICBvac	518.4 ± 5.717	92.22 ± 3.021

Appendix Figure S13. Figure S13: Quality control of OMV-vaccine production and LPS content by ELISA analysis. Data are presented as mean ± SEM (n=3).

Appendix Table S1. Information of neoantigen mutation peptides derived from Hepa 1-6 and Panc02 cell line

Cell line	ID	Gene	Mutation	Peptide (MT[b])
Hepa 1-6	chr7_126764306	Mapk3	S284F	MKARNYLQFLPSKTKVA
Hepa 1-6	chr1_181821532	Lbr	A341P	LYTHFLQLPLAATGFSV
Hepa 1-6	chr17_24511368	Traf7	C403W	WDTCTTYKWQKTLEGHD
Hepa 1-6	chr6_3375005	Samd9l	K752M	HVLWDLKQMFRCAVLKN
Hepa 1-6	chr18_67672646	Ptpn2	I383T	KRWLYWQPTLTKMGFVS
Hepa 1-6	chr17_25662545	Lmf1	F523V	RGEHYRYKVSLPGGQHA
Hepa 1-6	chr12_3591957	Dtnb	K40T	LSTYRTACTLRFVQKRC
Panc02	chr5_143478447	Daglb	G238C	LVPSDIAACFTLLHQQQ
Panc02	chr11_70218956	Slc16a13	H240N	IPYVHLVANLQDLGWDP
Panc02	Chr9_49028206	Usp28	W631L	DISVTESSLEELERDSY
Panc02	chr15_100169290	Dip2b	D558H	GETVVNVLHFKKDAGLW
Panc02	Chr2_25051725	Pnpla7	W1153C	GWWLLWKRCNPLATKVK
Panc02	chr15_98125587	Pfkm	A389S	NWEVYKLLSHVRPPVSK
Panc02	Chr2_121189374	Tubgcp4	A395G	VTEHDVNVGFQQSAHKV
Panc02	Chr9_20438211	Zfp26	F352L	YECKKCEKLFTHPVYLN

Appendix Table S2. The sequences of plasmids used in the study

Plasmid	DNA Sequence
1. pET28a-ClyA-Hepa1-6(Neoantigens)-Fc	The sequence of ClyA-HA-Hepa1-6 (Neo)-Fc as shown in the bottom was inserted into the pET28a back bone:<br>atgactgaaatcgttgcagataaaacggtagaagtagttaaaaacgcaatcgaaaccgcag<br>atggagcattagatctttataataaatatctcgatcaggtcatcccctggcagacctttgatgaa<br>accataaaagagttaagtcgctttaaacaggagtattcacaggcagcctccgttttagtcggc<br>gatattaaaaccttacttatggatagccaggataagtattttgaagcaacccaaacagtgtatg<br>aatggtgtggtgttgcgacgcaattgctcgcagcgtatattttgctatttgatgagtacaatga<br>gaagaaagcatccgcccagaaagacattctcattaaggtactggatgacggcatcacgaa<br>gctgaatgaagcgcaaaaatccctgctggtaagctcacaaagtttcaacaacgcttccggg<br>aaactgctggcgttagatagccagttaaccaatgatttttcagaaaaaagcagctatttccagt<br>cacaggtagataaaatcaggaaggaagcatatgccggtgccgcagccggtgtcgtcgcc<br>ggtccatttggattaatcatttcctattctattgctgcgggcgtagttgaaggaaaactgattcc<br>agaattgaagaacaagttaaaatctgtgcagaatttctttaccaccctgtctaacacggttaaa<br>caagcgaataaagatatcgatgccgccaaattgaaattaaccaccgaaatagccgccatcg<br>gtgagataaaaacggaaactgaaacaaccagattctacgttgattatgatgatttaatgctttct<br>ttgctaaaagaagcggccaaaaaaatgattaacacctgtaatgagtatcagaaaagacacg<br>gtaaaaagacactctttgaggtacctgaagtcggaggctacccatacgatgttccagattac<br>gcttacccatacgatgttccagattacgcttacccatacgatgttccagattacgctggaggc<br>ggatccatgaaggcccggaactacctgcagttcctgcccagcaagaccaaggtggcccg<br>gggccggaagcggcggagccggggcgagcactaccggtacaaggtgagcctgcccgg<br>cggccagcacgcccggggccggaagcggcggagccacgtgctgtgggacctgaagca<br>gatgttccggtgcgccgtgctgaagaaccggggccggaagcggcggagctgggacacc<br>tgcaccacctacaagtggcagaagaccctggagggccacgaccggggccggaagcgg<br>cggagcctgagcacctaccggaccgcctgcaccctgcggttcgtgcagaagcggtgccg<br>gggccggaagcggcggagcctgtacacccacttcctgcagctgcccctggccgccaccg<br>gcttcagcgtgcggggccggaagcggcggagcaagcggtggctgtactggcagcccac<br>cctgaccaagatgggcttcgtgagcgaattcggaggtggcggatctatggaagtgagcag<br>cgtgtttatttttccgccgaaaccgaaagatgtgctgaccattaccctgaccccgaaagtgac<br>ctgcgtggtggtggatattagcaaagatgatccggaagtgcagtttagctggtttgtggatga<br>tgtggaagtgcataccgcgcagacccagccgcgcgaagaacagtttaacagcacctttcg<br>cagcgtgagcgaactgccgattatgcatcaggattggctgaacggcaaagaatttaaatgc<br>cgcgtgaacagcgcggcgtttccggcgccgattgaaaaaaccattagcaaaaccaaaggc<br>cgcccgaaagcgccgcaggtgtataccattccgccgccgaaagaacagatggcgaaaga<br>taaagtgagcctgacctgcatgattaccgatttttttccggaagatattaccgtggaatggcag<br>tggaacggccagccggcggaaaactataaaaacacccagccgattatggataccgatggc<br>agctattttgtgtatagcaaactgaacgtgcagaaaagcaactgggaagcgggcaacacct<br>ttacctgcagcgtgctgcatgaaggcctgcataaccatcataccgaaaaaagcctgagcca<br>tagcccgggcaaagaataatag
2. pET28a-ClyA-Panc02(Neoantigens)-Fc	The sequence of ClyA-HA-Panc02 (Neo)-Fc as shown in the bottom was inserted into the pET28a back bone.<br>atgactgaaatcgttgcagataaaacggtagaagtagttaaaaacgcaatcgaaaccgcag<br>atggagcattagatctttataataaatatctcgatcaggtcatcccctggcagacctttgatgaa<br>accataaaagagttaagtcgctttaaacaggagtattcacaggcagcctccgttttagtcggc<br>gatattaaaaccttacttatggatagccaggataagtattttgaagcaacccaaacagtgtatg<br>aatggtgtggtgttgcgacgcaattgctcgcagcgtatattttgctatttgatgagtacaatga<br>gaagaaagcatccgcccagaaagacattctcattaaggtactggatgacggcatcacgaa<br>gctgaatgaagcgcaaaaatccctgctggtaagctcacaaagtttcaacaacgcttccggg<br>aaactgctggcgttagatagccagttaaccaatgatttttcagaaaaaagcagctatttccagt<br>cacaggtagataaaatcaggaaggaagcatatgccggtgccgcagccggtgtcgtcgcc<br>ggtccatttggattaatcatttcctattctattgctgcgggcgtagttgaaggaaaactgattcc<br>agaattgaagaacaagttaaaatctgtgcagaatttctttaccaccctgtctaacacggttaaa<br>caagcgaataaagatatcgatgccgccaaattgaaattaaccaccgaaatagccgccatcg<br>gtgagataaaaacggaaactgaaacaaccagattctacgttgattatgatgatttaatgctttct

	ttgctaaaagaagcggccaaaaaaatgattaacacctgtaatgagtatcagaaaagacacg<br>gtaaaaagacactctttgaggtacctgaagtcggaggctacccatacgatgttccagattac<br>gcttacccatacgatgttccagattacgcttacccatacgatgttccagattacgctggaggc<br>ggatccctggttccgagcgatattgcggcctgctttaccctgctgcatcagcagcagcggg<br>gccggaagcggcggagcattccgtacgtgcatctggtggcgaacctgcaggatctgggct<br>gggacccgcggggccggaagcggcggagcgatattagcgtgacggaaagctcactgga<br>agaactggaacgcgatagctatcggggccggaagcggcggagcggcgaaaccgtggtg<br>aacgttctgcacttcaaaaaagatgcgggtctgtggcggggccggaagcggcggagcgg<br>ctggtggctgctgtggaaacgctgcaatccgctggccaccaaagtgaaacggggccgga<br>agcggcggagcaactgggaagtttacaaactgctgagccatgtgcgcccgccggtgagc<br>aaacggggccggaagcggcggagcgtgaccgaacatgatgtgaatgtgggctttcagca<br>gagcgcgcacaaagtgcggggccggaagcggcggagctatgaatgtaaaaaatgcgaa<br>aaactgtttacccacccggtgtacctgaatgaattcggaggtggcggatctatggaagtgag<br>cagcgtgtttatttttccgccgaaaccgaaagatgtgctgaccattaccctgaccccgaaagt<br>gacctgcgtggtggtggatattagcaaagatgatccggaagtgcagtttagctggtttgtgg<br>atgatgtggaagtgcataccgcgcagacccagccgcgcgaagaacagtttaacagcacct<br>ttcgcagcgtgagcgaactgccgattatgcatcaggattggctgaacggcaaagaatttaaa<br>tgccgcgtgaacagcgcggcgtttccggcgccgattgaaaaaaccattagcaaaaccaaa<br>ggccgcccgaaagcgccgcaggtgtataccattccgccgccgaaagaacagatggcgaa<br>agataaagtgagcctgacctgcatgattaccgatttttttccggaagatattaccgtggaatgg<br>cagtggaacggccagccggcggaaaactataaaaacacccagccgattatggataccgat<br>ggcagctattttgtgtatagcaaactgaacgtgcagaaaagcaactgggaagcgggcaac<br>acctttacctgcagcgtgctgcatgaaggcctgcataaccatcataccgaaaaaagcctga<br>gccatagcccgggcaaagaataatag
3. pET28a-ClyA-MICA (a3)	the sequence of ClyA-HA-MICA (a3) as shown in the bottom was inserted into the pET28a back bone.<br>atgactgaaatcgttgcagataaaacggtagaagtagttaaaaacgcaatcgaaaccgcag<br>atggagcattagatctttataataaatatctcgatcaggtcatcccctggcagacctttgatgaa<br>accataaaagagttaagtcgctttaaacaggagtattcacaggcagcctccgttttagtcggc<br>gatattaaaaccttacttatggatagccaggataagtattttgaagcaacccaaacagtgtatg<br>aatggtgtggtgttgcgacgcaattgctcgcagcgtatattttgctatttgatgagtacaatga<br>gaagaaagcatccgcccagaaagacattctcattaaggtactggatgacggcatcacgaa<br>gctgaatgaagcgcaaaaatccctgctggtaagctcacaaagtttcaacaacgcttccggg<br>aaactgctggcgttagatagccagttaaccaatgatttttcagaaaaaagcagctatttccagt<br>cacaggtagataaaatcaggaaggaagcatatgccggtgccgcagccggtgtcgtcgcc<br>ggtccatttggattaatcatttcctattctattgctgcgggcgtagttgaaggaaaactgattcc<br>agaattgaagaacaagttaaaatctgtgcagaatttctttaccaccctgtctaacacggttaaa<br>caagcgaataaagatatcgatgccgccaaattgaaattaaccaccgaaatagccgccatcg<br>gtgagataaaaacggaaactgaaacaaccagattctacgttgattatgatgatttaatgctttct<br>ttgctaaaagaagcggccaaaaaaatgattaacacctgtaatgagtatcagaaaagacacg<br>gtaaaaagacactctttgaggtacctgaagtcggaggctacccatacgatgttccagattac<br>gcttacccatacgatgttccagattacgcttacccatacgatgttccagattacgctggaggc<br>ggatccgtccctccaatggtgaacgtgacaaggagcgaagcctccgagggcaacattact<br>gtgacatgtagggcttcttccttctatcccagaaacatcatcctgacctggcgccaggatggc<br>gtgtccctgagccacgacacacagcagtggggagacgttctgcccgacggcaacggcac<br>ttaccagacctgggtggccacccggatctgccggggcgaggagcagagatttacatgttac<br>atggagcacagcgggaatcactccacccatcccgtgcctagcggcaaggtgctggtgctg<br>cagtcccattaatag
4. pET28a-ClyB-MICB (a3)	the sequence of ClyA-HA-MICB (a3) as shown in the bottom was inserted into the pET28a back bone.<br>atgactgaaatcgttgcagataaaacggtagaagtagttaaaaacgcaatcgaaaccgcag<br>atggagcattagatctttataataaatatctcgatcaggtcatcccctggcagacctttgatgaa<br>accataaaagagttaagtcgctttaaacaggagtattcacaggcagcctccgttttagtcggc<br>gatattaaaaccttacttatggatagccaggataagtattttgaagcaacccaaacagtgtatg<br>aatggtgtggtgttgcgacgcaattgctcgcagcgtatattttgctatttgatgagtacaatga<br>gaagaaagcatccgcccagaaagacattctcattaaggtactggatgacggcatcacgaa<br>gctgaatgaagcgcaaaaatccctgctggtaagctcacaaagtttcaacaacgcttccggg<br>aaactgctggcgttagatagccagttaaccaatgatttttcagaaaaaagcagctatttccagt<br>cacaggtagataaaatcaggaaggaagcatatgccggtgccgcagccggtgtcgtcgcc

	ggtccatttggattaatcatttcctattctattgctgcgggcgtagttgaaggaaaactgattcc<br>agaattgaagaacaagttaaaatctgtgcagaatttctttaccaccctgtctaacacggttaaa<br>caagcgaataaagatatcgatgccgccaaattgaaattaaccaccgaaatagccgccatcg<br>gtgagataaaaacggaaactgaaacaaccagattctacgttgattatgatgatttaatgctttct<br>ttgctaaaagaagcggccaaaaaaatgattaacacctgtaatgagtatcagaaaagacacg<br>gtaaaaagacactctttgaggtacctgaagtcggaggctacccatacgatgttccagattac<br>gcttacccatacgatgttccagattacgcttacccatacgatgttccagattacgctggaggc<br>ggatccgtgcctcctatggtgaacgtgacttgcagcgaggtgagcgagggcaatatcacc<br>gtgacctgccgggcctccagcttttacccaagaaatatcaccctgacctggaggcaggacg<br>gcgtgtctctgagccataacacccagcagtggggcgacgtgctgcccgatggaaacggc<br>acctatcagacatgggtggctacaagaatccggcagggagaggaacagagattcacctgc<br>tatatggagcacagcggaaaccatggaacacaccccgtgccatccggaaaagccctggtg<br>ctgcagtcccagtaatag
5. pET28a-ClyA-MICA (a3)-<br>SHNTQQ(del)	the sequence of ClyA-HA-MICB (a3)-SHNTQQ(del) as shown in the bottom was inserted into the pET28a back bone.<br>atgactgaaatcgttgcagataaaacggtagaagtagttaaaaacgcaatcgaaaccgcag<br>atggagcattagatctttataataaatatctcgatcaggtcatcccctggcagacctttgatgaa<br>accataaaagagttaagtcgctttaaacaggagtattcacaggcagcctccgttttagtcggc<br>gatattaaaaccttacttatggatagccaggataagtattttgaagcaacccaaacagtgtatg<br>aatggtgtggtgttgcgacgcaattgctcgcagcgtatattttgctatttgatgagtacaatga<br>gaagaaagcatccgcccagaaagacattctcattaaggtactggatgacggcatcacgaa<br>gctgaatgaagcgcaaaaatccctgctggtaagctcacaaagtttcaacaacgcttccggg<br>aaactgctggcgttagatagccagttaaccaatgatttttcagaaaaaagcagctatttccagt<br>cacaggtagataaaatcaggaaggaagcatatgccggtgccgcagccggtgtcgtcgcc<br>ggtccatttggattaatcatttcctattctattgctgcgggcgtagttgaaggaaaactgattcc<br>agaattgaagaacaagttaaaatctgtgcagaatttctttaccaccctgtctaacacggttaaa<br>caagcgaataaagatatcgatgccgccaaattgaaattaaccaccgaaatagccgccatcg<br>gtgagataaaaacggaaactgaaacaaccagattctacgttgattatgatgatttaatgctttct<br>ttgctaaaagaagcggccaaaaaaatgattaacacctgtaatgagtatcagaaaagacacg<br>gtaaaaagacactctttgaggtacctgaagtcggaggctacccatacgatgttccagattac<br>gcttacccatacgatgttccagattacgcttacccatacgatgttccagattacgctggaggc<br>ggatccgtgcctcctatggtgaacgtgacttgcagcgaggtgagcgagggcaatatcacc<br>gtgacctgccgggcctccagcttttacccaagaaatatcaccctgacctggaggcaggacg<br>gcgtgtctctgtggggcgacgtgctgcccgatggaaacggcacctatcagacatgggtgg<br>ctacaagaatccggcagggagaggaacagagattcacctgctatatggagcacagcgga<br>aaccatggaacacaccccgtgccatccggaaaagccctggtgctgcagtcccagtaatag

Appendix Table S3. The sequences of the anti-MICA α3 antibodies: 1D5 and 13A9

1D5	Light Chain:<br>mgwsciilflvatatgvhseiiltqsptttmaaspgekititcsasssisshylhwyqqksgfspklliyrt<br>snlasgvparfsgsgsgtsysltigtmeaedvatyycqqgsslpltfgagtkleikrtvaapsvfifpps<br>deqlksgtasvvcllnnfypreakvqwkvdnalqsgnsqesvteqdskdstyslsstltlskadyekh<br>kvyacevthqglsspvtksfnrgec<br><br>Heavy Chain:<br>mgwsciilflvatatgvhseiqlqqsgpelvkpgasvkvsckasgyaftsqniywvkqshgkslew<br>igyiepynvvpmynpkfkgkatltvdkssssayihlnsltsedsaiyycarsgssnfdywgqgttltv<br>ssastkgpsvfplapsskstsggtaalgclvkdyfpepvtvswnsgaltsgvhtfpavlqssglyslss<br>vvtvpssslgtqtyicnvnhkpsntkvdkkvepkscdkthtcppcpapellggpsvflfppkpkdtl<br>misrtpevtcvvvdvshedpevkfnwyvdgvevhnaktkpreeqynstyrvvsvltvlhqdwln<br>gkeykckvsnkalpapiektiskakgqprepqvytlppsrdeltknqvsltclvkgfypsdiavewe<br>sngqpennykttppvldsdgsfflyskltvdksrwqqgnvfscsvmhealhnhytqkslslspgk
13A9	Light Chain:<br>mgwsciilflvatatgvhsdiqmtqspaslsasvgetvtitcrasgnihsylawyqqkqgkspqllvy<br>yaetladgvpsrfsgrgsgtqyslkinslqpedfgsyfcqqfwttpytfgggtkveikrtvaapsvfifp<br>psdeqlksgtasvvcllnnfypreakvqwkvdnalqsgnsqesvteqdskdstyslsstltlskadye<br>khkvyacevthqglsspvtksfnrgec<br><br>Heavy Chain:<br>mgwsciilflvatatgvhsqvqlqqsgaelvrpgtsvkvsckasgyaftnyliewvkqrpgqglewi<br>gainpgsgatnynekfkdkarltadkssntaylqfssltsddsavyfcarflgnyfdnwgqgatltvss<br>astkgpsvfplapsskstsggtaalgclvkdyfpepvtvswnsgaltsgvhtfpavlqssglyslssvv<br>tvpssslgtqtyicnvnhkpsntkvdkkvepkscdkthtcppcpapellggpsvflfppkpkdtlmi<br>srtpevtcvvvdvshedpevkfnwyvdgvevhnaktkpreeqynstyrvvsvltvlhqdwlngk<br>eykckvsnkalpapiektiskakgqprepqvytlppsrdeltknqvsltclvkgfypsdiavewesn<br>gqpennykttppvldsdgsfflyskltvdksrwqqgnvfscsvmhealhnhytqkslslspgk

Appendix Table S4. Statistical details for box plots in Figures 5F and 5G

		minima	maxima	centre	lower_whisker	upper_whisker	Percentile_25%, 75%
MHC-I	CTRL	0	4.940	3.061	1.505	4.570	2.654, 3.421
	OMICBvac	0	4.706	3.098	1.630	4.482	2.699, 3.412
	ONEOvac	0	4.468	2.962	1.416	4.420	2.542, 3.294
	Combined	0	4.668	3.333	2.121	4.514	3.019, 3.617
MHC-II	CTRL	0	3.460	0	0	1.053	0, 2.633
	OMICBvac	0	3.583	0	0	1.390	0, 3.475
	ONEOvac	0	3.982	0	0	1.100	0, 2.749
	Combined	0	3.871	1.028	0	1.667	0, 3.871
Pyroptosis Score	CTRL	-0.306	0.507	-0.072	-0.306	0.179	-0.127, -0.005
	OMICBvac	-0.335	0.491	-0.056	-0.318	0.219	-0.116, 0.018
	ONEOvac	-0.289	0.457	-0.025	-0.289	0.292	-0.096, 0.059
	Combined	-0.308	0.435	-0.031	-0.308	0.266	-0.097, 0.048
