# Supplementary figures and images for: Neoantigens and shared MICB α3 antigen dual-targeted vaccine generates potent antitumor immunity (part 1 of 2)
Source: EMBO Mol Med. 2026 Apr 17;18(6):2098–123. doi: 10.1038/s44321-026-00424-6 (PMC13269783; doi:10.1038/s44321-026-00424-6)

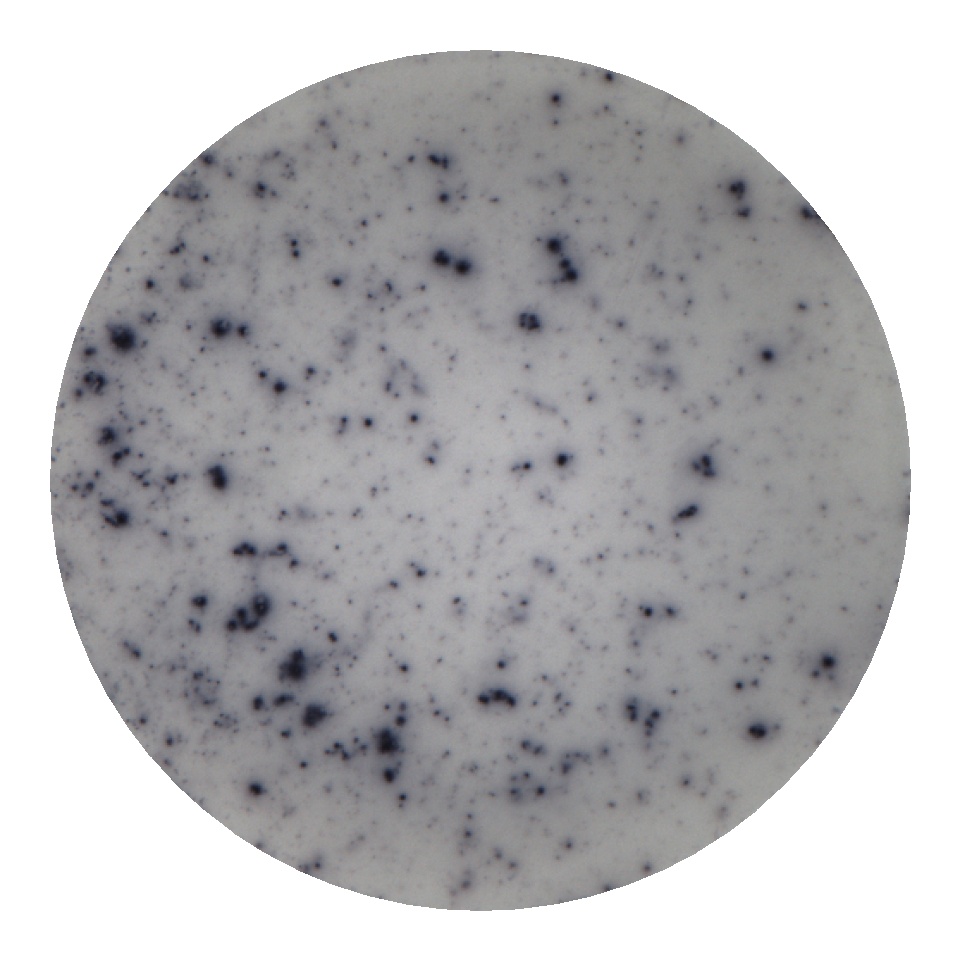

Supplement: Supplementary file 3 — Source data Fig. 1 [file 44321_2026_424_MOESM3_ESM.zip › Figure 1 Source Data/Figure 1I/5╬╝g 2.jpg]

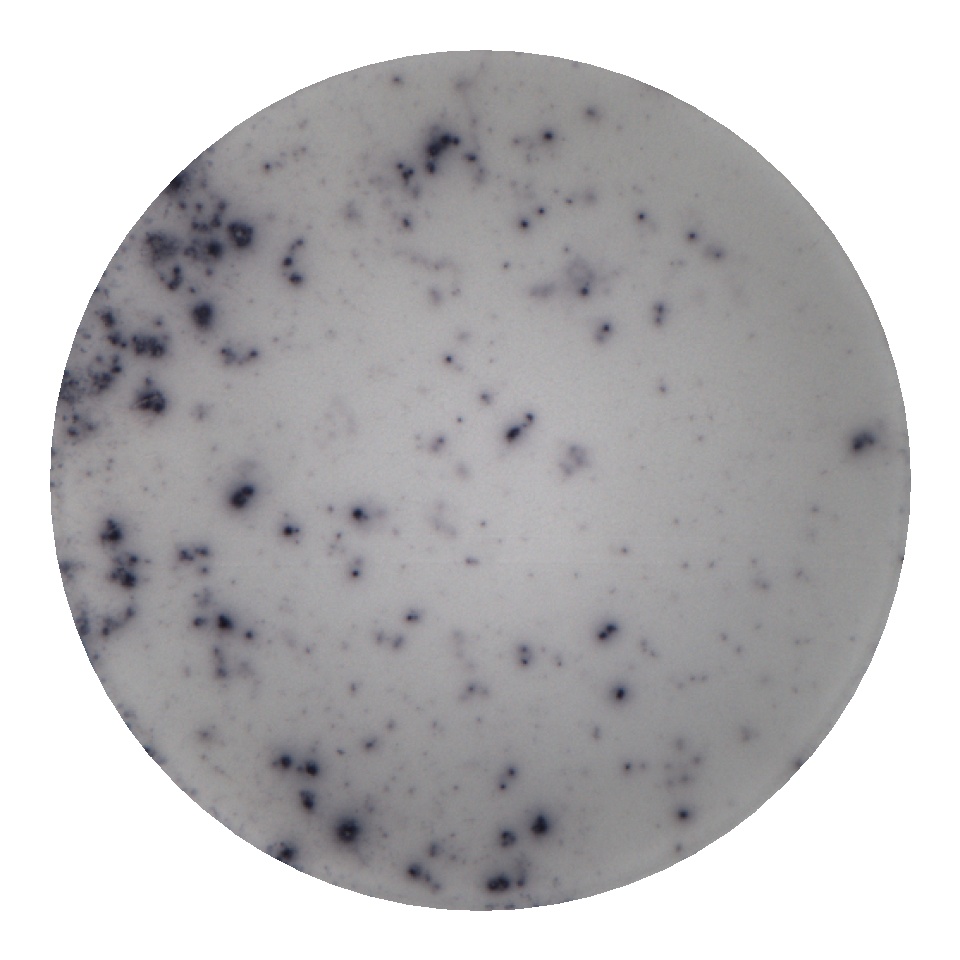

Supplement: Supplementary file 3 — Source data Fig. 1 [file 44321_2026_424_MOESM3_ESM.zip › Figure 1 Source Data/Figure 1I/5╬╝g 3.jpg]

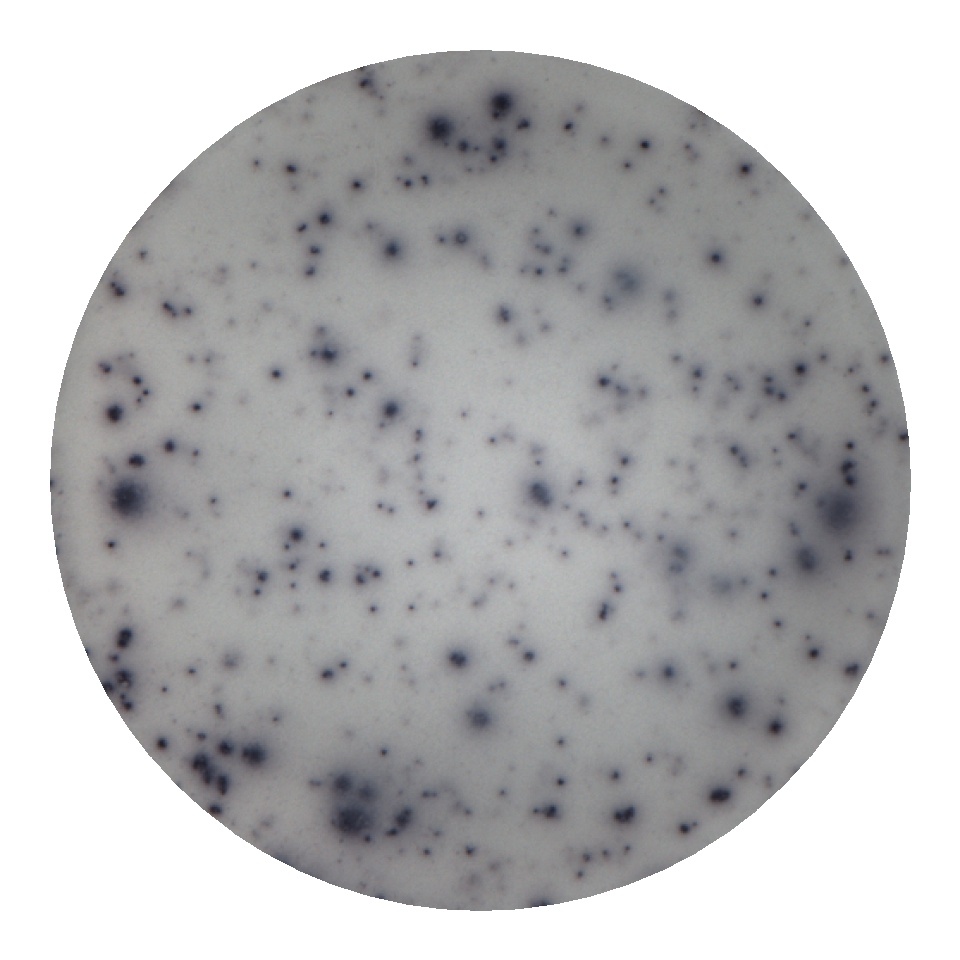

Supplement: Supplementary file 3 — Source data Fig. 1 [file 44321_2026_424_MOESM3_ESM.zip › Figure 1 Source Data/Figure 1I/20╬╝g 1.jpg]

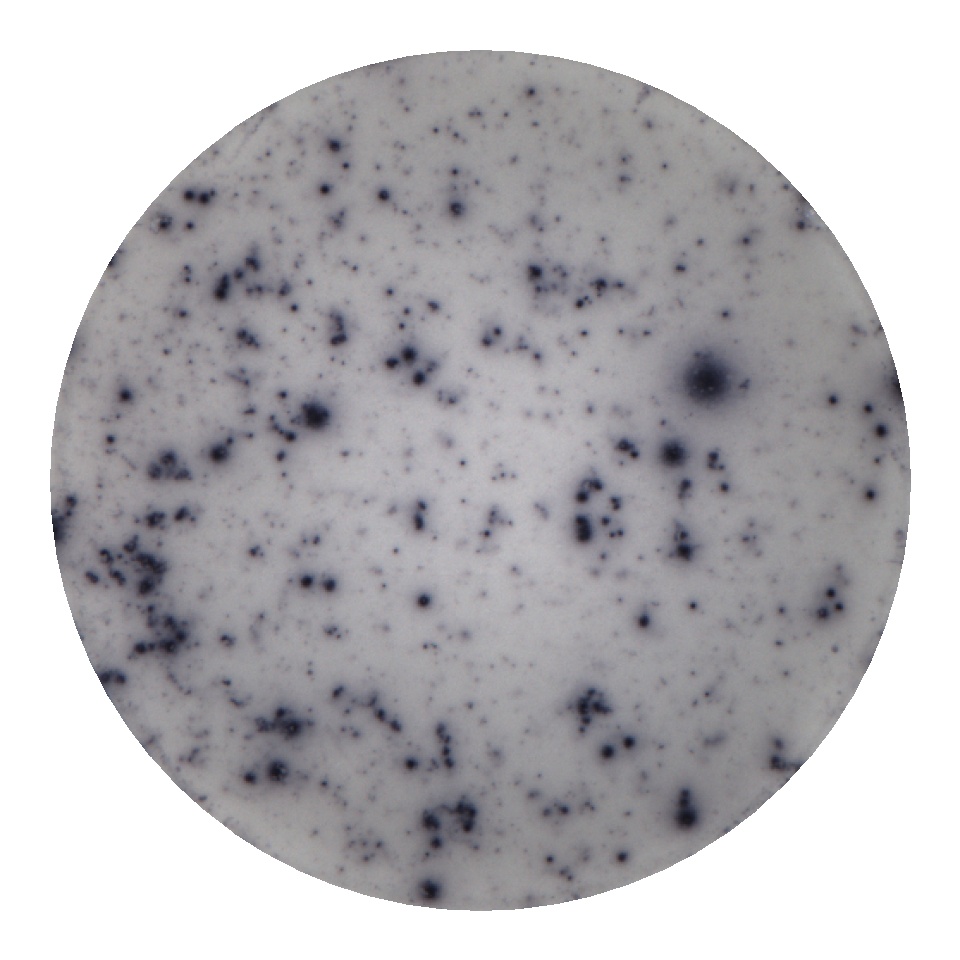

Supplement: Supplementary file 3 — Source data Fig. 1 [file 44321_2026_424_MOESM3_ESM.zip › Figure 1 Source Data/Figure 1I/20╬╝g 3.jpg]

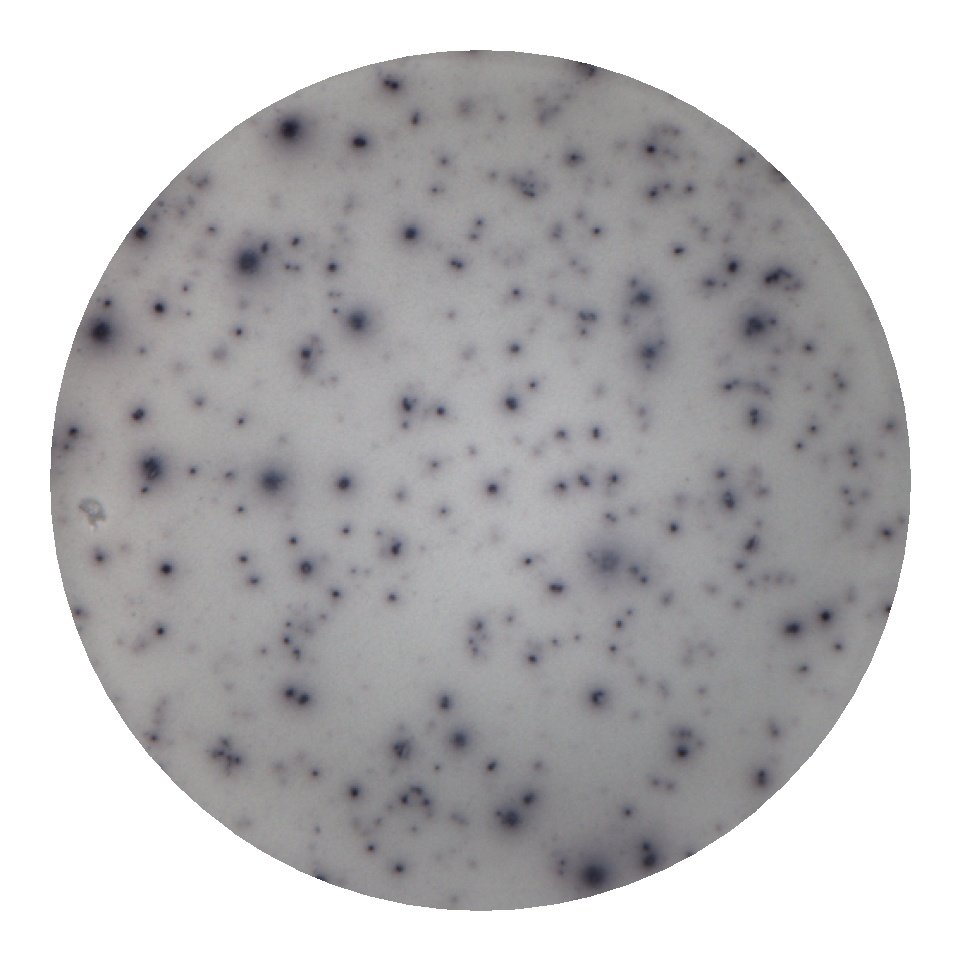

Supplement: Supplementary file 3 — Source data Fig. 1 [file 44321_2026_424_MOESM3_ESM.zip › Figure 1 Source Data/Figure 1I/5╬╝g 1.jpg]

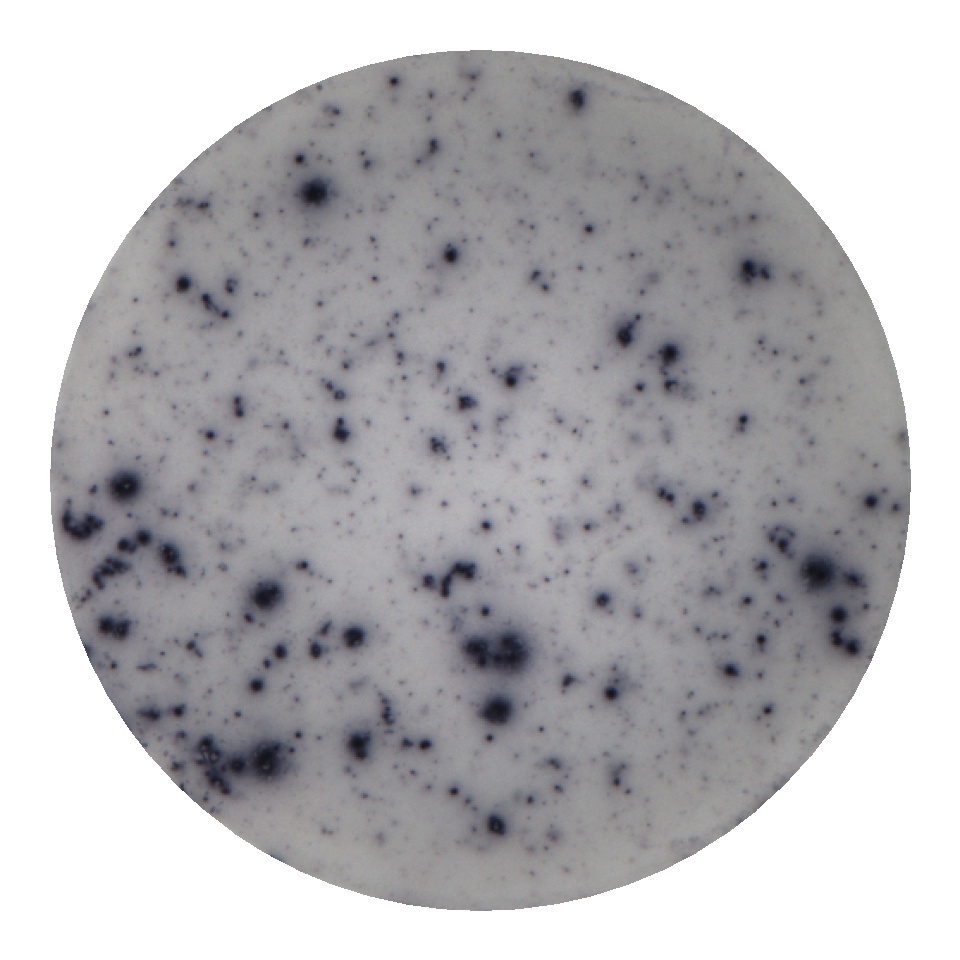

Supplement: Supplementary file 3 — Source data Fig. 1 [file 44321_2026_424_MOESM3_ESM.zip › Figure 1 Source Data/Figure 1I/20╬╝g 2.jpg]

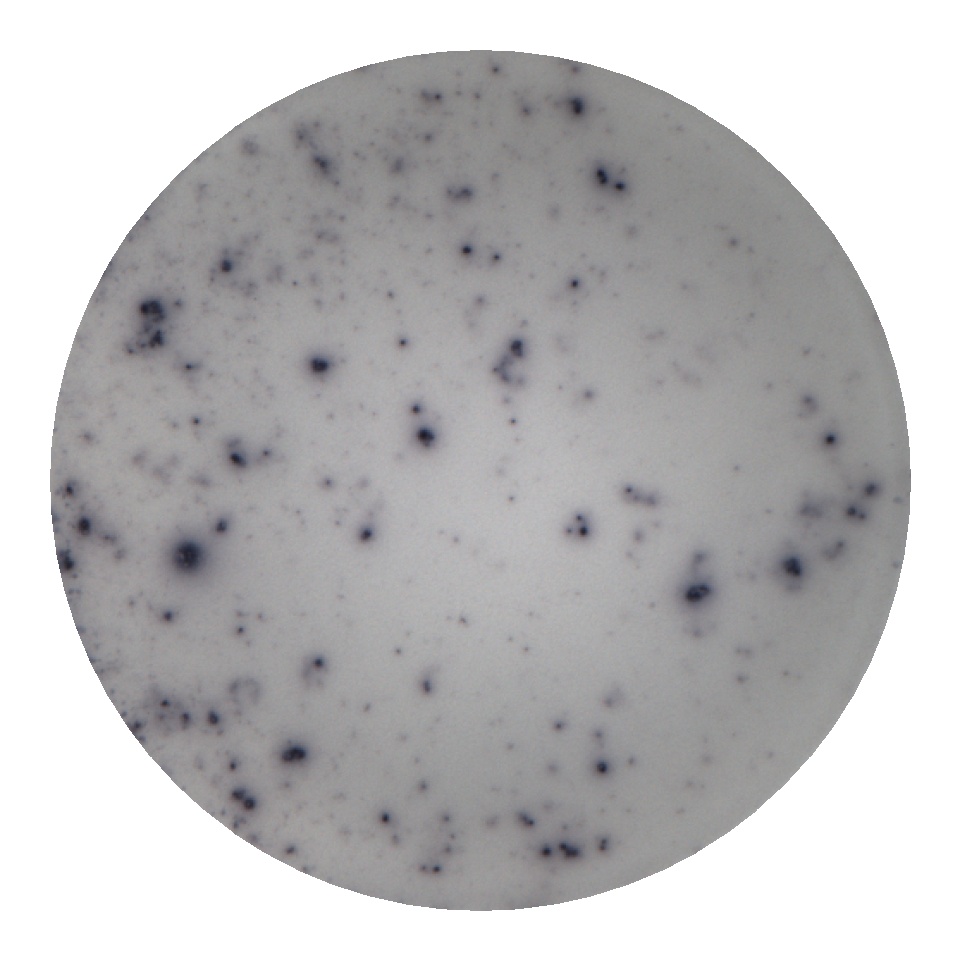

Supplement: Supplementary file 3 — Source data Fig. 1 [file 44321_2026_424_MOESM3_ESM.zip › Figure 1 Source Data/Figure 1I/2╬╝g 1.jpg]

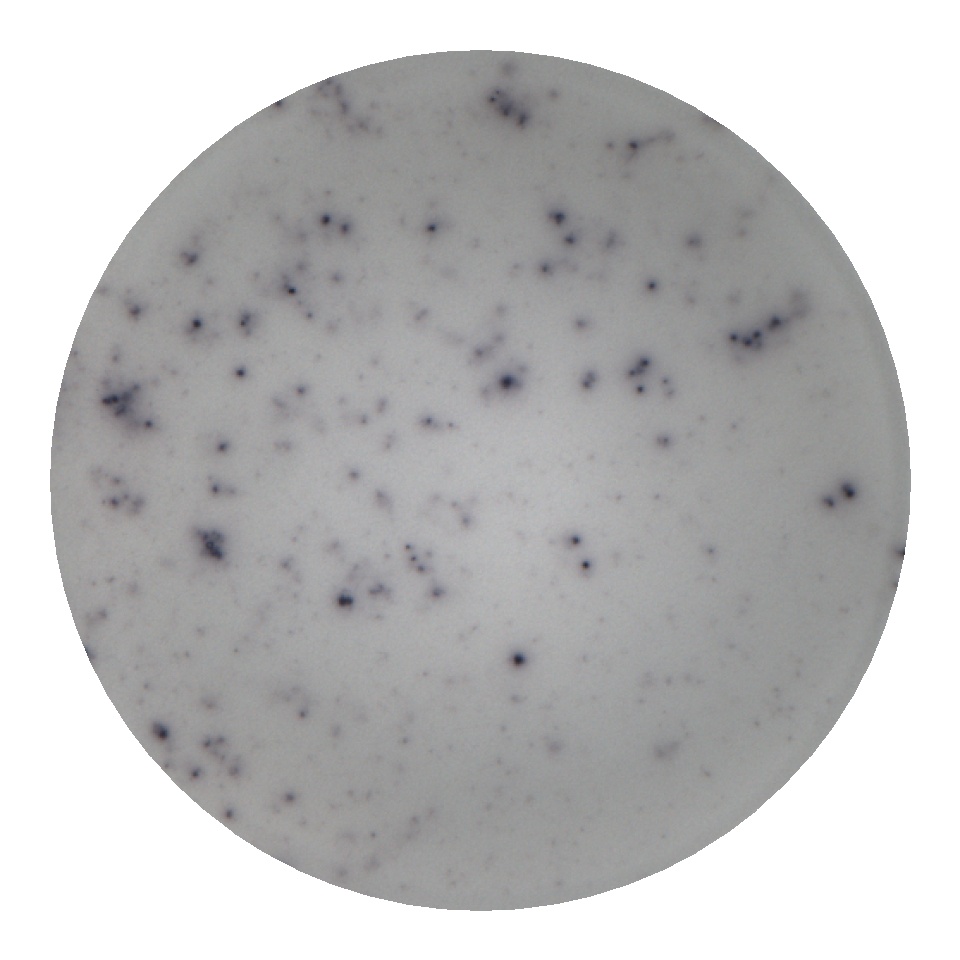

Supplement: Supplementary file 3 — Source data Fig. 1 [file 44321_2026_424_MOESM3_ESM.zip › Figure 1 Source Data/Figure 1I/2╬╝g 3.jpg]

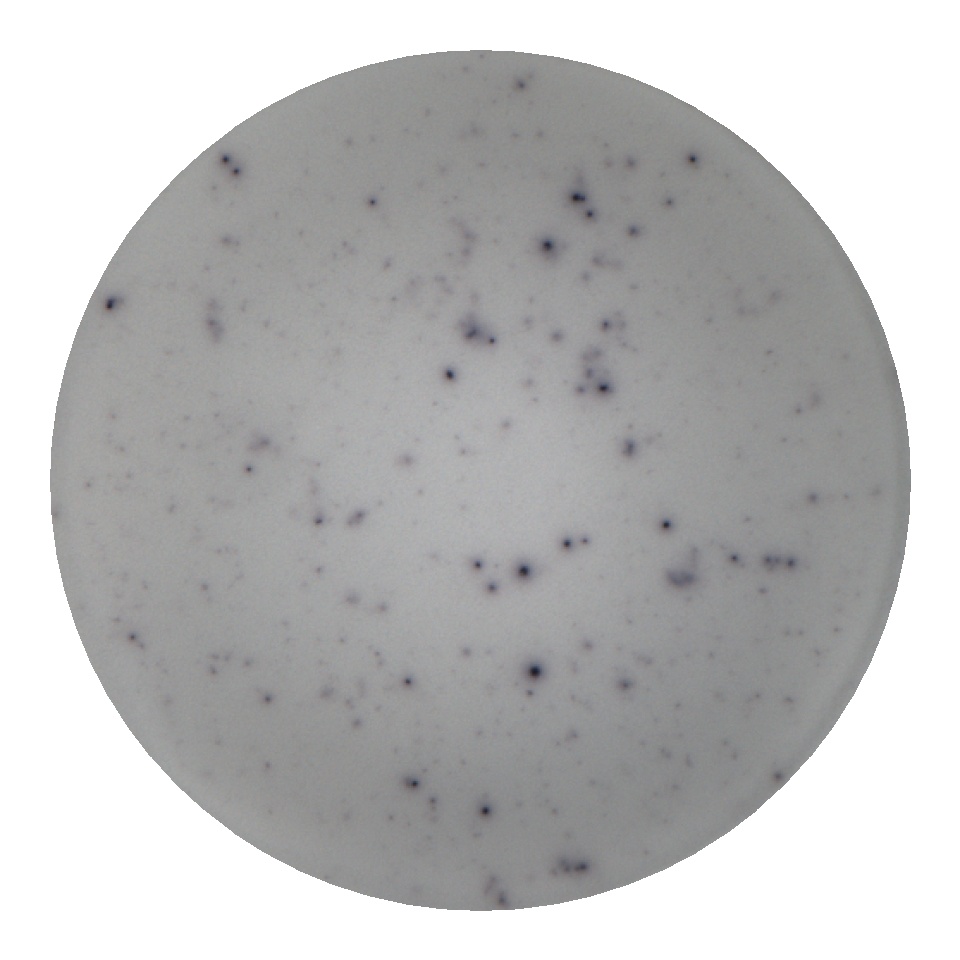

Supplement: Supplementary file 3 — Source data Fig. 1 [file 44321_2026_424_MOESM3_ESM.zip › Figure 1 Source Data/Figure 1I/2╬╝g 2.jpg]

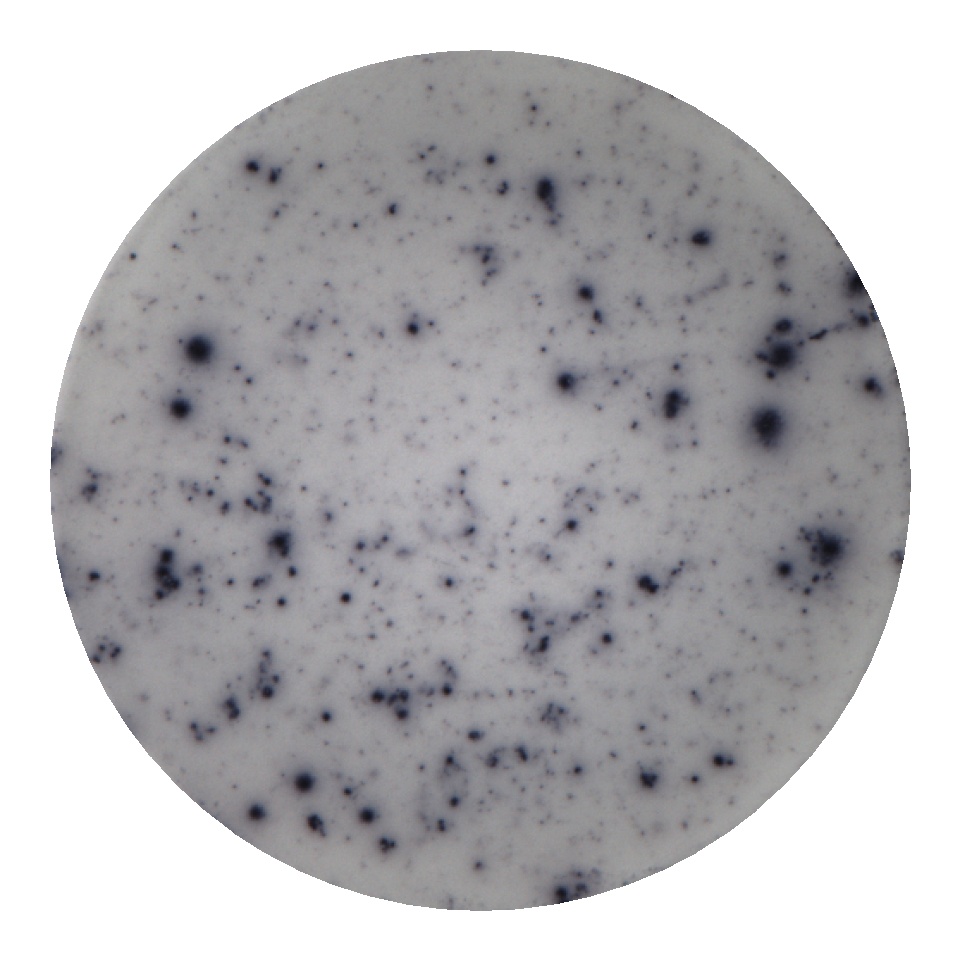

Supplement: Supplementary file 3 — Source data Fig. 1 [file 44321_2026_424_MOESM3_ESM.zip › Figure 1 Source Data/Figure 1I/10╬╝g 3.jpg]

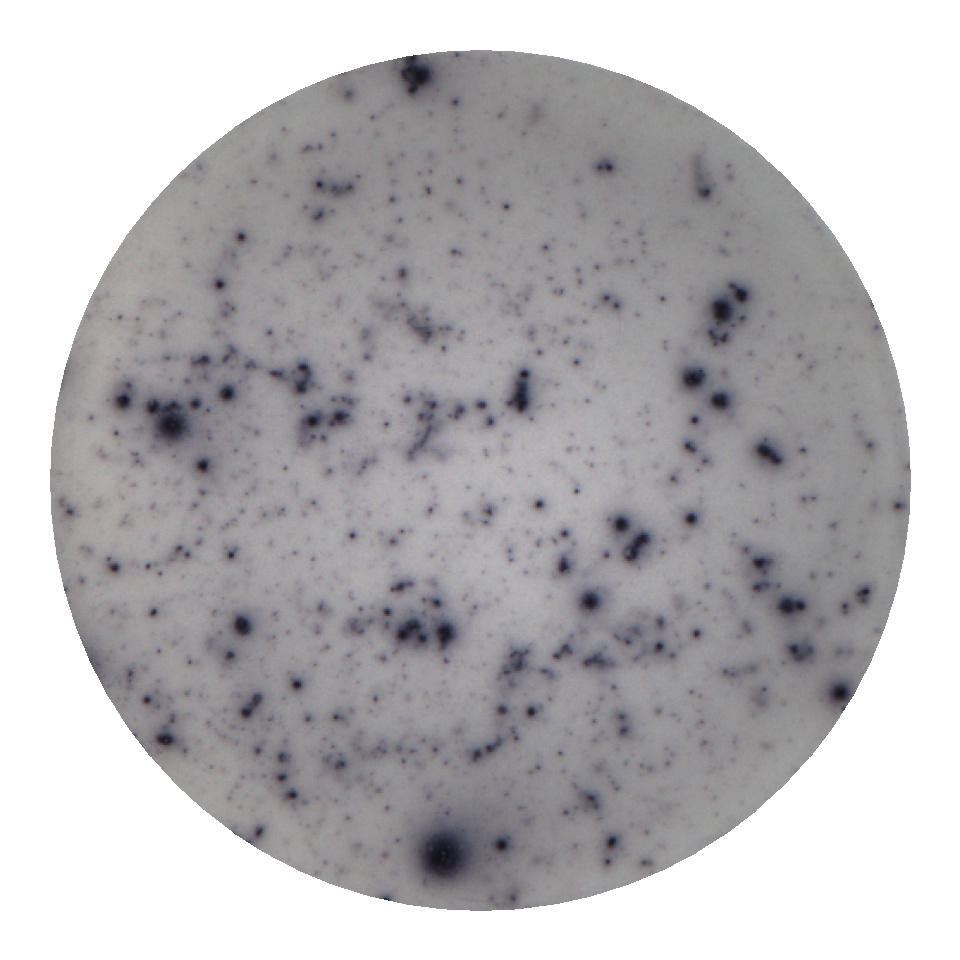

Supplement: Supplementary file 3 — Source data Fig. 1 [file 44321_2026_424_MOESM3_ESM.zip › Figure 1 Source Data/Figure 1I/10╬╝g 2.jpg]

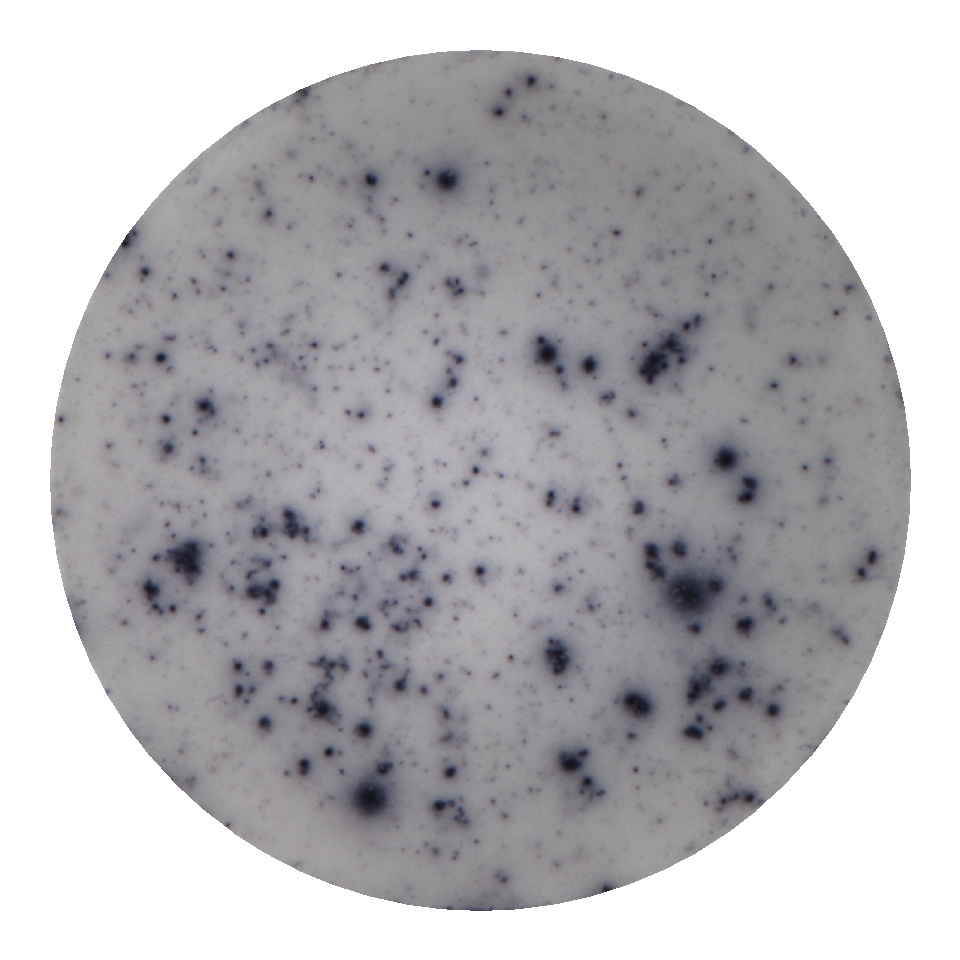

Supplement: Supplementary file 3 — Source data Fig. 1 [file 44321_2026_424_MOESM3_ESM.zip › Figure 1 Source Data/Figure 1I/10╬╝g 1.jpg]

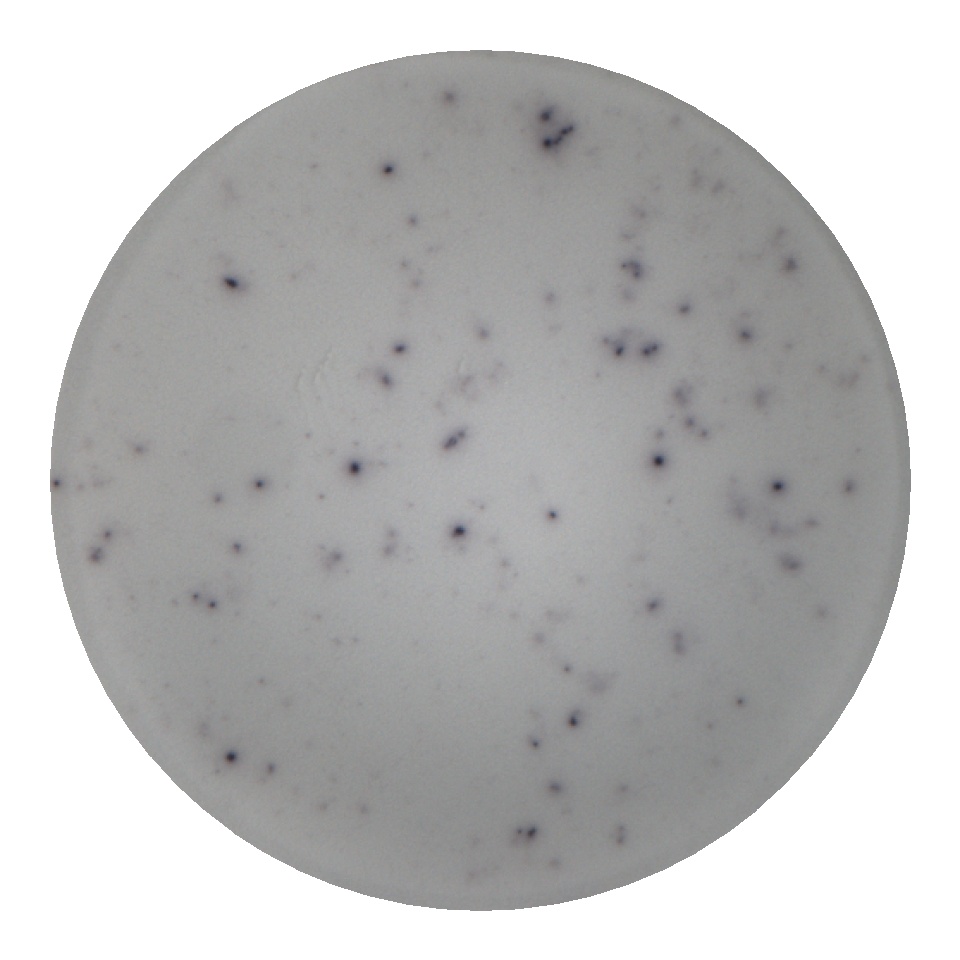

Supplement: Supplementary file 3 — Source data Fig. 1 [file 44321_2026_424_MOESM3_ESM.zip › Figure 1 Source Data/Figure 1I/OMVs 1.jpg]

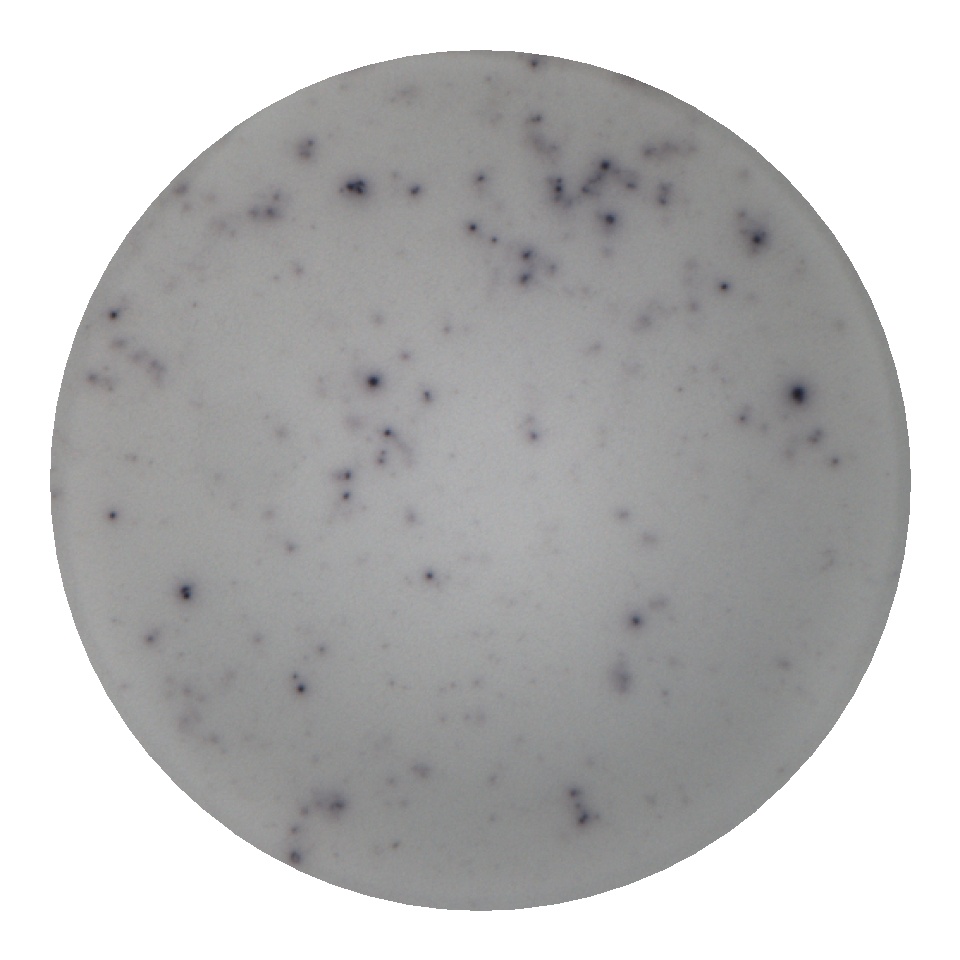

Supplement: Supplementary file 3 — Source data Fig. 1 [file 44321_2026_424_MOESM3_ESM.zip › Figure 1 Source Data/Figure 1I/OMVs 2.jpg]

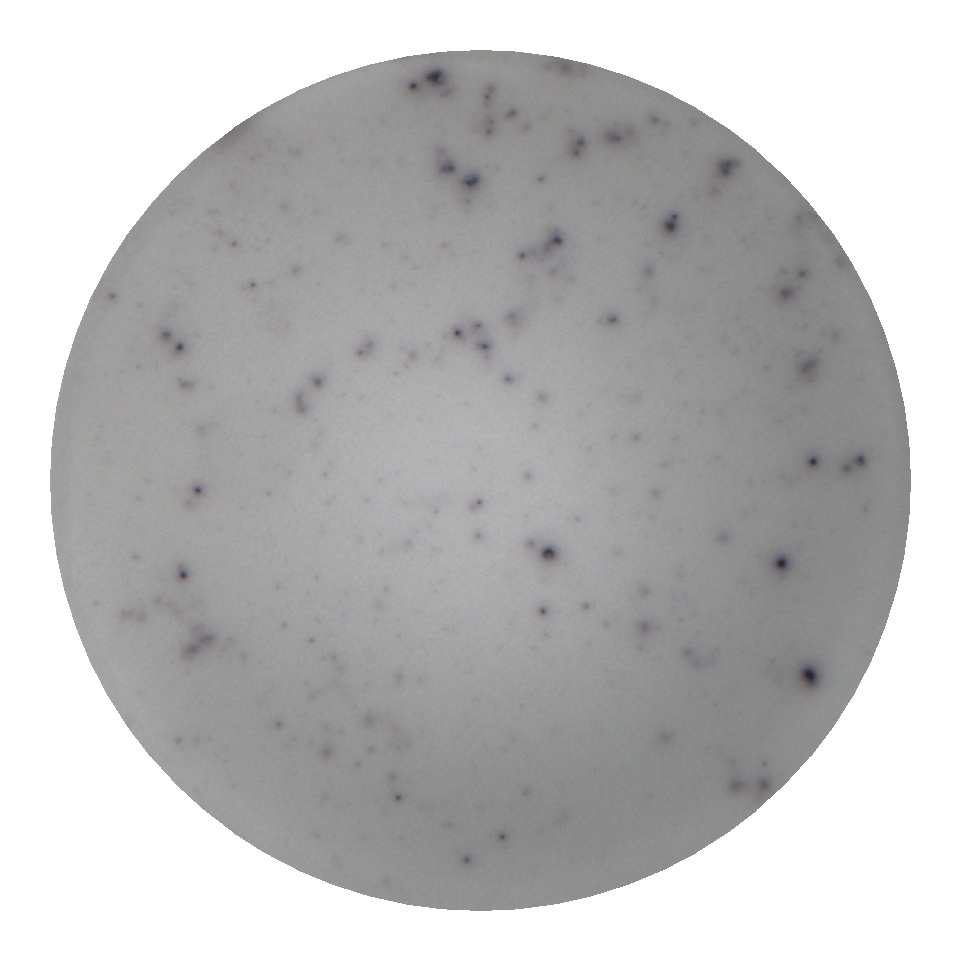

Supplement: Supplementary file 3 — Source data Fig. 1 [file 44321_2026_424_MOESM3_ESM.zip › Figure 1 Source Data/Figure 1I/OMVs 3.jpg]

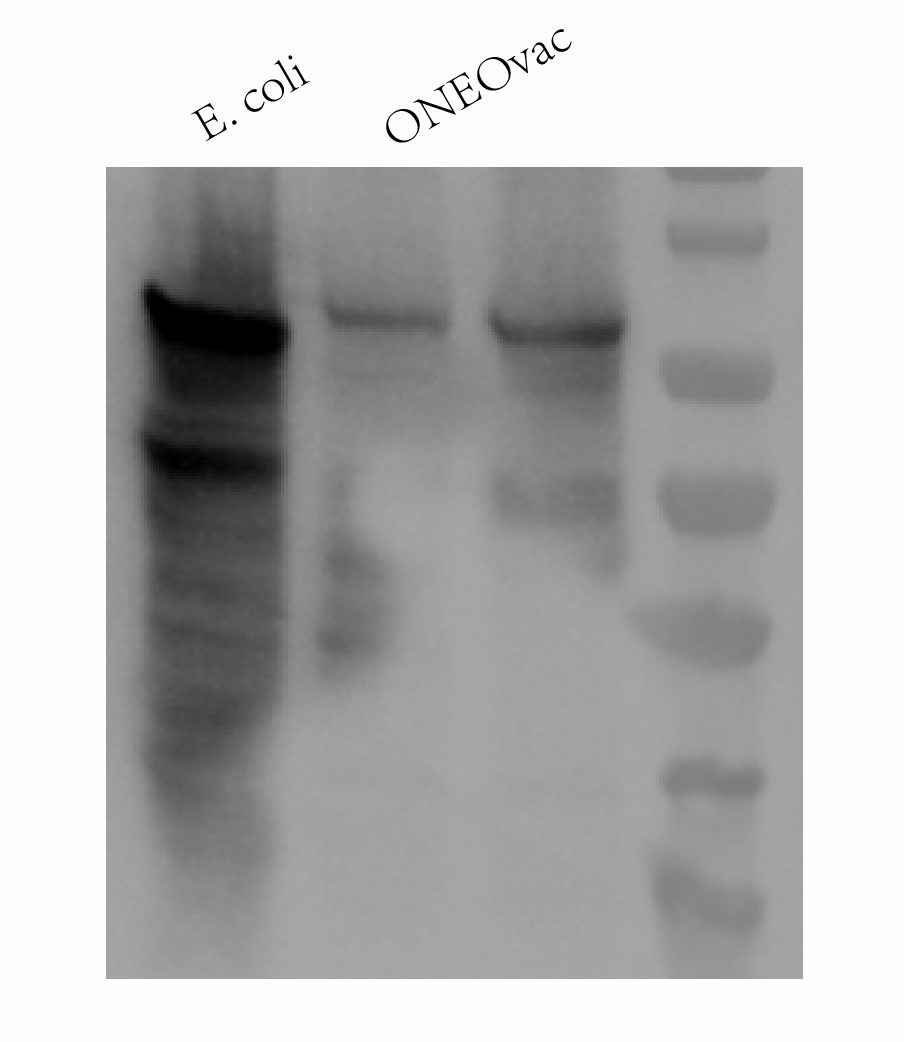

Supplement: Supplementary file 3 — Source data Fig. 1 [file 44321_2026_424_MOESM3_ESM.zip › Figure 1 Source Data/Figure 1C/Rosetta-nf-omv.png]

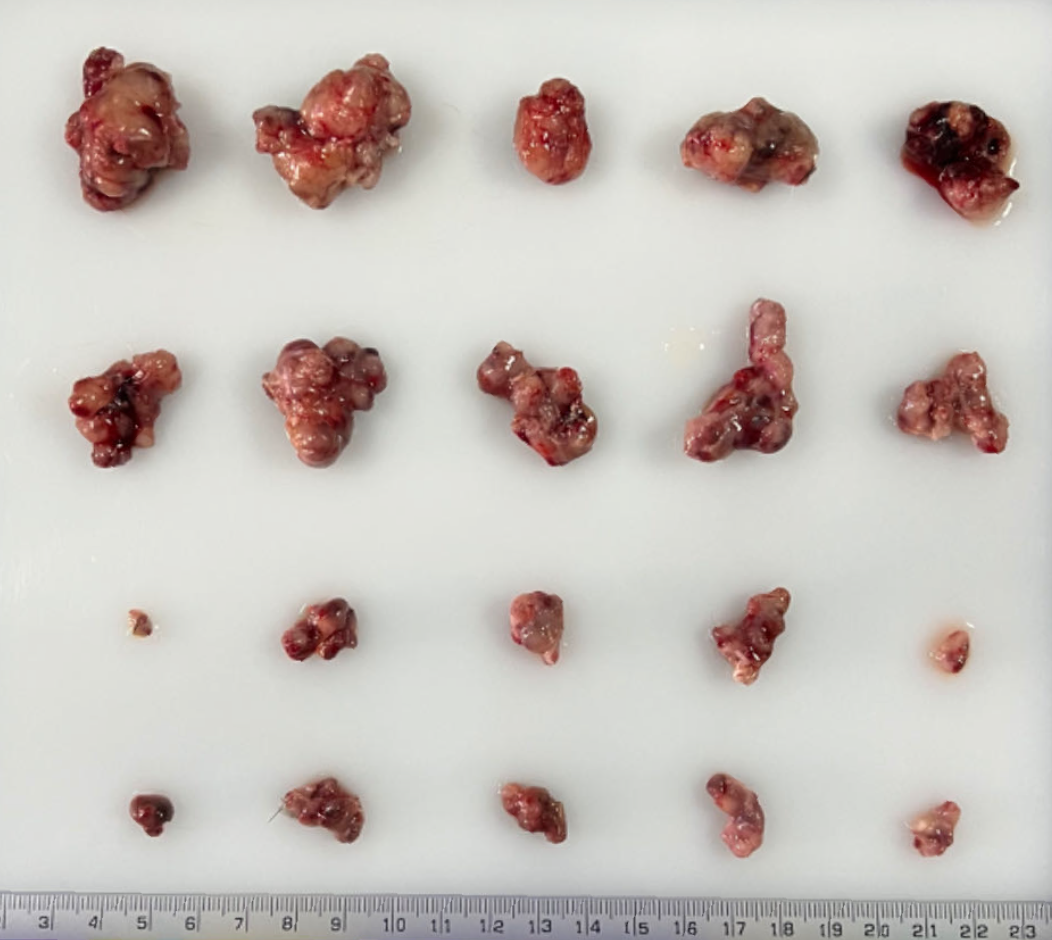

Supplement: Supplementary file 4 — Source data Fig. 2 [file 44321_2026_424_MOESM4_ESM.zip › Figure 2 Source Data/Figure 2C/2C.png]

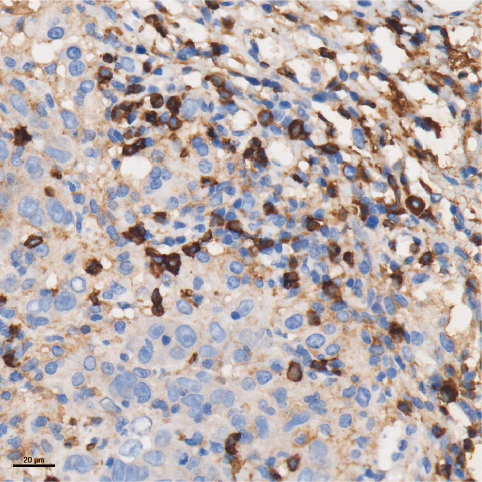

Supplement: Supplementary file 4 — Source data Fig. 2 [file 44321_2026_424_MOESM4_ESM.zip › Figure 2 Source Data/Figure 2D/CD4/OMICBvac 2.png]

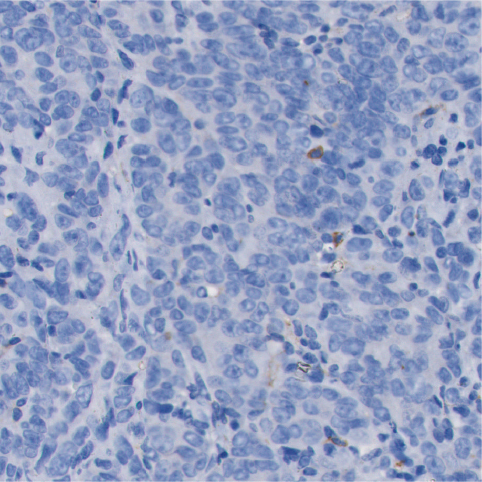

Supplement: Supplementary file 4 — Source data Fig. 2 [file 44321_2026_424_MOESM4_ESM.zip › Figure 2 Source Data/Figure 2D/CD4/CTRL 2.png]

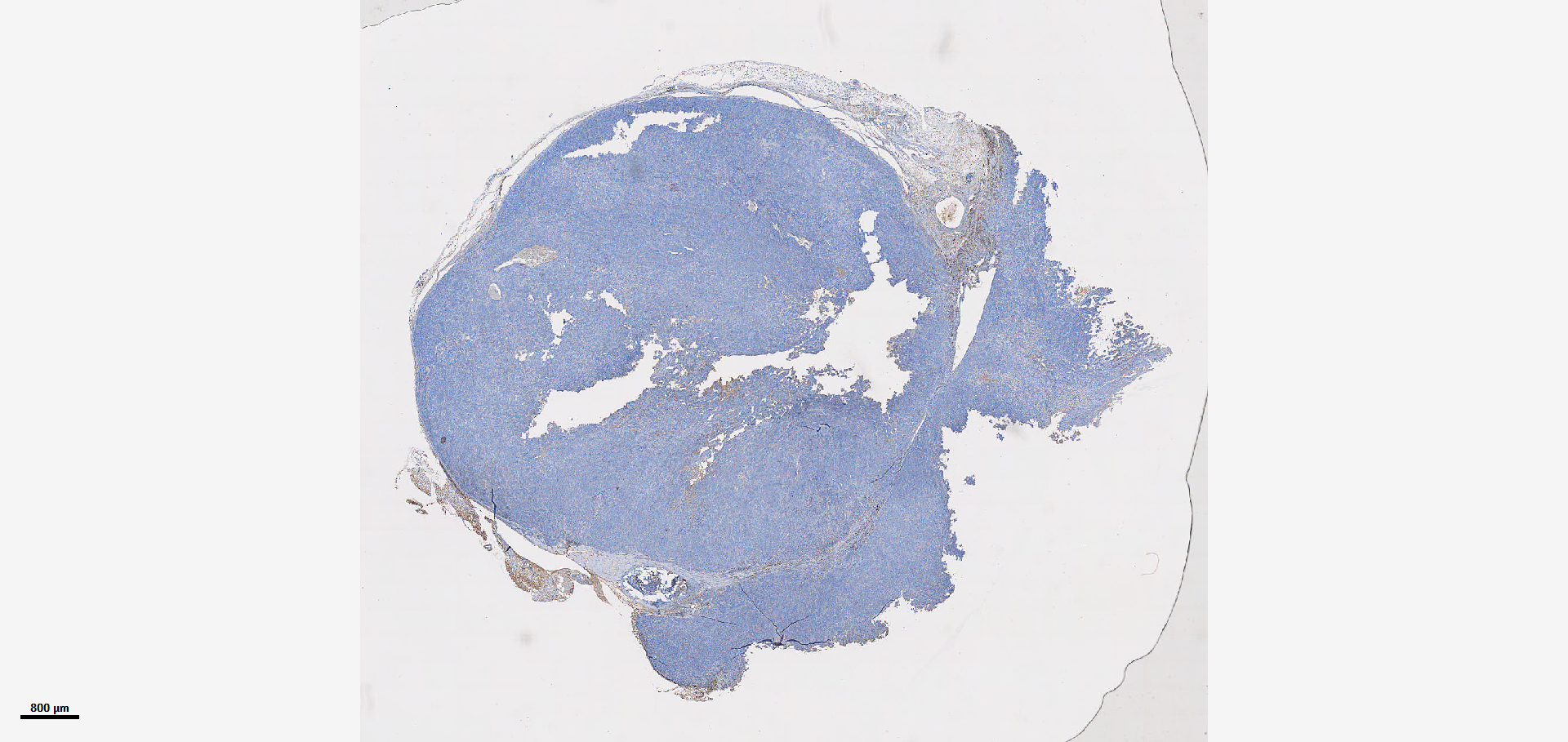

Supplement: Supplementary file 4 — Source data Fig. 2 [file 44321_2026_424_MOESM4_ESM.zip › Figure 2 Source Data/Figure 2D/CD4/CTRL 1.tif]

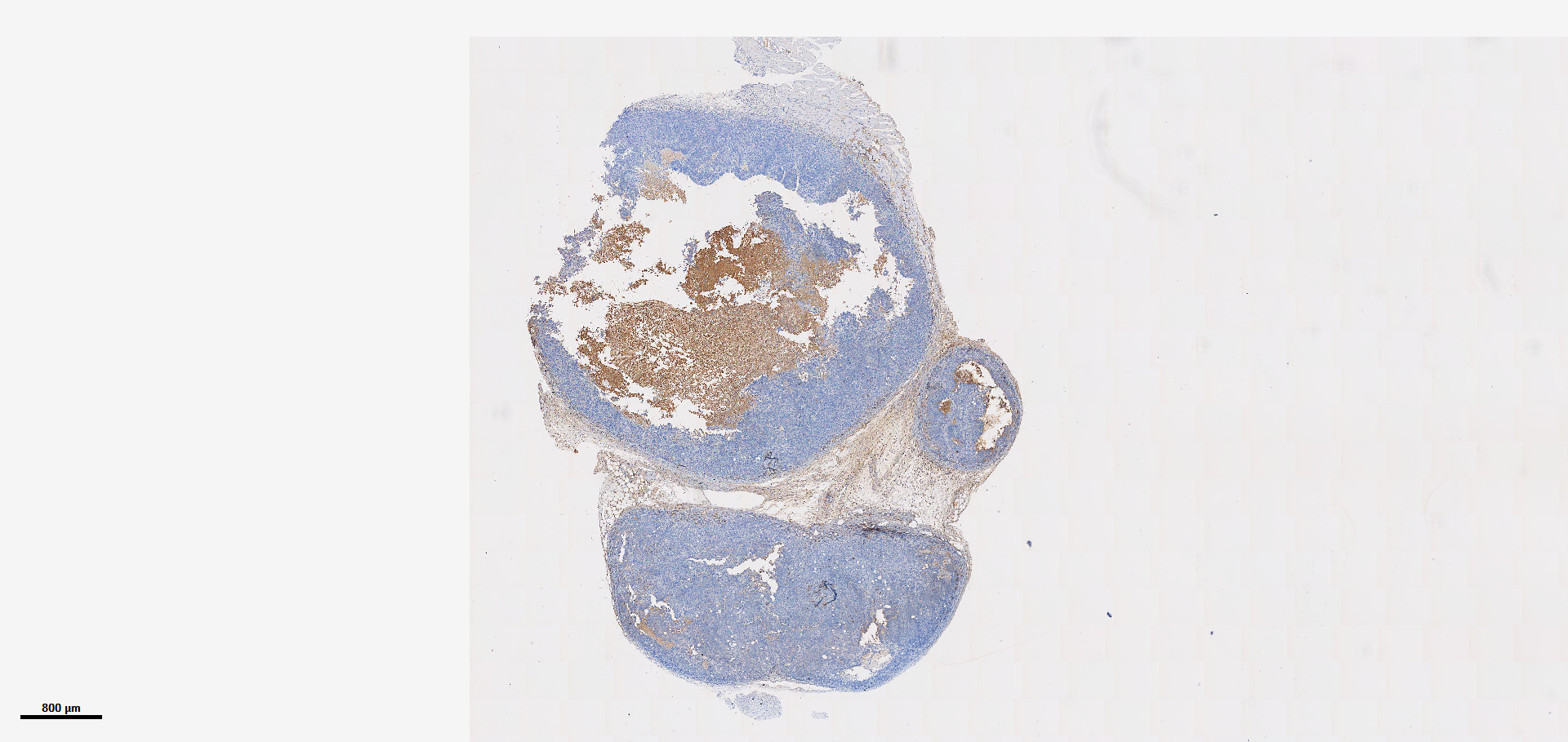

Supplement: Supplementary file 4 — Source data Fig. 2 [file 44321_2026_424_MOESM4_ESM.zip › Figure 2 Source Data/Figure 2D/CD4/OMICBvac 1.tif]

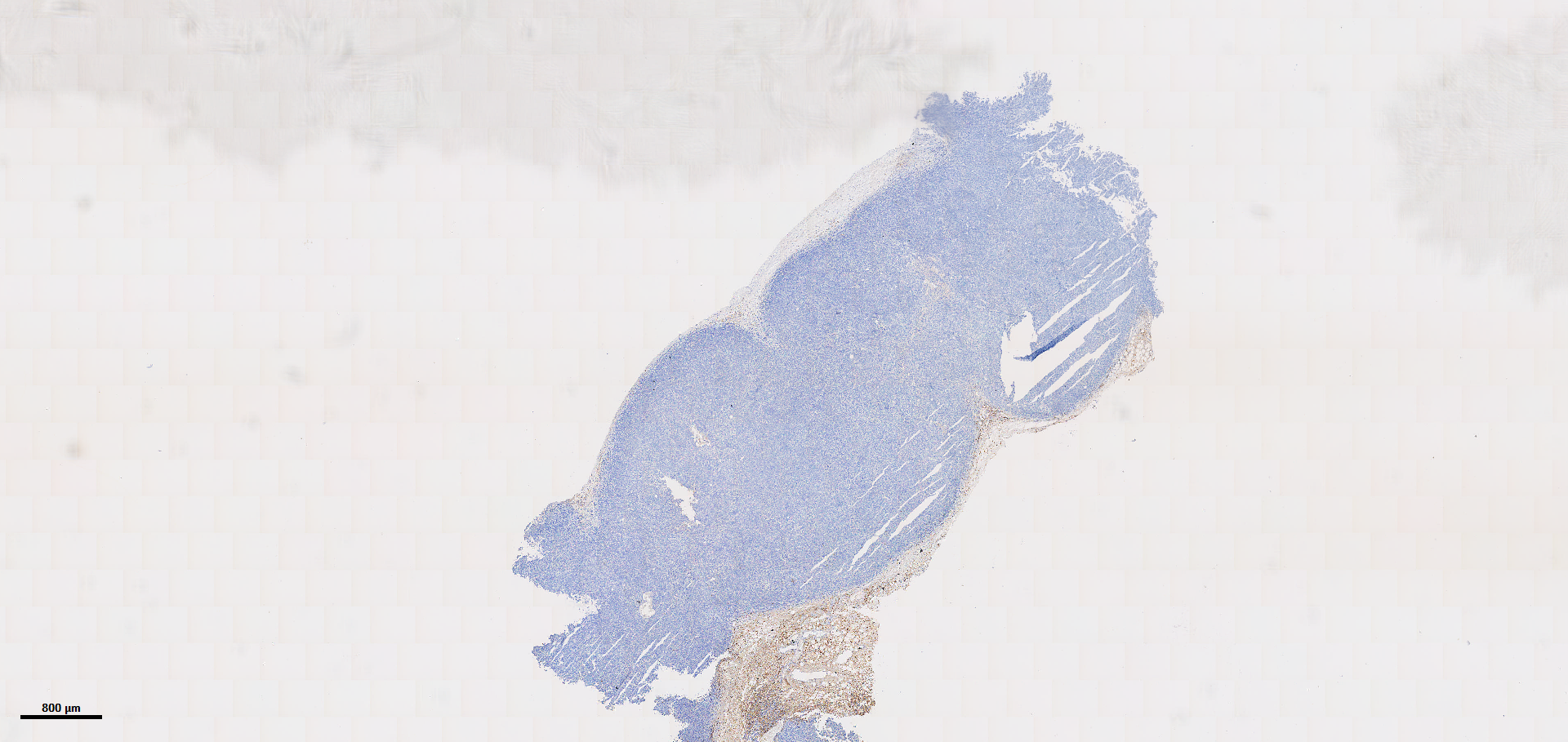

Supplement: Supplementary file 4 — Source data Fig. 2 [file 44321_2026_424_MOESM4_ESM.zip › Figure 2 Source Data/Figure 2D/CD4/OMVs 1.tif]

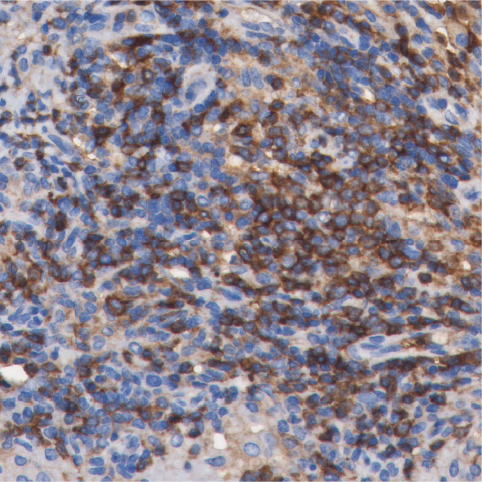

Supplement: Supplementary file 4 — Source data Fig. 2 [file 44321_2026_424_MOESM4_ESM.zip › Figure 2 Source Data/Figure 2D/CD4/ONEOvac 2.png]

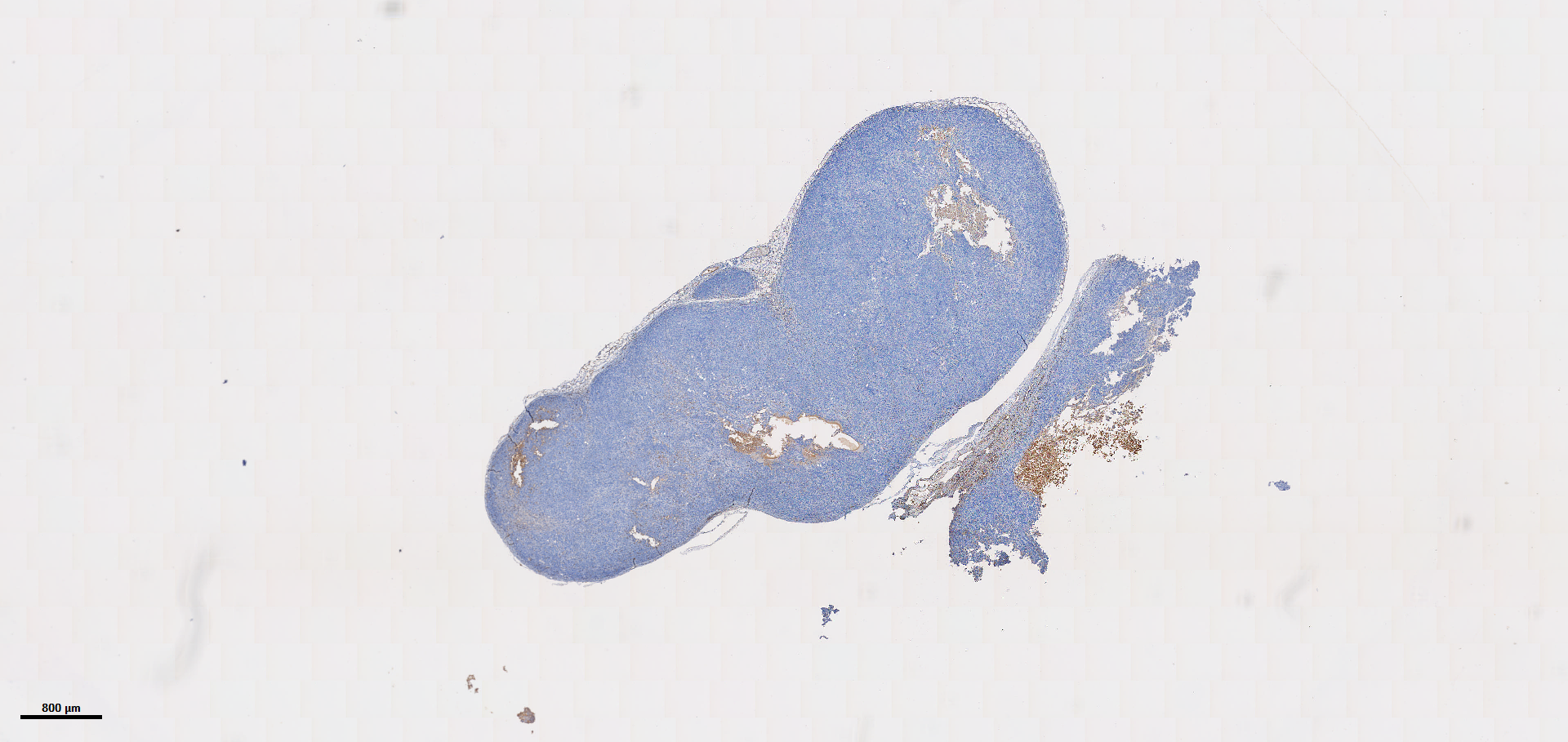

Supplement: Supplementary file 4 — Source data Fig. 2 [file 44321_2026_424_MOESM4_ESM.zip › Figure 2 Source Data/Figure 2D/CD4/ONEOvac 1.tif]

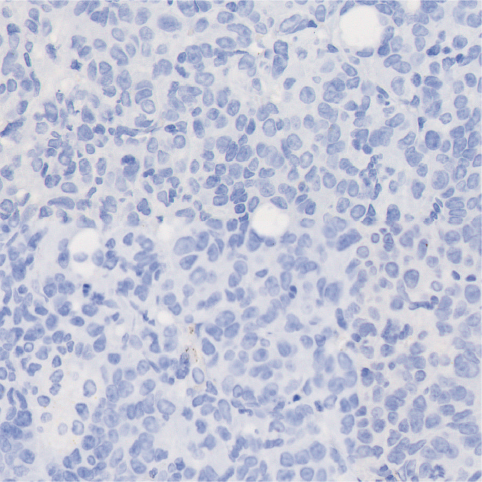

Supplement: Supplementary file 4 — Source data Fig. 2 [file 44321_2026_424_MOESM4_ESM.zip › Figure 2 Source Data/Figure 2D/CD4/OMVs 2.png]

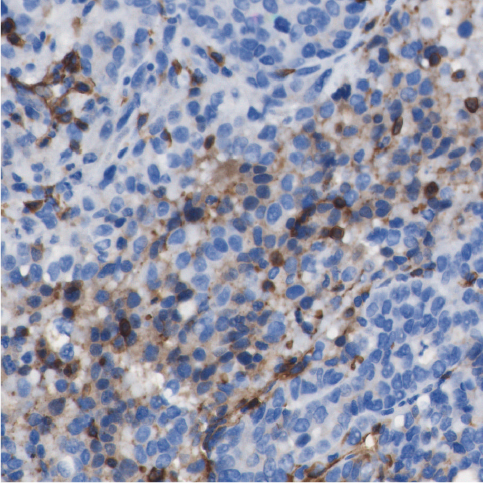

Supplement: Supplementary file 4 — Source data Fig. 2 [file 44321_2026_424_MOESM4_ESM.zip › Figure 2 Source Data/Figure 2D/CD8/OMICBvac 2.png]

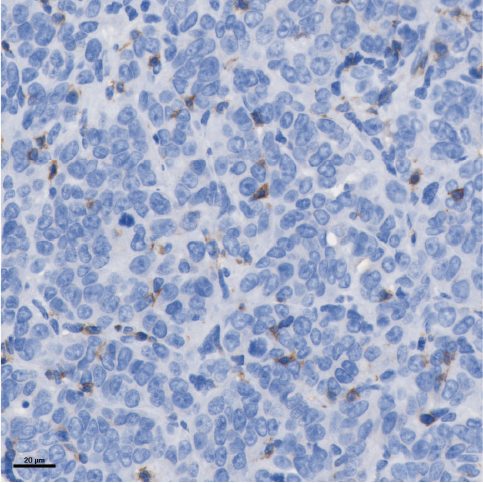

Supplement: Supplementary file 4 — Source data Fig. 2 [file 44321_2026_424_MOESM4_ESM.zip › Figure 2 Source Data/Figure 2D/CD8/CTRL 2.png]

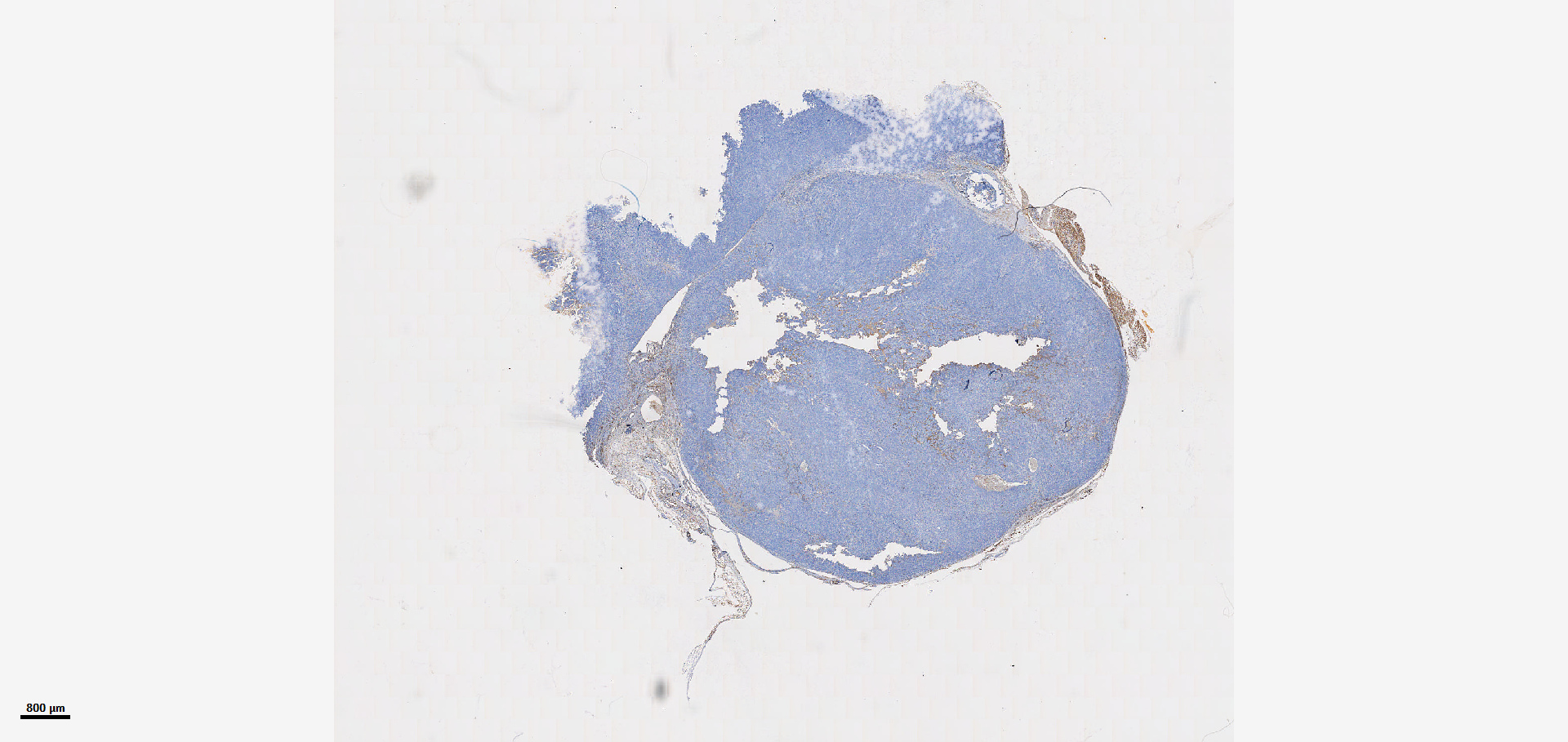

Supplement: Supplementary file 4 — Source data Fig. 2 [file 44321_2026_424_MOESM4_ESM.zip › Figure 2 Source Data/Figure 2D/CD8/CTRL 1.tif]

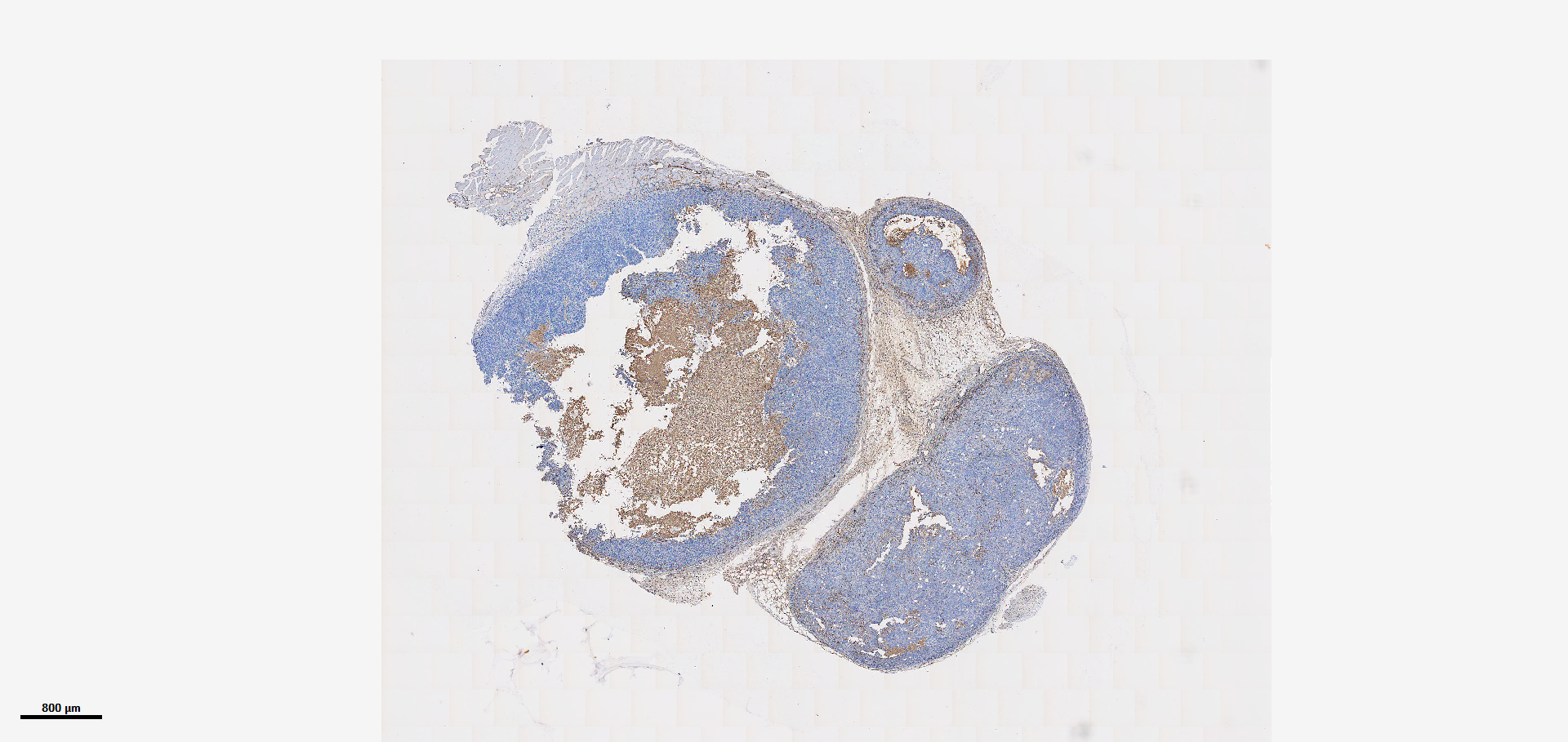

Supplement: Supplementary file 4 — Source data Fig. 2 [file 44321_2026_424_MOESM4_ESM.zip › Figure 2 Source Data/Figure 2D/CD8/OMICBvac 1.tif]

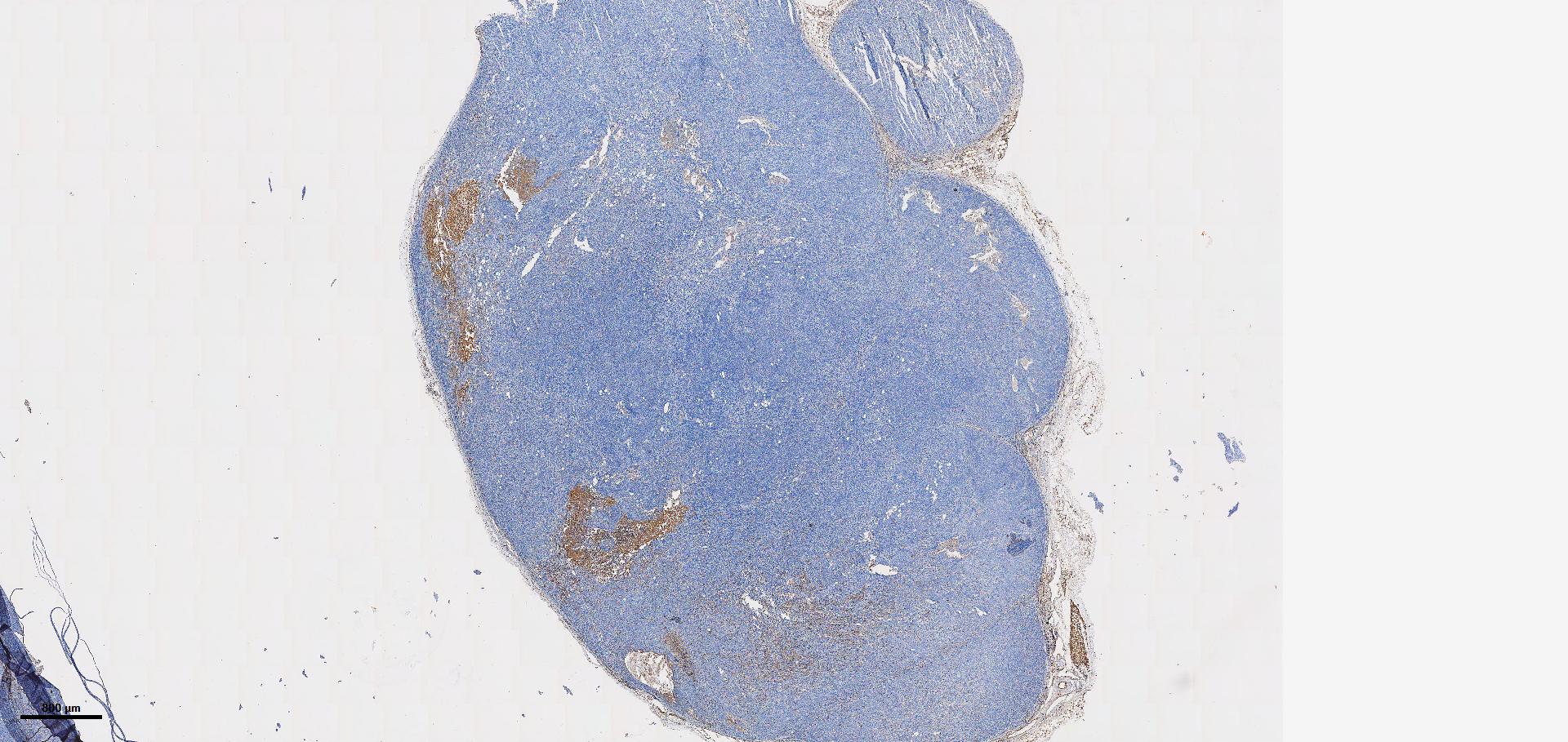

Supplement: Supplementary file 4 — Source data Fig. 2 [file 44321_2026_424_MOESM4_ESM.zip › Figure 2 Source Data/Figure 2D/CD8/OMVs 1.tif]

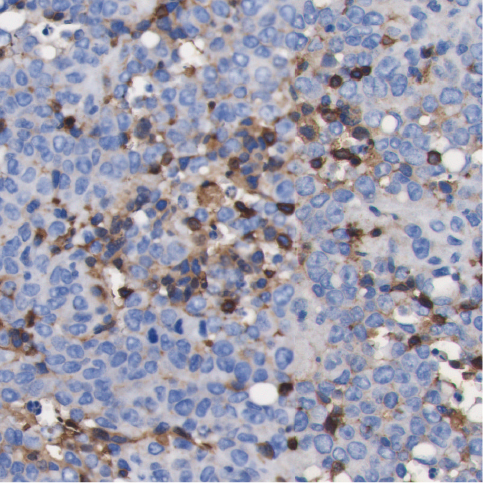

Supplement: Supplementary file 4 — Source data Fig. 2 [file 44321_2026_424_MOESM4_ESM.zip › Figure 2 Source Data/Figure 2D/CD8/ONEOvac 2.png]

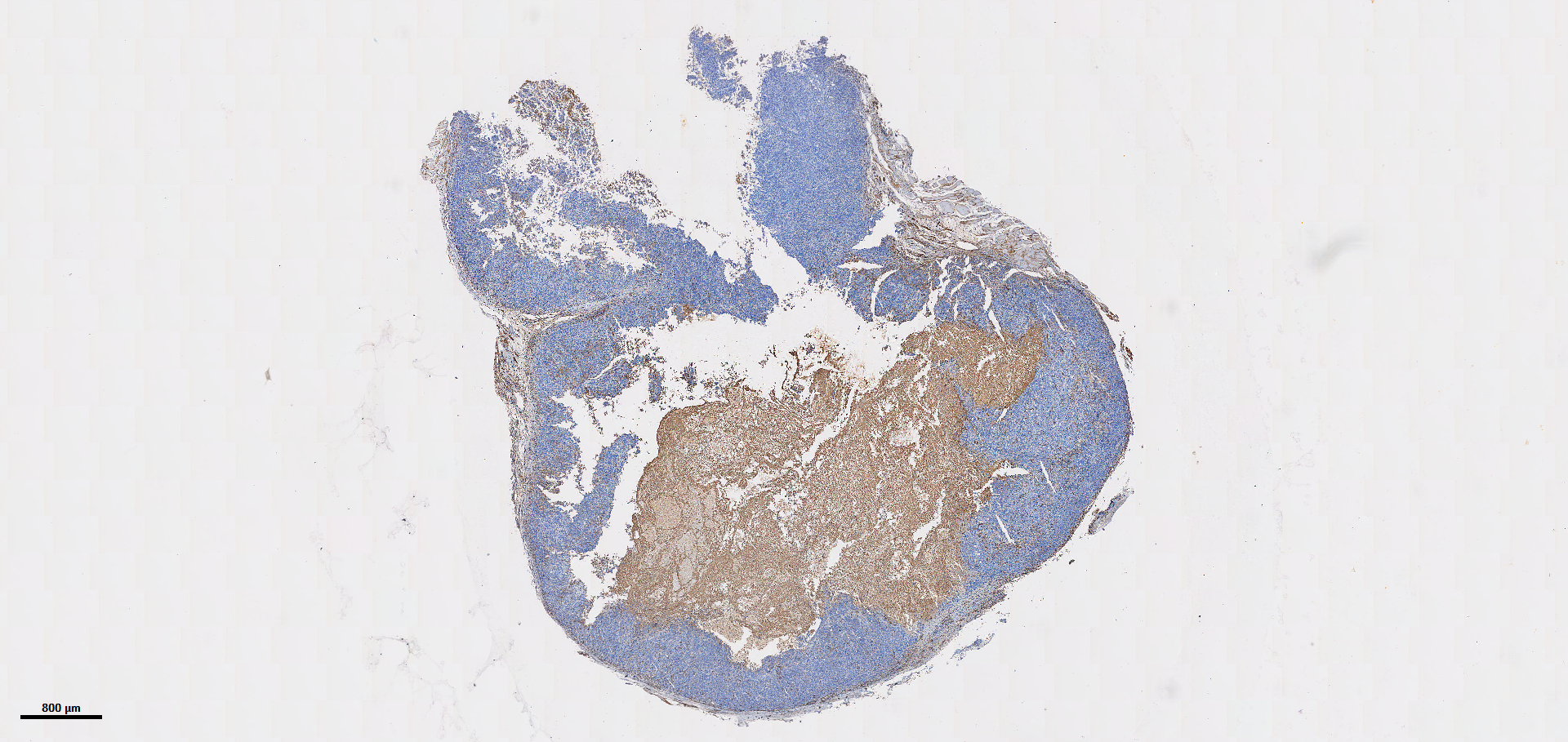

Supplement: Supplementary file 4 — Source data Fig. 2 [file 44321_2026_424_MOESM4_ESM.zip › Figure 2 Source Data/Figure 2D/CD8/ONEOvac 1.tif]

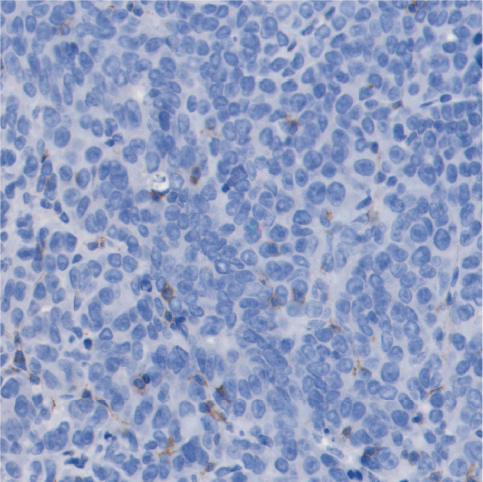

Supplement: Supplementary file 4 — Source data Fig. 2 [file 44321_2026_424_MOESM4_ESM.zip › Figure 2 Source Data/Figure 2D/CD8/OMVs 2.png]

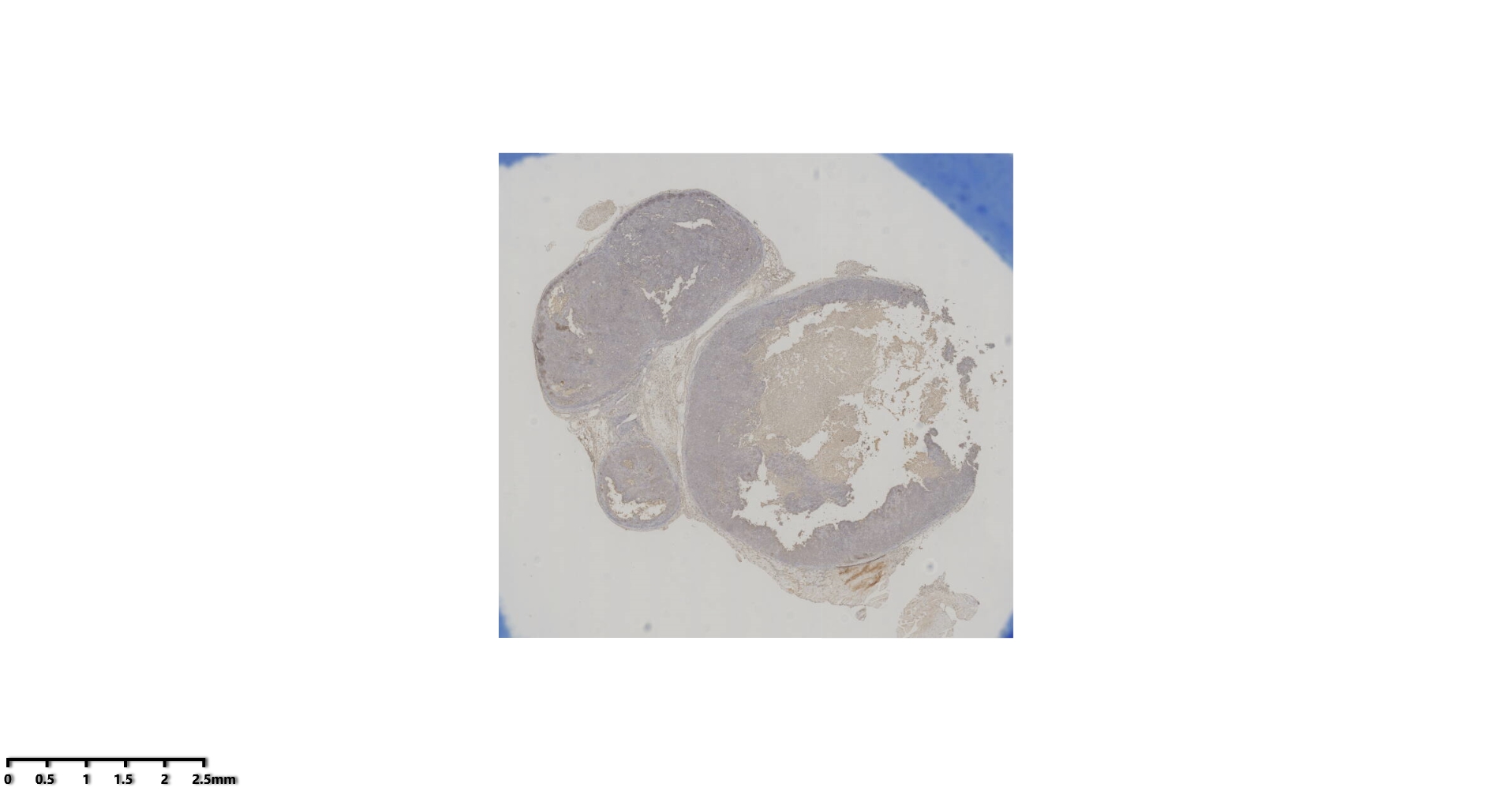

Supplement: Supplementary file 4 — Source data Fig. 2 [file 44321_2026_424_MOESM4_ESM.zip › Figure 2 Source Data/Figure 2D/NK/OMICBvac 1.jpg]

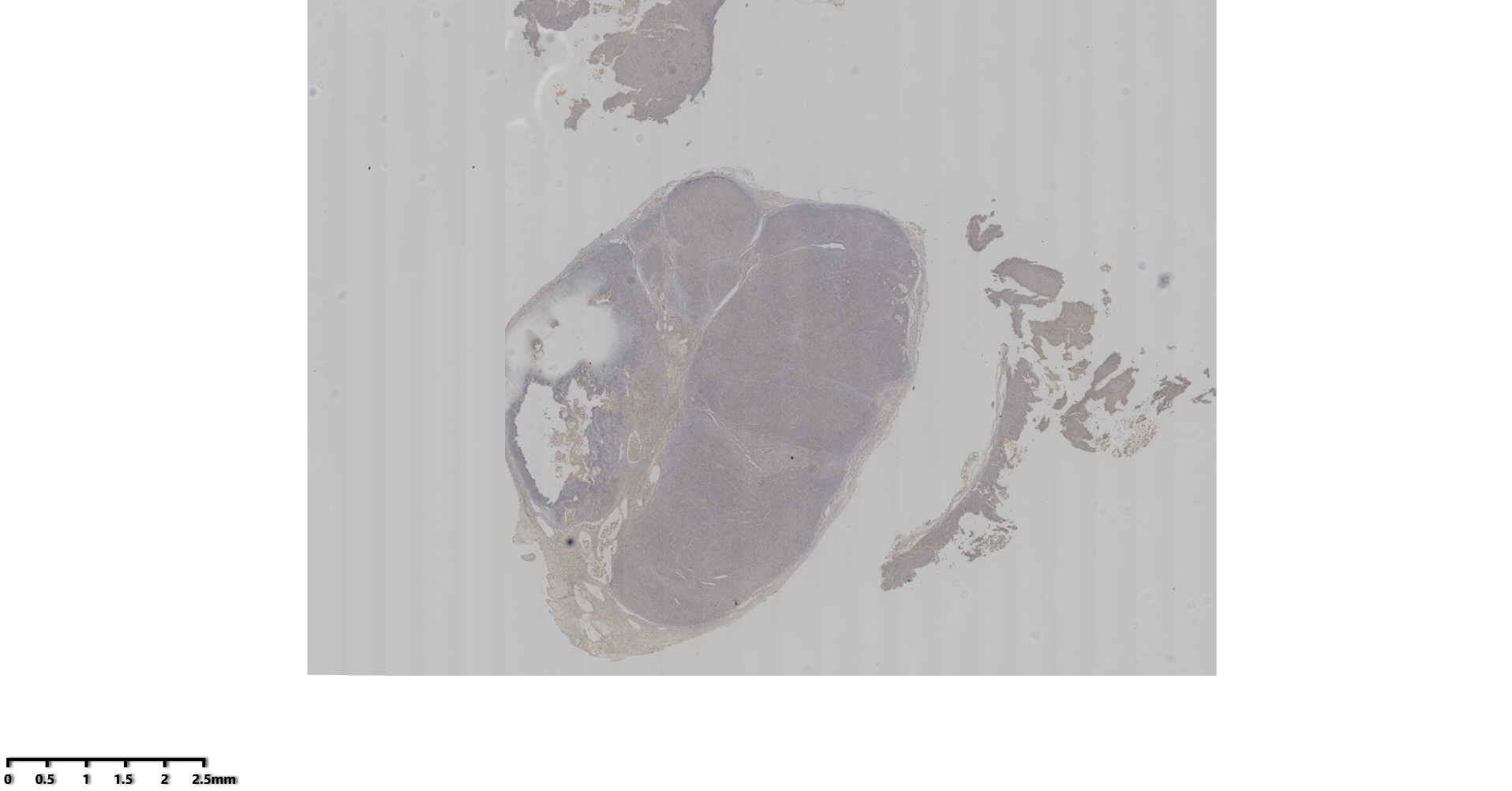

Supplement: Supplementary file 4 — Source data Fig. 2 [file 44321_2026_424_MOESM4_ESM.zip › Figure 2 Source Data/Figure 2D/NK/CTRL 1.jpg]

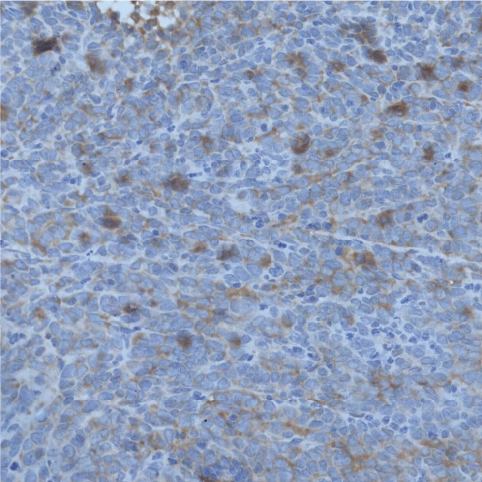

Supplement: Supplementary file 4 — Source data Fig. 2 [file 44321_2026_424_MOESM4_ESM.zip › Figure 2 Source Data/Figure 2D/NK/OMICBvac 2.png]

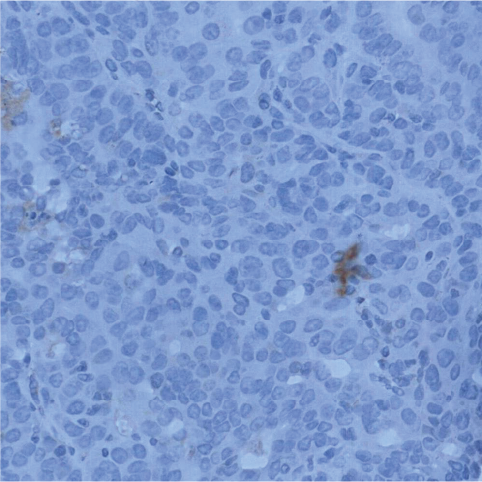

Supplement: Supplementary file 4 — Source data Fig. 2 [file 44321_2026_424_MOESM4_ESM.zip › Figure 2 Source Data/Figure 2D/NK/CTRL 2.png]

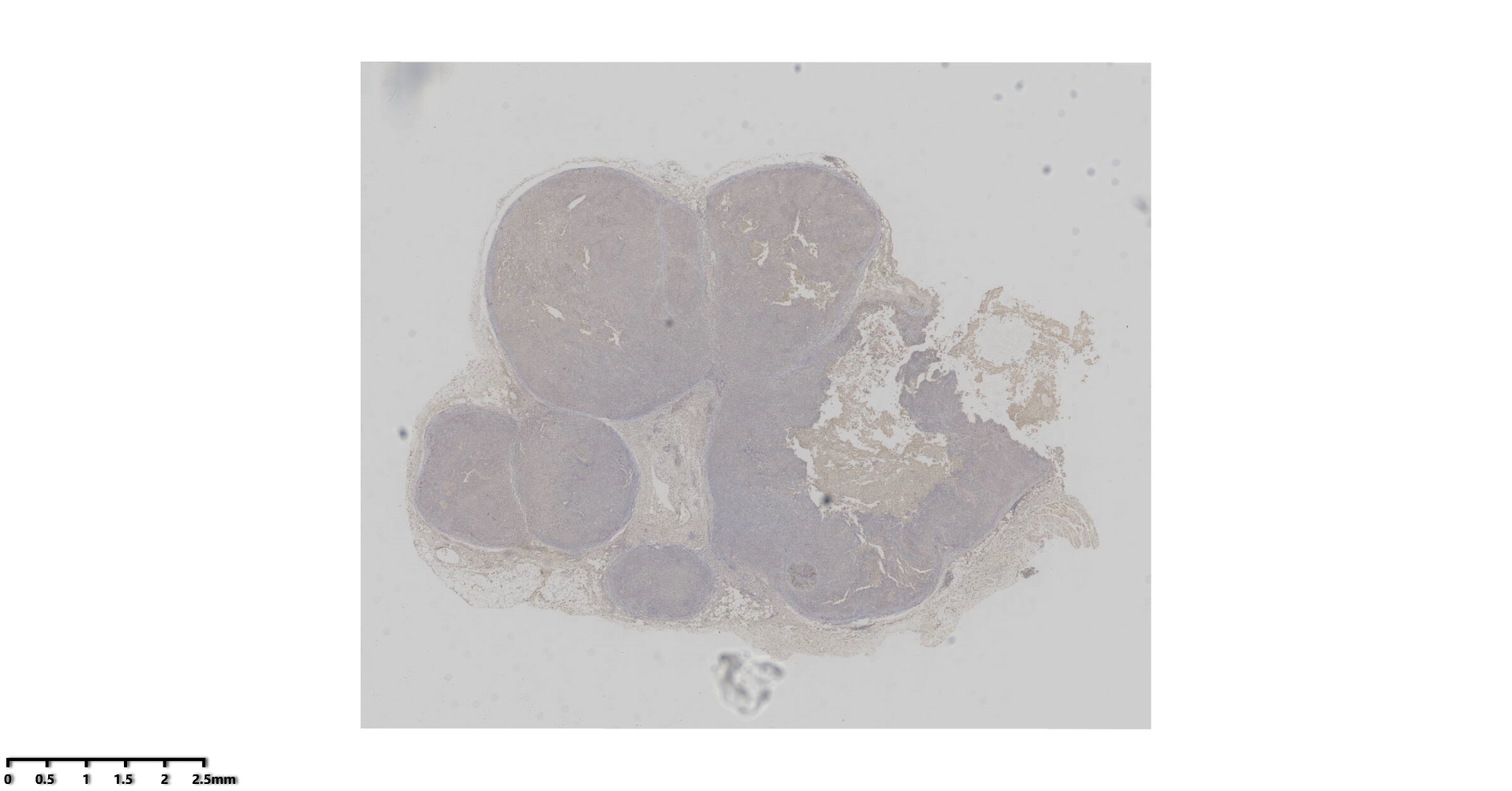

Supplement: Supplementary file 4 — Source data Fig. 2 [file 44321_2026_424_MOESM4_ESM.zip › Figure 2 Source Data/Figure 2D/NK/ONEOvac 1.jpg]

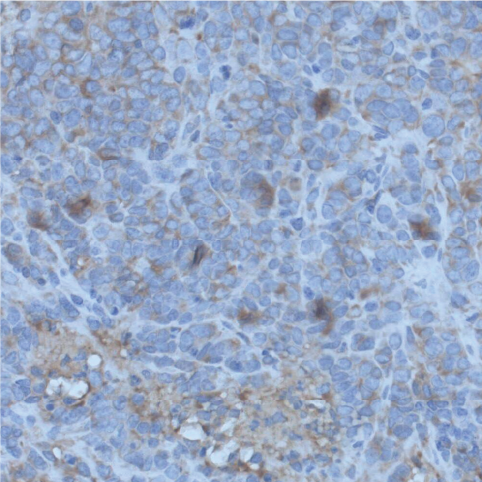

Supplement: Supplementary file 4 — Source data Fig. 2 [file 44321_2026_424_MOESM4_ESM.zip › Figure 2 Source Data/Figure 2D/NK/ONEOvac 2.png]

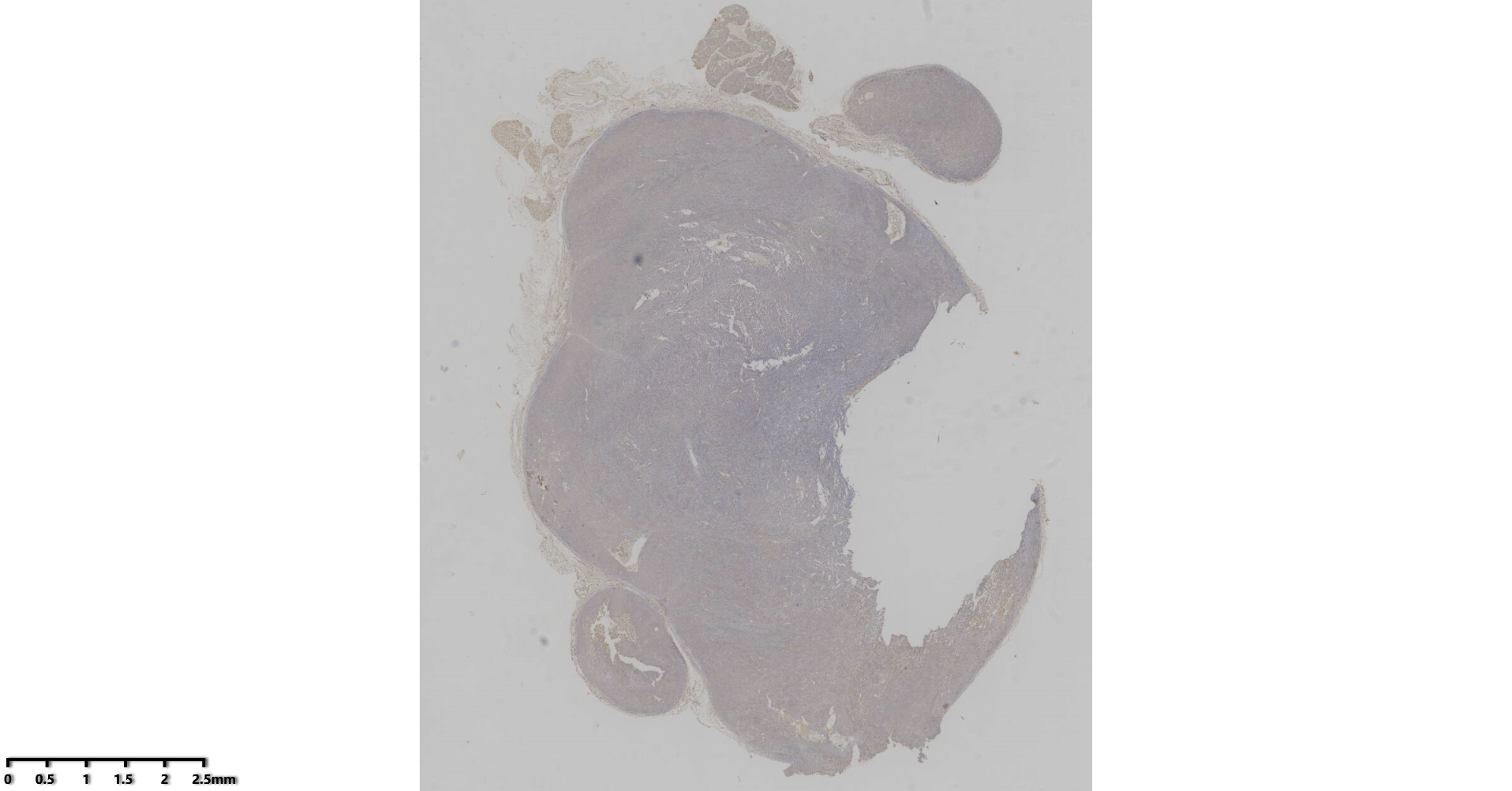

Supplement: Supplementary file 4 — Source data Fig. 2 [file 44321_2026_424_MOESM4_ESM.zip › Figure 2 Source Data/Figure 2D/NK/OMVs 1.jpg]

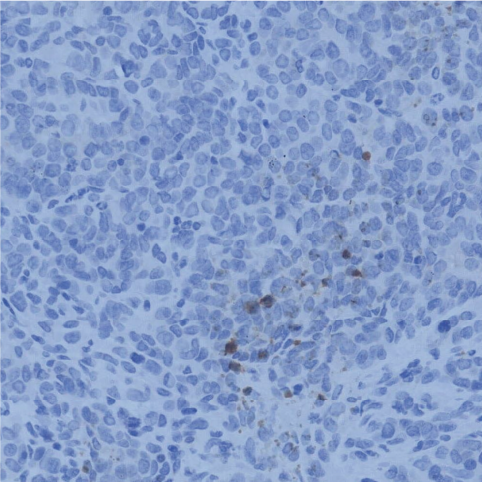

Supplement: Supplementary file 4 — Source data Fig. 2 [file 44321_2026_424_MOESM4_ESM.zip › Figure 2 Source Data/Figure 2D/NK/OMVs 2.png]

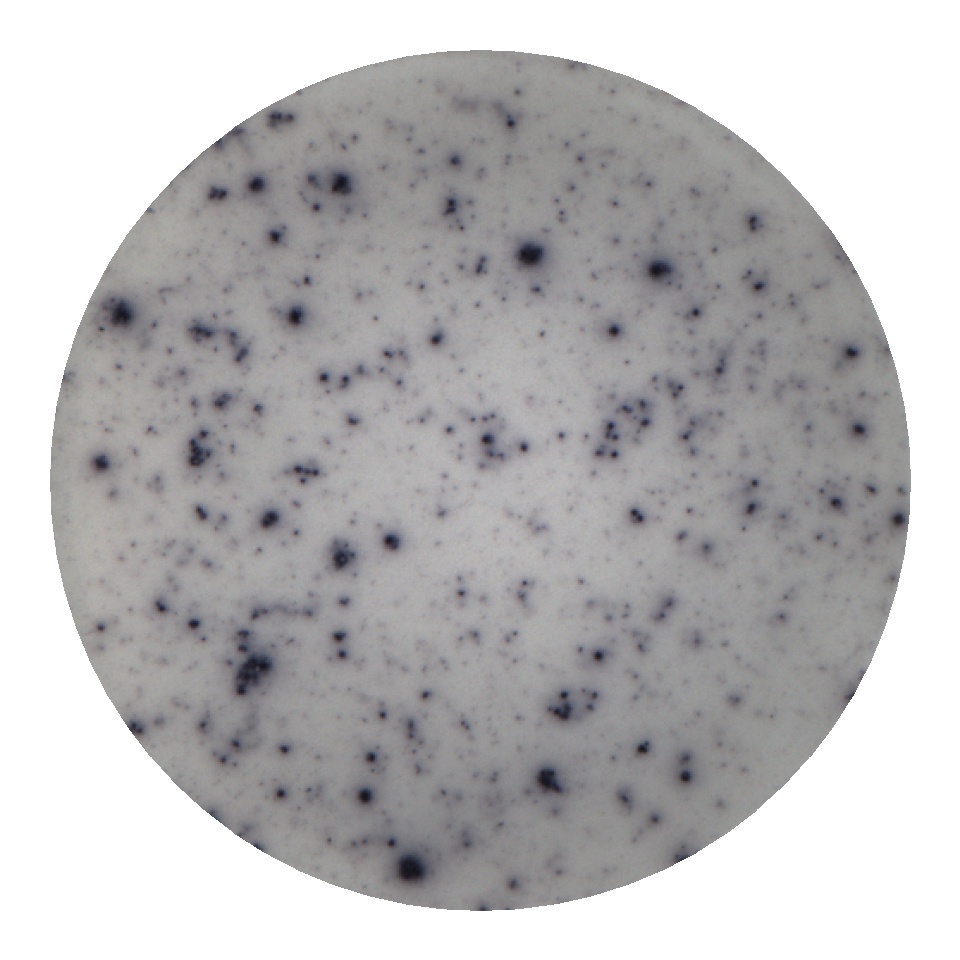

Supplement: Supplementary file 5 — Source data Fig. 3 [file 44321_2026_424_MOESM5_ESM.zip › Figure 3 Source Data/Figure 3A/Fusion injection 2.jpg]

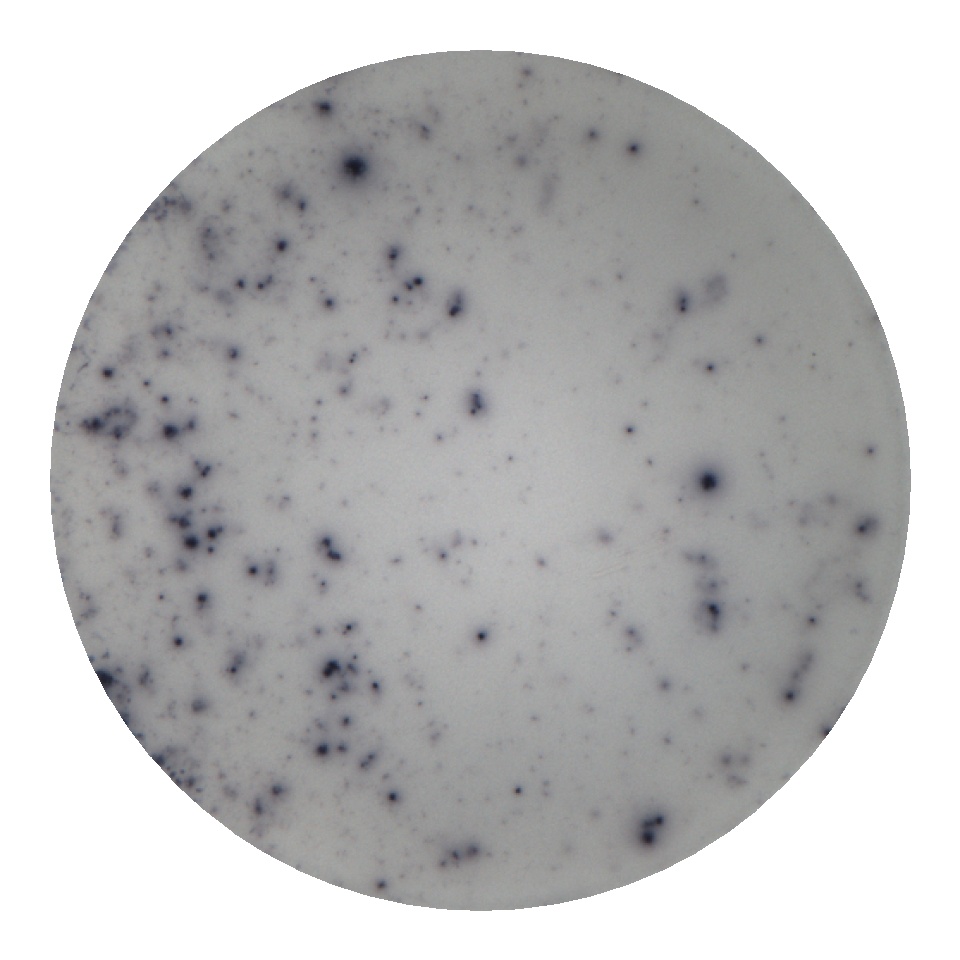

Supplement: Supplementary file 5 — Source data Fig. 3 [file 44321_2026_424_MOESM5_ESM.zip › Figure 3 Source Data/Figure 3A/Fusion injection 3.jpg]

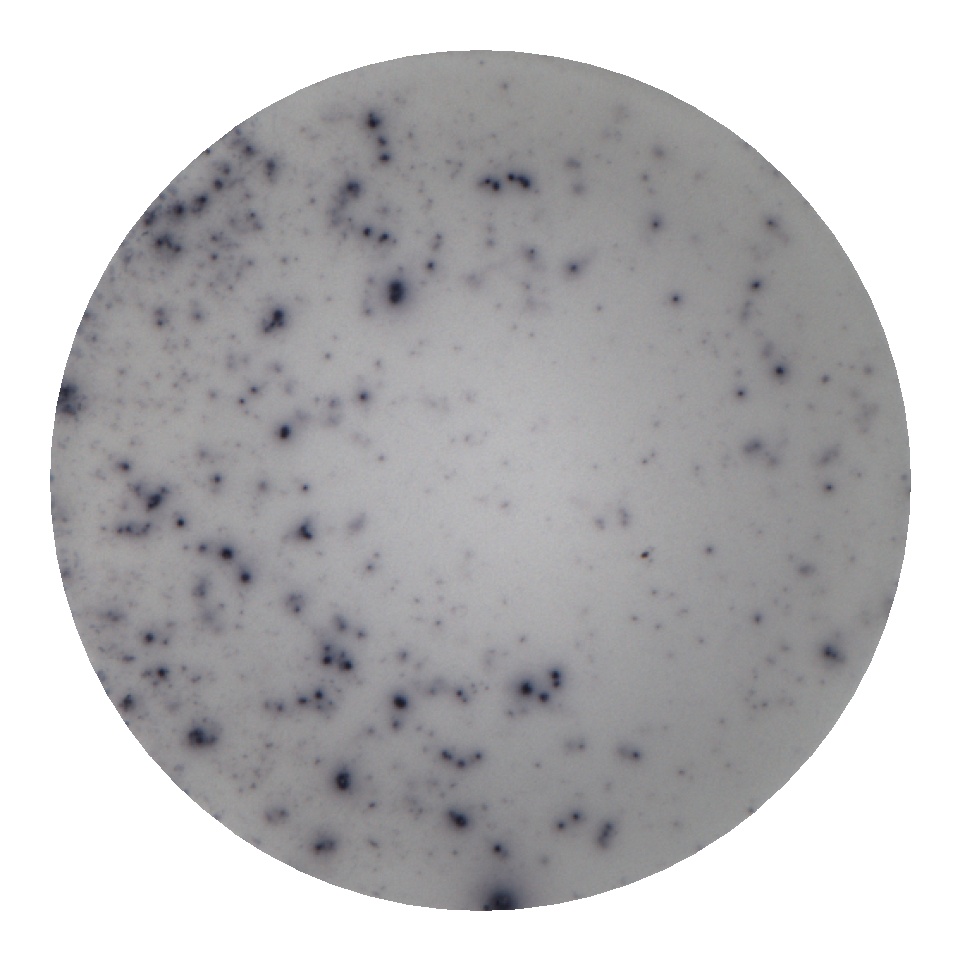

Supplement: Supplementary file 5 — Source data Fig. 3 [file 44321_2026_424_MOESM5_ESM.zip › Figure 3 Source Data/Figure 3A/Fusion injection 1.jpg]

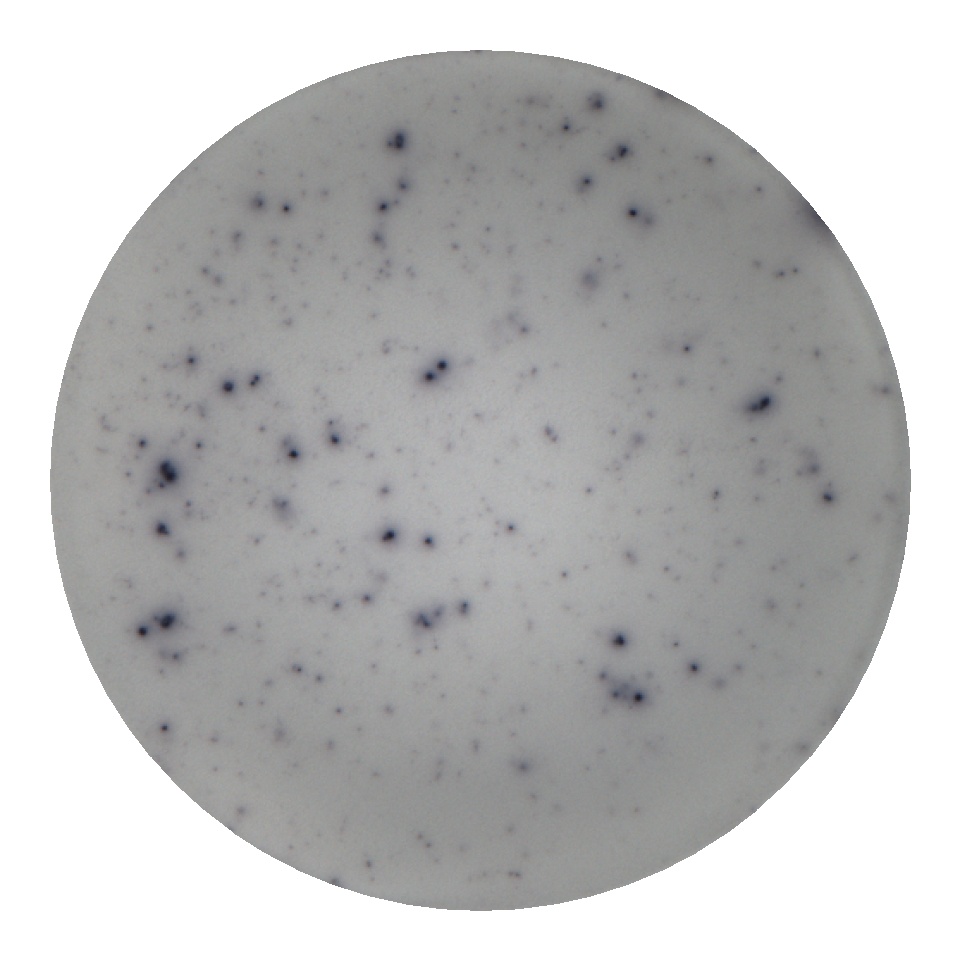

Supplement: Supplementary file 5 — Source data Fig. 3 [file 44321_2026_424_MOESM5_ESM.zip › Figure 3 Source Data/Figure 3A/CTRL 1.jpg]

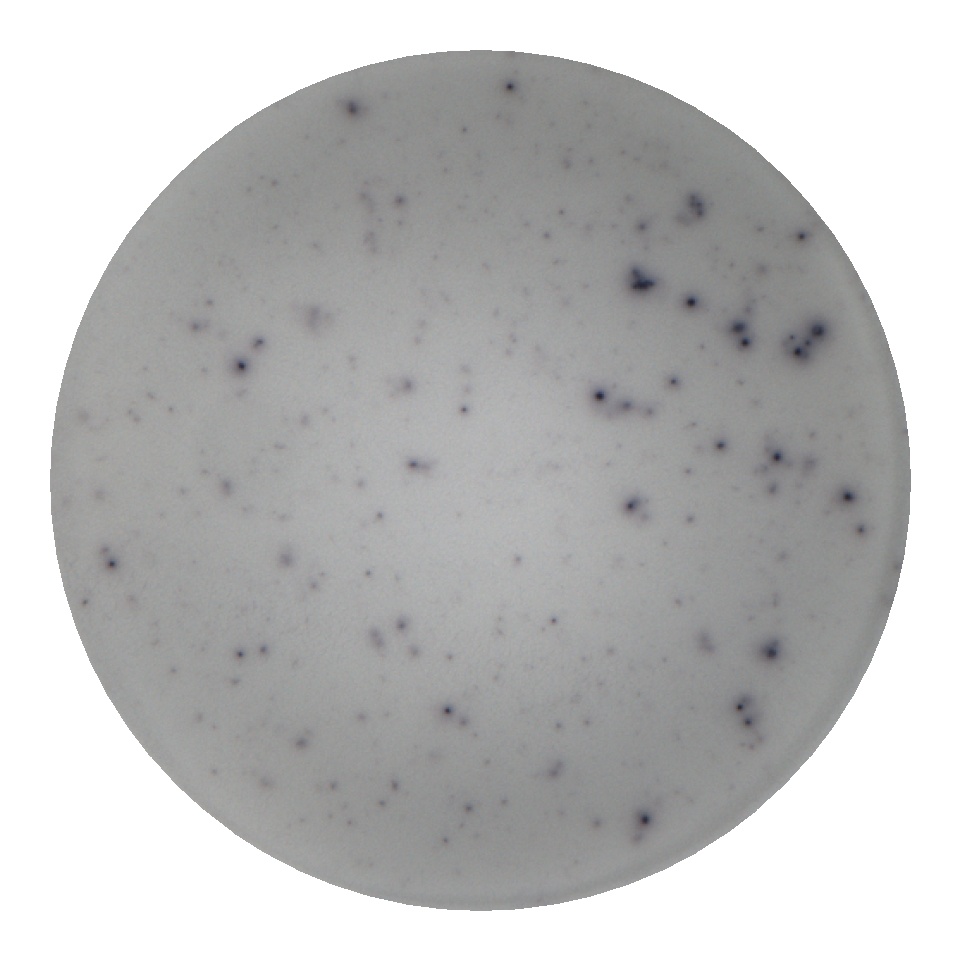

Supplement: Supplementary file 5 — Source data Fig. 3 [file 44321_2026_424_MOESM5_ESM.zip › Figure 3 Source Data/Figure 3A/CTRL 3.jpg]

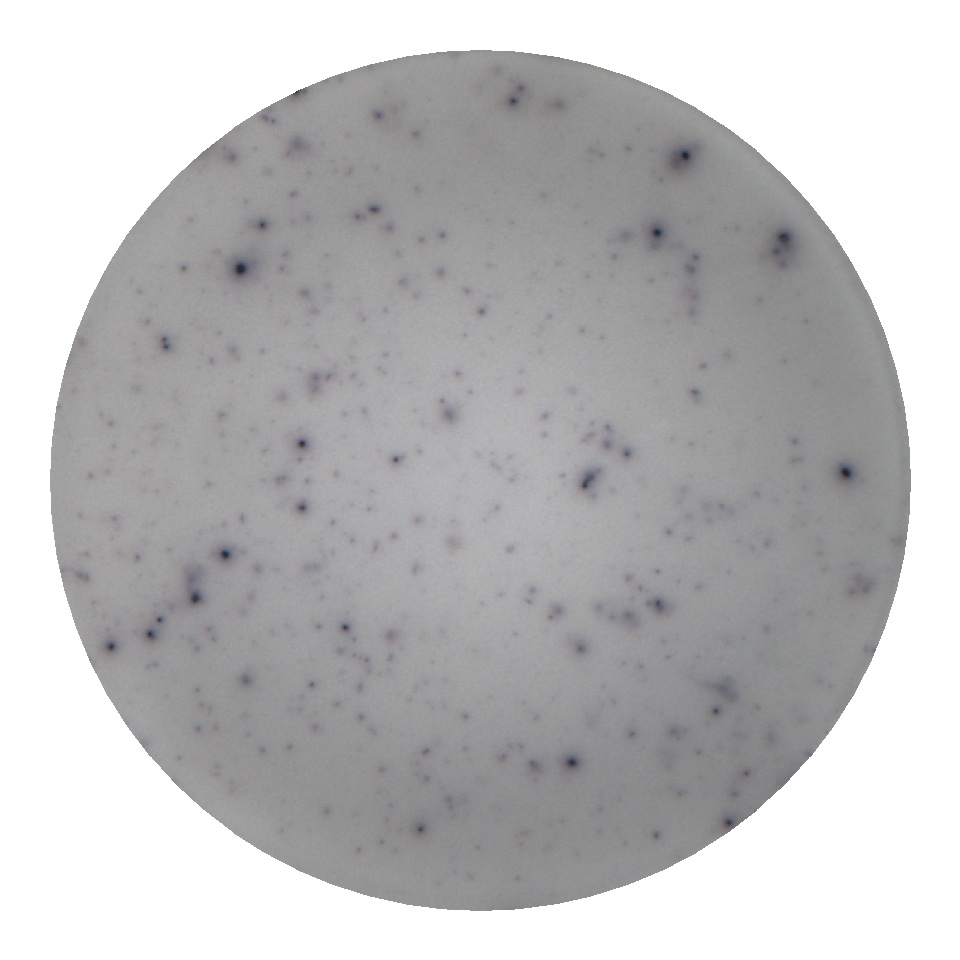

Supplement: Supplementary file 5 — Source data Fig. 3 [file 44321_2026_424_MOESM5_ESM.zip › Figure 3 Source Data/Figure 3A/CTRL 2.jpg]

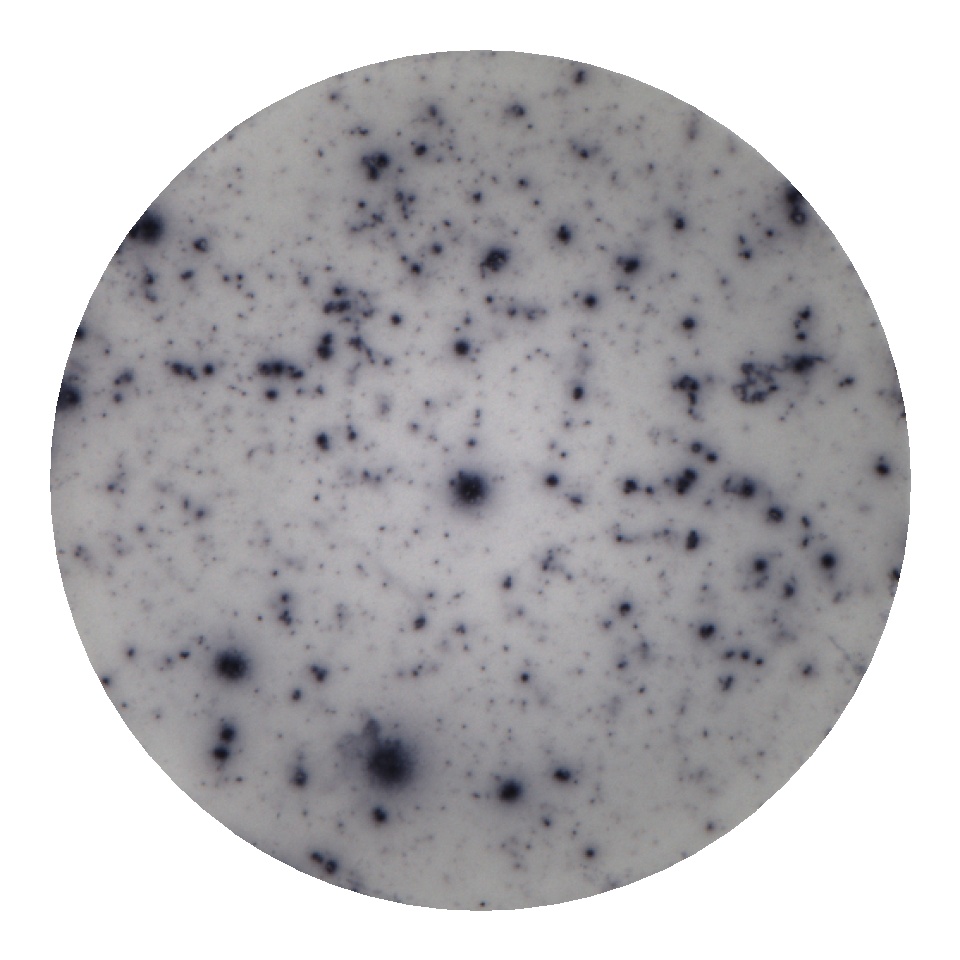

Supplement: Supplementary file 5 — Source data Fig. 3 [file 44321_2026_424_MOESM5_ESM.zip › Figure 3 Source Data/Figure 3A/Bilateral injection 1.jpg]

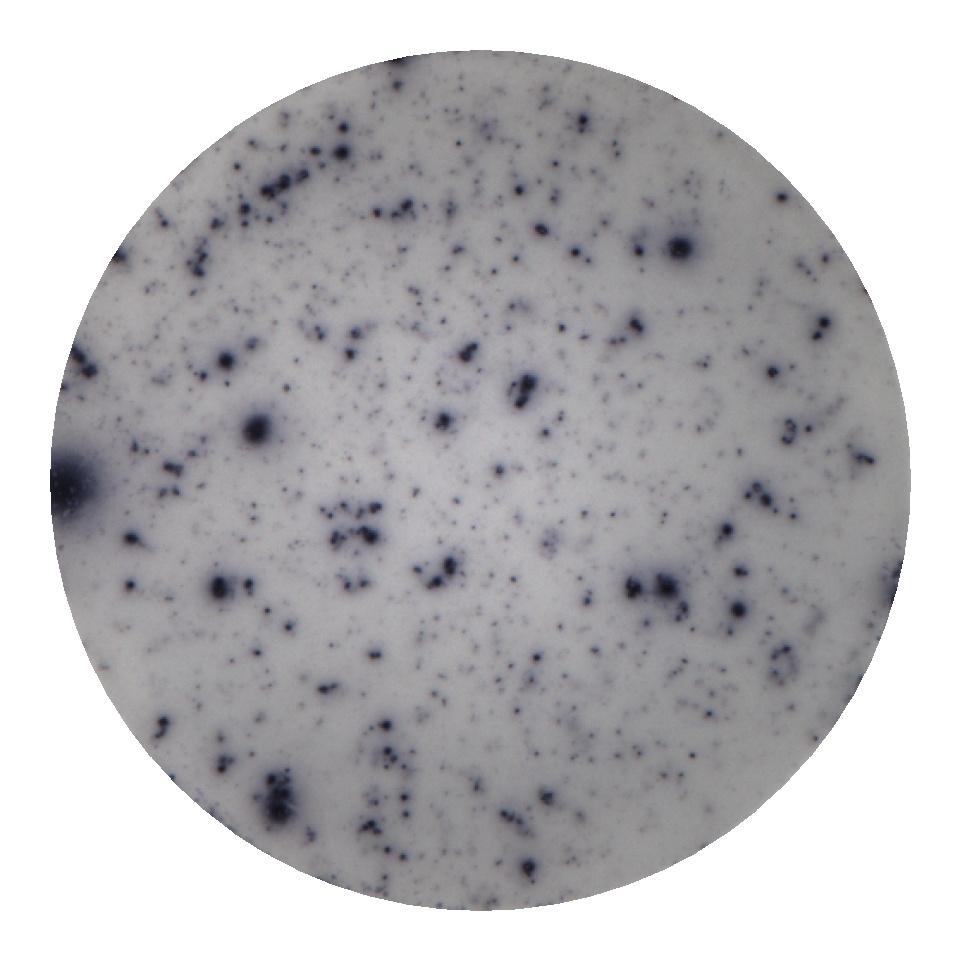

Supplement: Supplementary file 5 — Source data Fig. 3 [file 44321_2026_424_MOESM5_ESM.zip › Figure 3 Source Data/Figure 3A/Bilateral injection 3.jpg]

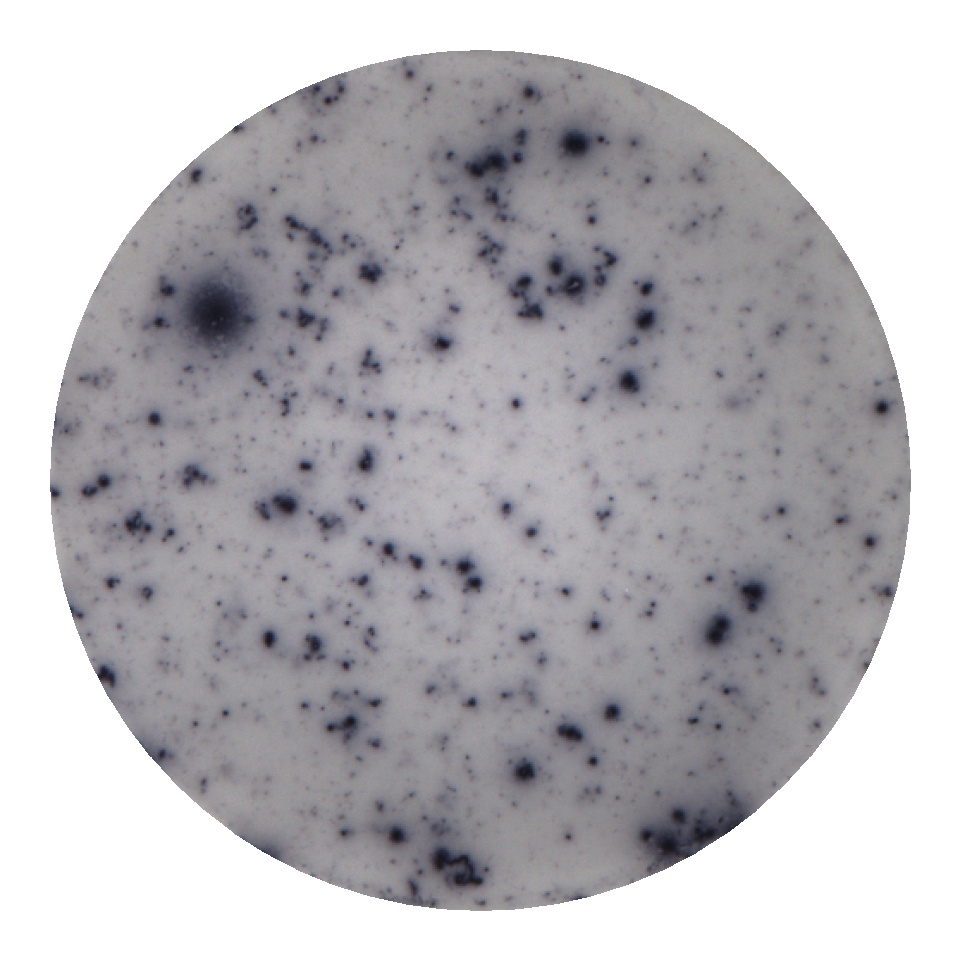

Supplement: Supplementary file 5 — Source data Fig. 3 [file 44321_2026_424_MOESM5_ESM.zip › Figure 3 Source Data/Figure 3A/Bilateral injection 2.jpg]

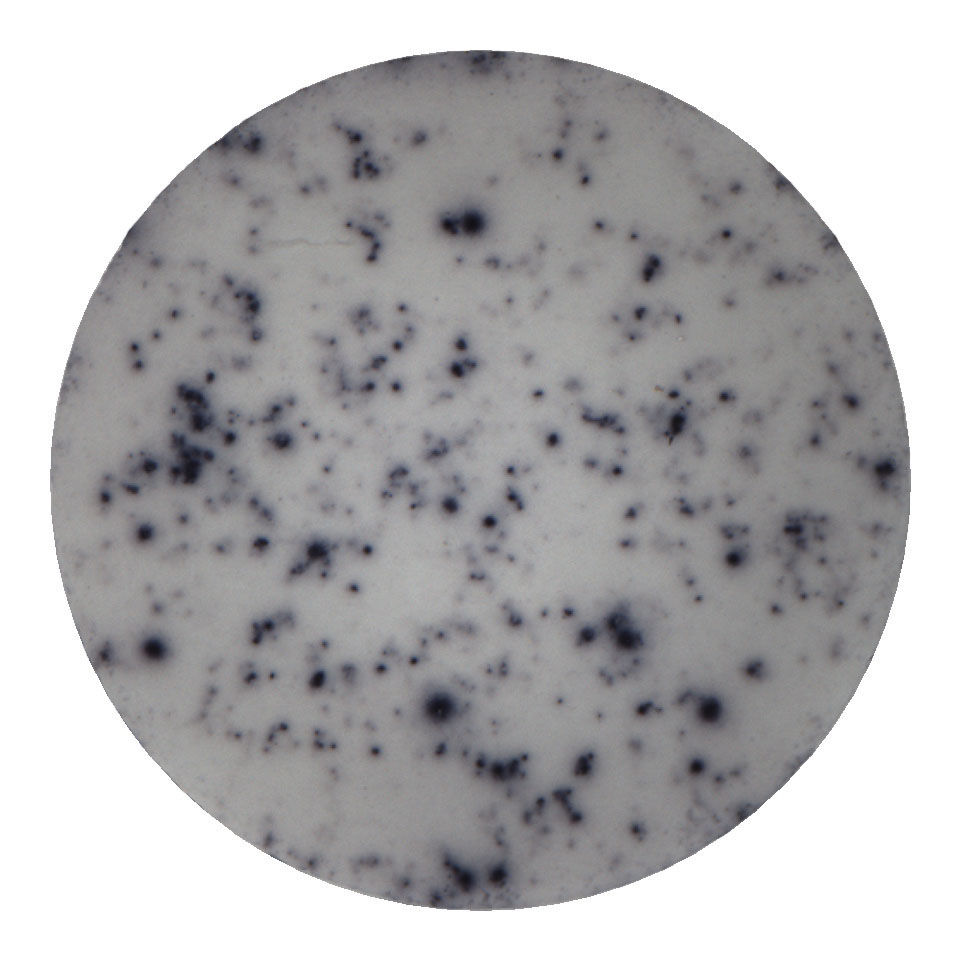

Supplement: Supplementary file 5 — Source data Fig. 3 [file 44321_2026_424_MOESM5_ESM.zip › Figure 3 Source Data/Figure 3A/Unilateral injection 1.jpg]

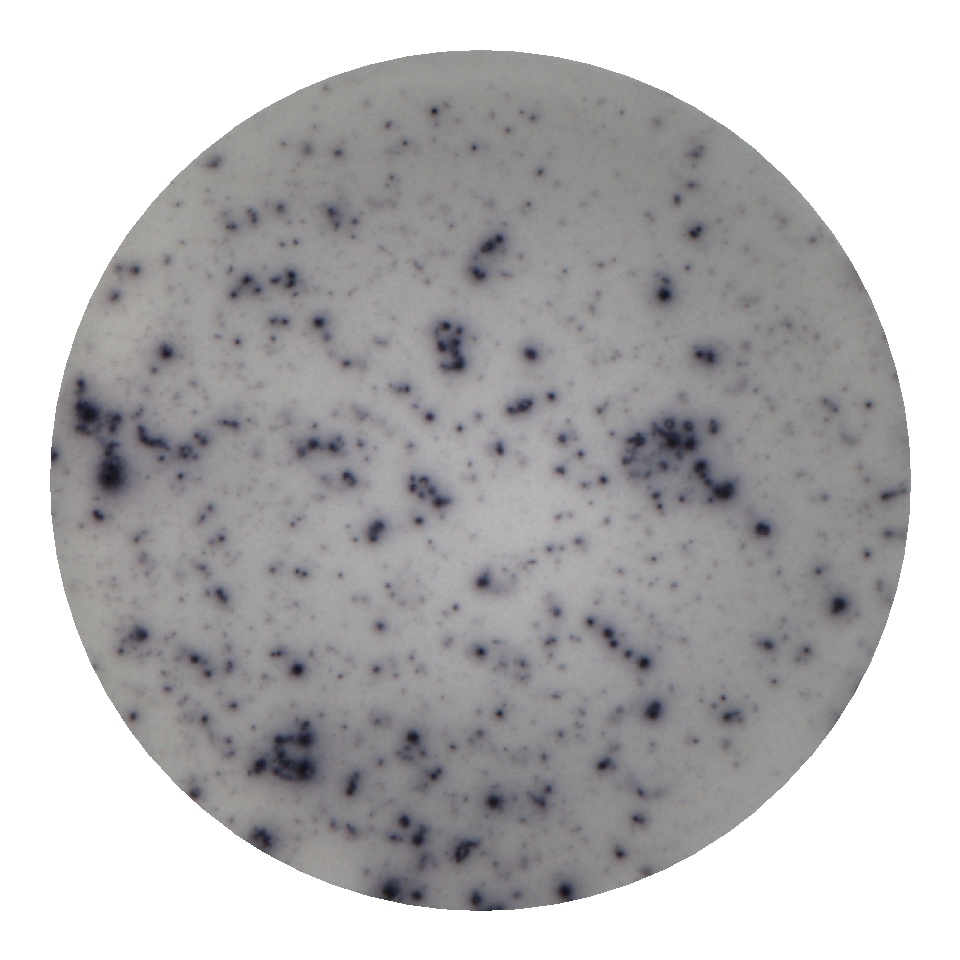

Supplement: Supplementary file 5 — Source data Fig. 3 [file 44321_2026_424_MOESM5_ESM.zip › Figure 3 Source Data/Figure 3A/Unilateral injection 3.jpg]

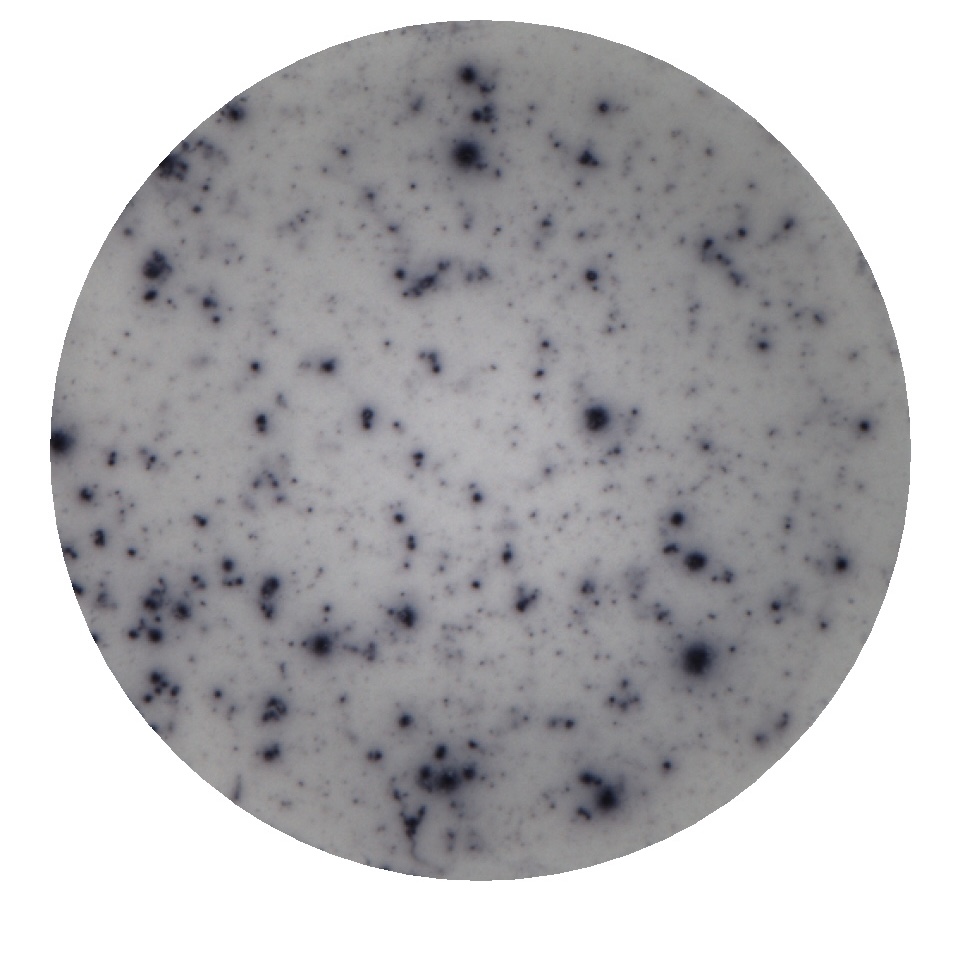

Supplement: Supplementary file 5 — Source data Fig. 3 [file 44321_2026_424_MOESM5_ESM.zip › Figure 3 Source Data/Figure 3A/Unilateral injection 2.jpg]

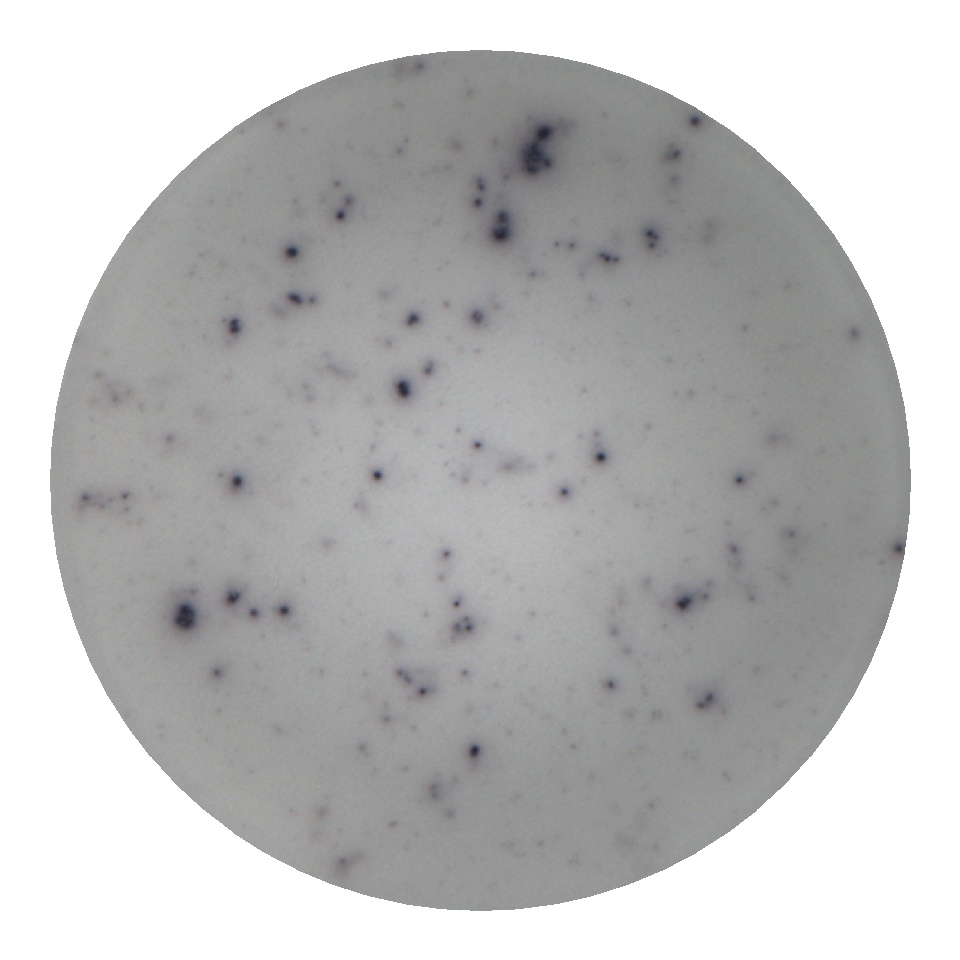

Supplement: Supplementary file 5 — Source data Fig. 3 [file 44321_2026_424_MOESM5_ESM.zip › Figure 3 Source Data/Figure 3A/OMVs 1.jpg]

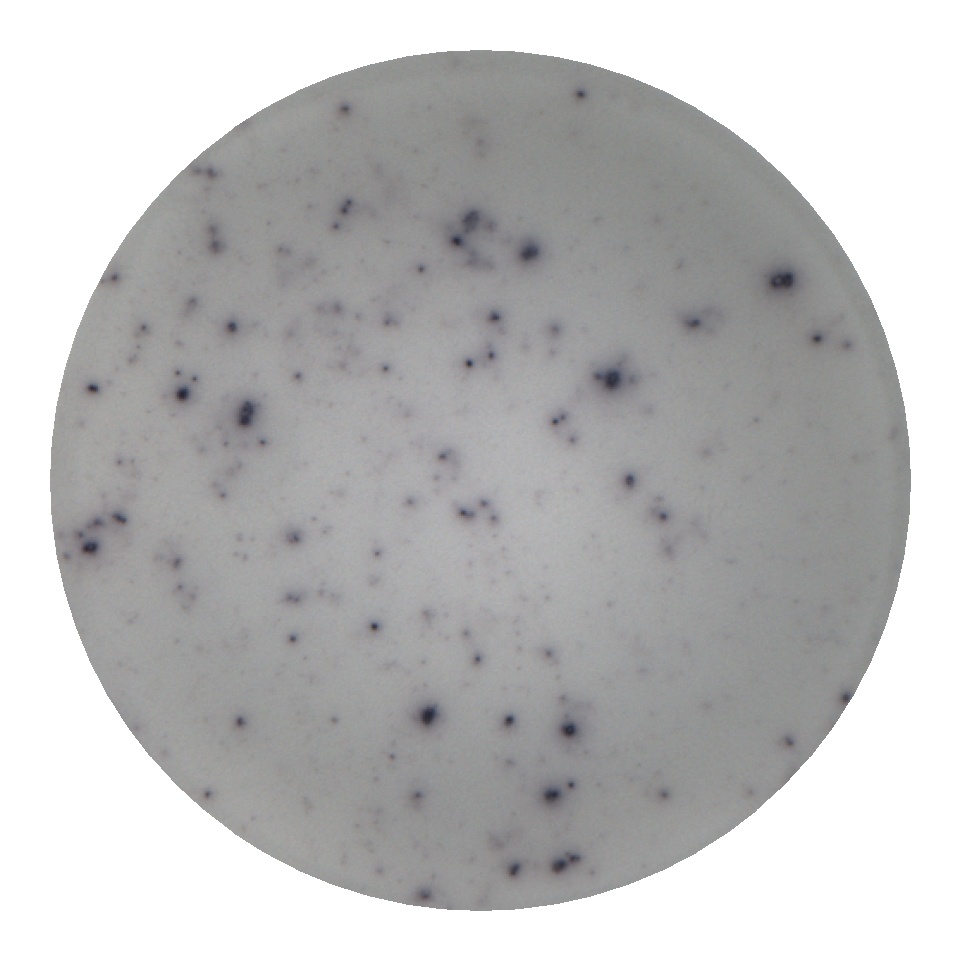

Supplement: Supplementary file 5 — Source data Fig. 3 [file 44321_2026_424_MOESM5_ESM.zip › Figure 3 Source Data/Figure 3A/OMVs 2.jpg]

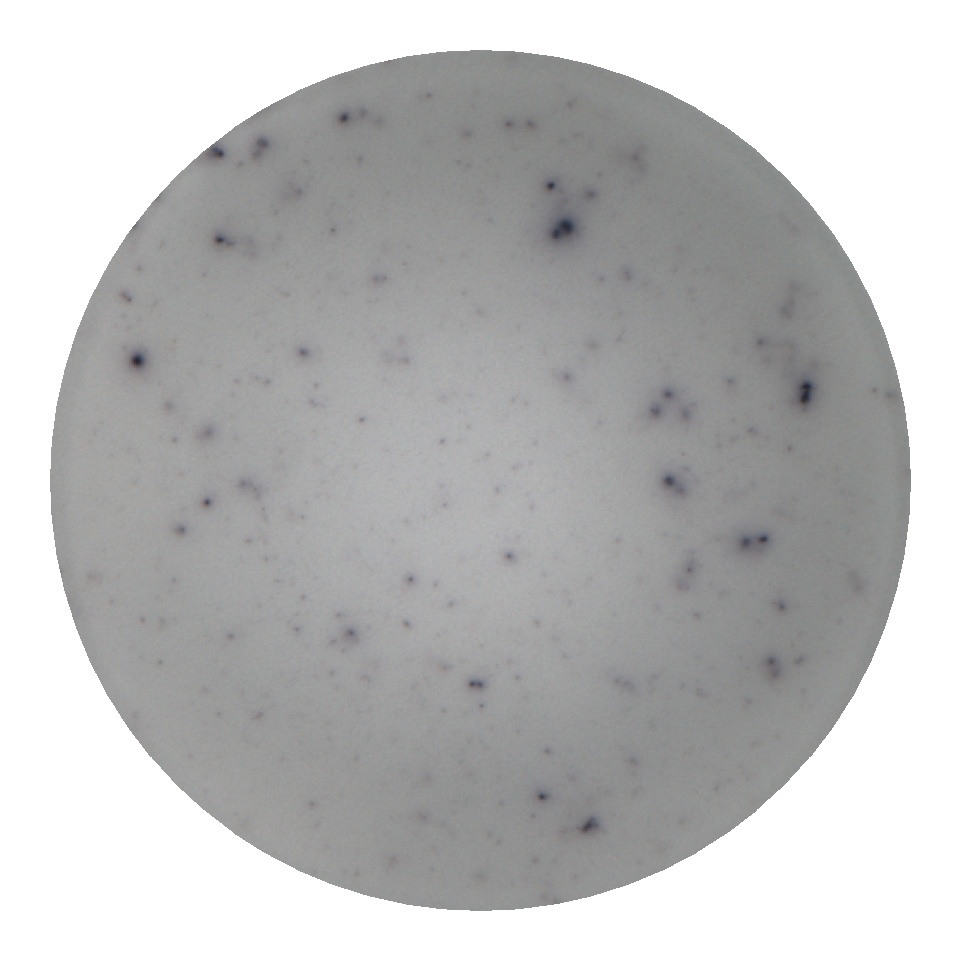

Supplement: Supplementary file 5 — Source data Fig. 3 [file 44321_2026_424_MOESM5_ESM.zip › Figure 3 Source Data/Figure 3A/OMVs 3.jpg]

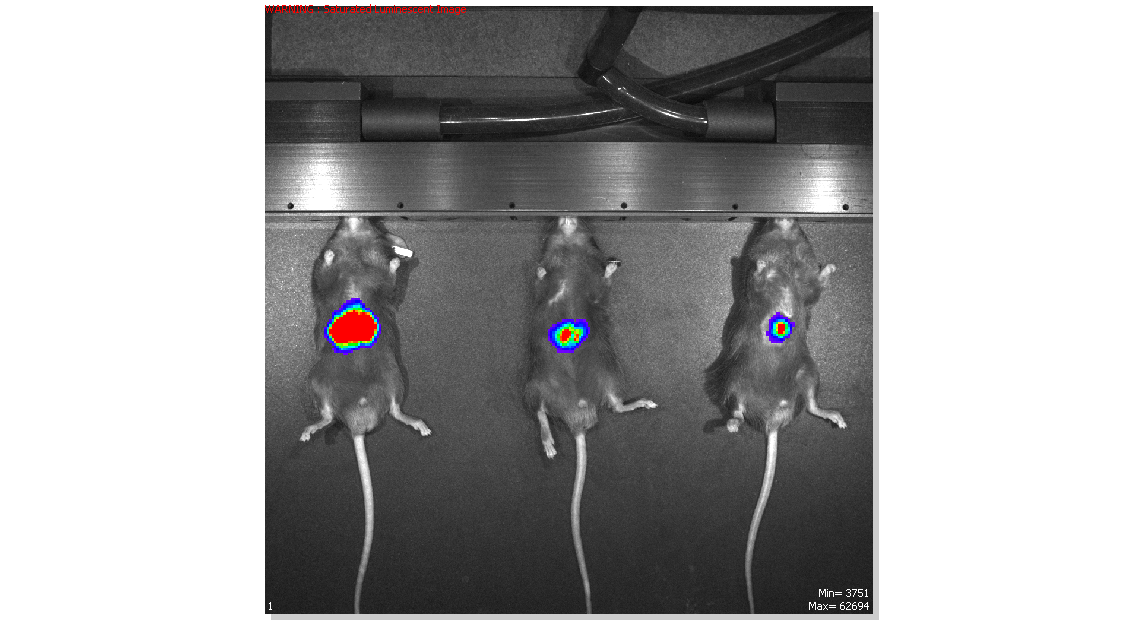

Supplement: Supplementary file 5 — Source data Fig. 3 [file 44321_2026_424_MOESM5_ESM.zip › Figure 3 Source Data/Figure 3D/Day 14/LFZ-PBS.tif]

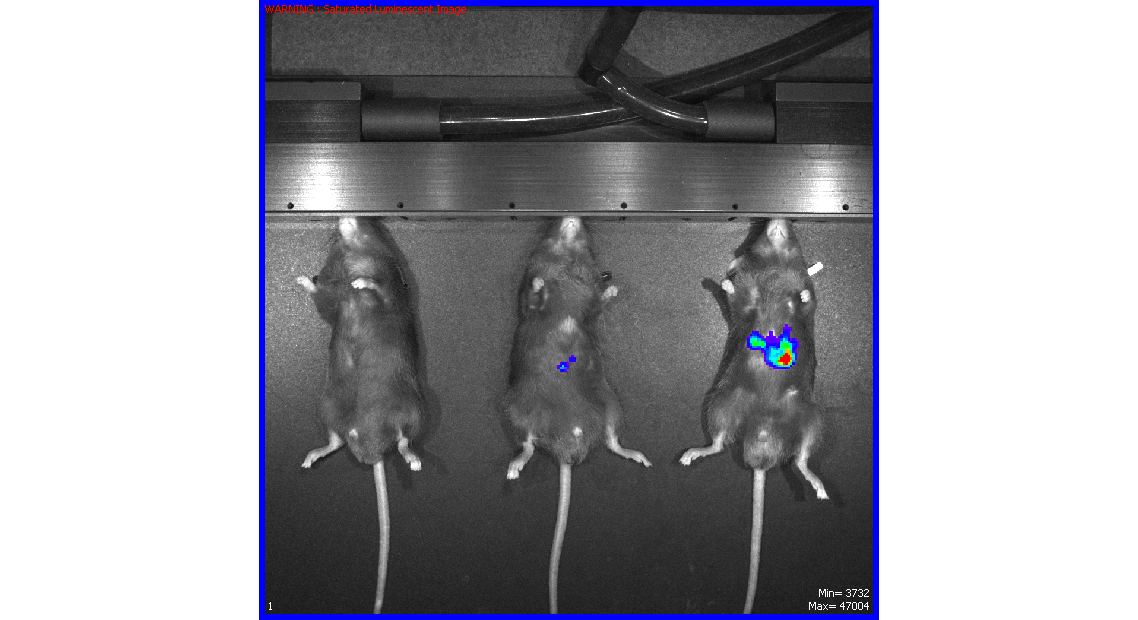

Supplement: Supplementary file 5 — Source data Fig. 3 [file 44321_2026_424_MOESM5_ESM.zip › Figure 3 Source Data/Figure 3D/Day 14/LFZ-ND-PD1.tif]

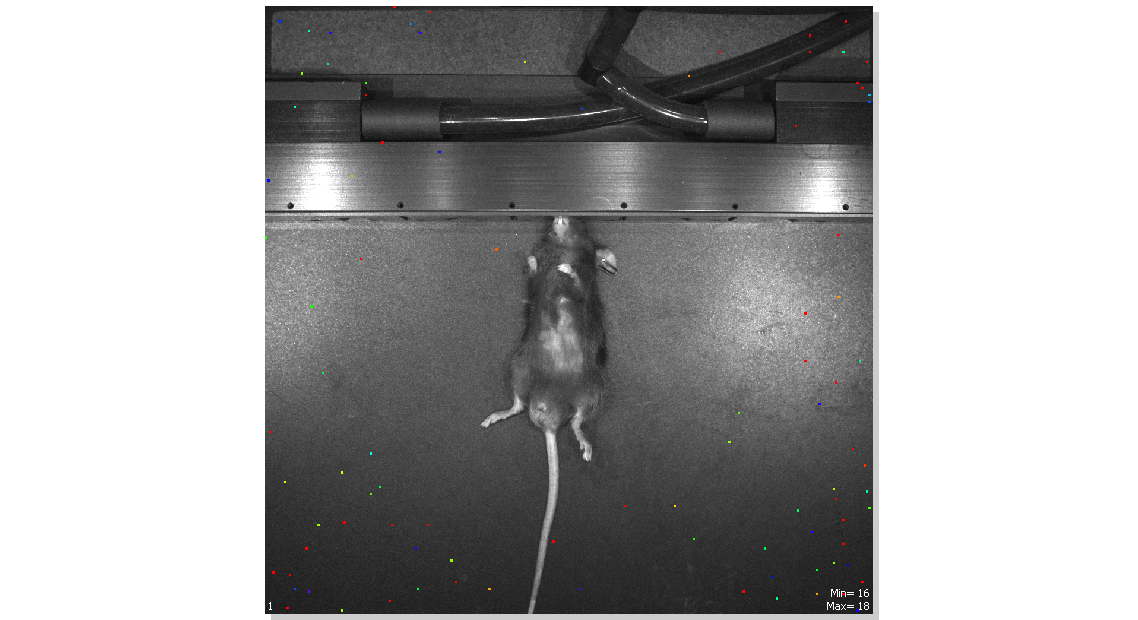

Supplement: Supplementary file 5 — Source data Fig. 3 [file 44321_2026_424_MOESM5_ESM.zip › Figure 3 Source Data/Figure 3D/Day 14/NF-FC-2-1.tif]

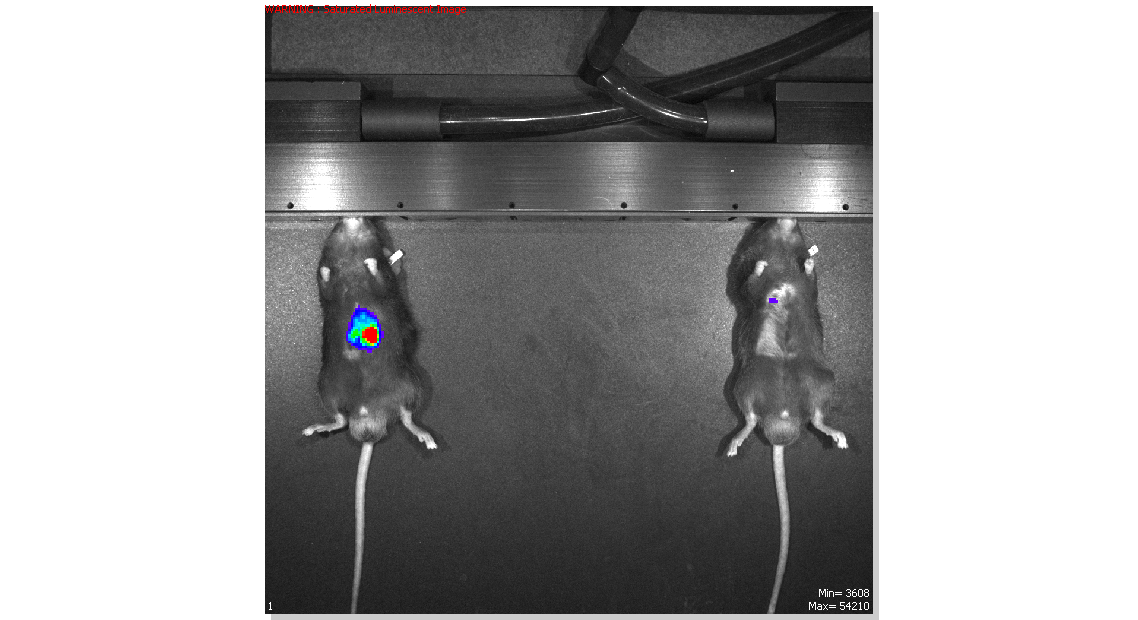

Supplement: Supplementary file 5 — Source data Fig. 3 [file 44321_2026_424_MOESM5_ESM.zip › Figure 3 Source Data/Figure 3D/Day 14/NFFC+MICB-2.tif]

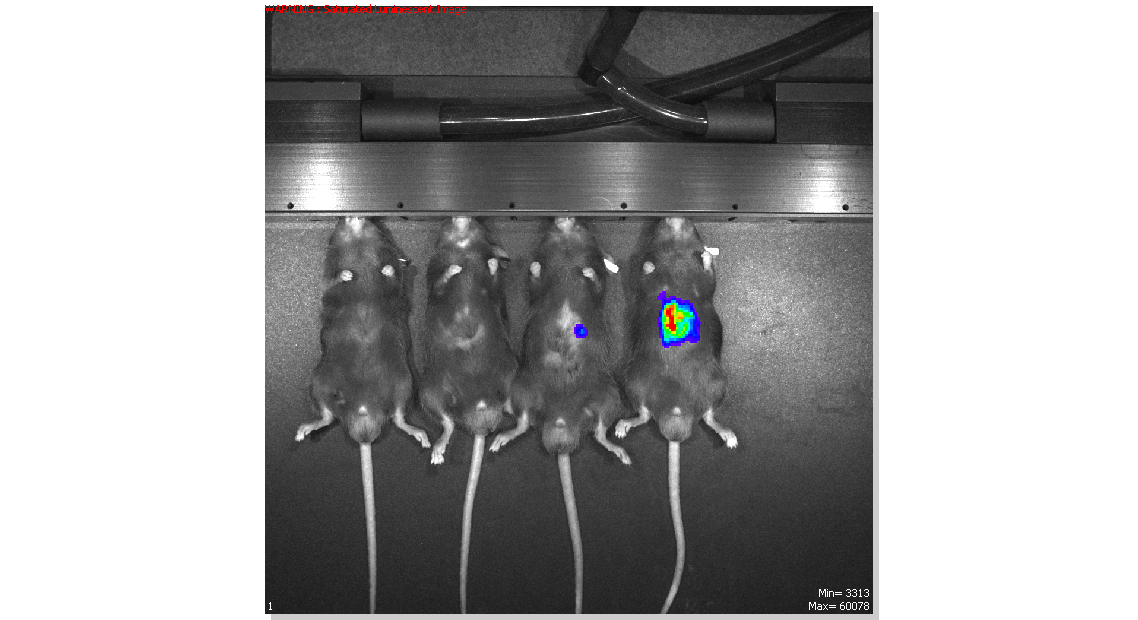

Supplement: Supplementary file 5 — Source data Fig. 3 [file 44321_2026_424_MOESM5_ESM.zip › Figure 3 Source Data/Figure 3D/Day 14/DUO-1.tif]

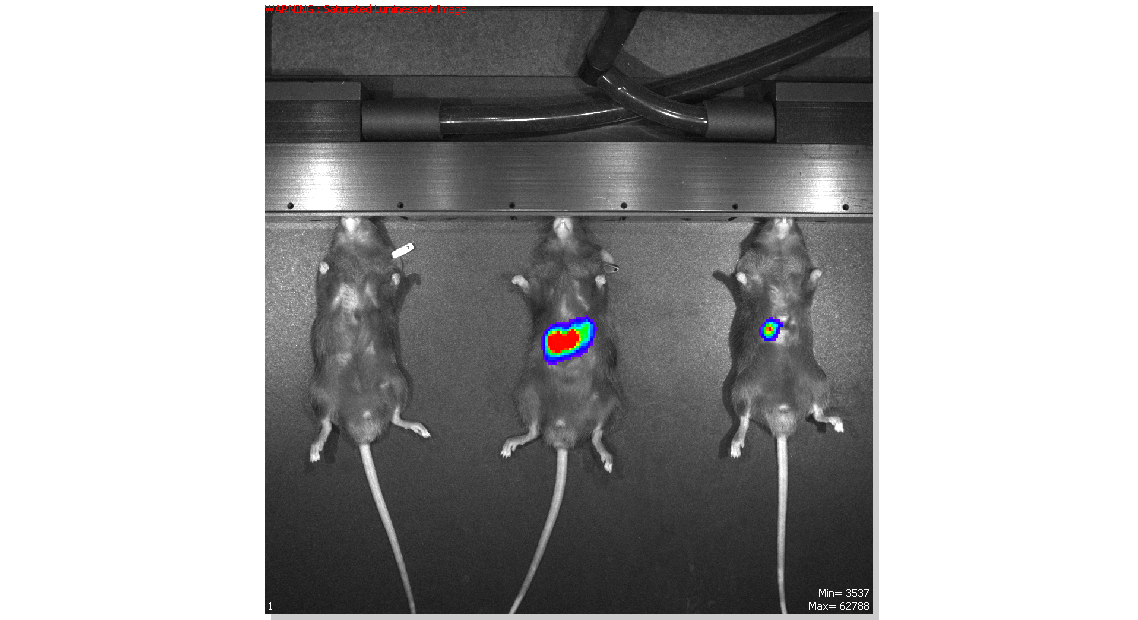

Supplement: Supplementary file 5 — Source data Fig. 3 [file 44321_2026_424_MOESM5_ESM.zip › Figure 3 Source Data/Figure 3D/Day 14/LFZ-PCSK9.tif]

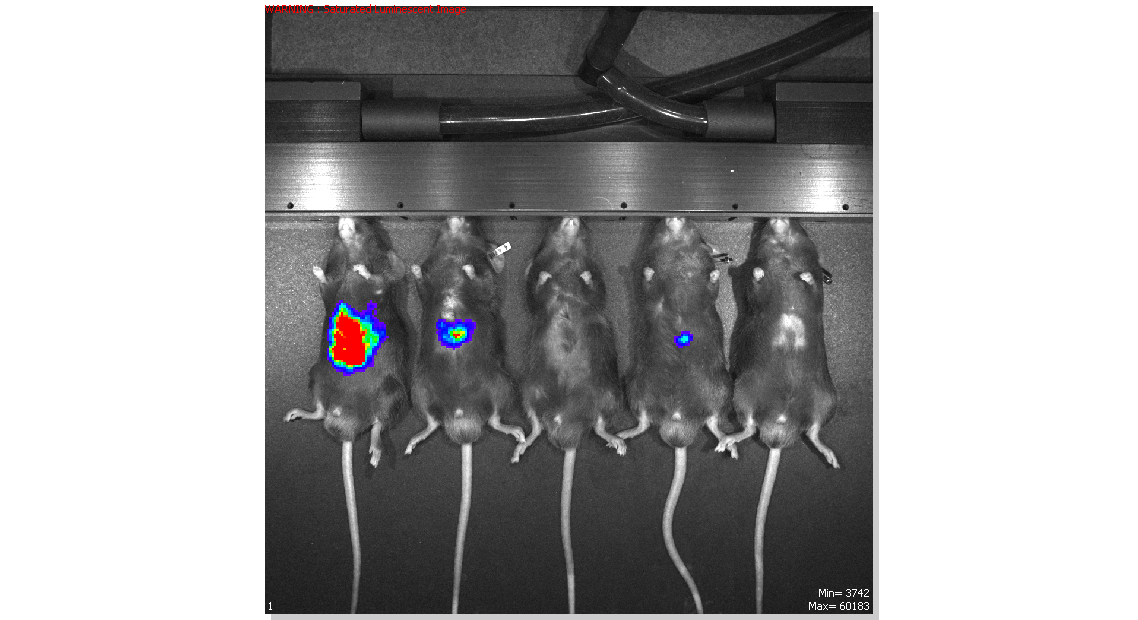

Supplement: Supplementary file 5 — Source data Fig. 3 [file 44321_2026_424_MOESM5_ESM.zip › Figure 3 Source Data/Figure 3D/Day 14/NFFC+MICB-1.tif]

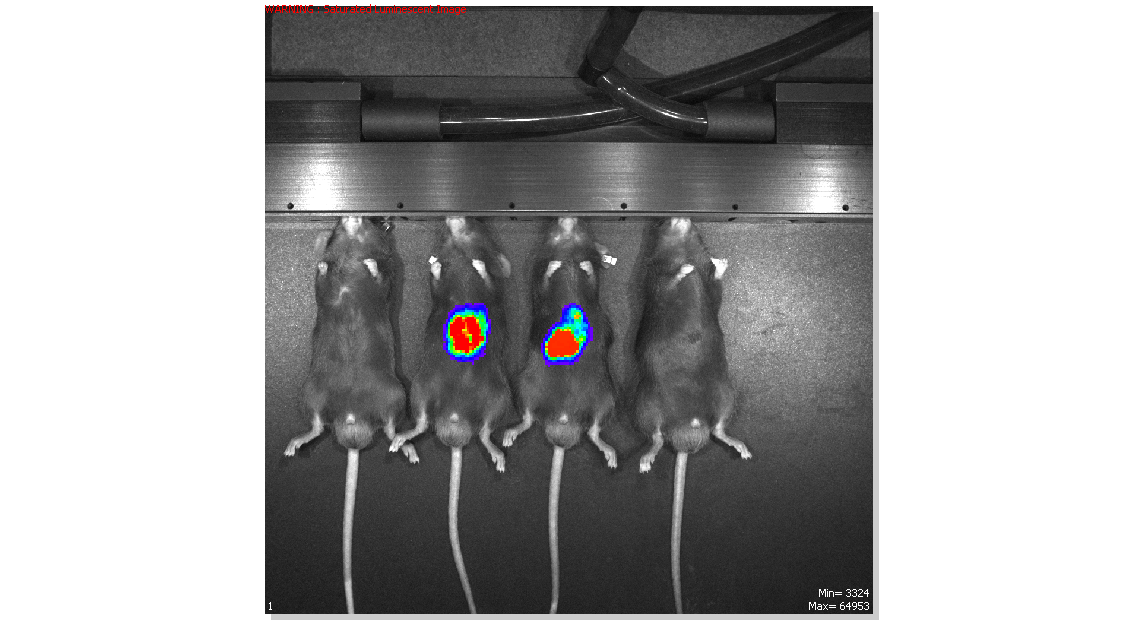

Supplement: Supplementary file 5 — Source data Fig. 3 [file 44321_2026_424_MOESM5_ESM.zip › Figure 3 Source Data/Figure 3D/Day 14/DUO-2.tif]

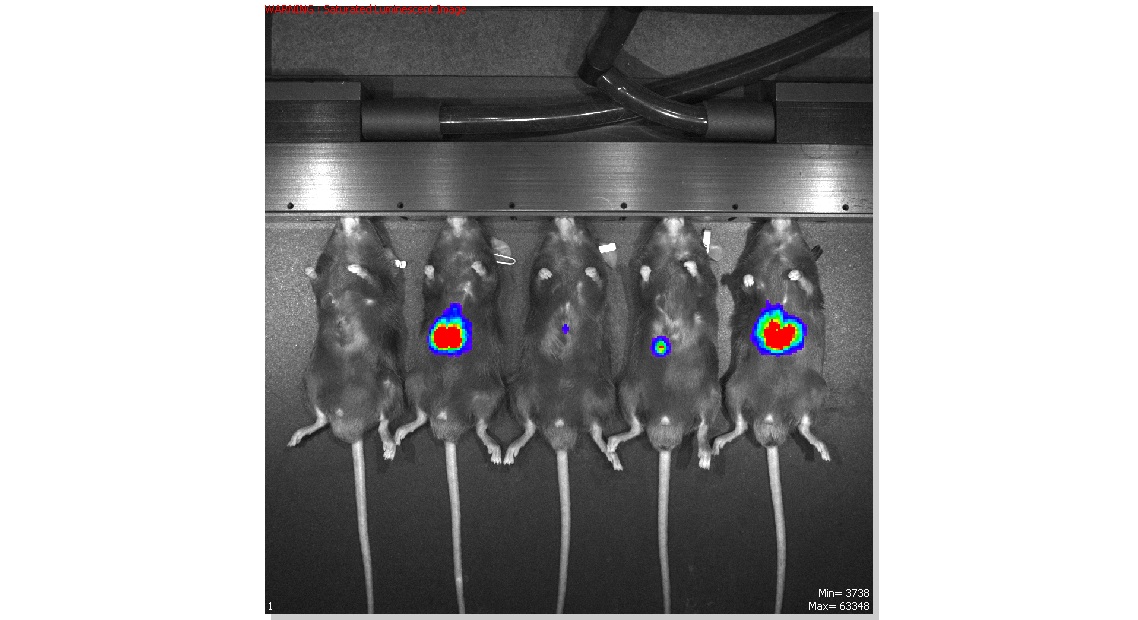

Supplement: Supplementary file 5 — Source data Fig. 3 [file 44321_2026_424_MOESM5_ESM.zip › Figure 3 Source Data/Figure 3D/Day 14/OMV-1.tif]

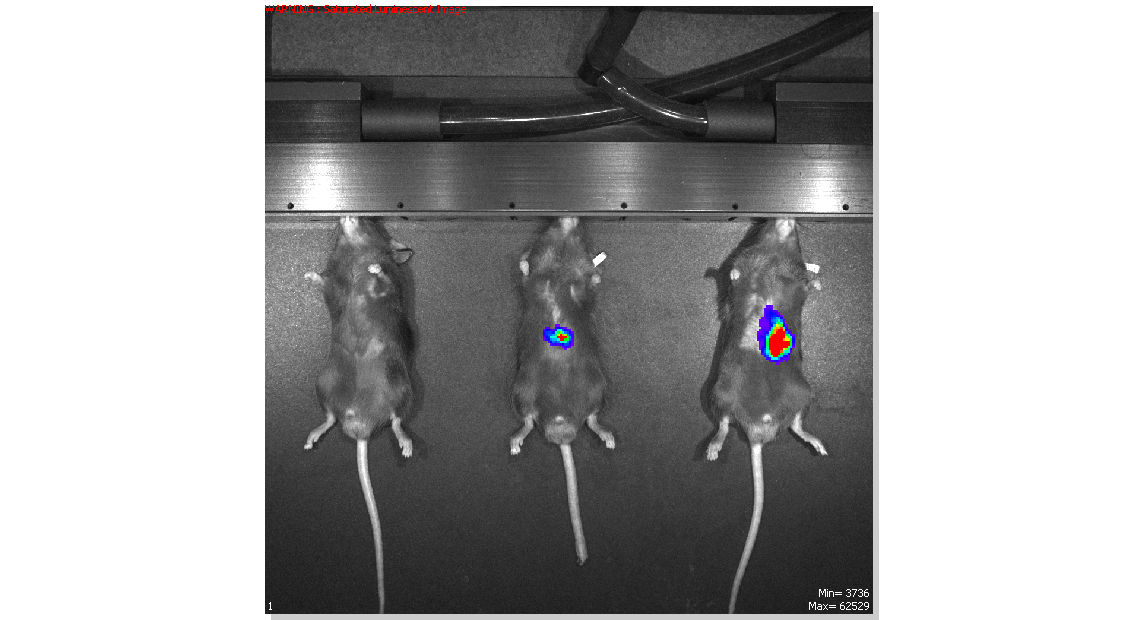

Supplement: Supplementary file 5 — Source data Fig. 3 [file 44321_2026_424_MOESM5_ESM.zip › Figure 3 Source Data/Figure 3D/Day 14/LFZ-PD1.tif]

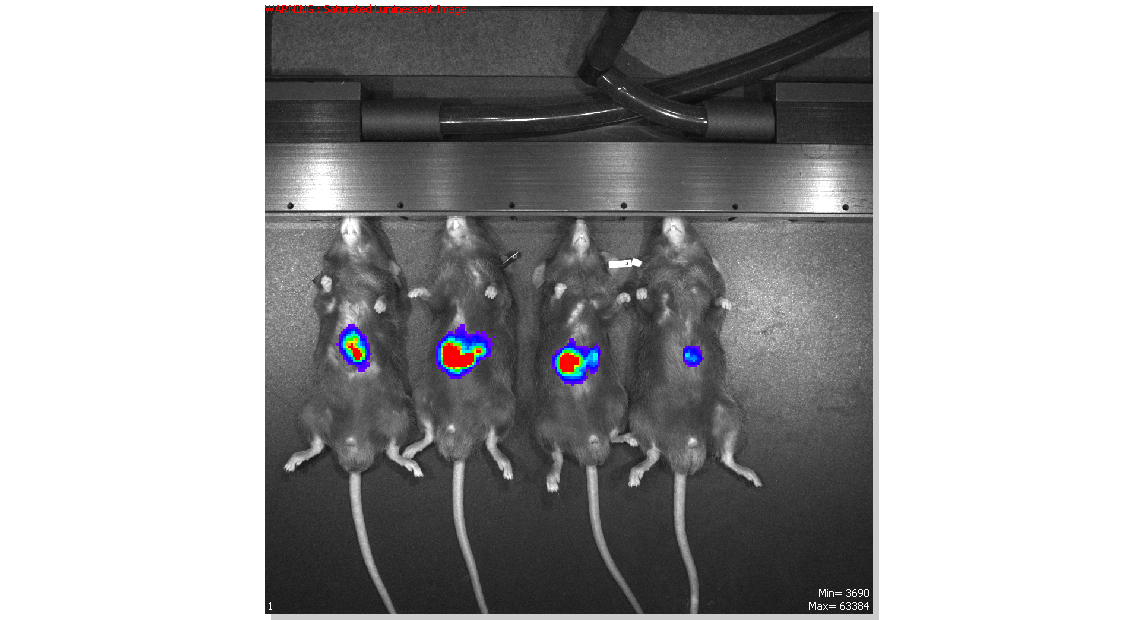

Supplement: Supplementary file 5 — Source data Fig. 3 [file 44321_2026_424_MOESM5_ESM.zip › Figure 3 Source Data/Figure 3D/Day 14/LFZ-ND-PBS.tif]

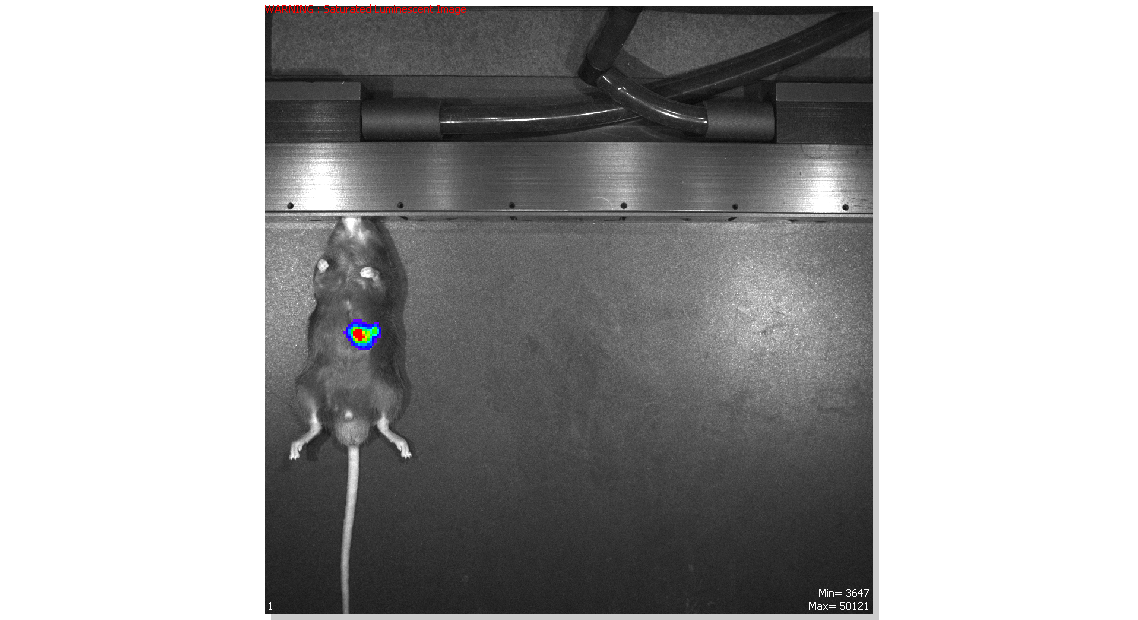

Supplement: Supplementary file 5 — Source data Fig. 3 [file 44321_2026_424_MOESM5_ESM.zip › Figure 3 Source Data/Figure 3D/Day 14/OMV-2.tif]

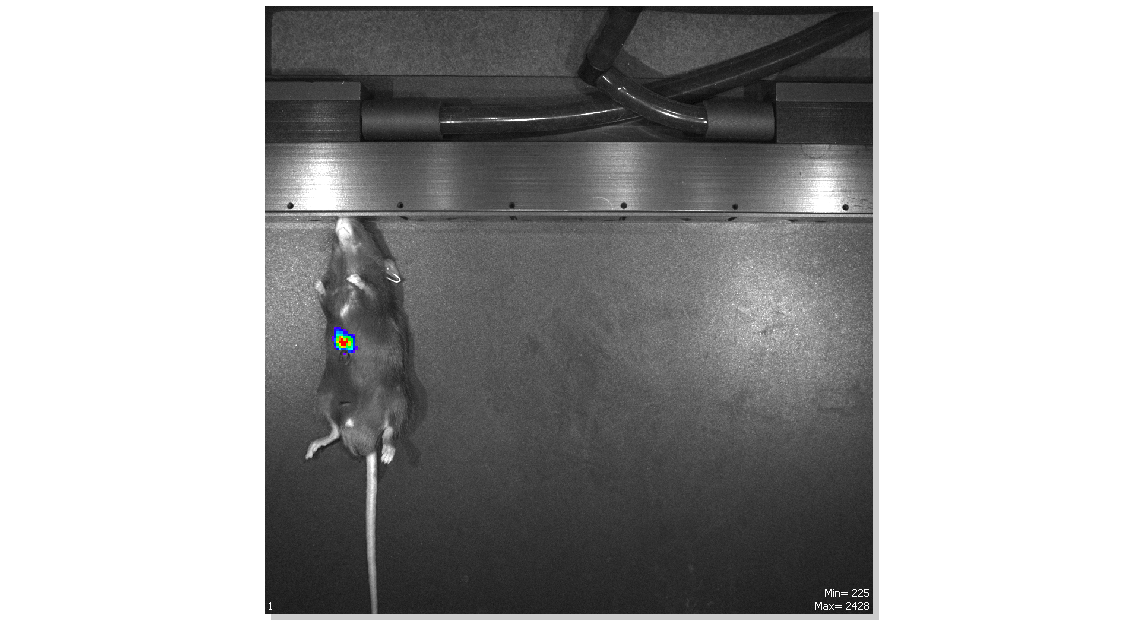

Supplement: Supplementary file 5 — Source data Fig. 3 [file 44321_2026_424_MOESM5_ESM.zip › Figure 3 Source Data/Figure 3D/Day 14/NF-FC-2.tif]

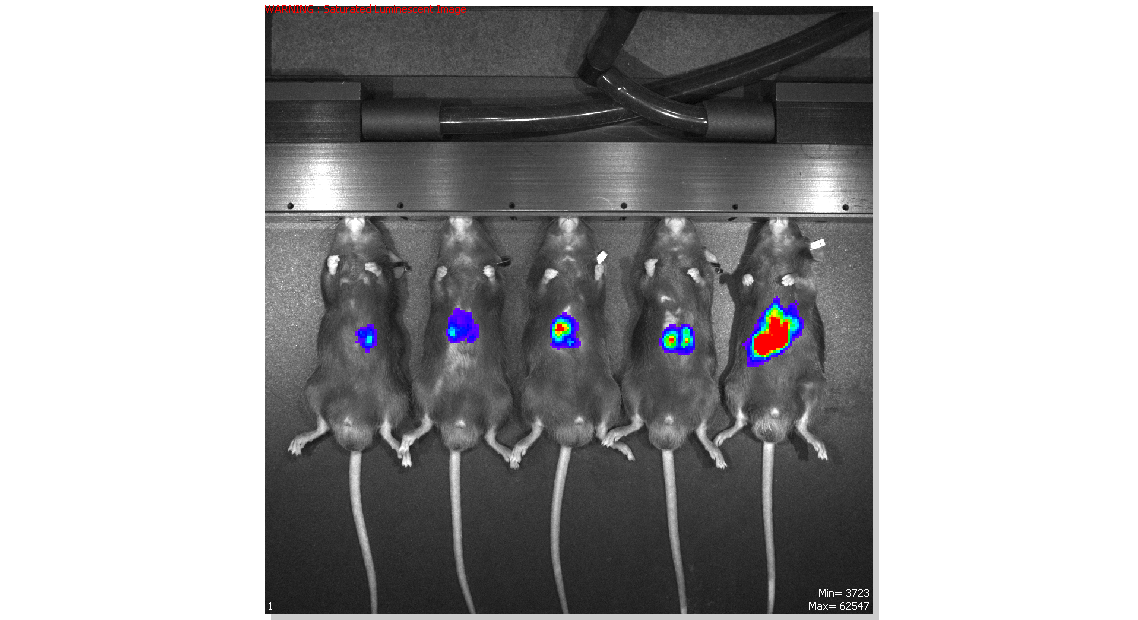

Supplement: Supplementary file 5 — Source data Fig. 3 [file 44321_2026_424_MOESM5_ESM.zip › Figure 3 Source Data/Figure 3D/Day 14/NF-FC-1.tif]

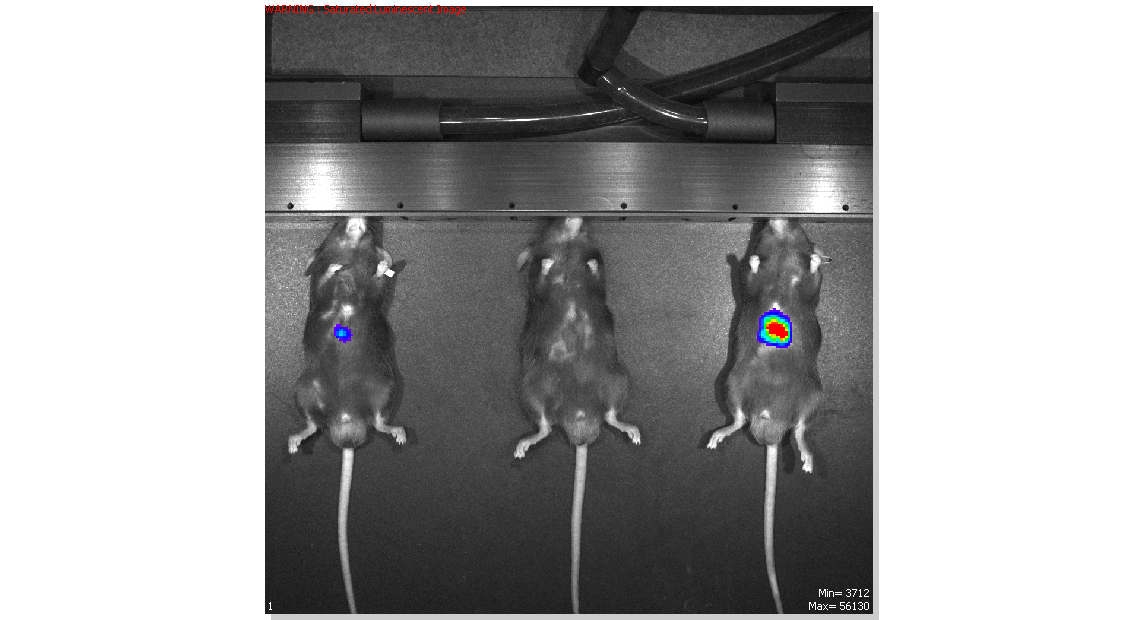

Supplement: Supplementary file 5 — Source data Fig. 3 [file 44321_2026_424_MOESM5_ESM.zip › Figure 3 Source Data/Figure 3D/Day 14/MICB-2.tif]

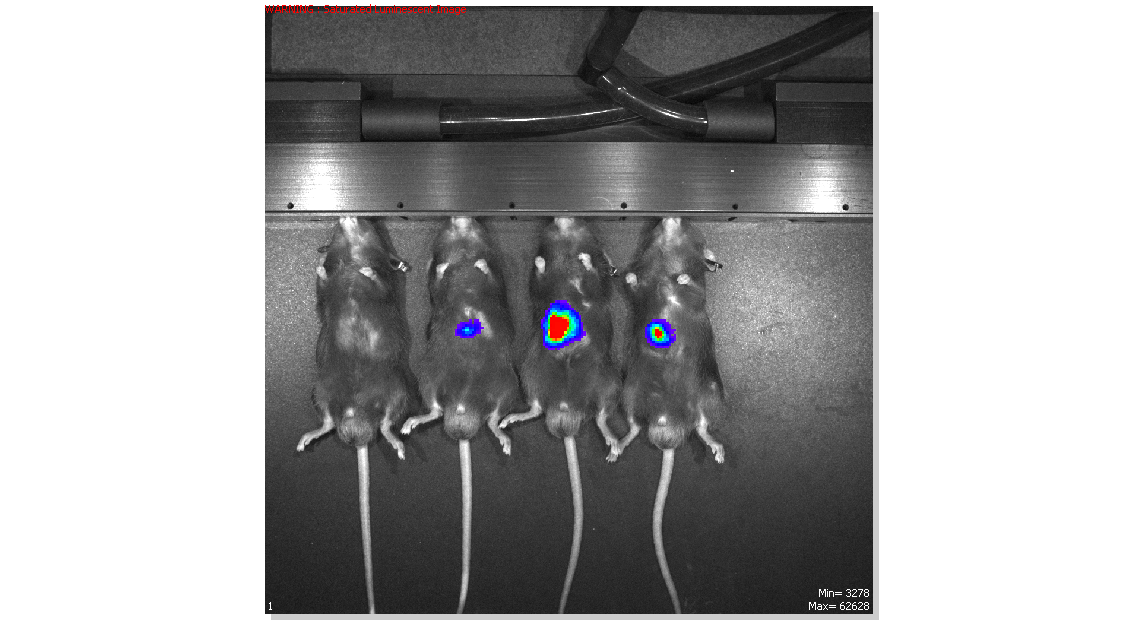

Supplement: Supplementary file 5 — Source data Fig. 3 [file 44321_2026_424_MOESM5_ESM.zip › Figure 3 Source Data/Figure 3D/Day 14/MICB-1.tif]

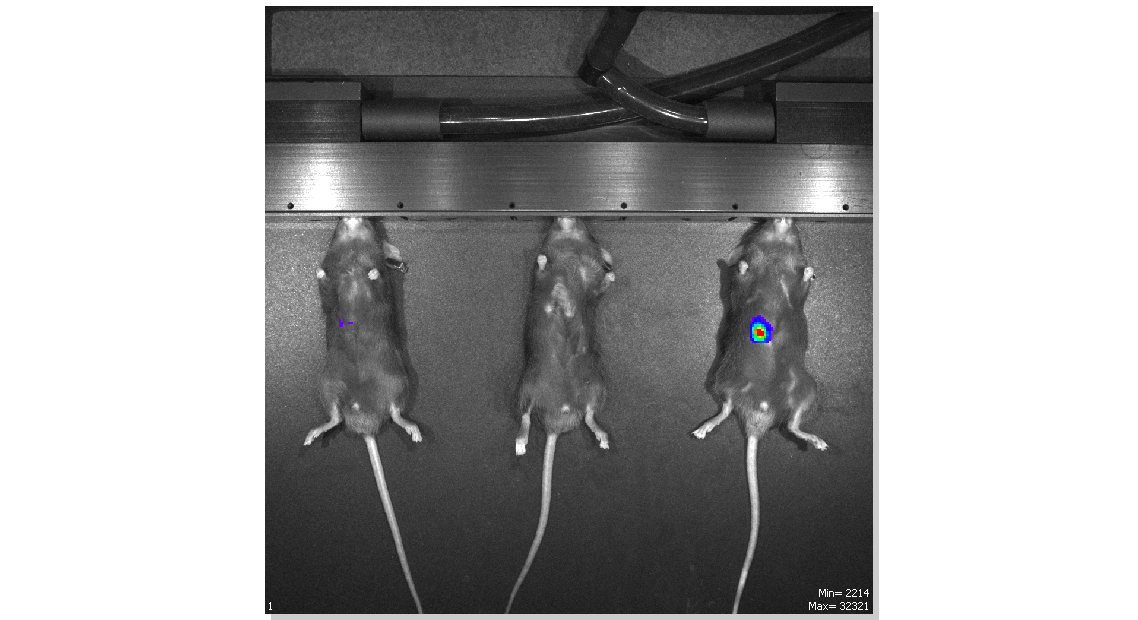

Supplement: Supplementary file 5 — Source data Fig. 3 [file 44321_2026_424_MOESM5_ESM.zip › Figure 3 Source Data/Figure 3D/Day 14/LFZ-NEO+PCSK9.tif]

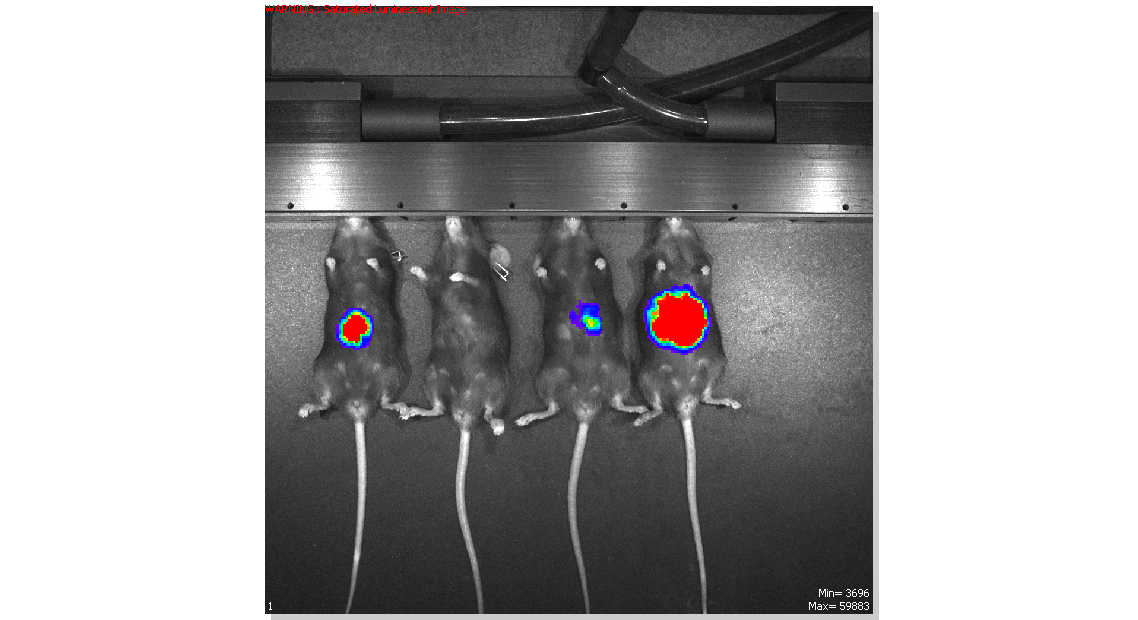

Supplement: Supplementary file 5 — Source data Fig. 3 [file 44321_2026_424_MOESM5_ESM.zip › Figure 3 Source Data/Figure 3D/Day 14/LFZ-MCD MA.tif]

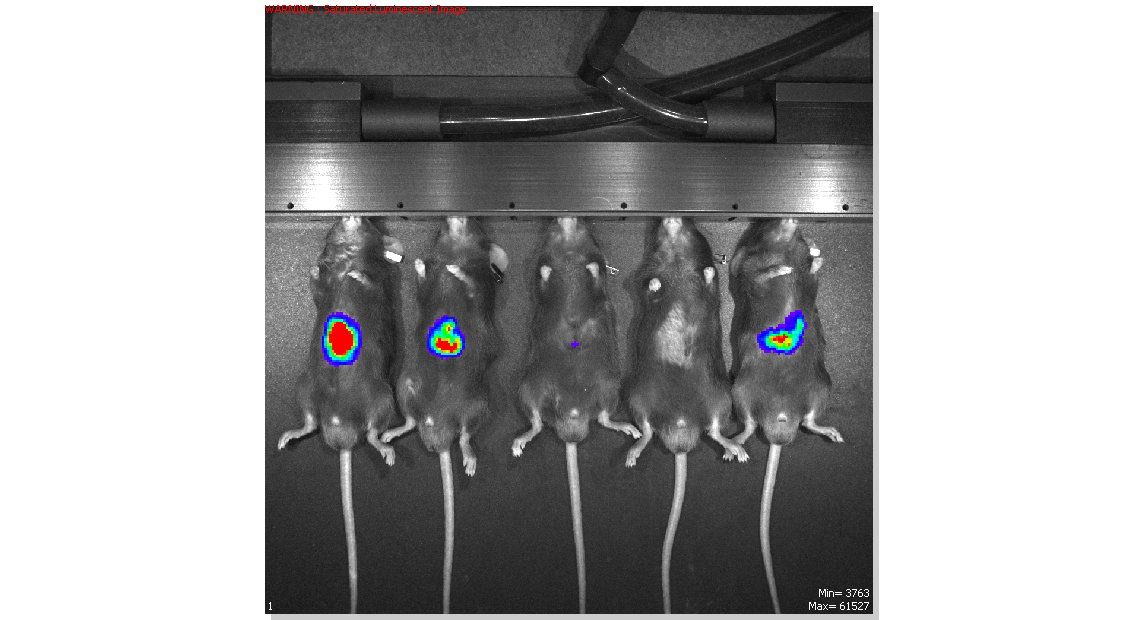

Supplement: Supplementary file 5 — Source data Fig. 3 [file 44321_2026_424_MOESM5_ESM.zip › Figure 3 Source Data/Figure 3D/Day 14/PBS-1.tif]

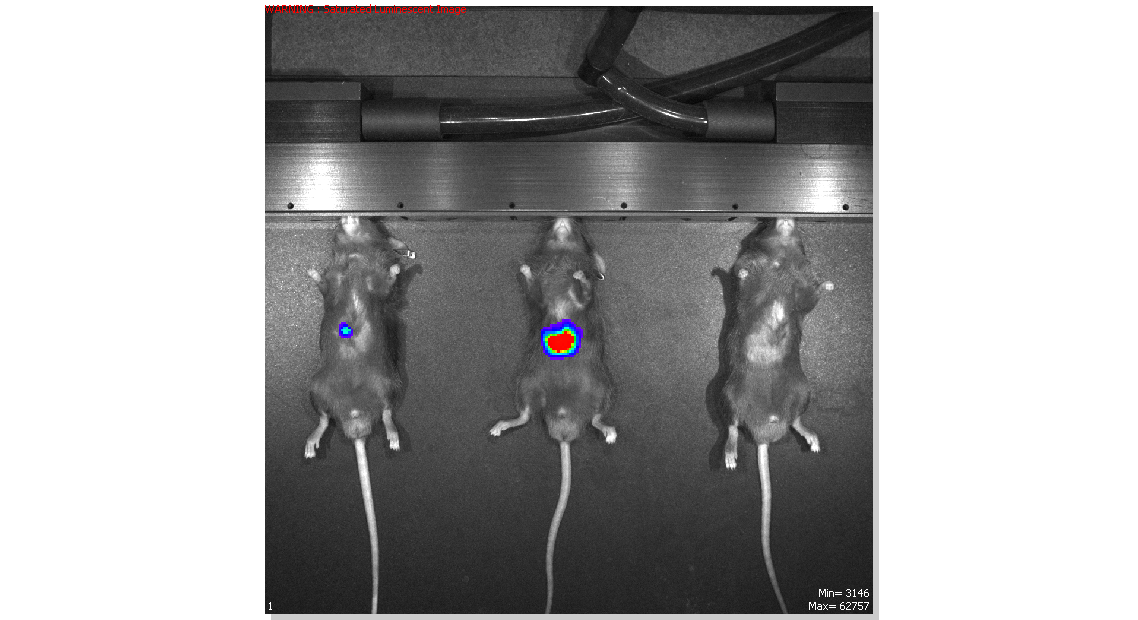

Supplement: Supplementary file 5 — Source data Fig. 3 [file 44321_2026_424_MOESM5_ESM.zip › Figure 3 Source Data/Figure 3D/Day 14/LFZ-NEO.tif]

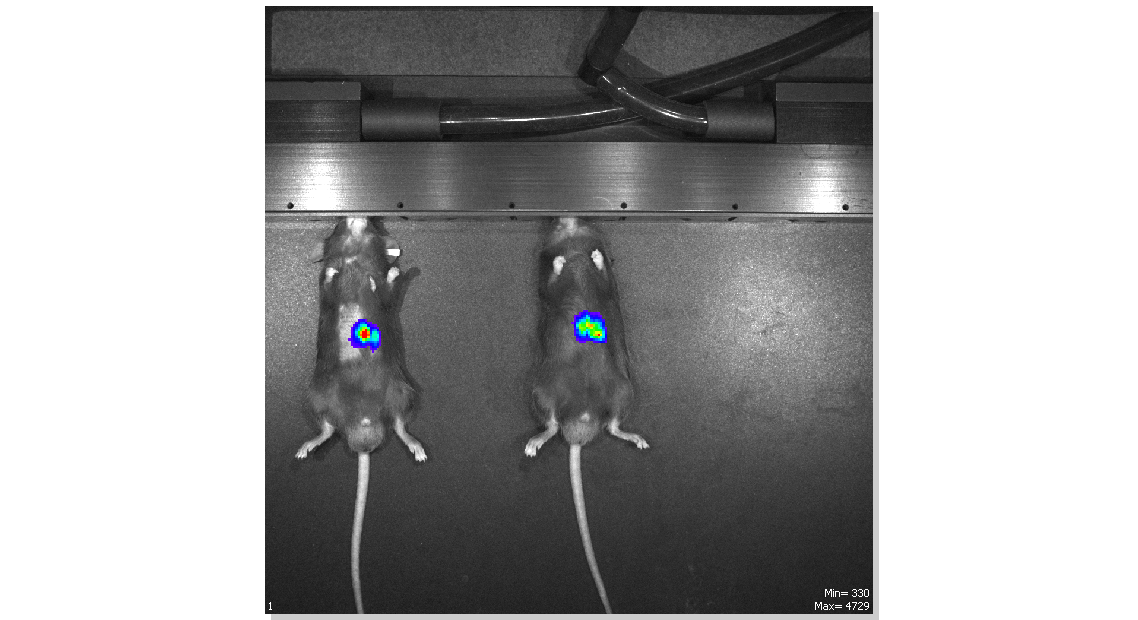

Supplement: Supplementary file 5 — Source data Fig. 3 [file 44321_2026_424_MOESM5_ESM.zip › Figure 3 Source Data/Figure 3D/Day 14/PBS-2.tif]

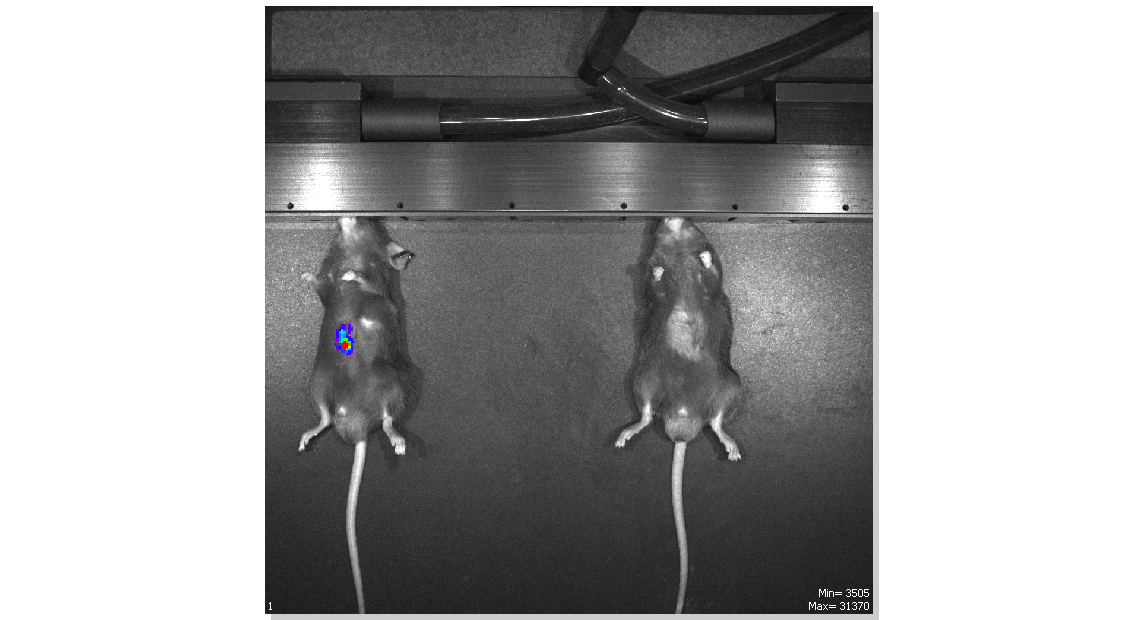

Supplement: Supplementary file 5 — Source data Fig. 3 [file 44321_2026_424_MOESM5_ESM.zip › Figure 3 Source Data/Figure 3D/Day 14/PBS-722-720.tif]

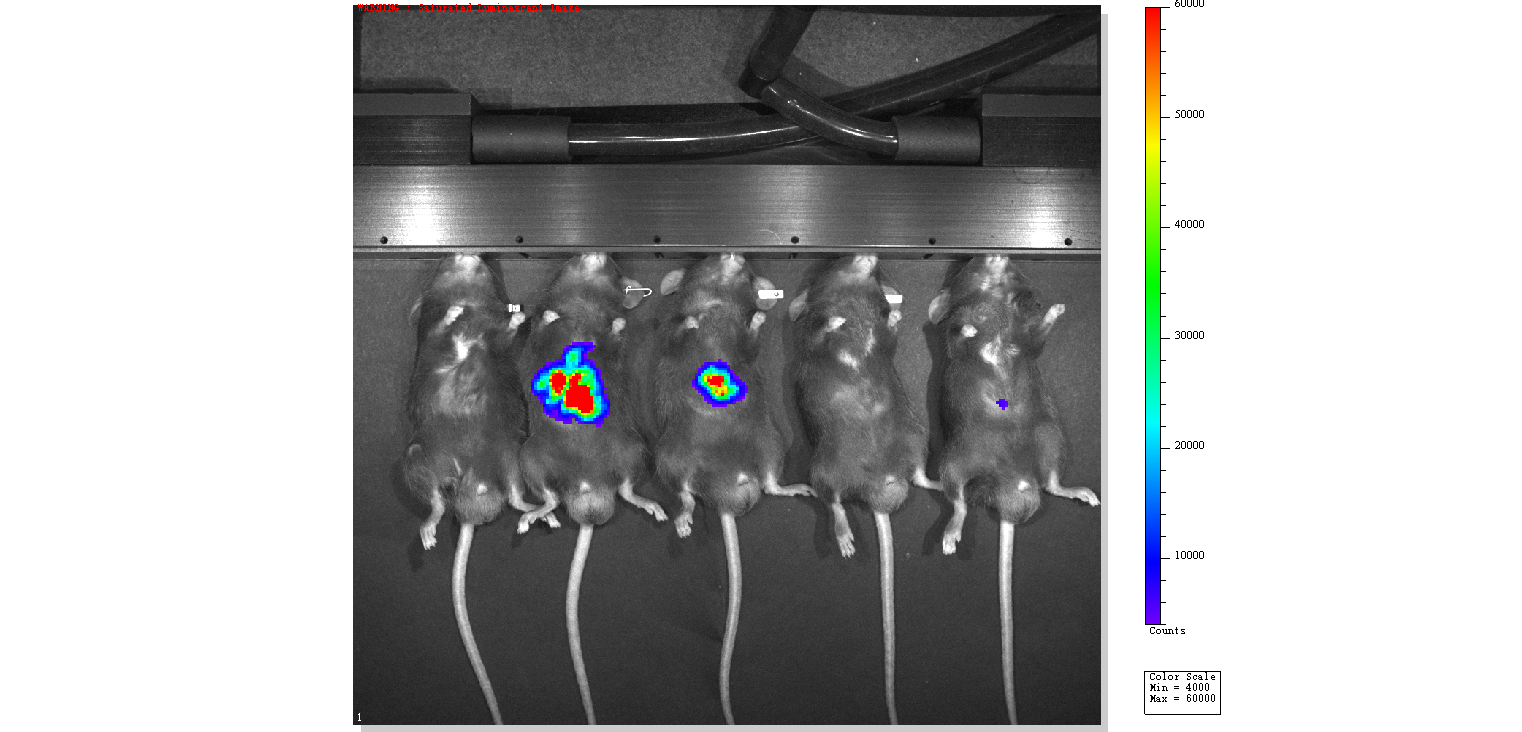

Supplement: Supplementary file 5 — Source data Fig. 3 [file 44321_2026_424_MOESM5_ESM.zip › Figure 3 Source Data/Figure 3D/Day 35/G4.tif]

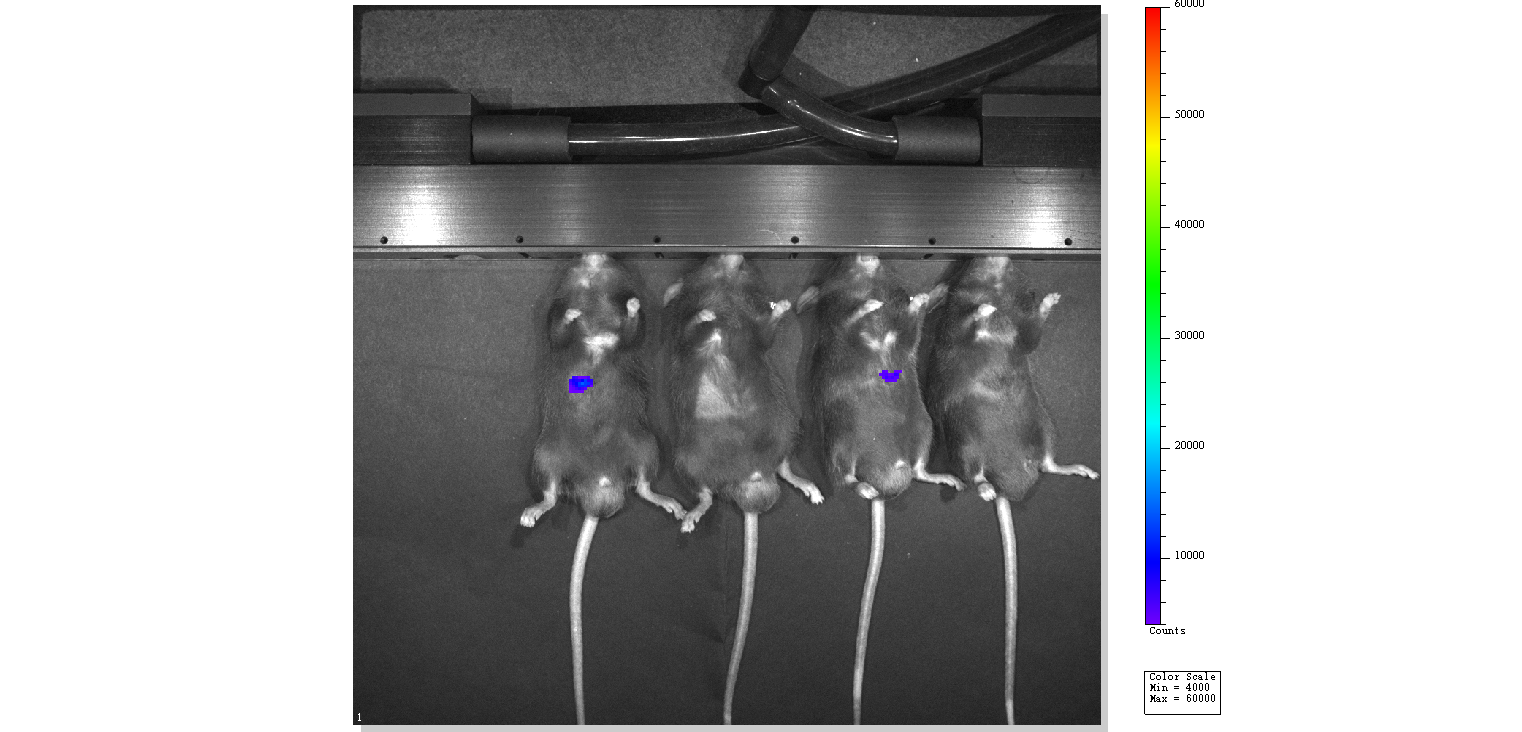

Supplement: Supplementary file 5 — Source data Fig. 3 [file 44321_2026_424_MOESM5_ESM.zip › Figure 3 Source Data/Figure 3D/Day 35/G2.tif]

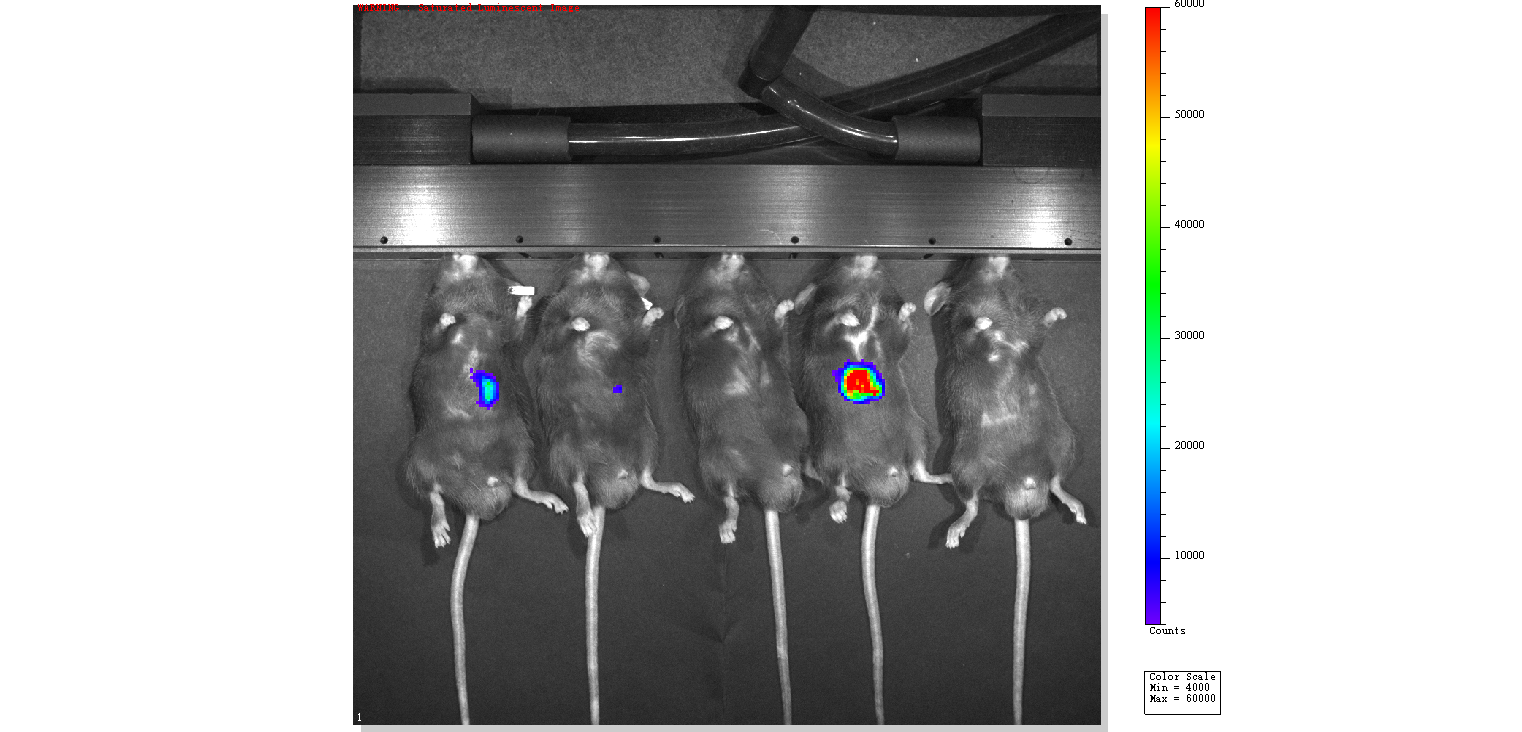

Supplement: Supplementary file 5 — Source data Fig. 3 [file 44321_2026_424_MOESM5_ESM.zip › Figure 3 Source Data/Figure 3D/Day 35/G3.tif]

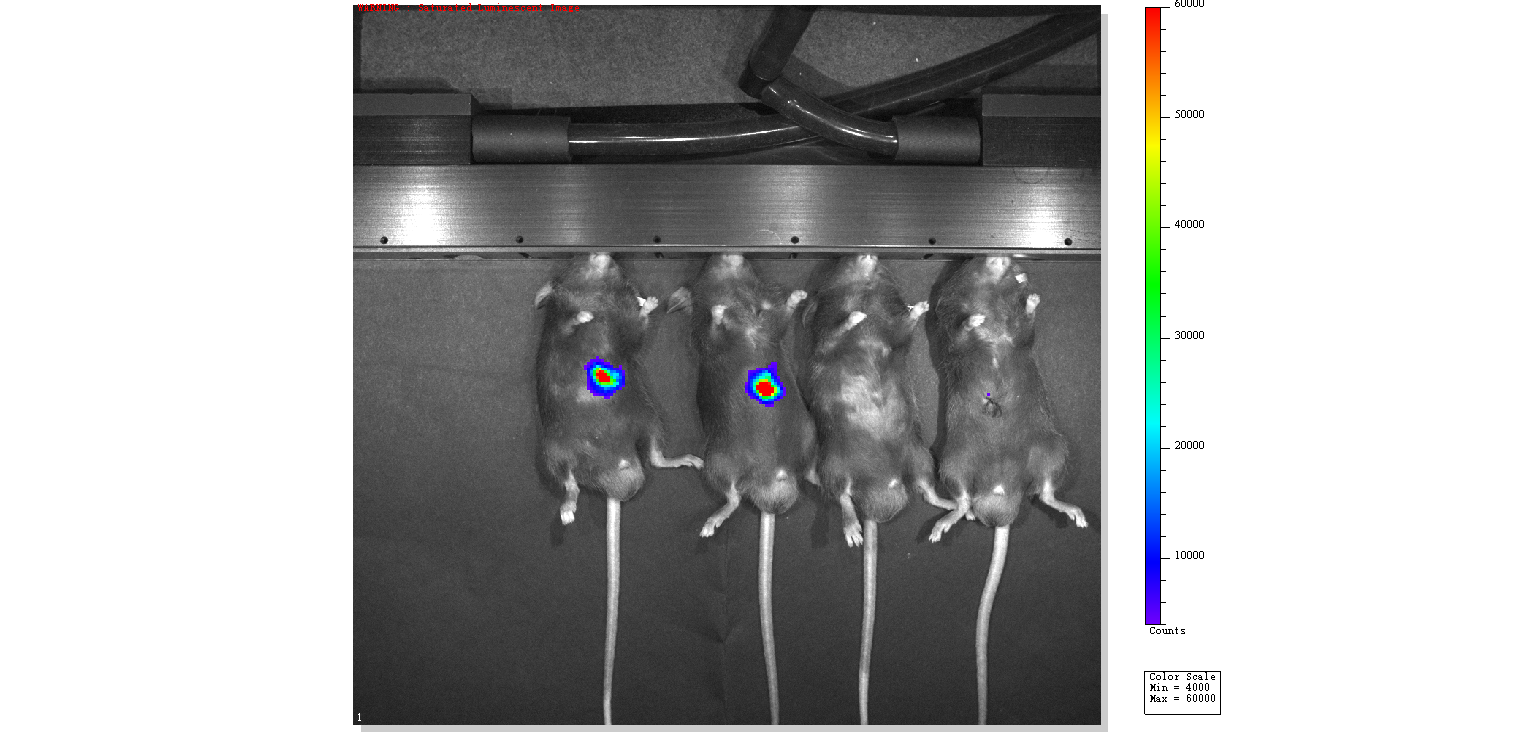

Supplement: Supplementary file 5 — Source data Fig. 3 [file 44321_2026_424_MOESM5_ESM.zip › Figure 3 Source Data/Figure 3D/Day 35/G1.tif]

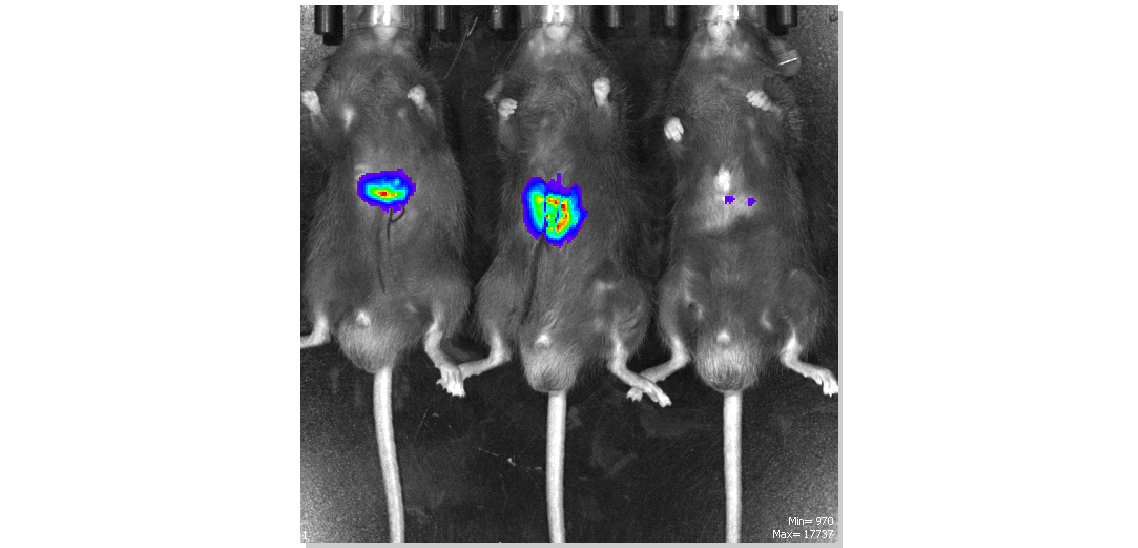

Supplement: Supplementary file 5 — Source data Fig. 3 [file 44321_2026_424_MOESM5_ESM.zip › Figure 3 Source Data/Figure 3D/Day 0/13.tif]

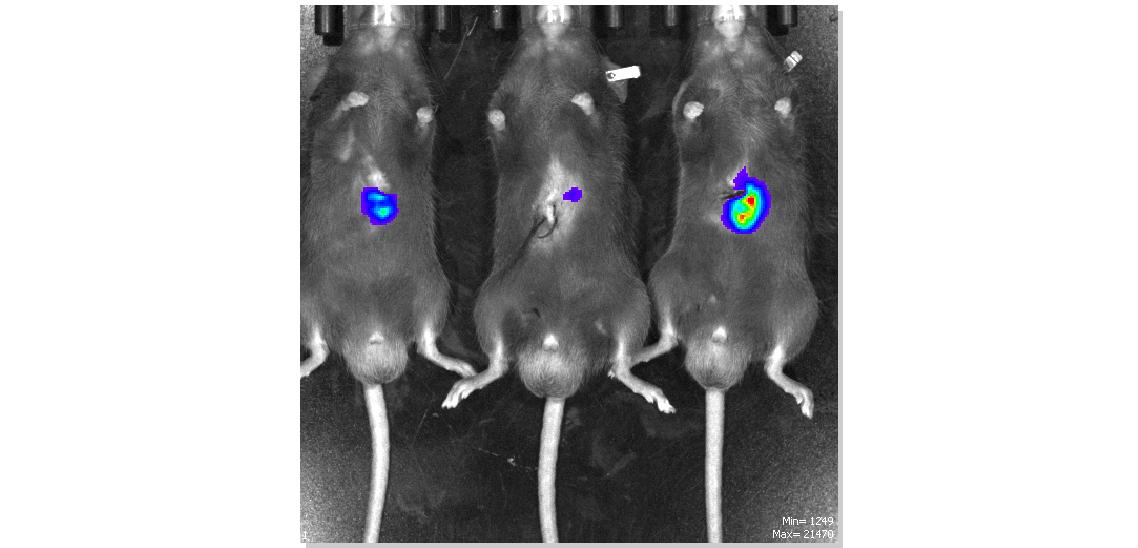

Supplement: Supplementary file 5 — Source data Fig. 3 [file 44321_2026_424_MOESM5_ESM.zip › Figure 3 Source Data/Figure 3D/Day 0/12.tif]

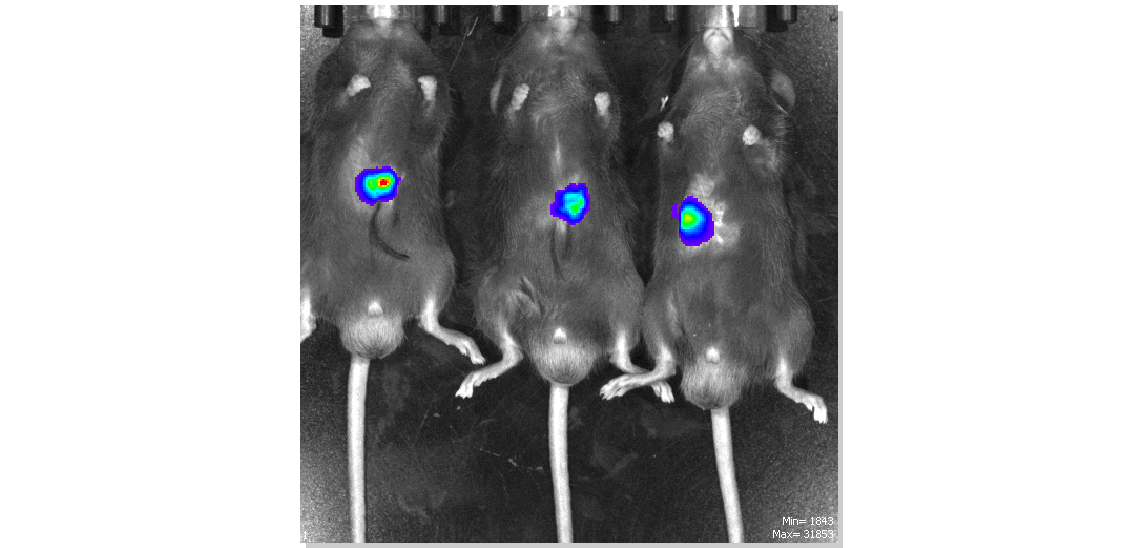

Supplement: Supplementary file 5 — Source data Fig. 3 [file 44321_2026_424_MOESM5_ESM.zip › Figure 3 Source Data/Figure 3D/Day 0/10.tif]

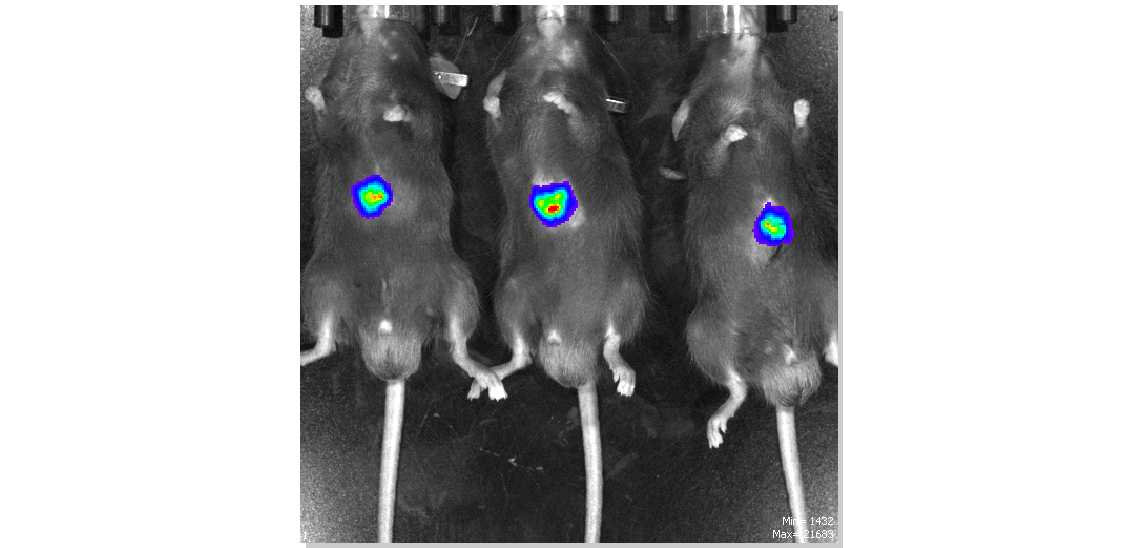

Supplement: Supplementary file 5 — Source data Fig. 3 [file 44321_2026_424_MOESM5_ESM.zip › Figure 3 Source Data/Figure 3D/Day 0/11.tif]

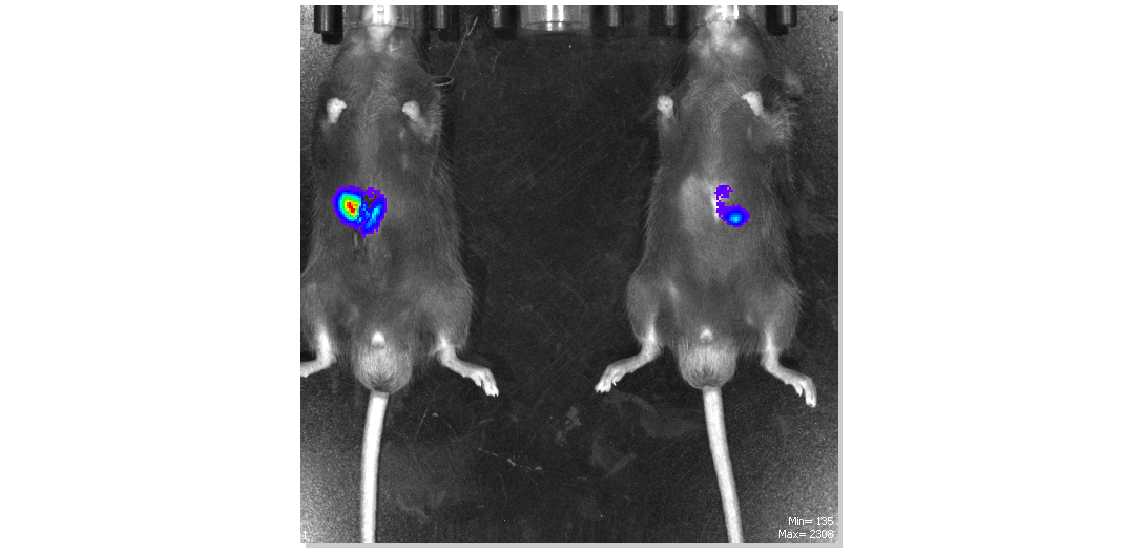

Supplement: Supplementary file 5 — Source data Fig. 3 [file 44321_2026_424_MOESM5_ESM.zip › Figure 3 Source Data/Figure 3D/Day 0/15.tif]

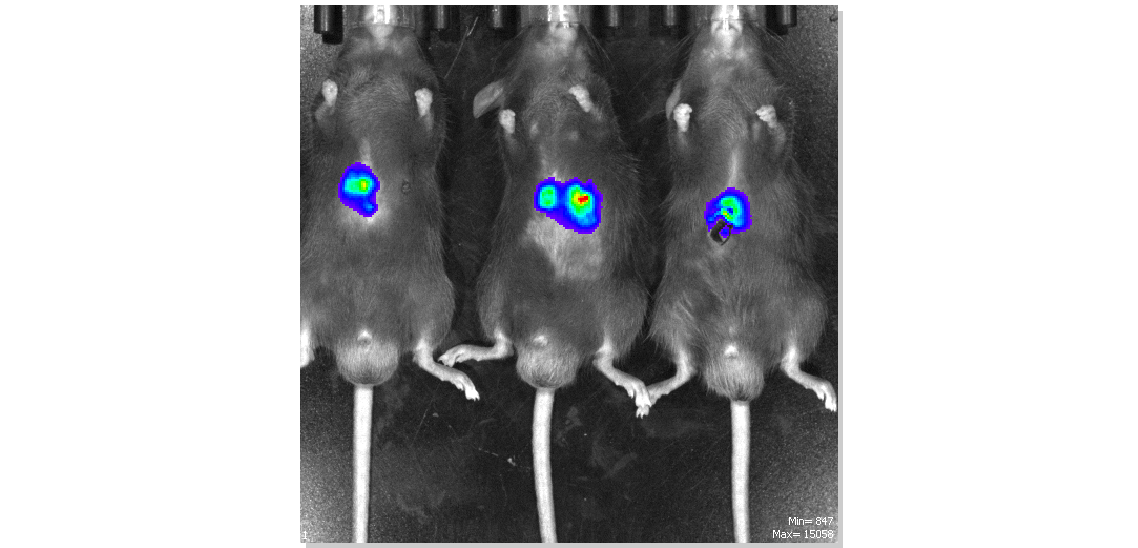

Supplement: Supplementary file 5 — Source data Fig. 3 [file 44321_2026_424_MOESM5_ESM.zip › Figure 3 Source Data/Figure 3D/Day 0/14.tif]

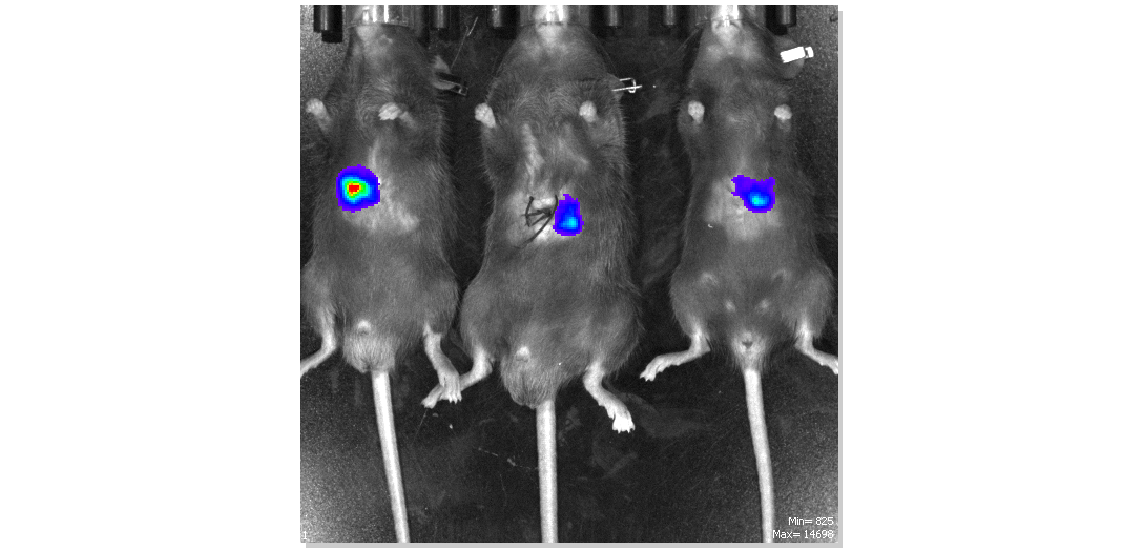

Supplement: Supplementary file 5 — Source data Fig. 3 [file 44321_2026_424_MOESM5_ESM.zip › Figure 3 Source Data/Figure 3D/Day 0/16.tif]

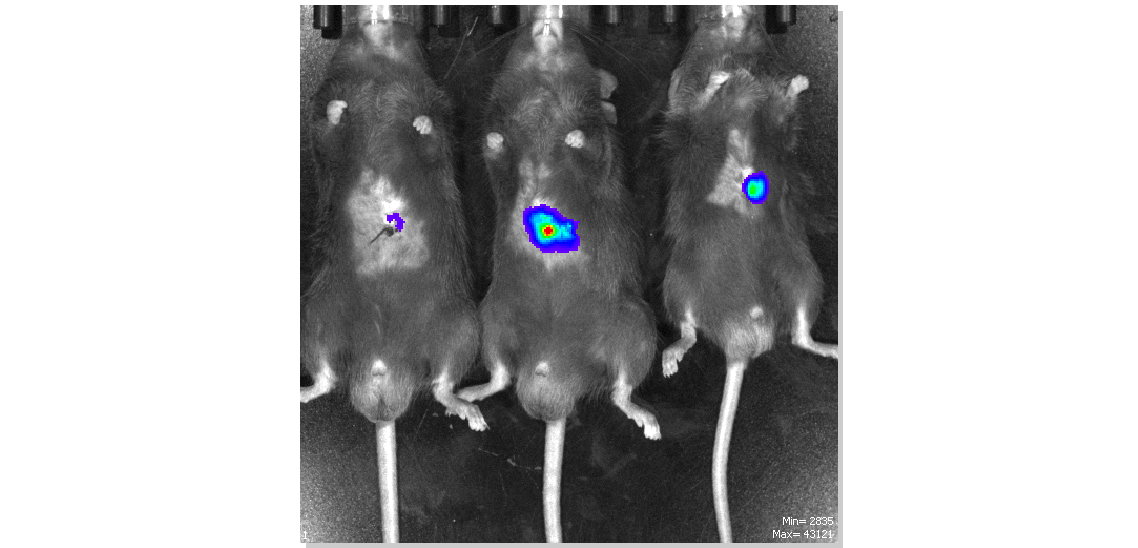

Supplement: Supplementary file 5 — Source data Fig. 3 [file 44321_2026_424_MOESM5_ESM.zip › Figure 3 Source Data/Figure 3D/Day 0/17.tif]

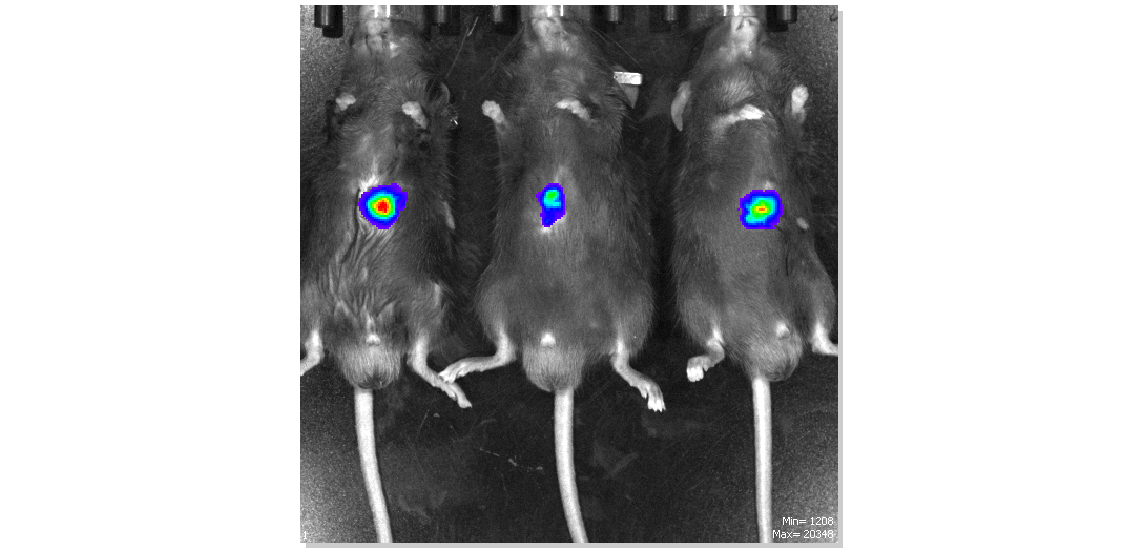

Supplement: Supplementary file 5 — Source data Fig. 3 [file 44321_2026_424_MOESM5_ESM.zip › Figure 3 Source Data/Figure 3D/Day 0/9.tif]

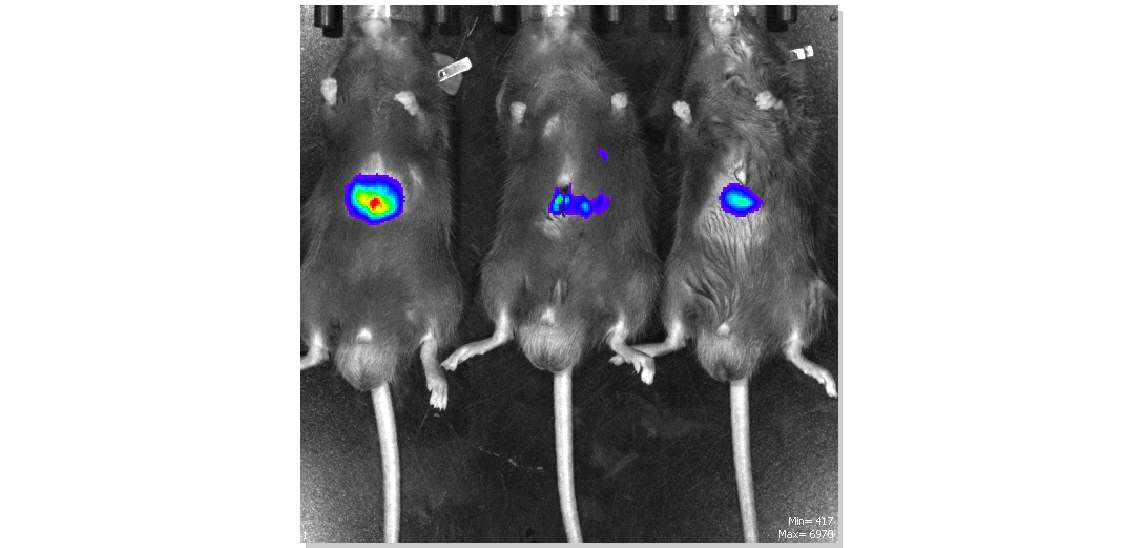

Supplement: Supplementary file 5 — Source data Fig. 3 [file 44321_2026_424_MOESM5_ESM.zip › Figure 3 Source Data/Figure 3D/Day 0/8.tif]

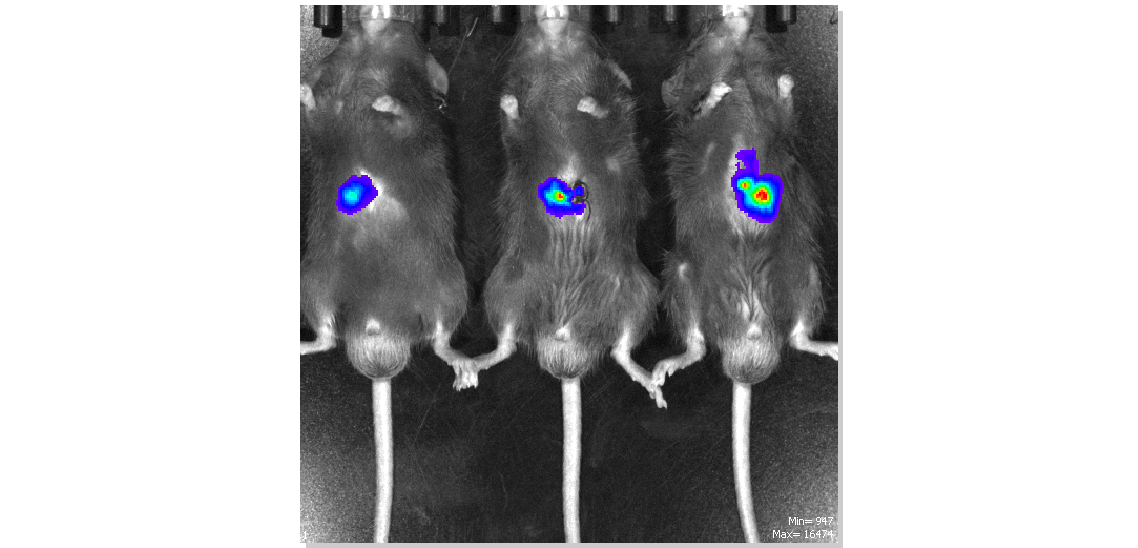

Supplement: Supplementary file 5 — Source data Fig. 3 [file 44321_2026_424_MOESM5_ESM.zip › Figure 3 Source Data/Figure 3D/Day 0/3.tif]

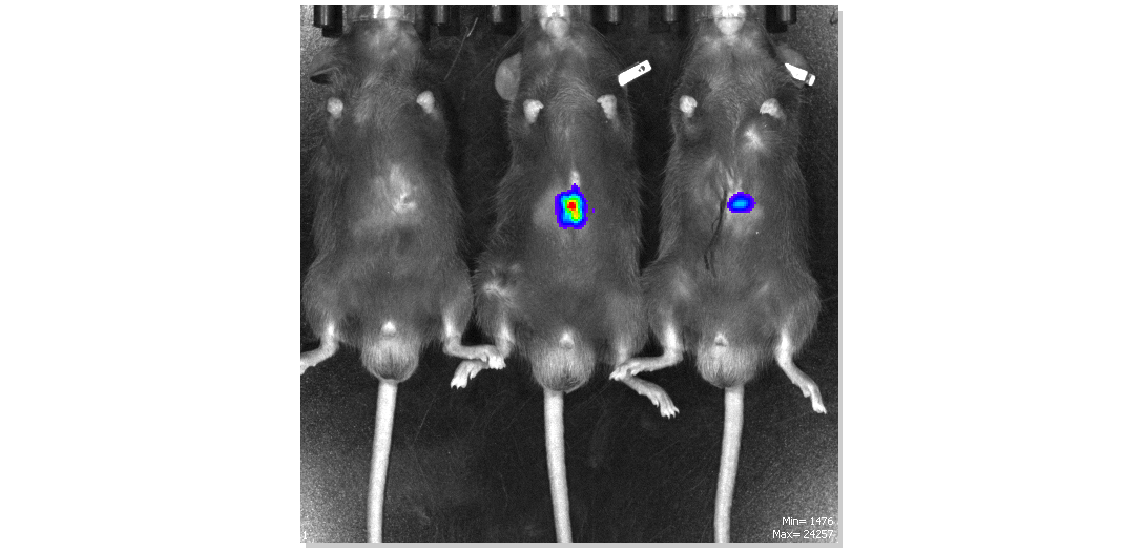

Supplement: Supplementary file 5 — Source data Fig. 3 [file 44321_2026_424_MOESM5_ESM.zip › Figure 3 Source Data/Figure 3D/Day 0/2.tif]

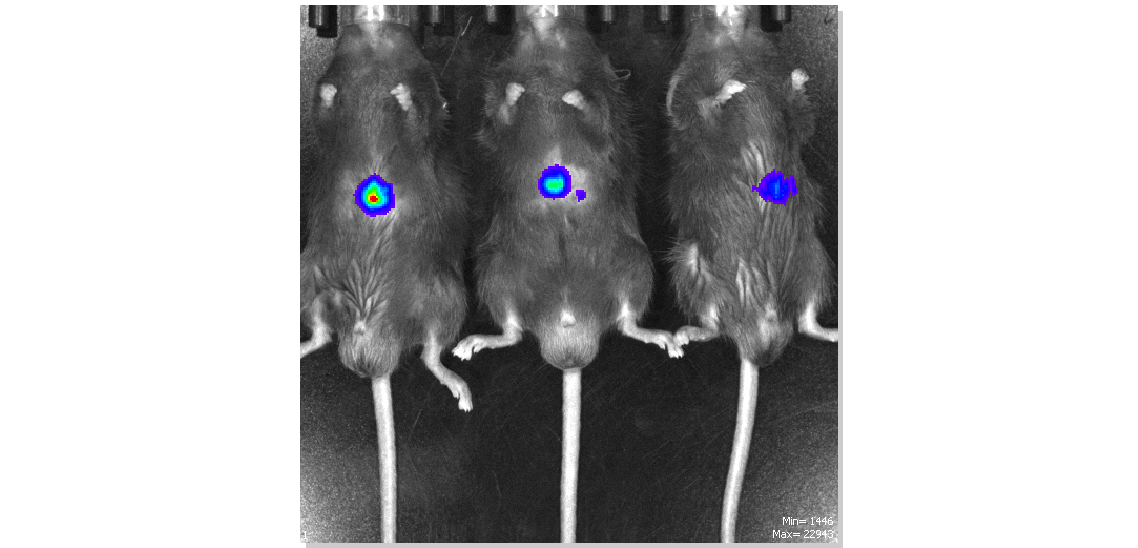

Supplement: Supplementary file 5 — Source data Fig. 3 [file 44321_2026_424_MOESM5_ESM.zip › Figure 3 Source Data/Figure 3D/Day 0/1.tif]

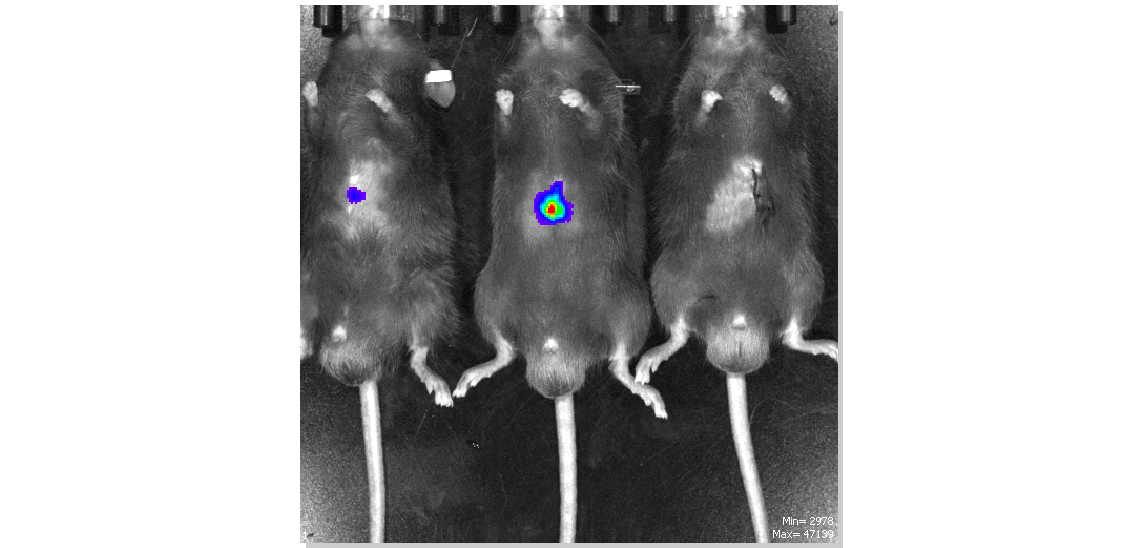

Supplement: Supplementary file 5 — Source data Fig. 3 [file 44321_2026_424_MOESM5_ESM.zip › Figure 3 Source Data/Figure 3D/Day 0/5.tif]

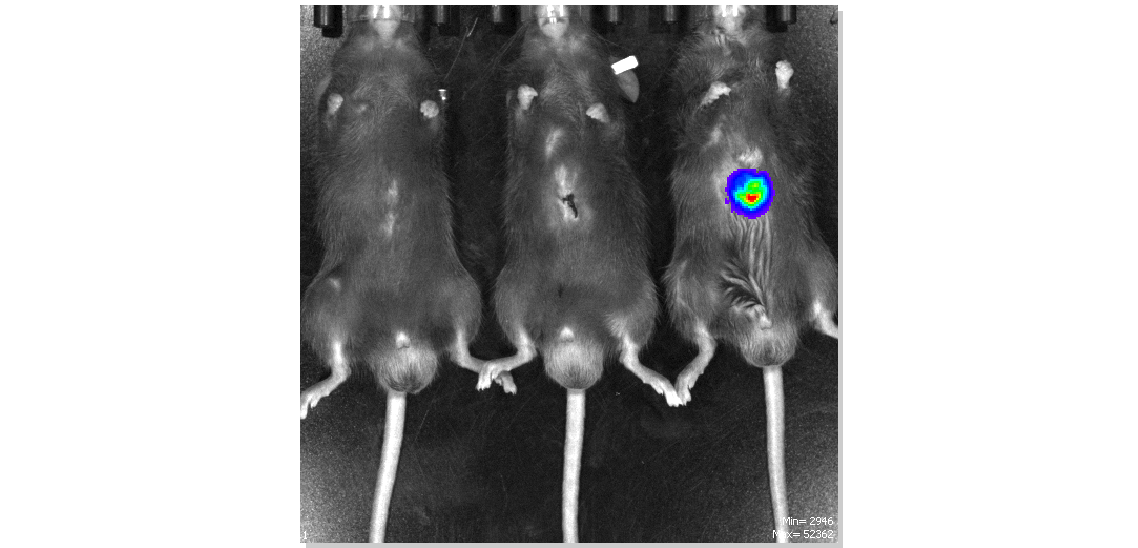

Supplement: Supplementary file 5 — Source data Fig. 3 [file 44321_2026_424_MOESM5_ESM.zip › Figure 3 Source Data/Figure 3D/Day 0/4.tif]

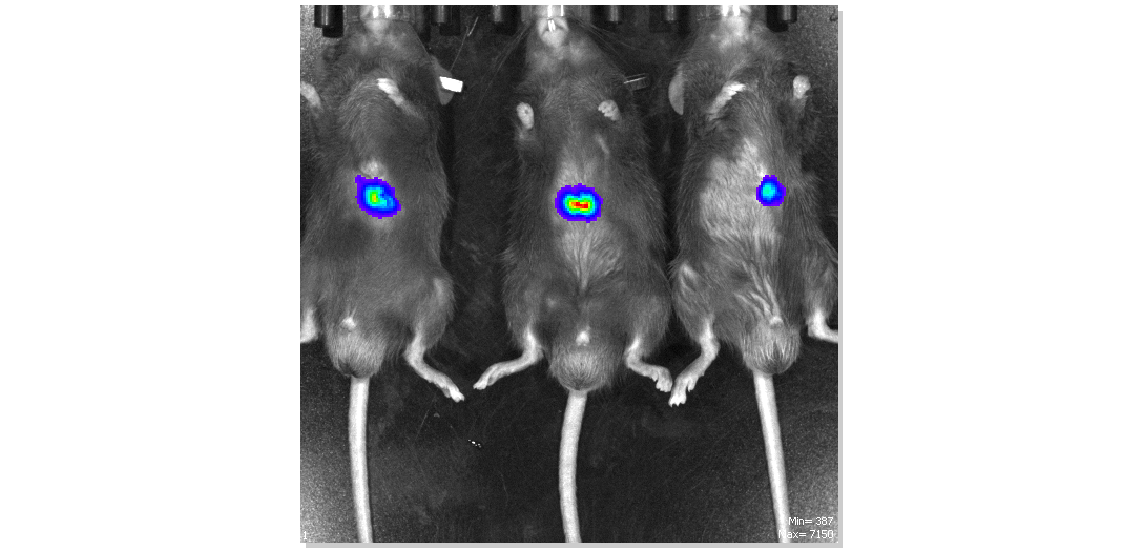

Supplement: Supplementary file 5 — Source data Fig. 3 [file 44321_2026_424_MOESM5_ESM.zip › Figure 3 Source Data/Figure 3D/Day 0/6.tif]

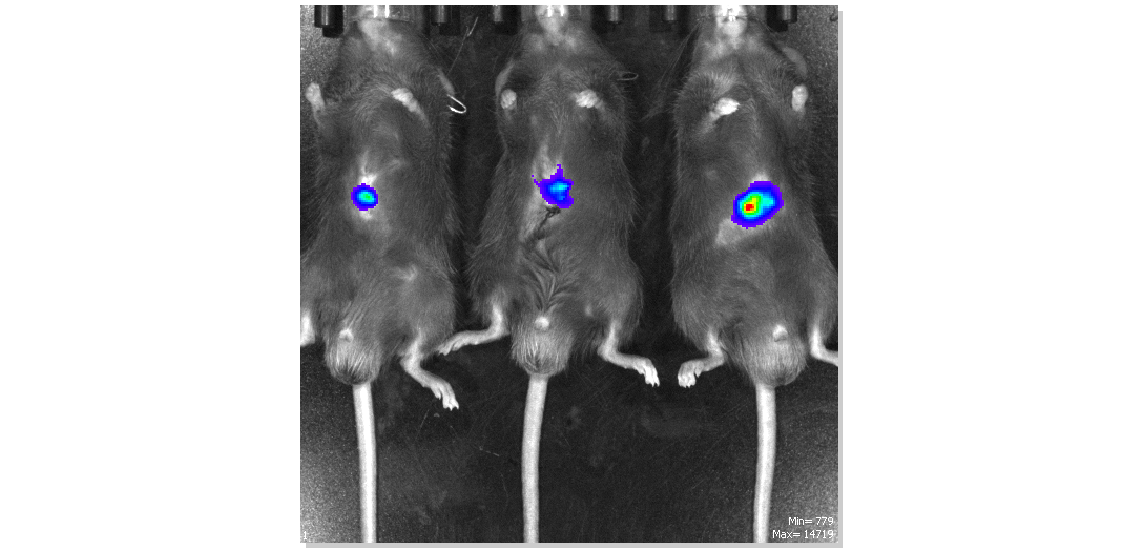

Supplement: Supplementary file 5 — Source data Fig. 3 [file 44321_2026_424_MOESM5_ESM.zip › Figure 3 Source Data/Figure 3D/Day 0/7.tif]

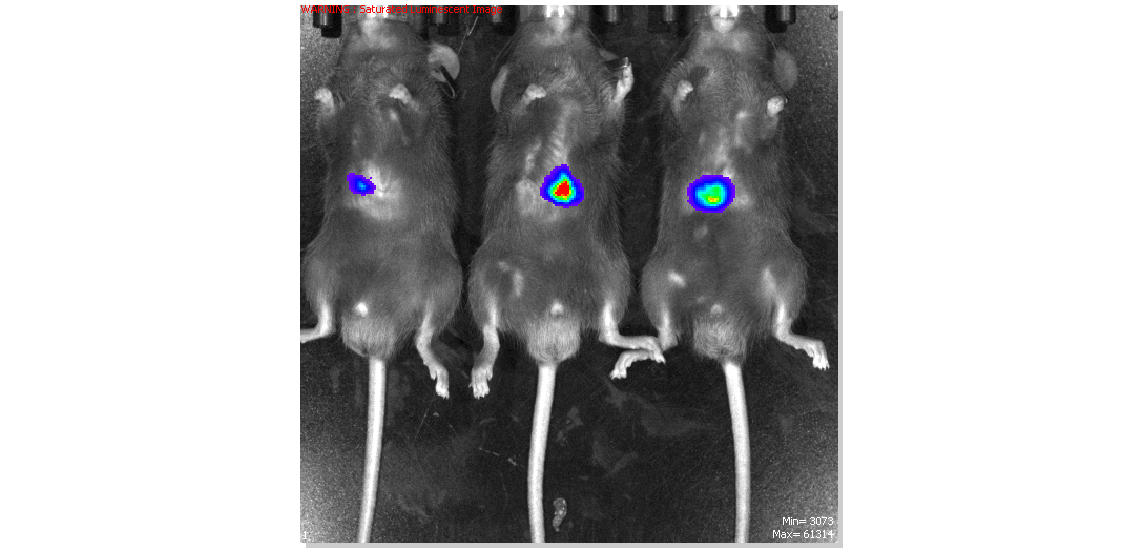

Supplement: Supplementary file 5 — Source data Fig. 3 [file 44321_2026_424_MOESM5_ESM.zip › Figure 3 Source Data/Figure 3D/Day 0/19.tif]
