# Supplementary figures and images for: Neoantigens and shared MICB α3 antigen dual-targeted vaccine generates potent antitumor immunity (part 2 of 2)
Source: EMBO Mol Med. 2026 Apr 17;18(6):2098–123. doi: 10.1038/s44321-026-00424-6 (PMC13269783; doi:10.1038/s44321-026-00424-6)

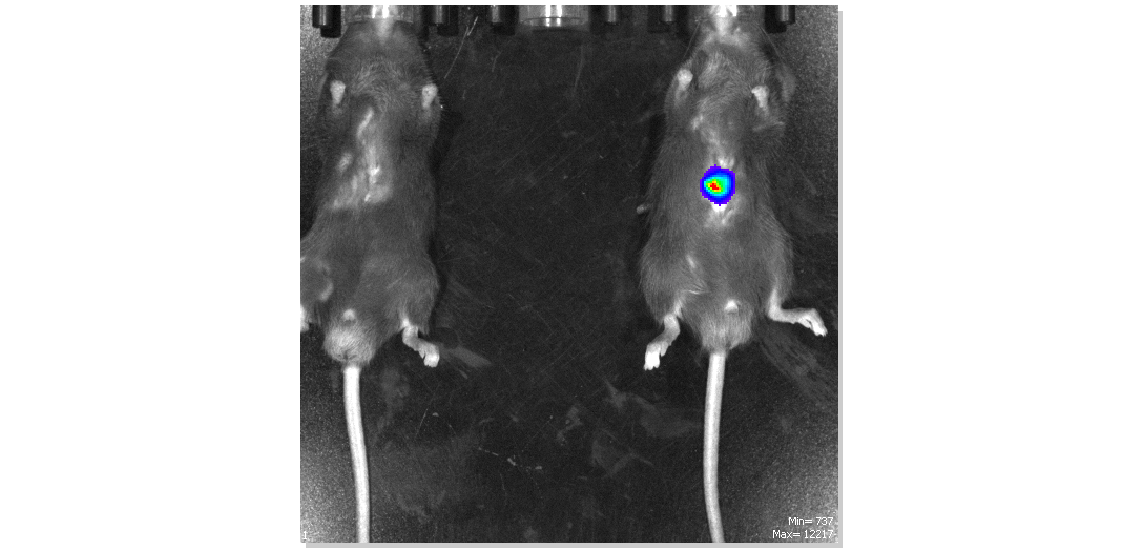

Supplement: Supplementary file 5 — Source data Fig. 3 [file 44321_2026_424_MOESM5_ESM.zip › Figure 3 Source Data/Figure 3D/Day 0/25.tif]

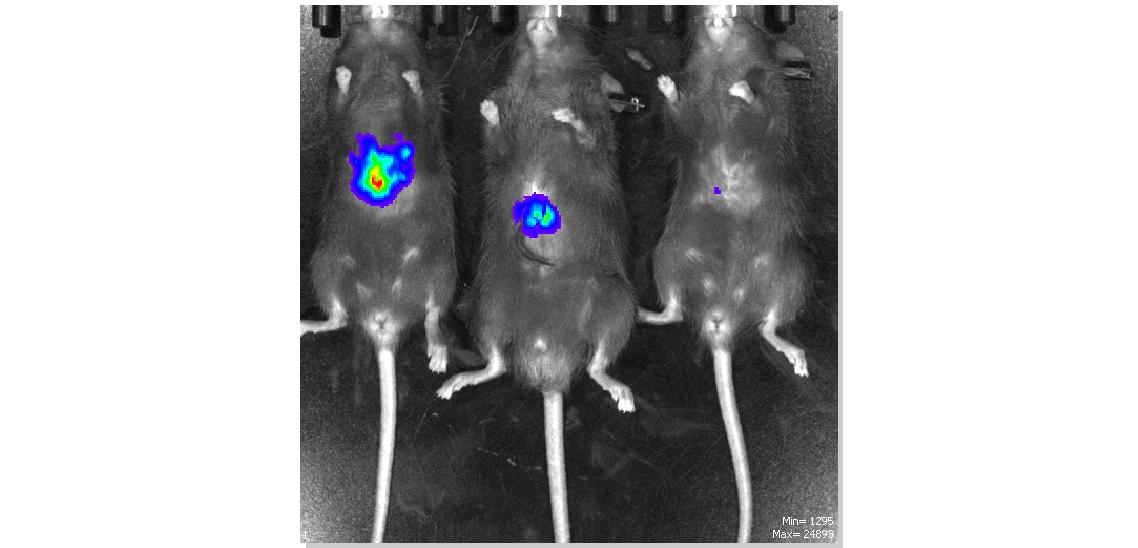

Supplement: Supplementary file 5 — Source data Fig. 3 [file 44321_2026_424_MOESM5_ESM.zip › Figure 3 Source Data/Figure 3D/Day 0/24.tif]

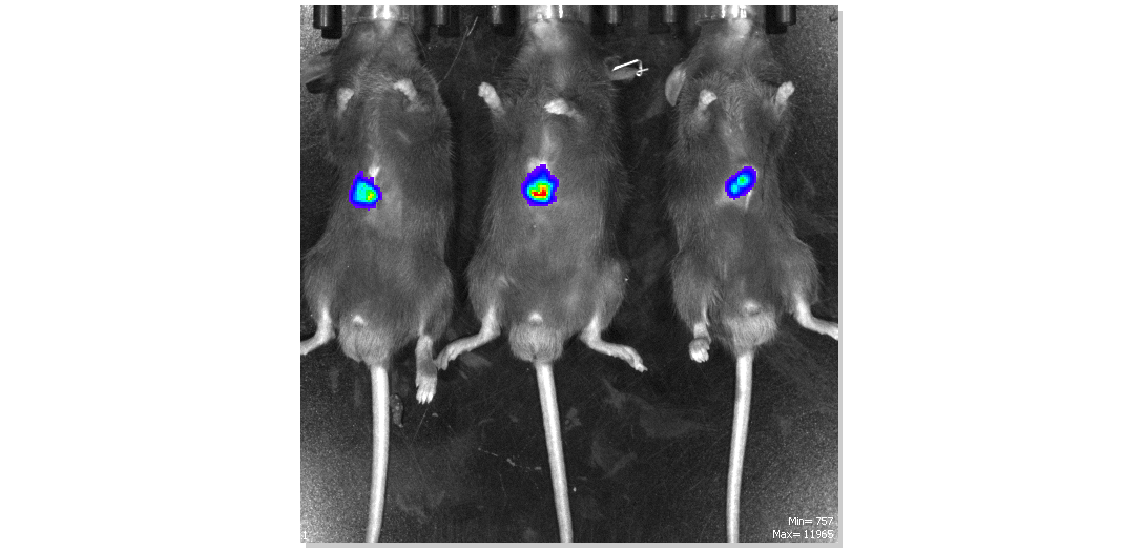

Supplement: Supplementary file 5 — Source data Fig. 3 [file 44321_2026_424_MOESM5_ESM.zip › Figure 3 Source Data/Figure 3D/Day 0/18.tif]

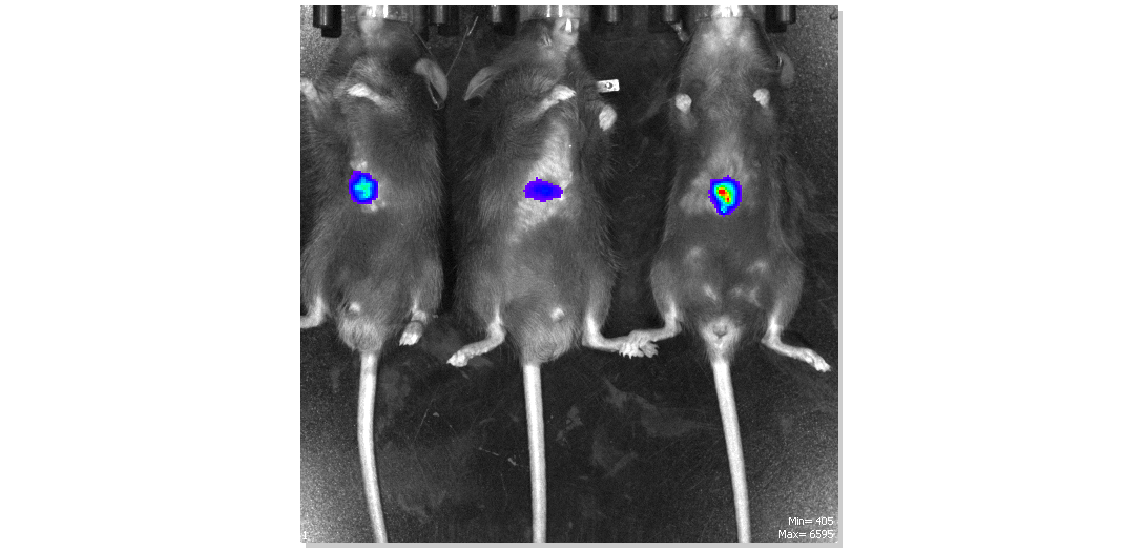

Supplement: Supplementary file 5 — Source data Fig. 3 [file 44321_2026_424_MOESM5_ESM.zip › Figure 3 Source Data/Figure 3D/Day 0/20.tif]

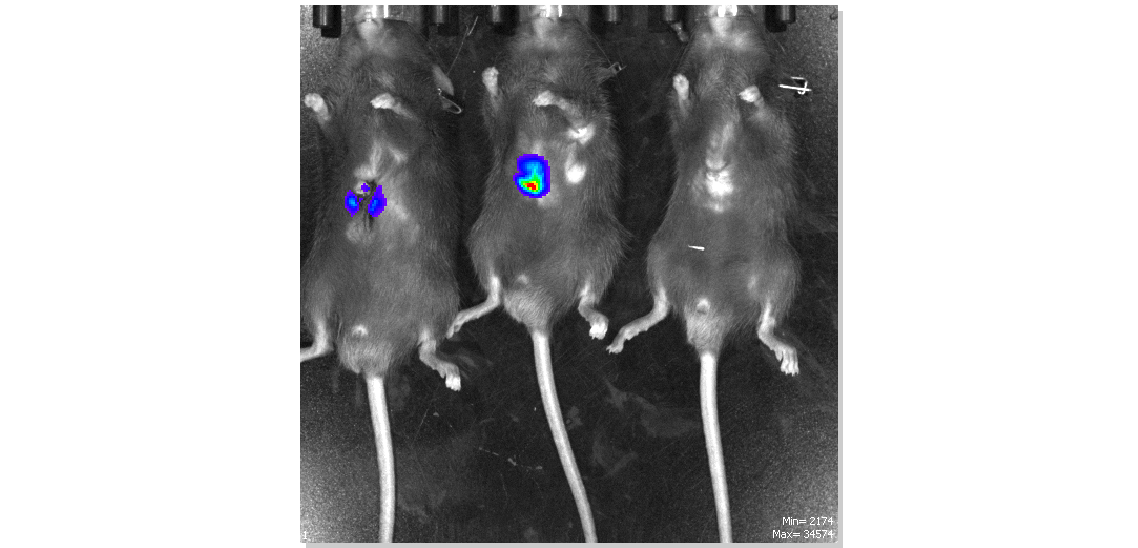

Supplement: Supplementary file 5 — Source data Fig. 3 [file 44321_2026_424_MOESM5_ESM.zip › Figure 3 Source Data/Figure 3D/Day 0/21.tif]

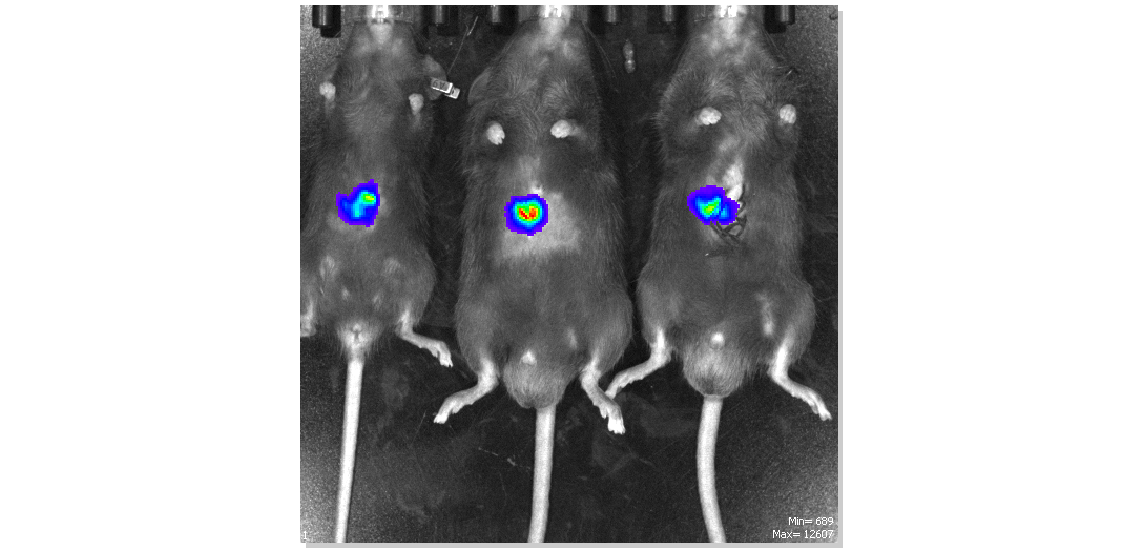

Supplement: Supplementary file 5 — Source data Fig. 3 [file 44321_2026_424_MOESM5_ESM.zip › Figure 3 Source Data/Figure 3D/Day 0/23.tif]

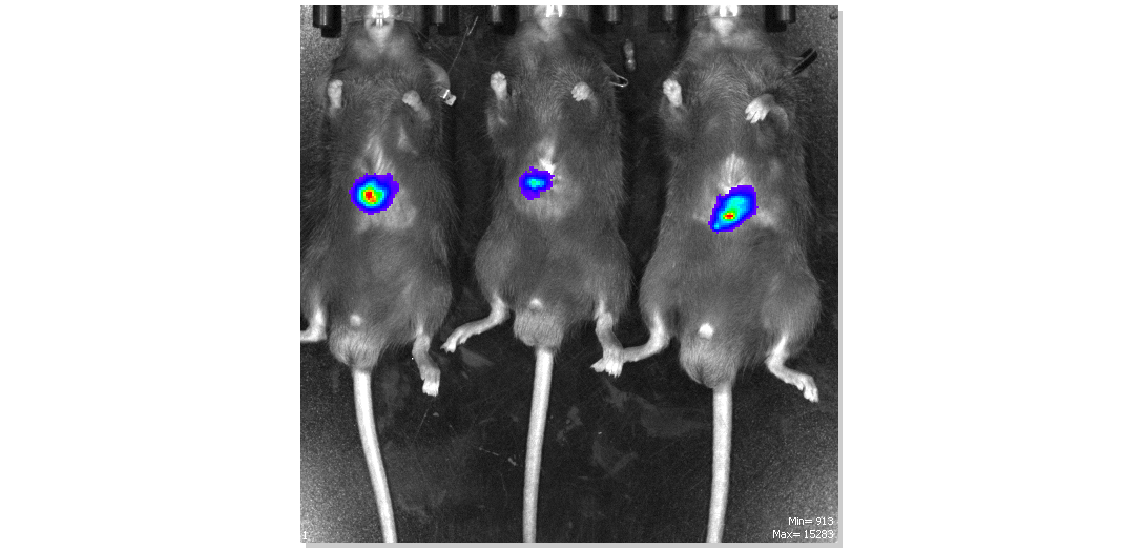

Supplement: Supplementary file 5 — Source data Fig. 3 [file 44321_2026_424_MOESM5_ESM.zip › Figure 3 Source Data/Figure 3D/Day 0/22.tif]

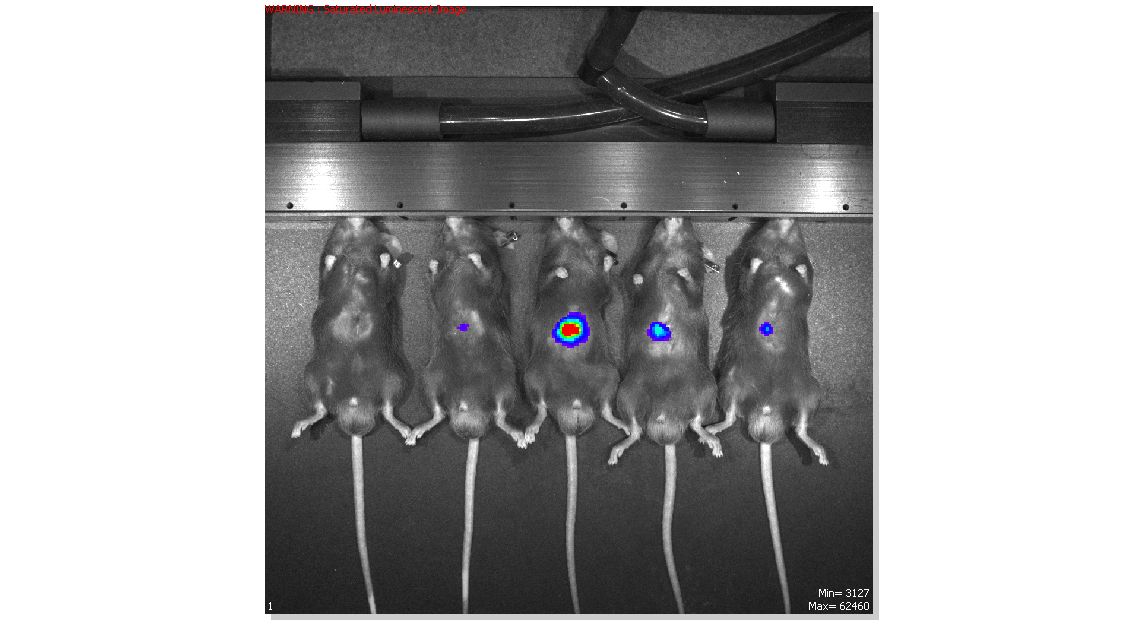

Supplement: Supplementary file 5 — Source data Fig. 3 [file 44321_2026_424_MOESM5_ESM.zip › Figure 3 Source Data/Figure 3D/Day 7/MICB-1-YHH.tif]

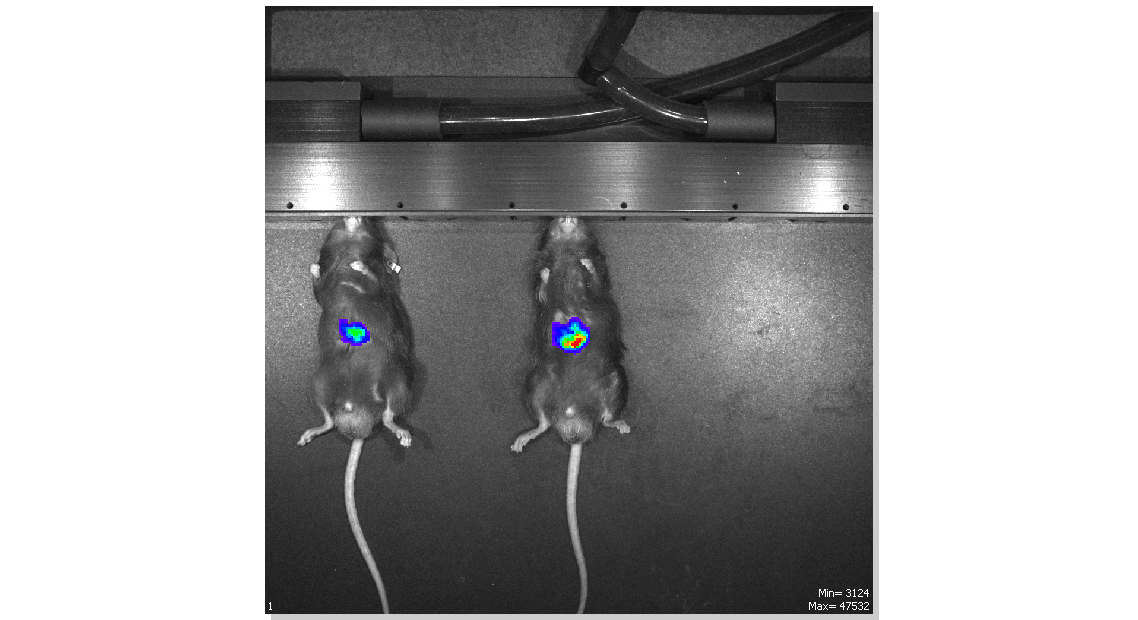

Supplement: Supplementary file 5 — Source data Fig. 3 [file 44321_2026_424_MOESM5_ESM.zip › Figure 3 Source Data/Figure 3D/Day 7/OMV-2-YHH.tif]

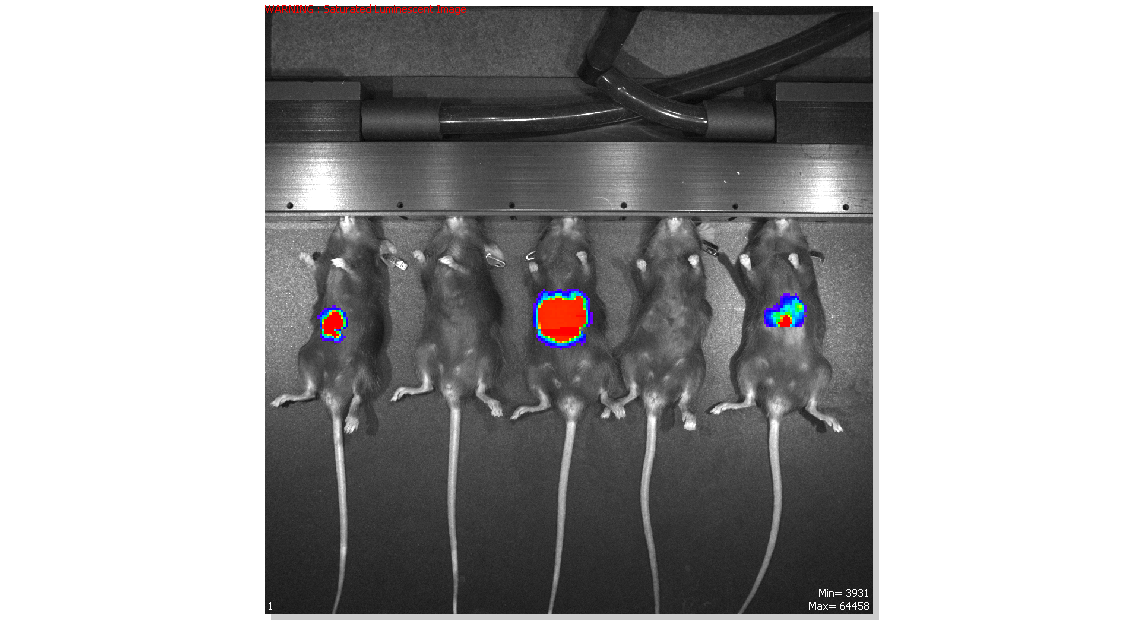

Supplement: Supplementary file 5 — Source data Fig. 3 [file 44321_2026_424_MOESM5_ESM.zip › Figure 3 Source Data/Figure 3D/Day 7/lfz-1.tif]

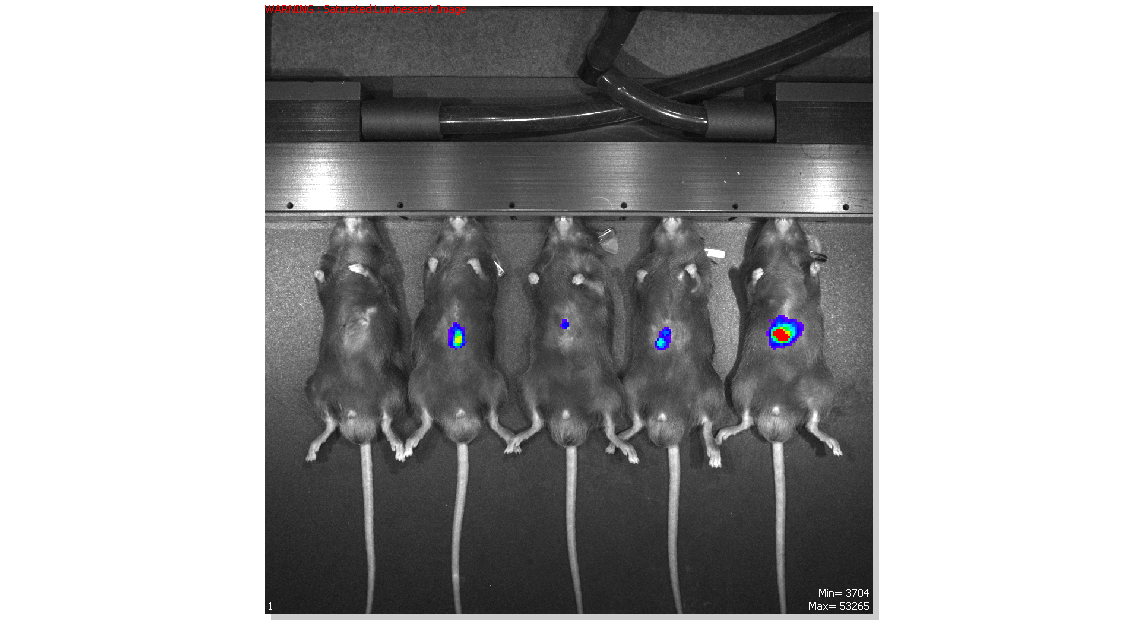

Supplement: Supplementary file 5 — Source data Fig. 3 [file 44321_2026_424_MOESM5_ESM.zip › Figure 3 Source Data/Figure 3D/Day 7/OMV-1-YHH.tif]

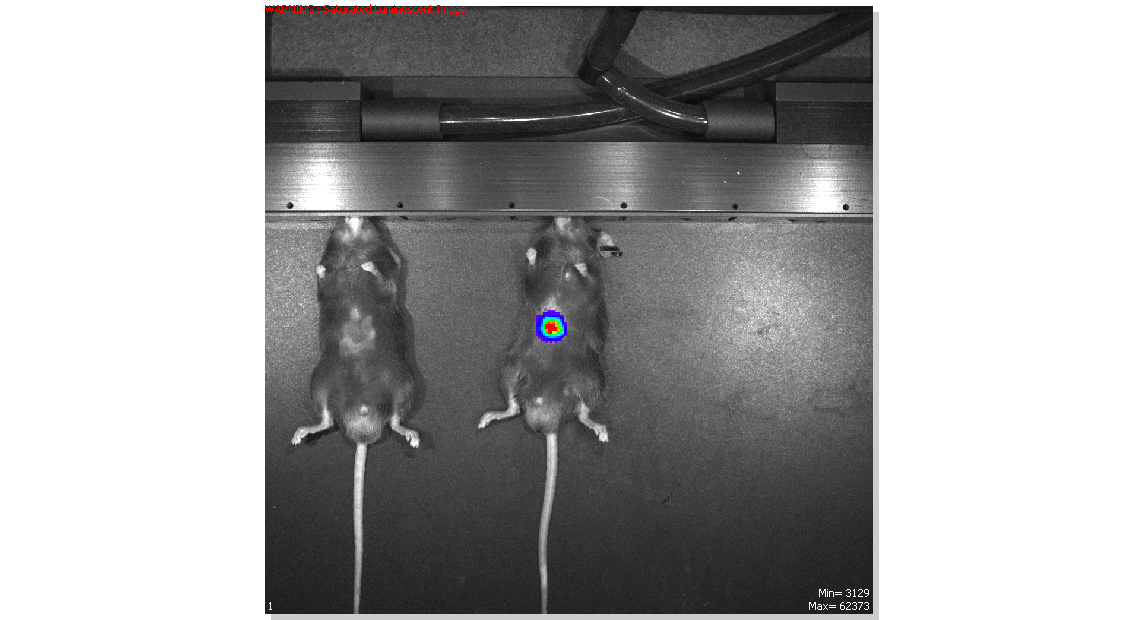

Supplement: Supplementary file 5 — Source data Fig. 3 [file 44321_2026_424_MOESM5_ESM.zip › Figure 3 Source Data/Figure 3D/Day 7/MICB-2-YHH.tif]

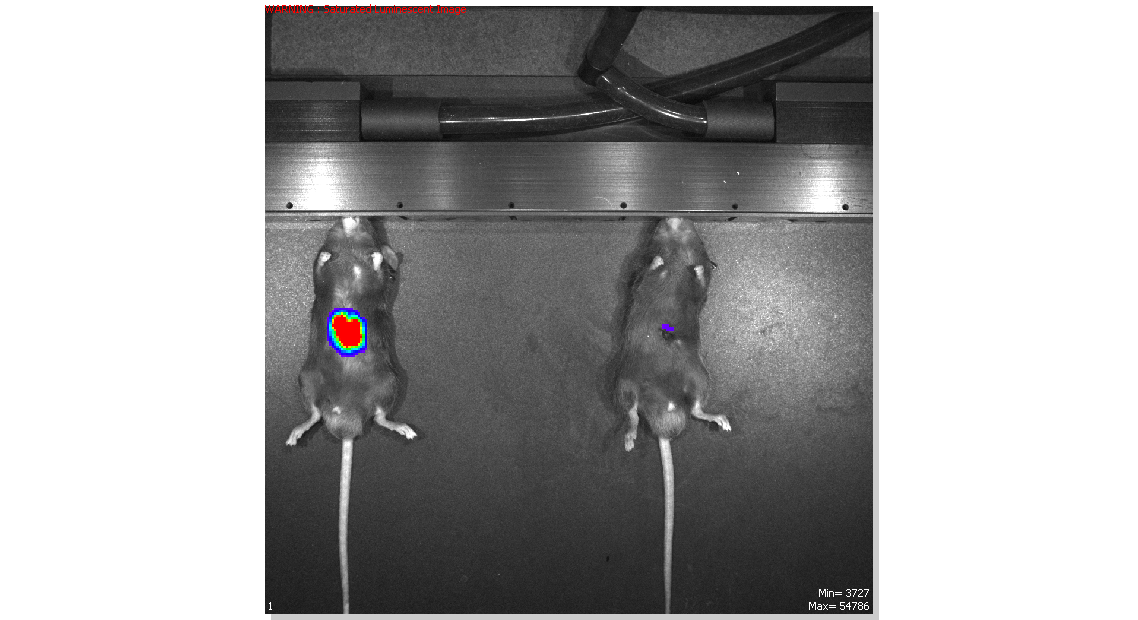

Supplement: Supplementary file 5 — Source data Fig. 3 [file 44321_2026_424_MOESM5_ESM.zip › Figure 3 Source Data/Figure 3D/Day 7/NFFC-2-YHH.tif]

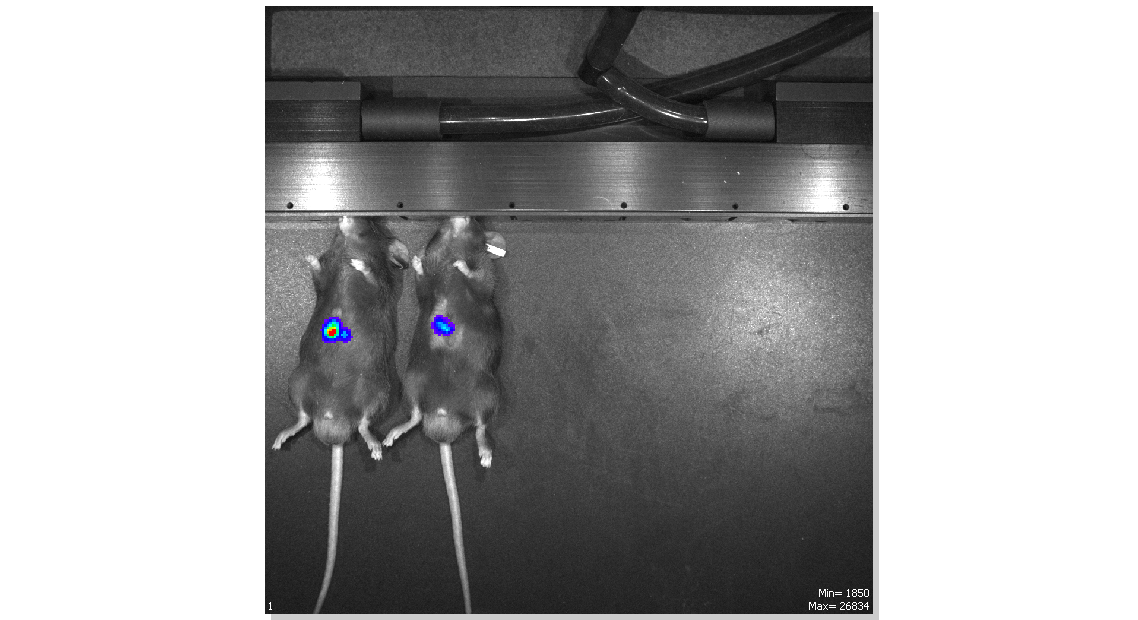

Supplement: Supplementary file 5 — Source data Fig. 3 [file 44321_2026_424_MOESM5_ESM.zip › Figure 3 Source Data/Figure 3D/Day 7/PBS-2-YHH.tif]

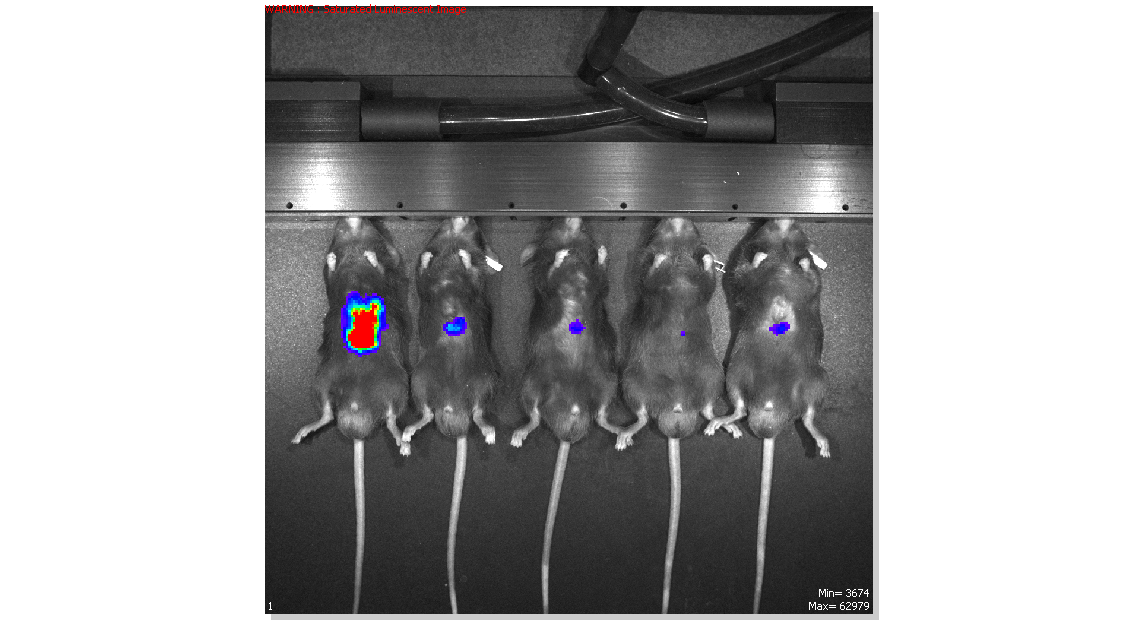

Supplement: Supplementary file 5 — Source data Fig. 3 [file 44321_2026_424_MOESM5_ESM.zip › Figure 3 Source Data/Figure 3D/Day 7/NFFC-MICB-1-YHH.tif]

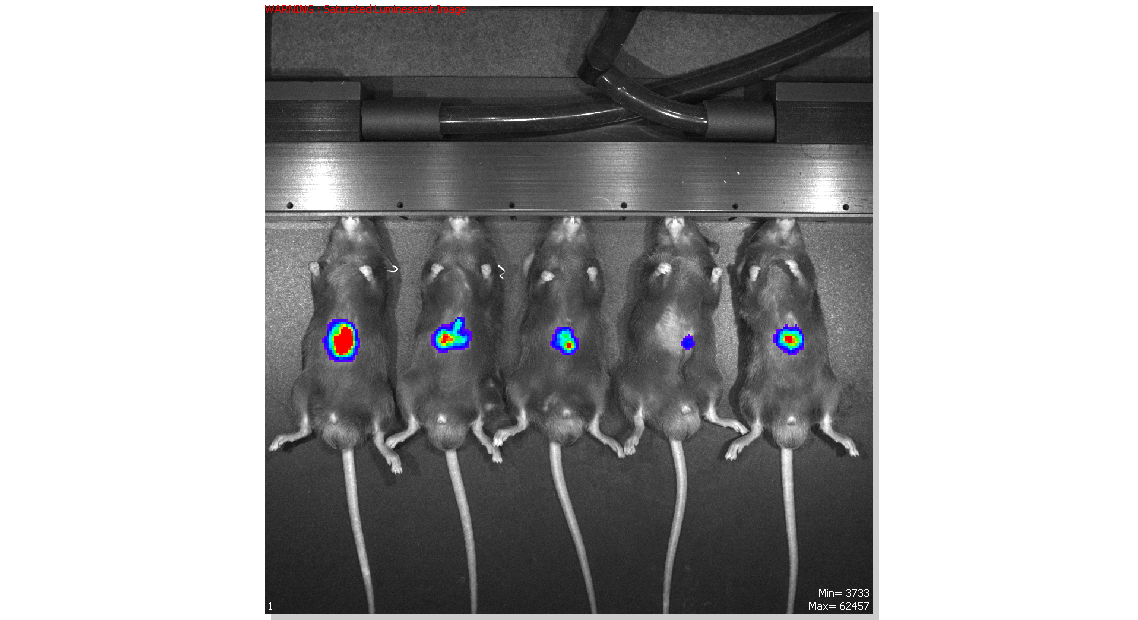

Supplement: Supplementary file 5 — Source data Fig. 3 [file 44321_2026_424_MOESM5_ESM.zip › Figure 3 Source Data/Figure 3D/Day 7/PBS-1-YHH.tif]

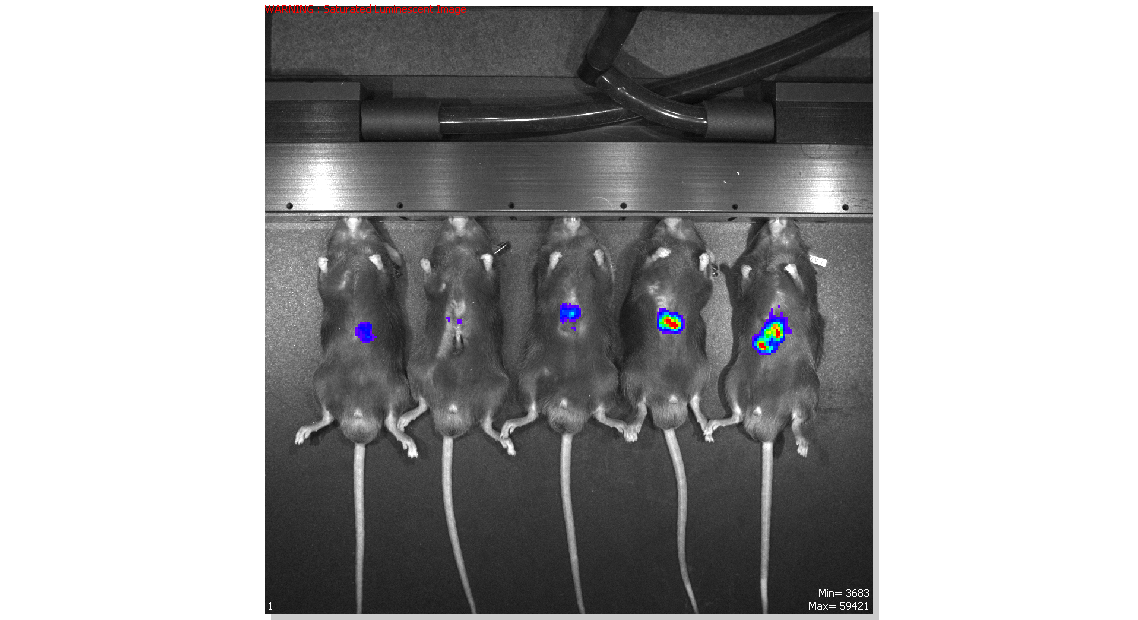

Supplement: Supplementary file 5 — Source data Fig. 3 [file 44321_2026_424_MOESM5_ESM.zip › Figure 3 Source Data/Figure 3D/Day 7/NFFC-1-YHH.tif]

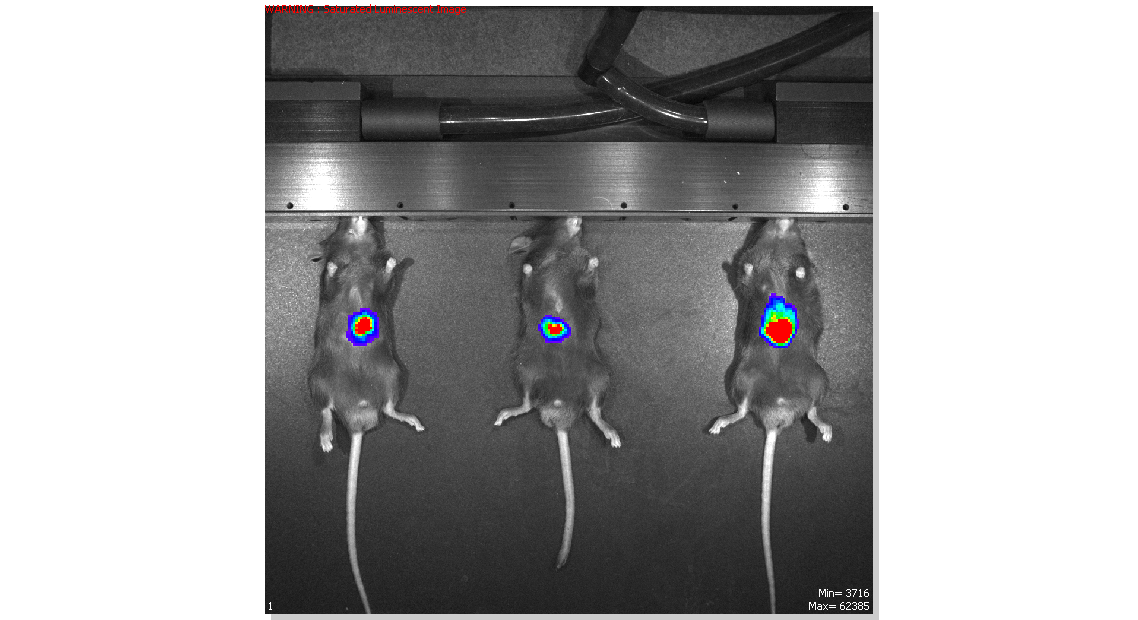

Supplement: Supplementary file 5 — Source data Fig. 3 [file 44321_2026_424_MOESM5_ESM.zip › Figure 3 Source Data/Figure 3D/Day 7/LLFZ-2.tif]

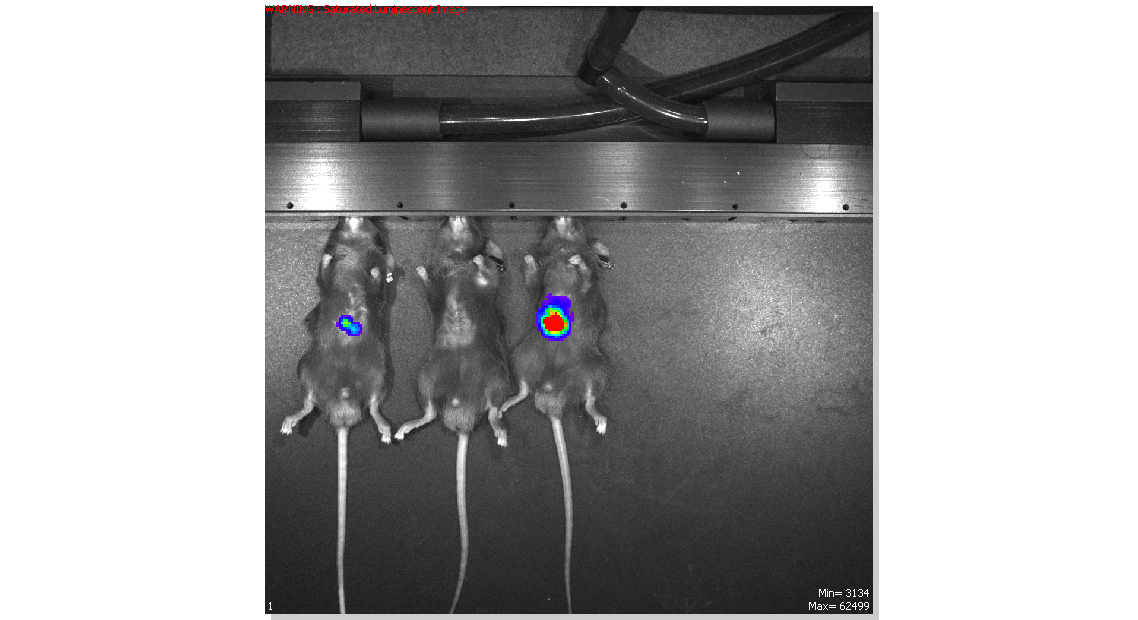

Supplement: Supplementary file 5 — Source data Fig. 3 [file 44321_2026_424_MOESM5_ESM.zip › Figure 3 Source Data/Figure 3D/Day 7/LLFZ-3.tif]

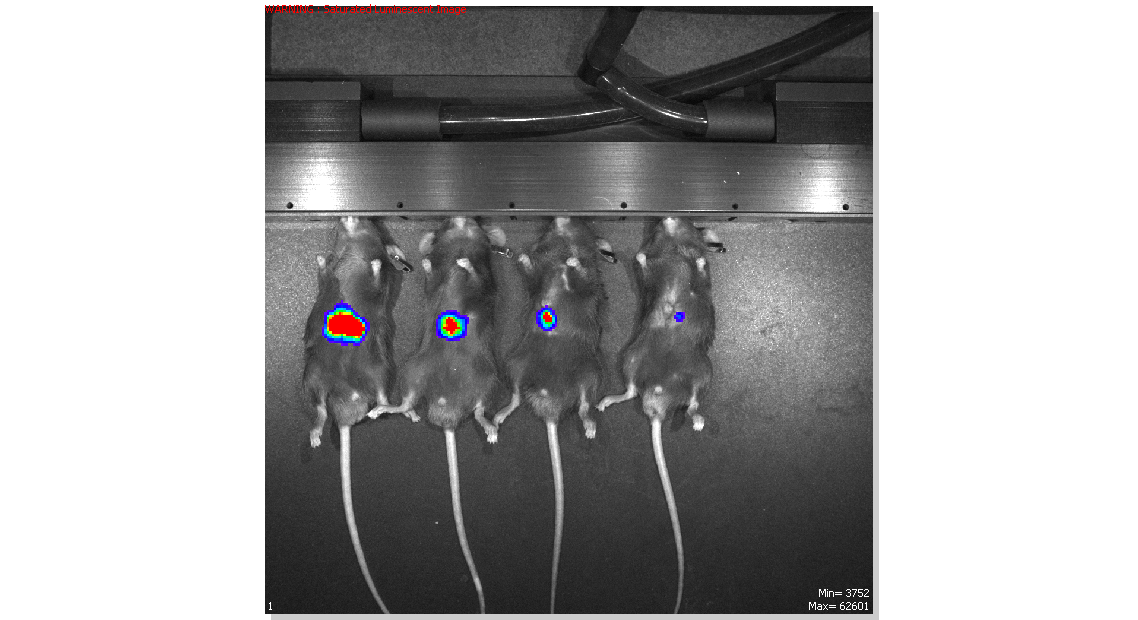

Supplement: Supplementary file 5 — Source data Fig. 3 [file 44321_2026_424_MOESM5_ESM.zip › Figure 3 Source Data/Figure 3D/Day 7/LLFZ-1.tif]

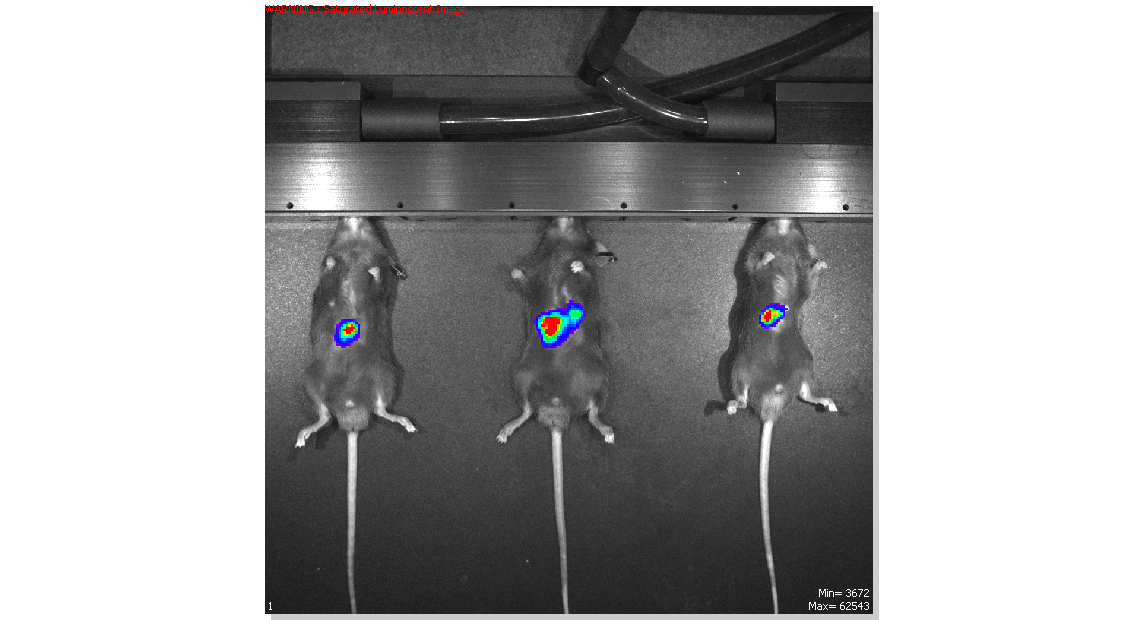

Supplement: Supplementary file 5 — Source data Fig. 3 [file 44321_2026_424_MOESM5_ESM.zip › Figure 3 Source Data/Figure 3D/Day 7/LLFZ-4.tif]

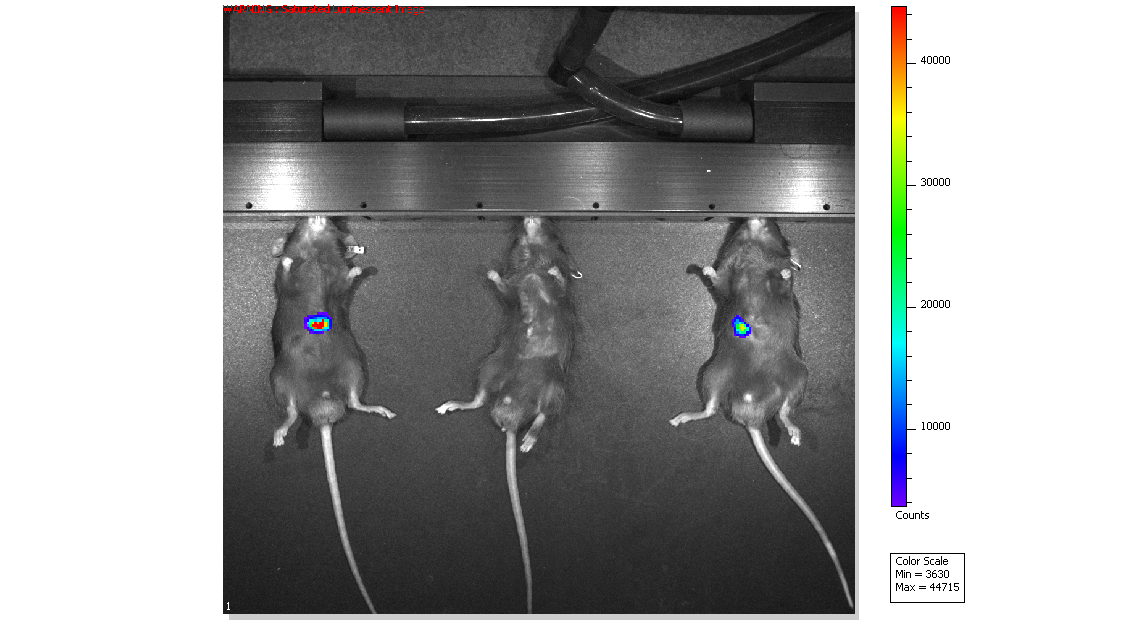

Supplement: Supplementary file 5 — Source data Fig. 3 [file 44321_2026_424_MOESM5_ESM.zip › Figure 3 Source Data/Figure 3D/Day 7/LLFZ-5.tif]

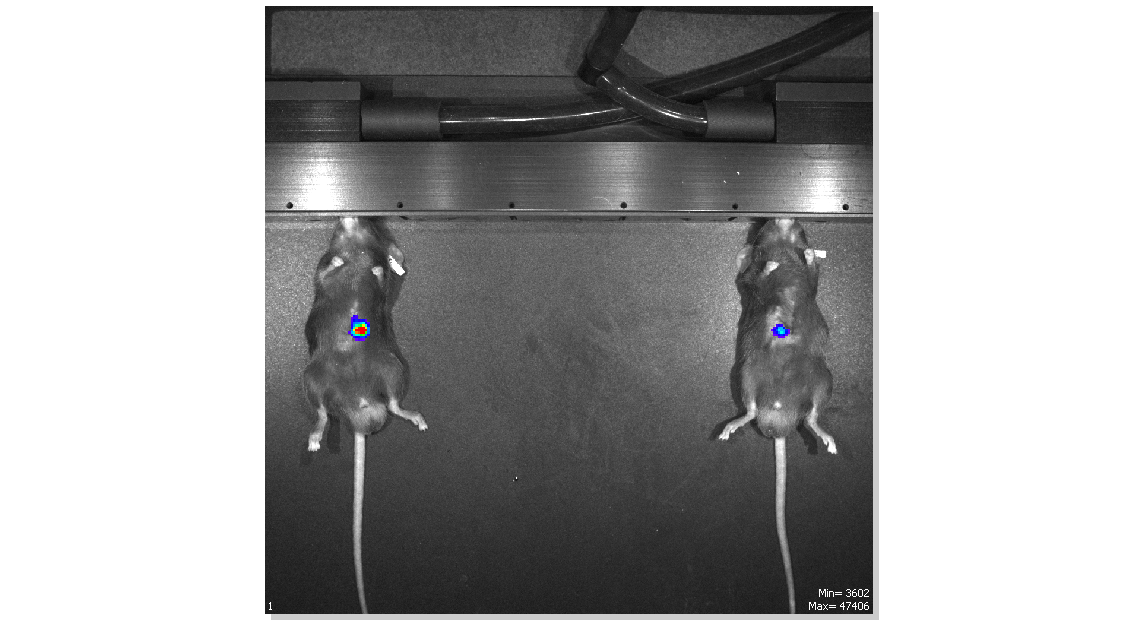

Supplement: Supplementary file 5 — Source data Fig. 3 [file 44321_2026_424_MOESM5_ESM.zip › Figure 3 Source Data/Figure 3D/Day 7/NFFC-MICB-2-YHH.tif]

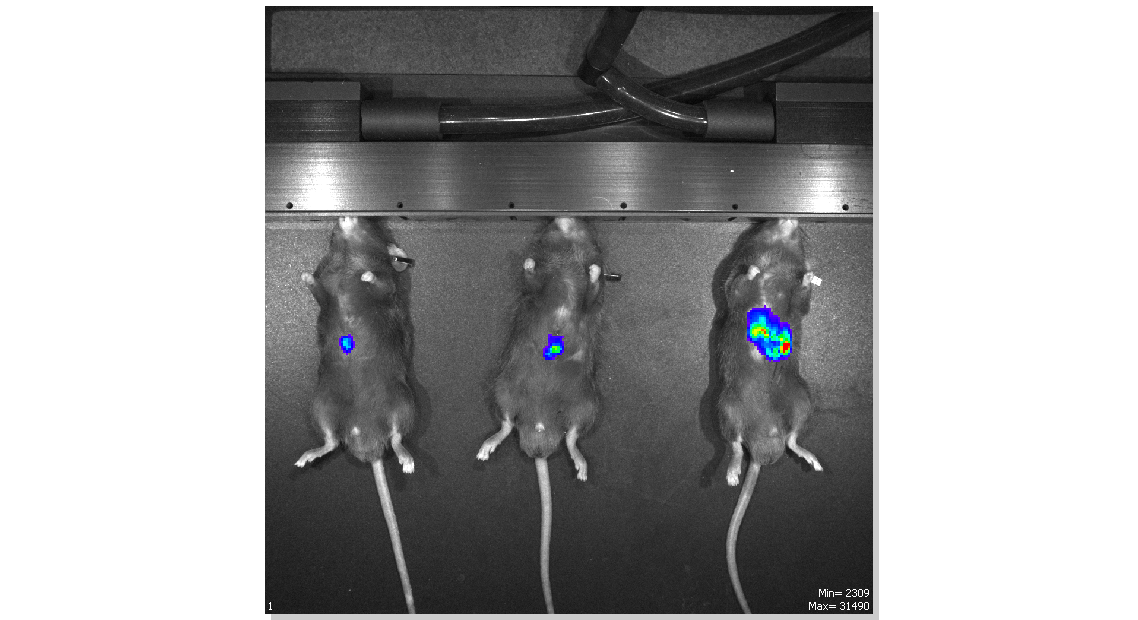

Supplement: Supplementary file 5 — Source data Fig. 3 [file 44321_2026_424_MOESM5_ESM.zip › Figure 3 Source Data/Figure 3D/Day 7/LLFZ-7.tif]

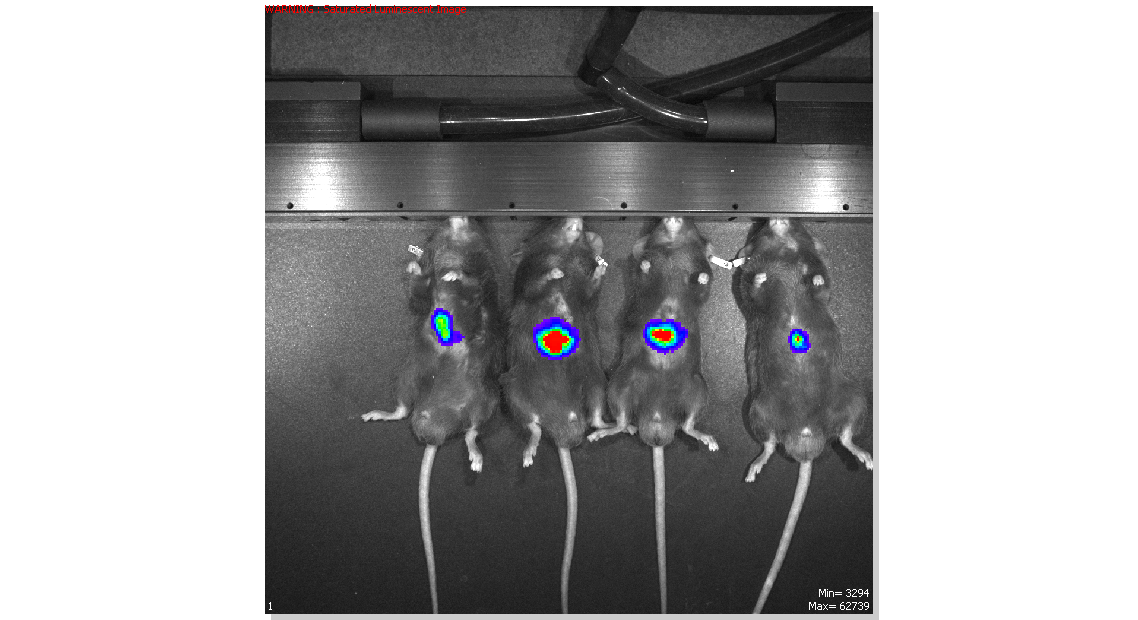

Supplement: Supplementary file 5 — Source data Fig. 3 [file 44321_2026_424_MOESM5_ESM.zip › Figure 3 Source Data/Figure 3D/Day 7/LLFZ-6.tif]

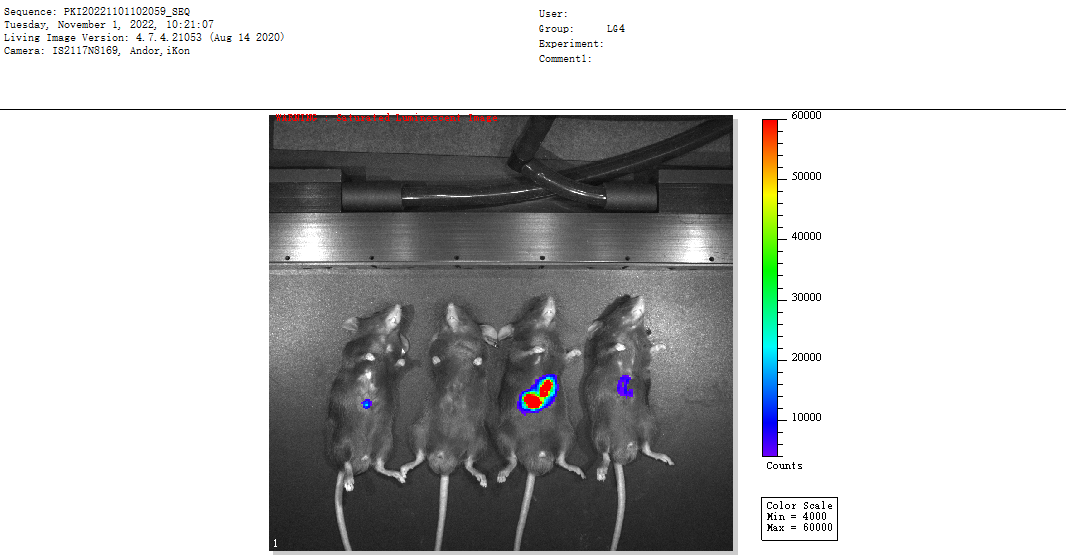

Supplement: Supplementary file 5 — Source data Fig. 3 [file 44321_2026_424_MOESM5_ESM.zip › Figure 3 Source Data/Figure 3D/Day 21/G4.tif]

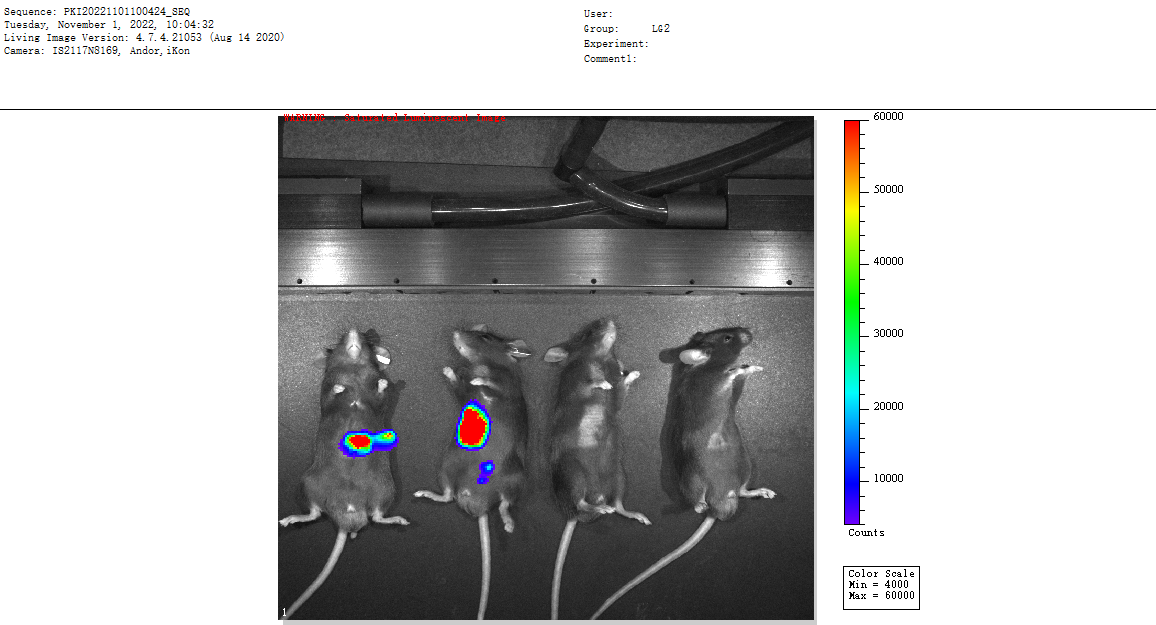

Supplement: Supplementary file 5 — Source data Fig. 3 [file 44321_2026_424_MOESM5_ESM.zip › Figure 3 Source Data/Figure 3D/Day 21/G2.tif]

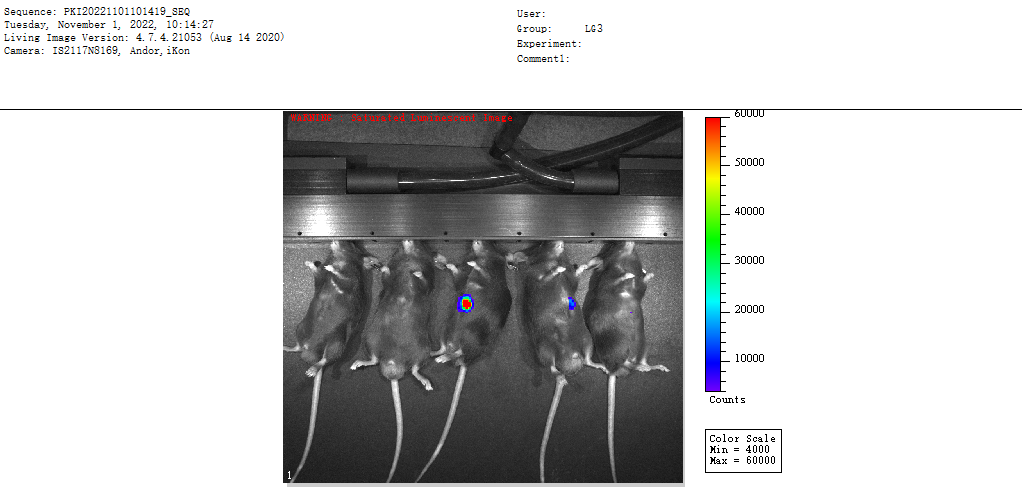

Supplement: Supplementary file 5 — Source data Fig. 3 [file 44321_2026_424_MOESM5_ESM.zip › Figure 3 Source Data/Figure 3D/Day 21/G3.tif]

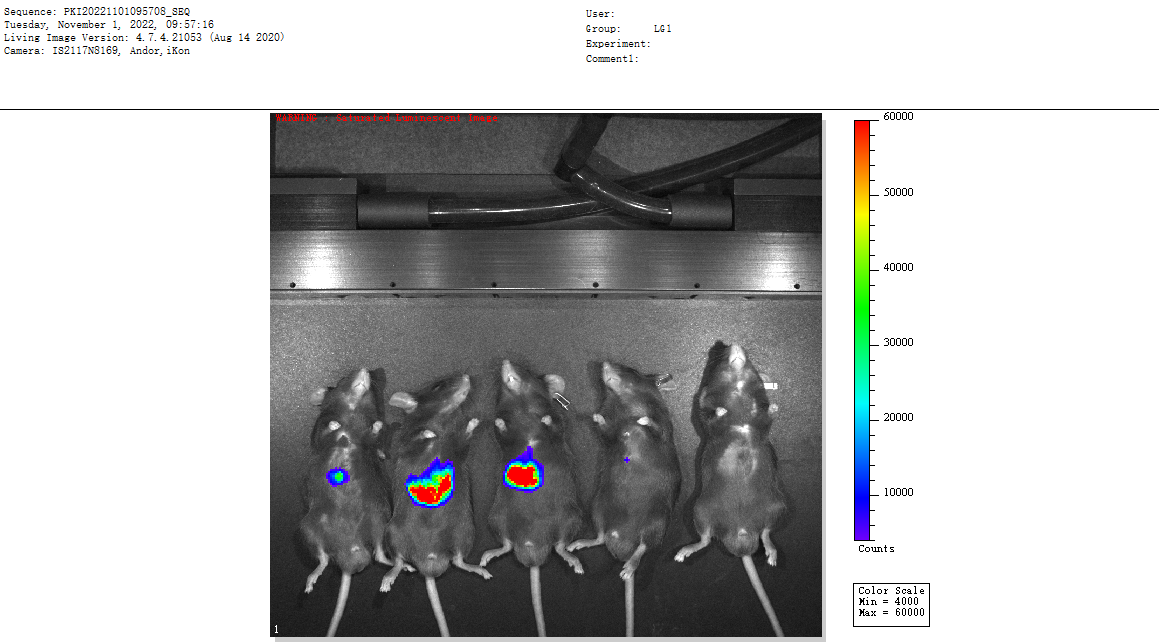

Supplement: Supplementary file 5 — Source data Fig. 3 [file 44321_2026_424_MOESM5_ESM.zip › Figure 3 Source Data/Figure 3D/Day 21/G1.tif]

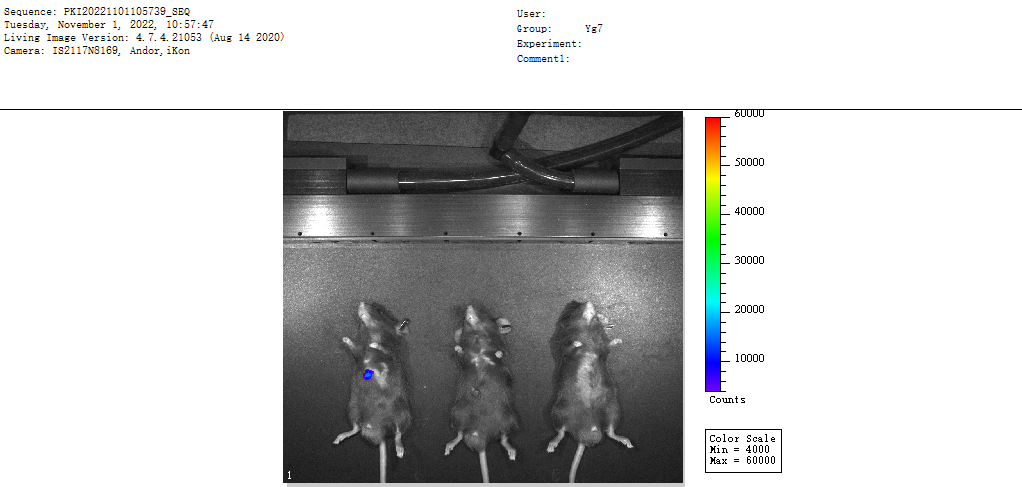

Supplement: Supplementary file 5 — Source data Fig. 3 [file 44321_2026_424_MOESM5_ESM.zip › Figure 3 Source Data/Figure 3D/Day 21/YG7.tif]

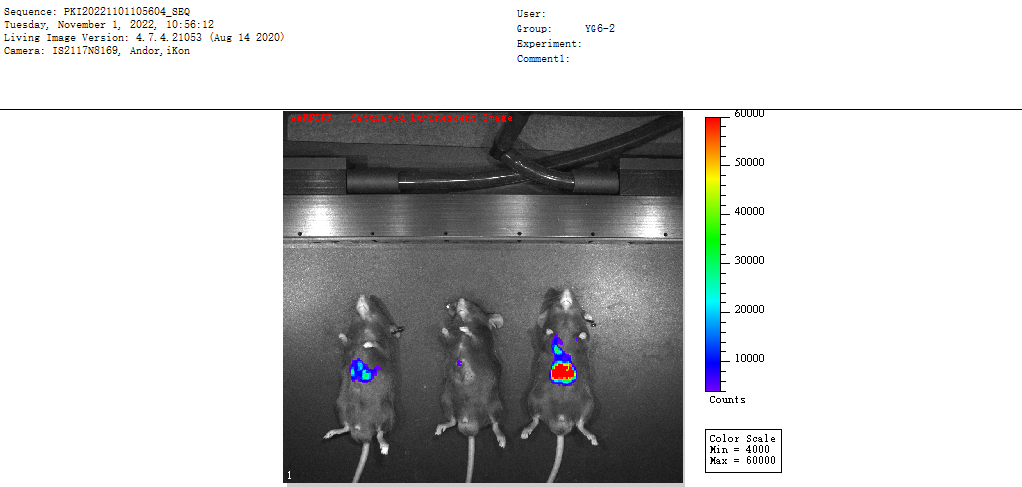

Supplement: Supplementary file 5 — Source data Fig. 3 [file 44321_2026_424_MOESM5_ESM.zip › Figure 3 Source Data/Figure 3D/Day 21/YG6.tif]

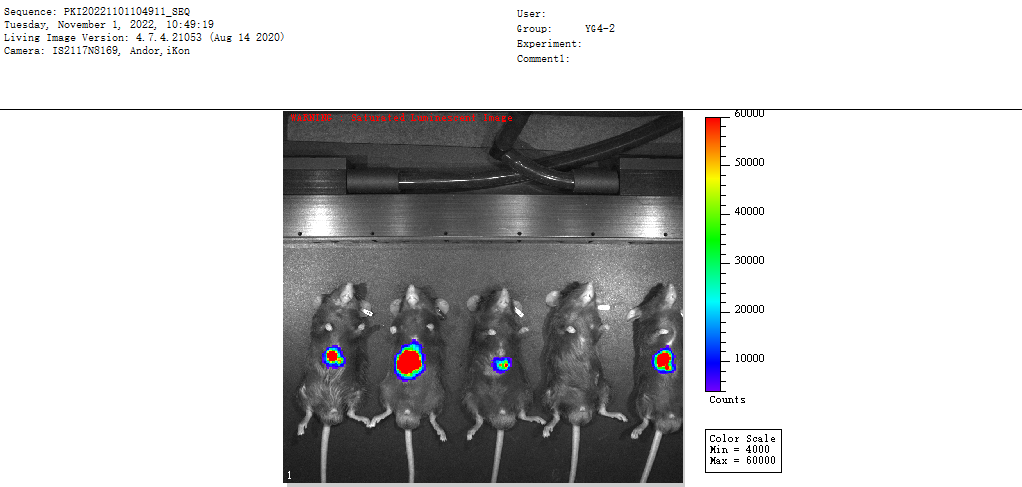

Supplement: Supplementary file 5 — Source data Fig. 3 [file 44321_2026_424_MOESM5_ESM.zip › Figure 3 Source Data/Figure 3D/Day 21/YG4.tif]

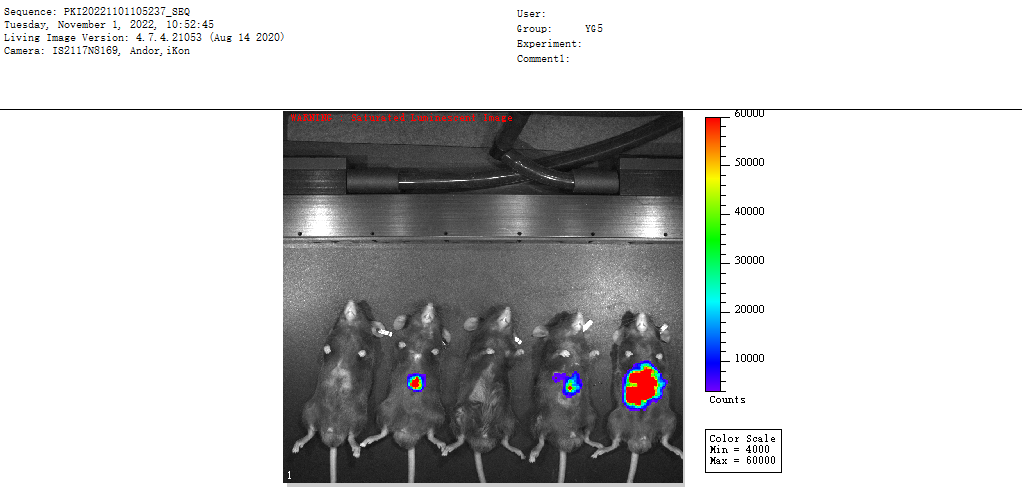

Supplement: Supplementary file 5 — Source data Fig. 3 [file 44321_2026_424_MOESM5_ESM.zip › Figure 3 Source Data/Figure 3D/Day 21/YG5.tif]

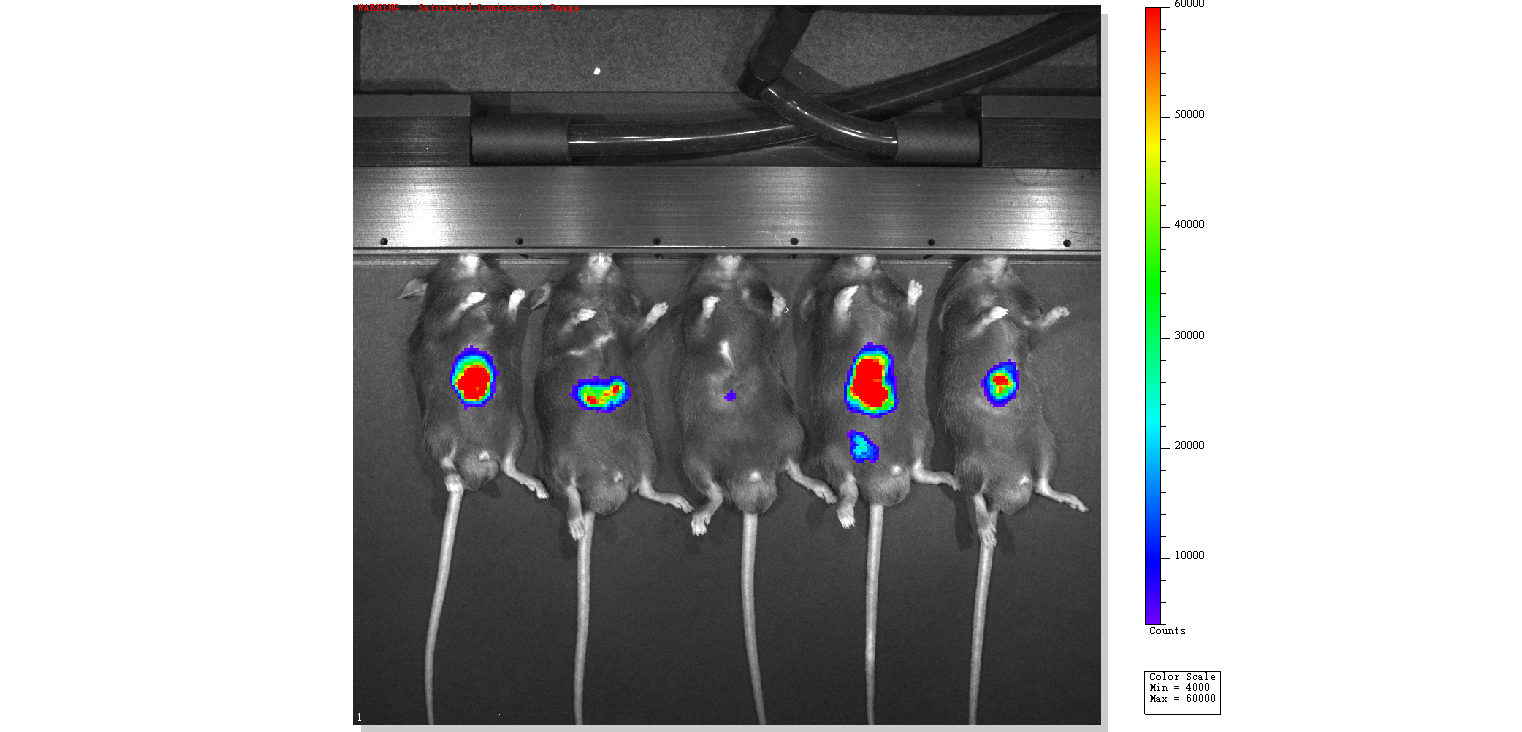

Supplement: Supplementary file 5 — Source data Fig. 3 [file 44321_2026_424_MOESM5_ESM.zip › Figure 3 Source Data/Figure 3D/Day 28/G4.tif]

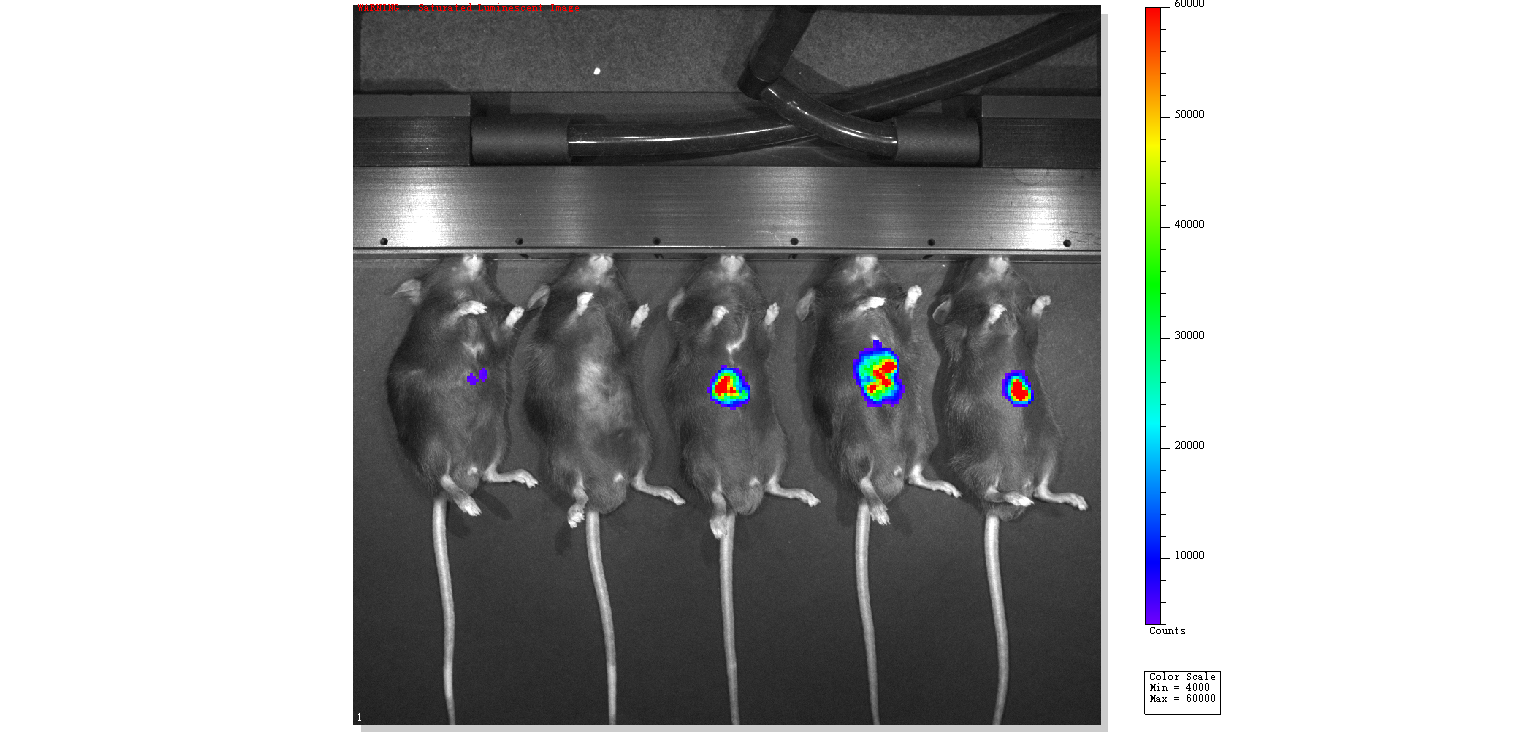

Supplement: Supplementary file 5 — Source data Fig. 3 [file 44321_2026_424_MOESM5_ESM.zip › Figure 3 Source Data/Figure 3D/Day 28/G5.tif]

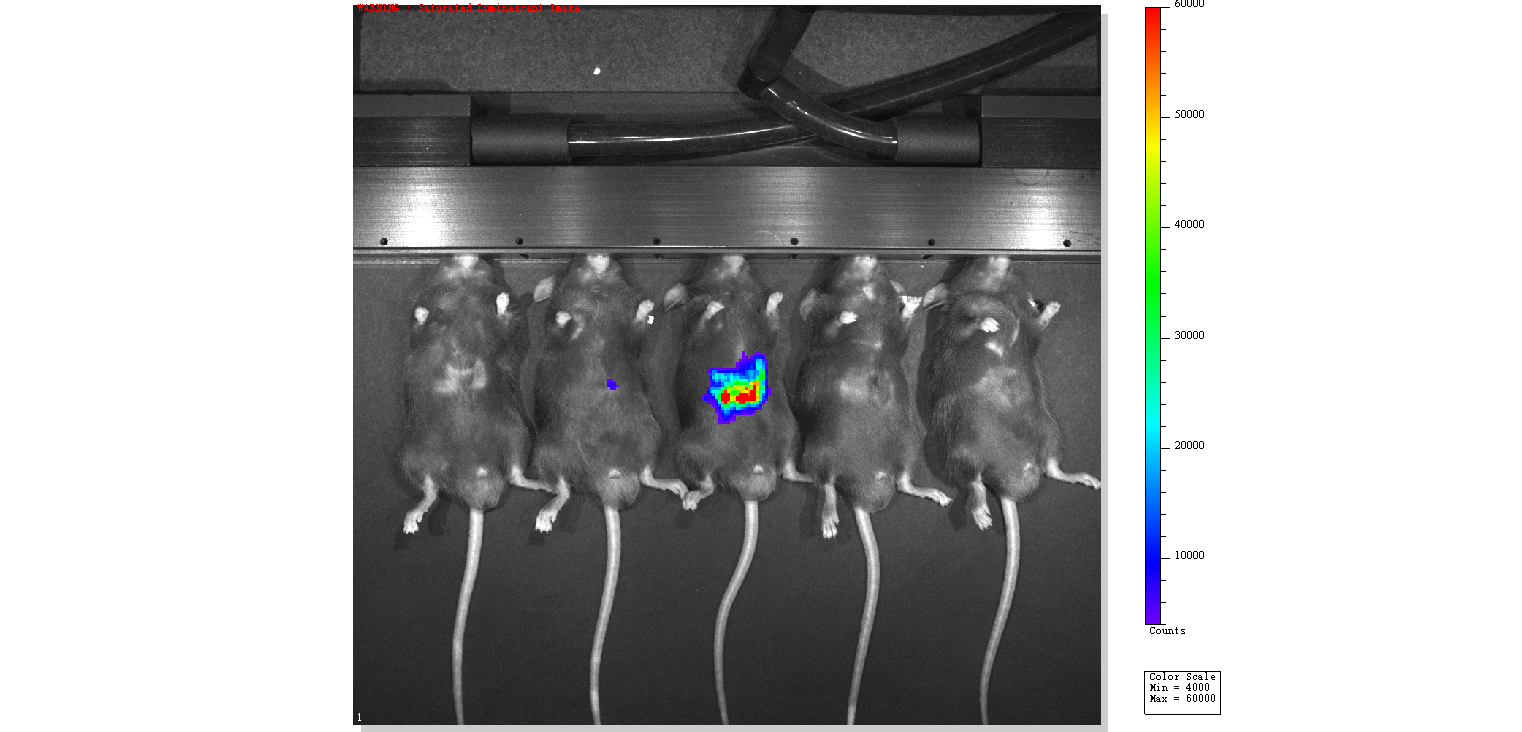

Supplement: Supplementary file 5 — Source data Fig. 3 [file 44321_2026_424_MOESM5_ESM.zip › Figure 3 Source Data/Figure 3D/Day 28/G6.tif]

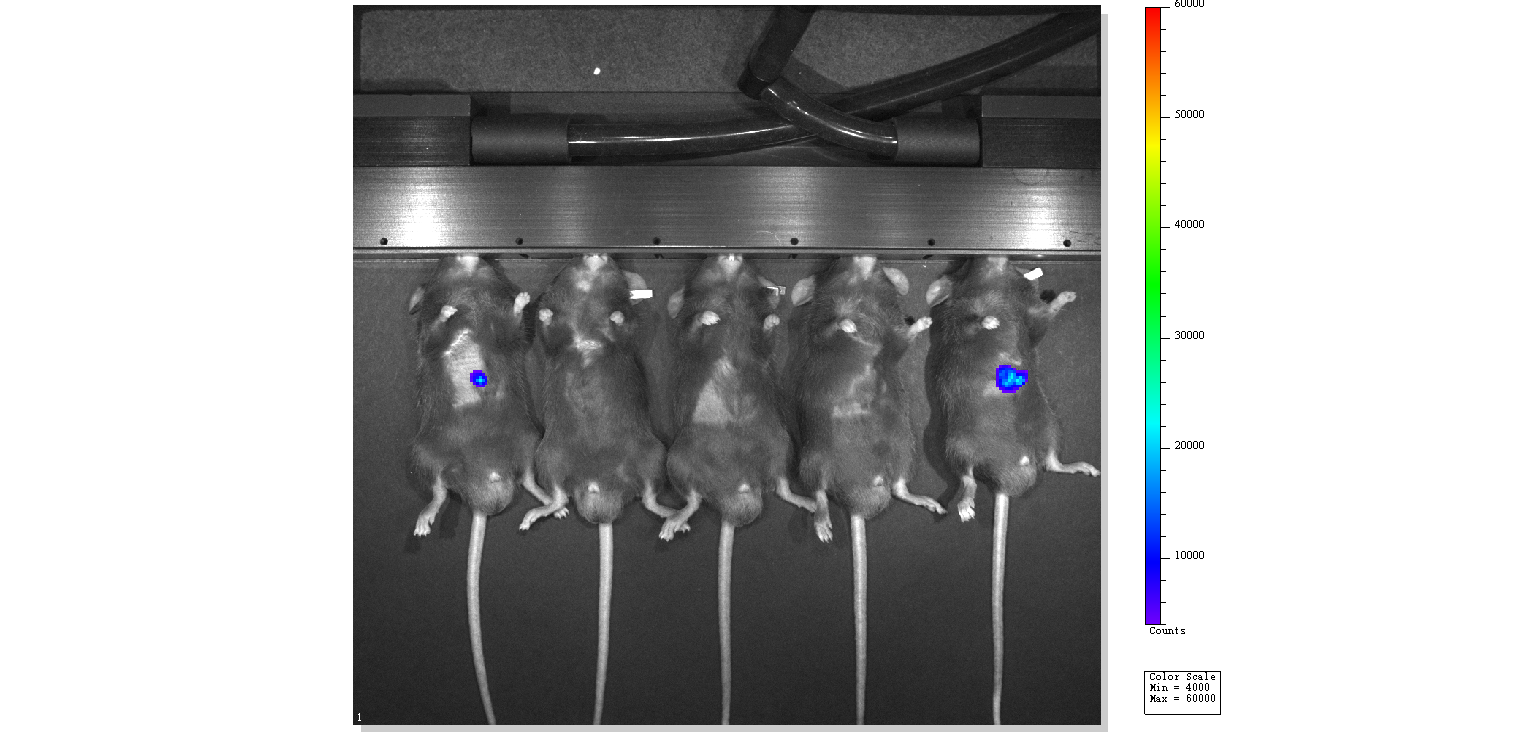

Supplement: Supplementary file 5 — Source data Fig. 3 [file 44321_2026_424_MOESM5_ESM.zip › Figure 3 Source Data/Figure 3D/Day 28/G2.tif]

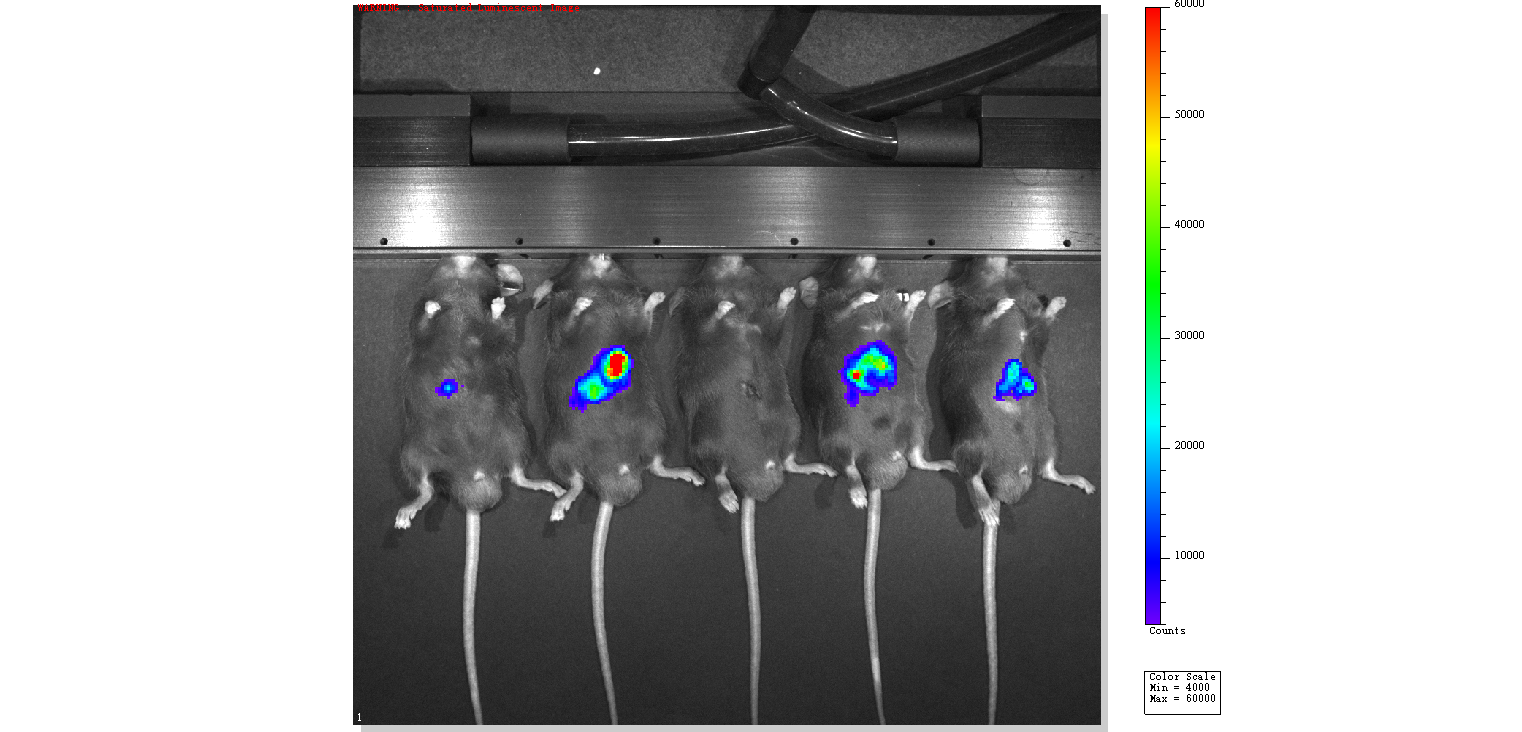

Supplement: Supplementary file 5 — Source data Fig. 3 [file 44321_2026_424_MOESM5_ESM.zip › Figure 3 Source Data/Figure 3D/Day 28/G3.tif]

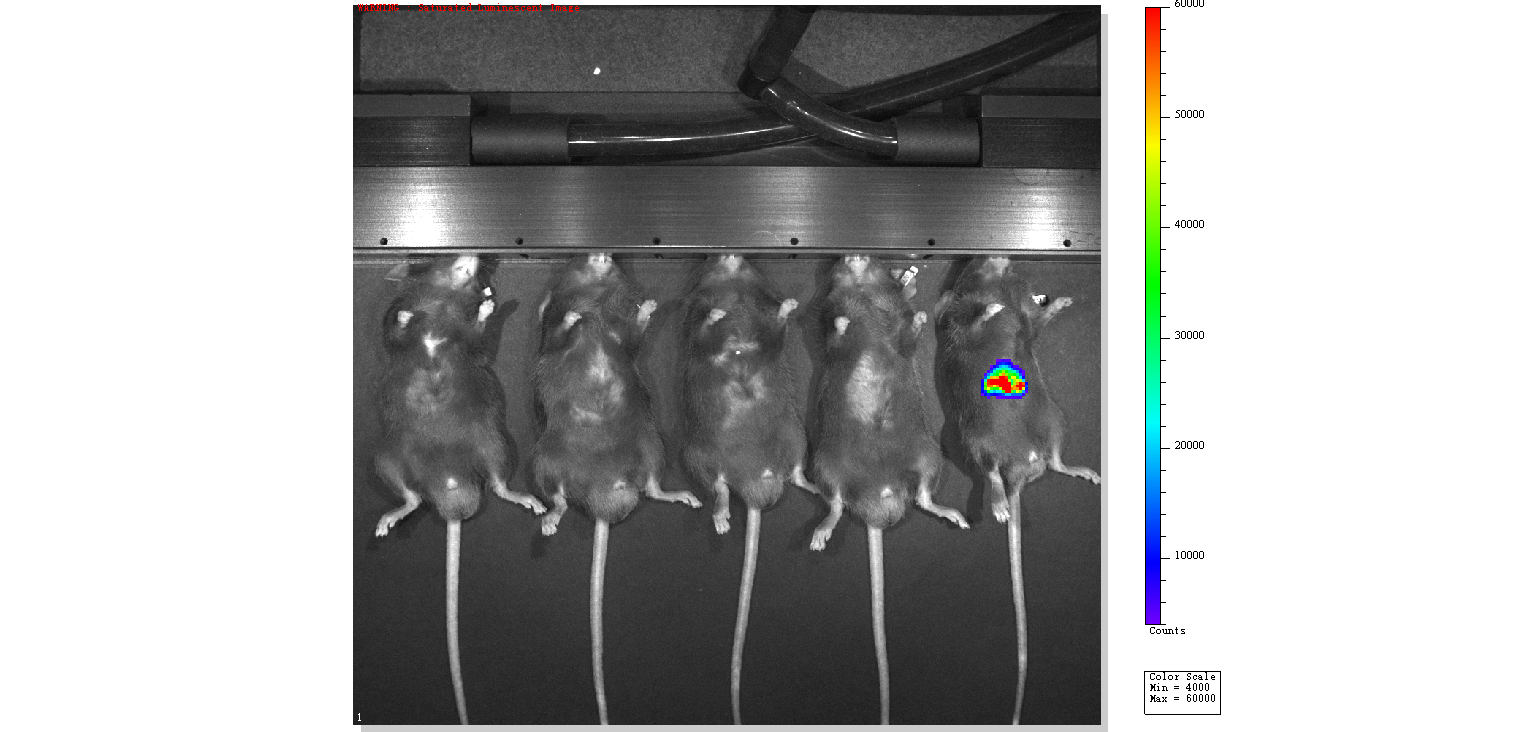

Supplement: Supplementary file 5 — Source data Fig. 3 [file 44321_2026_424_MOESM5_ESM.zip › Figure 3 Source Data/Figure 3D/Day 28/G1.tif]

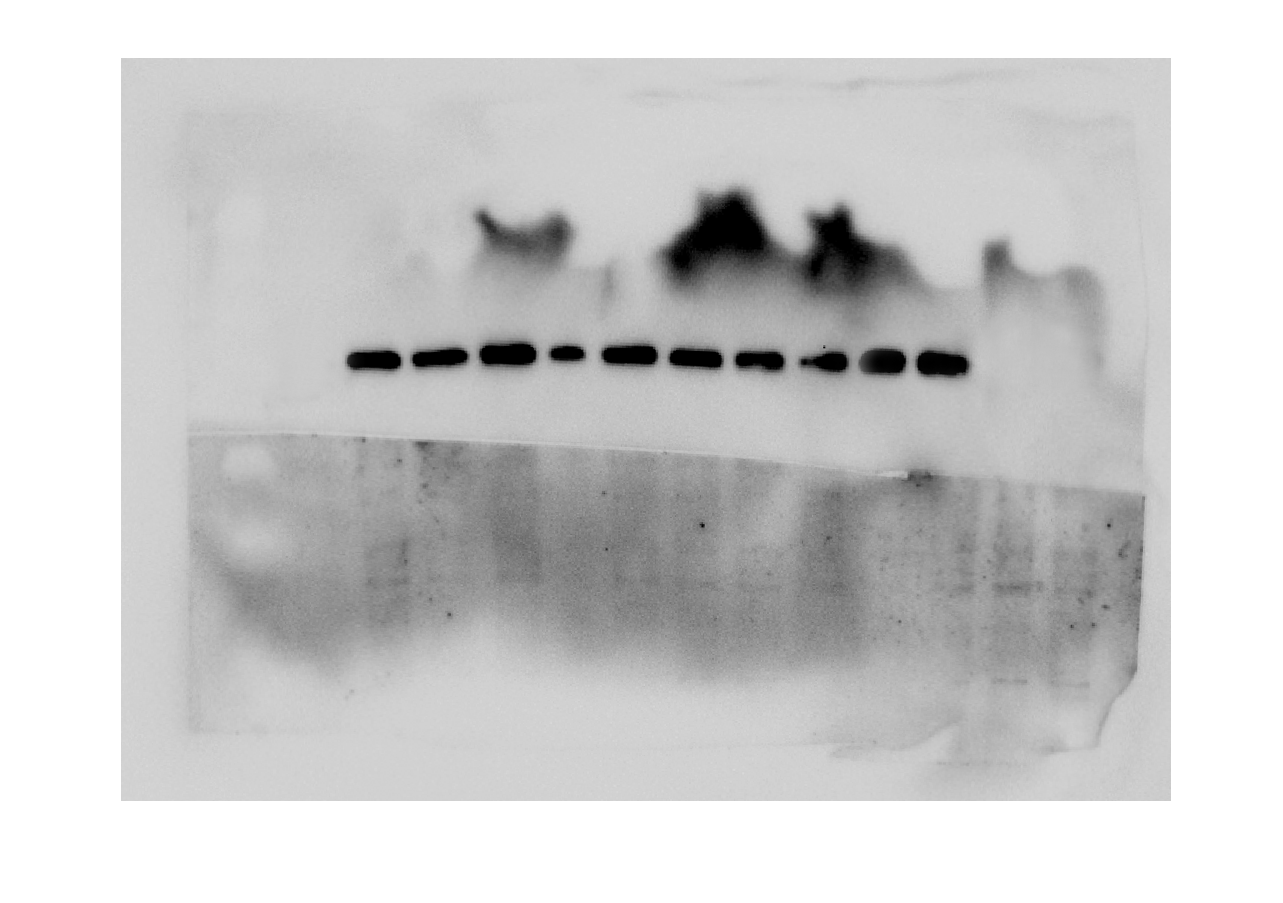

Supplement: Supplementary file 6 — Source data Fig. 4 [file 44321_2026_424_MOESM6_ESM.zip › Figure 4 Source Data/Figure 4E/GAPDH.tif]

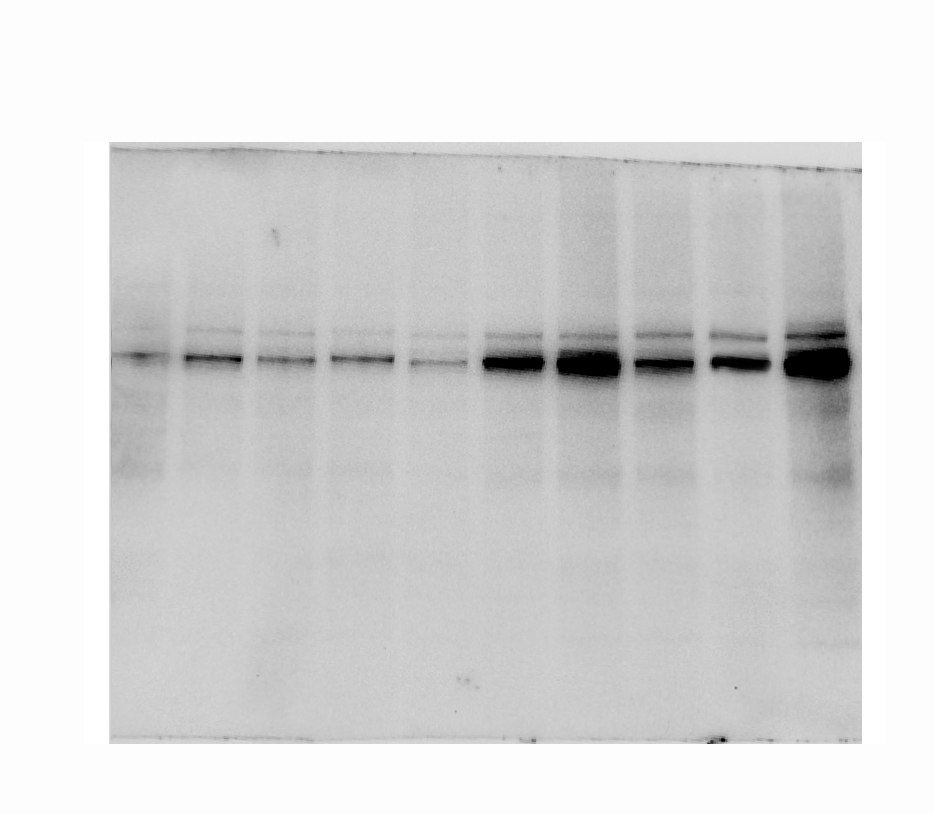

Supplement: Supplementary file 6 — Source data Fig. 4 [file 44321_2026_424_MOESM6_ESM.zip › Figure 4 Source Data/Figure 4E/N-GSDMD.tif]

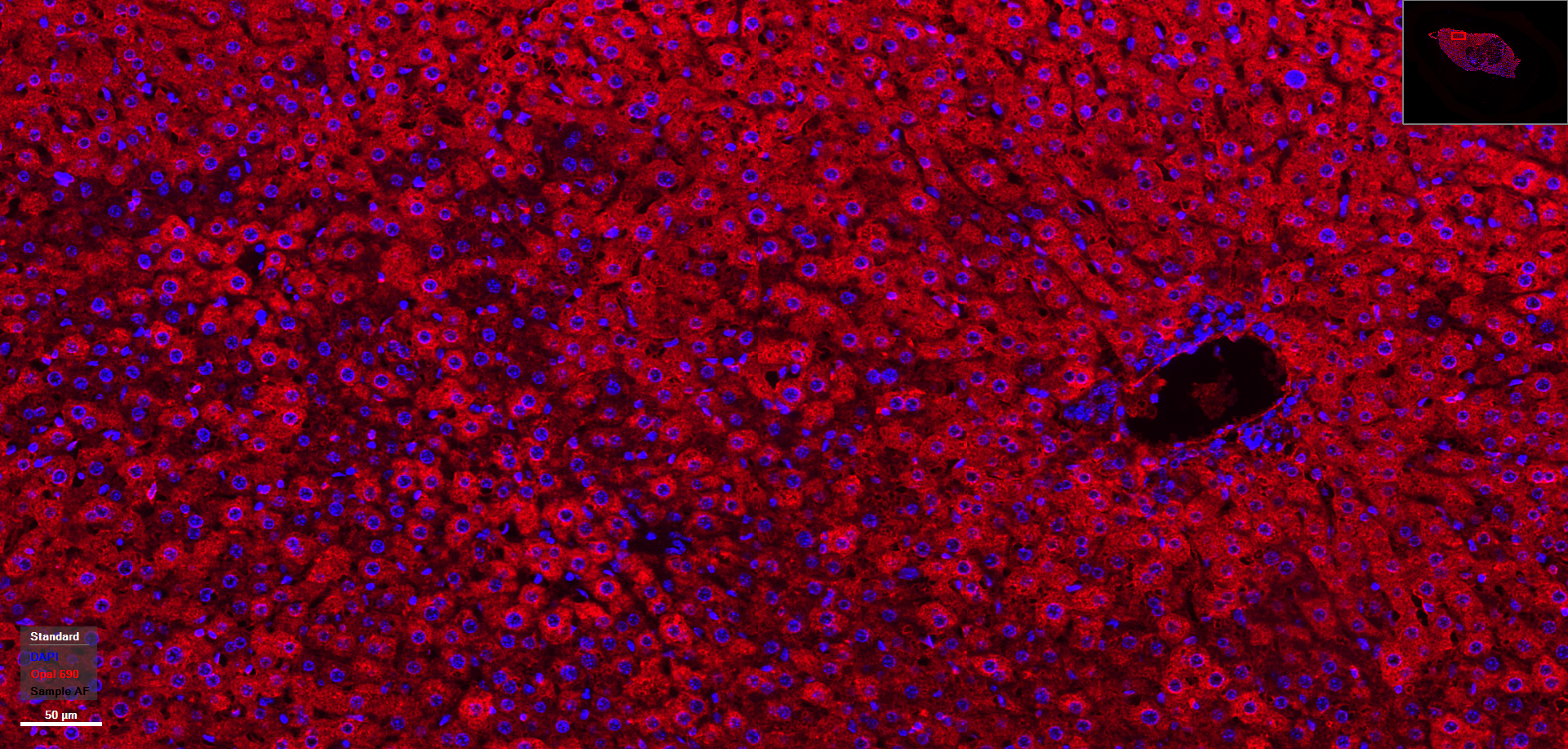

Supplement: Supplementary file 6 — Source data Fig. 4 [file 44321_2026_424_MOESM6_ESM.zip › Figure 4 Source Data/Figure 4A/Combined.tif]

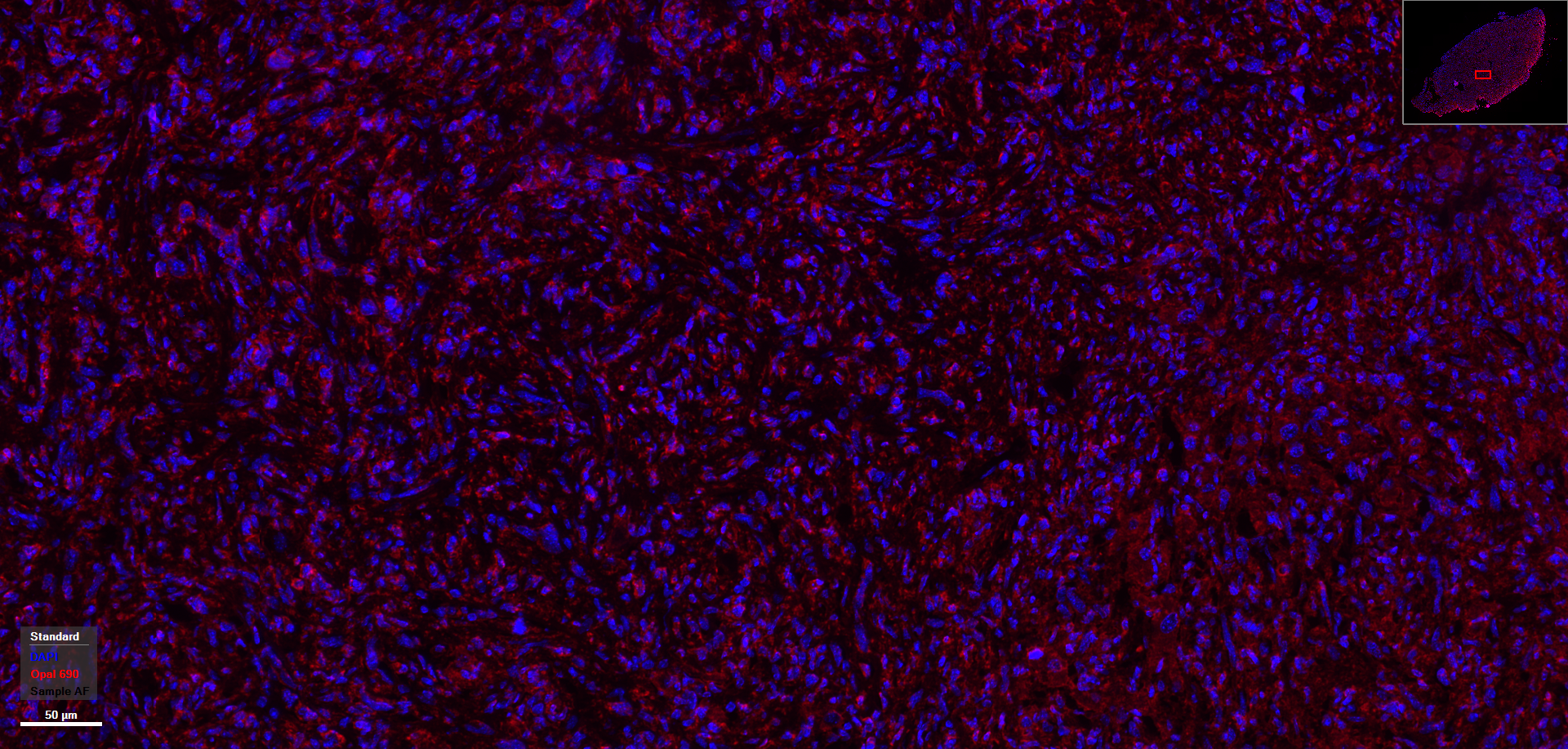

Supplement: Supplementary file 6 — Source data Fig. 4 [file 44321_2026_424_MOESM6_ESM.zip › Figure 4 Source Data/Figure 4A/CTRL.tif]

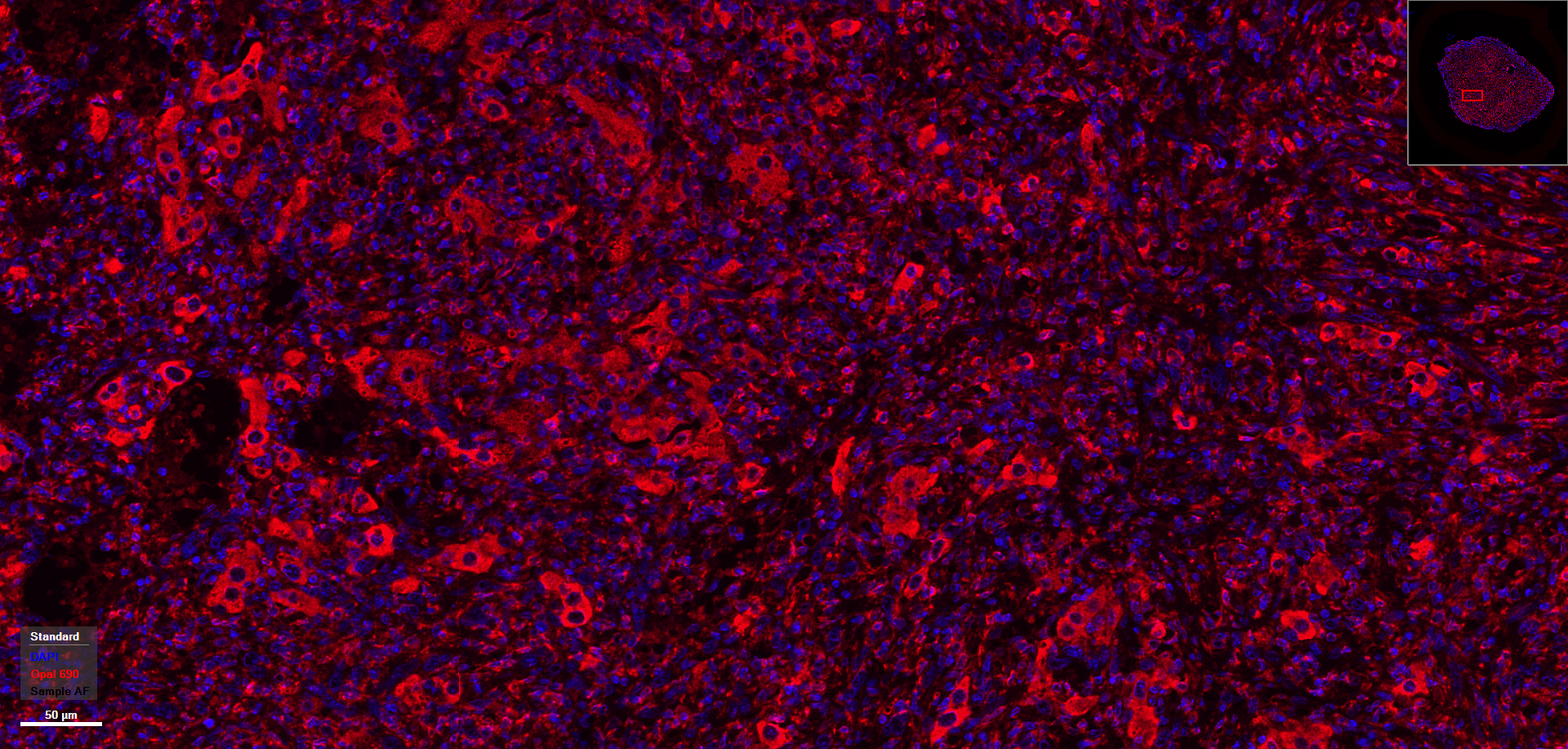

Supplement: Supplementary file 6 — Source data Fig. 4 [file 44321_2026_424_MOESM6_ESM.zip › Figure 4 Source Data/Figure 4A/ONEOvac.tif]

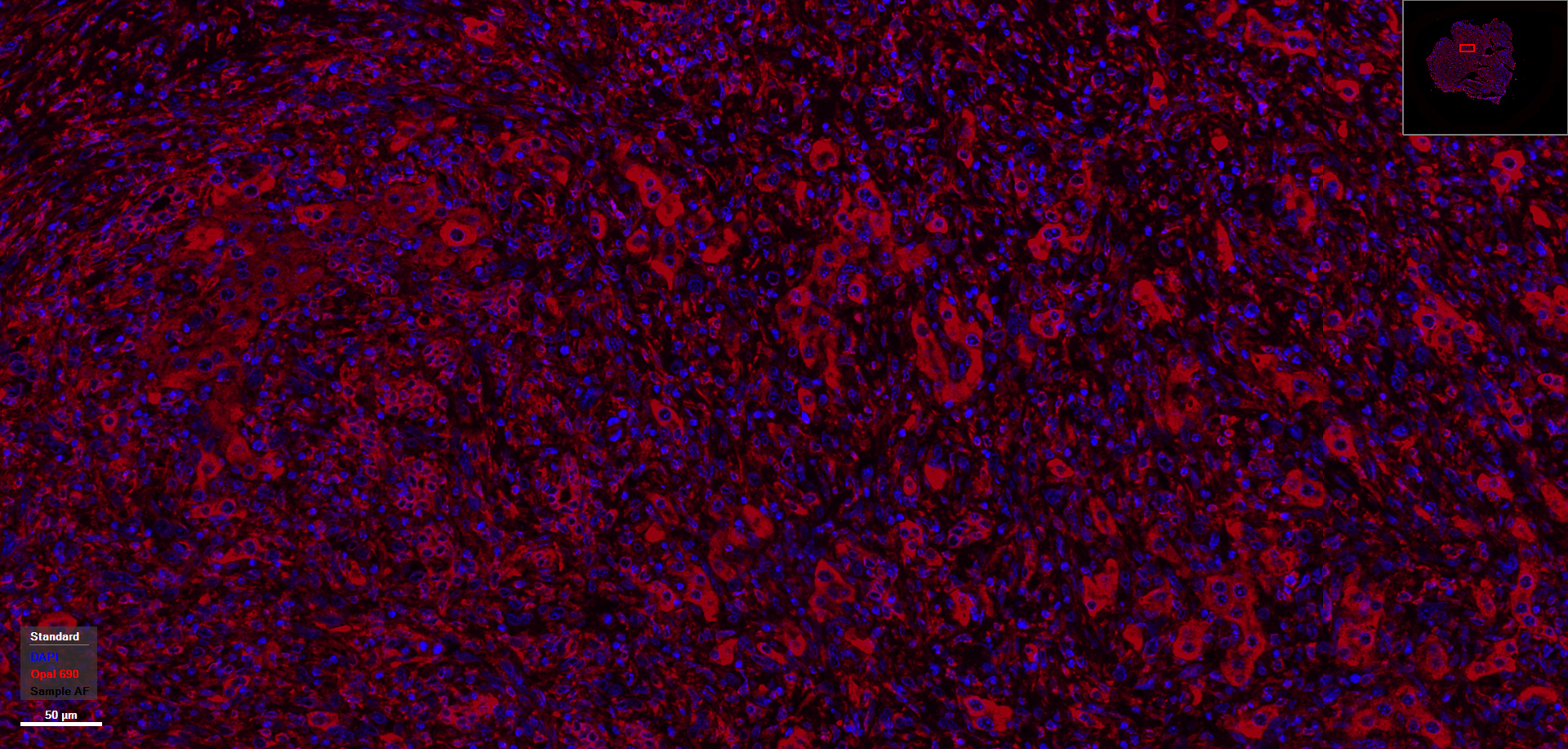

Supplement: Supplementary file 6 — Source data Fig. 4 [file 44321_2026_424_MOESM6_ESM.zip › Figure 4 Source Data/Figure 4A/OMICBvac.tif]

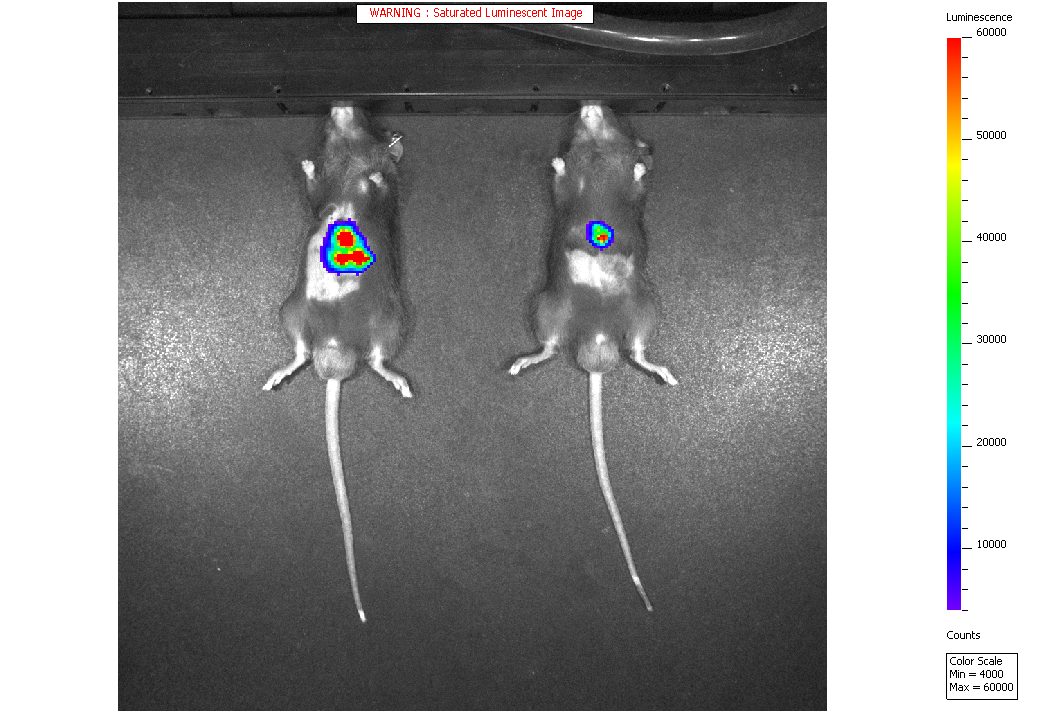

Supplement: Supplementary file 8 — Source data Fig. 7 [file 44321_2026_424_MOESM8_ESM.zip › Figure 7 Source Data/Figure 7B/Day 14/88-94.tif]

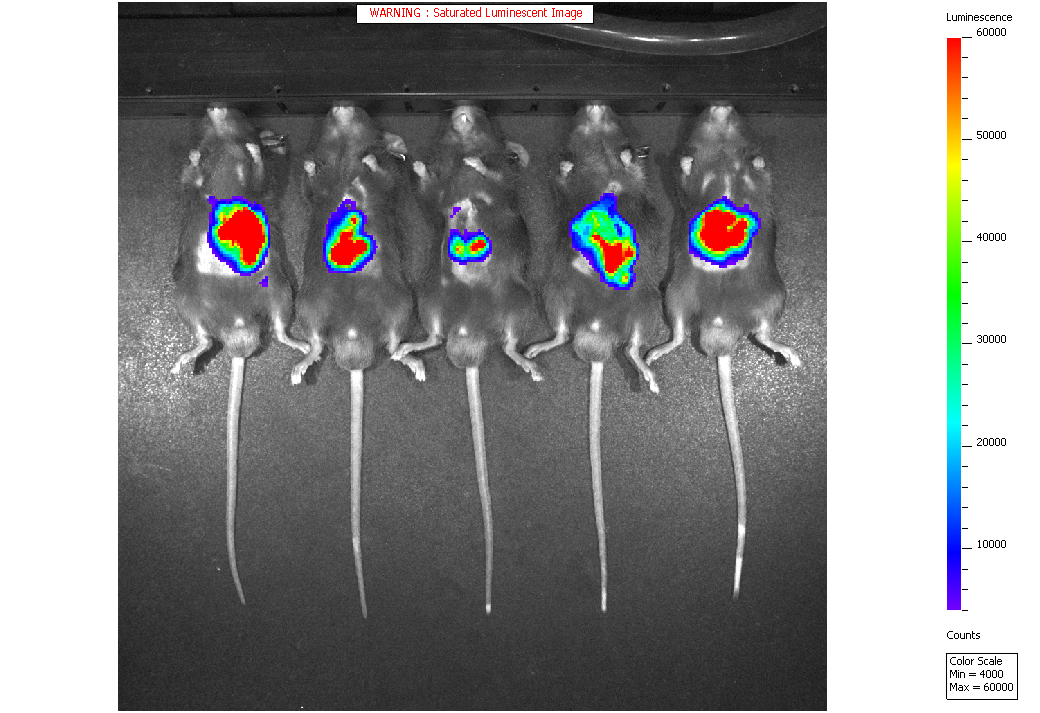

Supplement: Supplementary file 8 — Source data Fig. 7 [file 44321_2026_424_MOESM8_ESM.zip › Figure 7 Source Data/Figure 7B/Day 14/4-COM.tif]

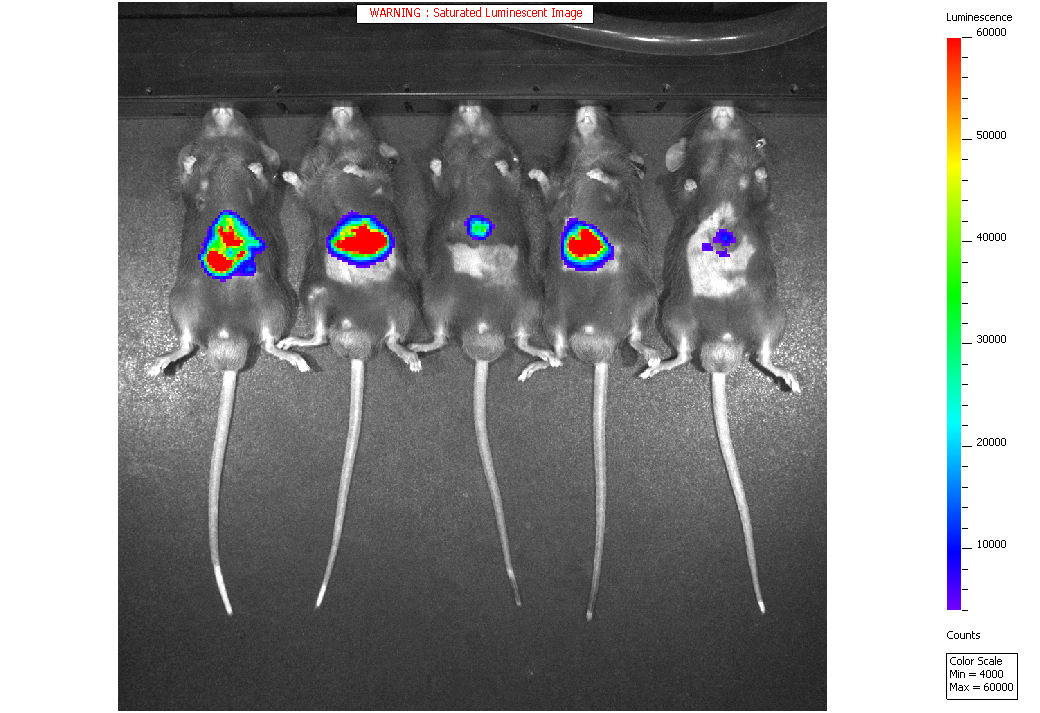

Supplement: Supplementary file 8 — Source data Fig. 7 [file 44321_2026_424_MOESM8_ESM.zip › Figure 7 Source Data/Figure 7B/Day 14/3-COM-IL-15-IN.tif]

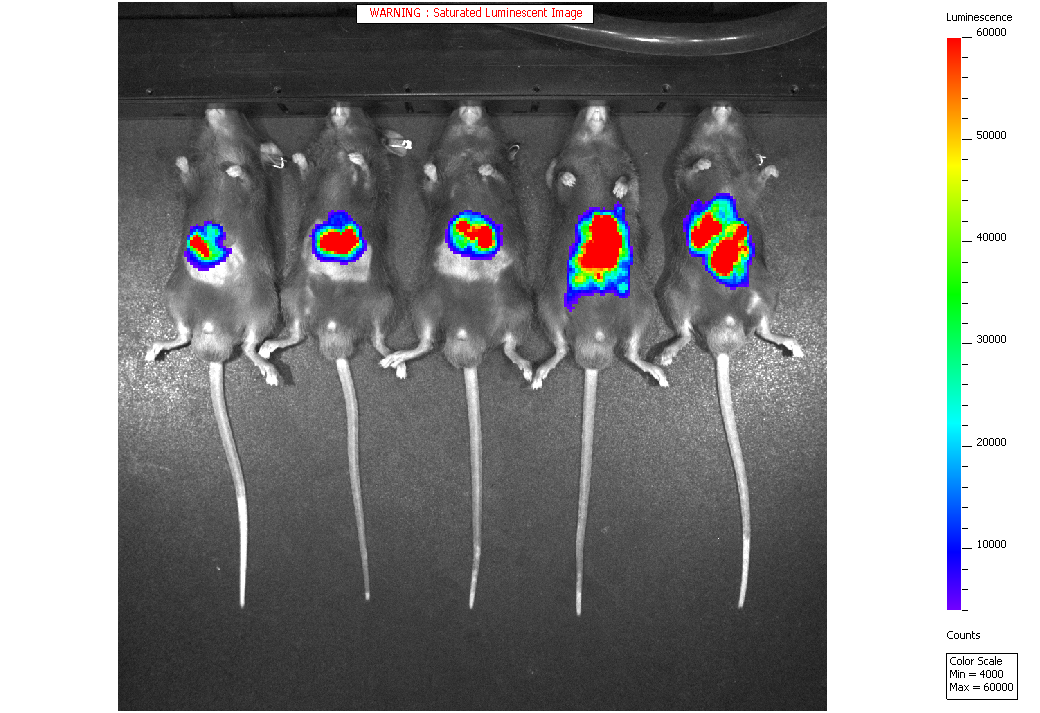

Supplement: Supplementary file 8 — Source data Fig. 7 [file 44321_2026_424_MOESM8_ESM.zip › Figure 7 Source Data/Figure 7B/Day 14/2-IL-15-IN.tif]

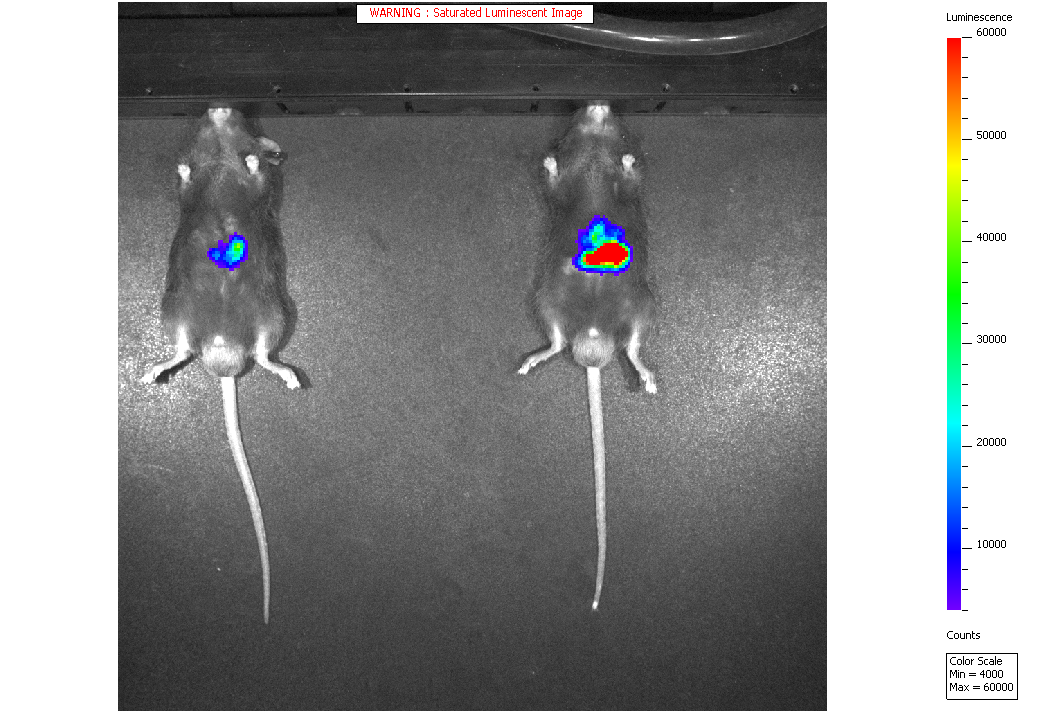

Supplement: Supplementary file 8 — Source data Fig. 7 [file 44321_2026_424_MOESM8_ESM.zip › Figure 7 Source Data/Figure 7B/Day 14/1-PBS-3.tif]

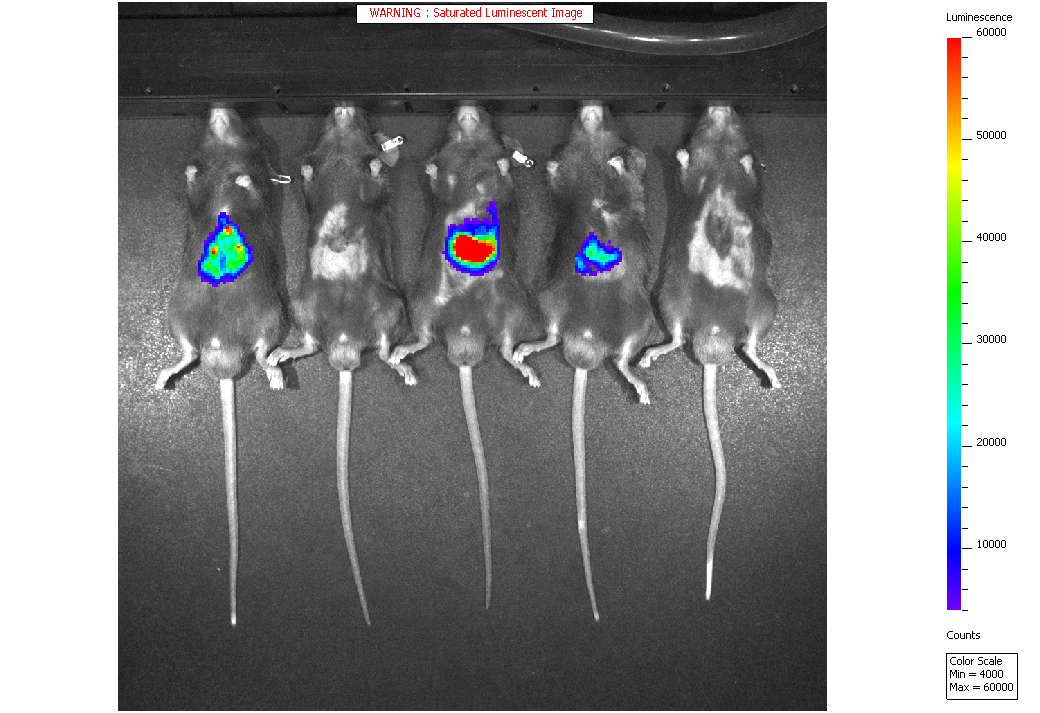

Supplement: Supplementary file 8 — Source data Fig. 7 [file 44321_2026_424_MOESM8_ESM.zip › Figure 7 Source Data/Figure 7B/Day 14/5.tif]

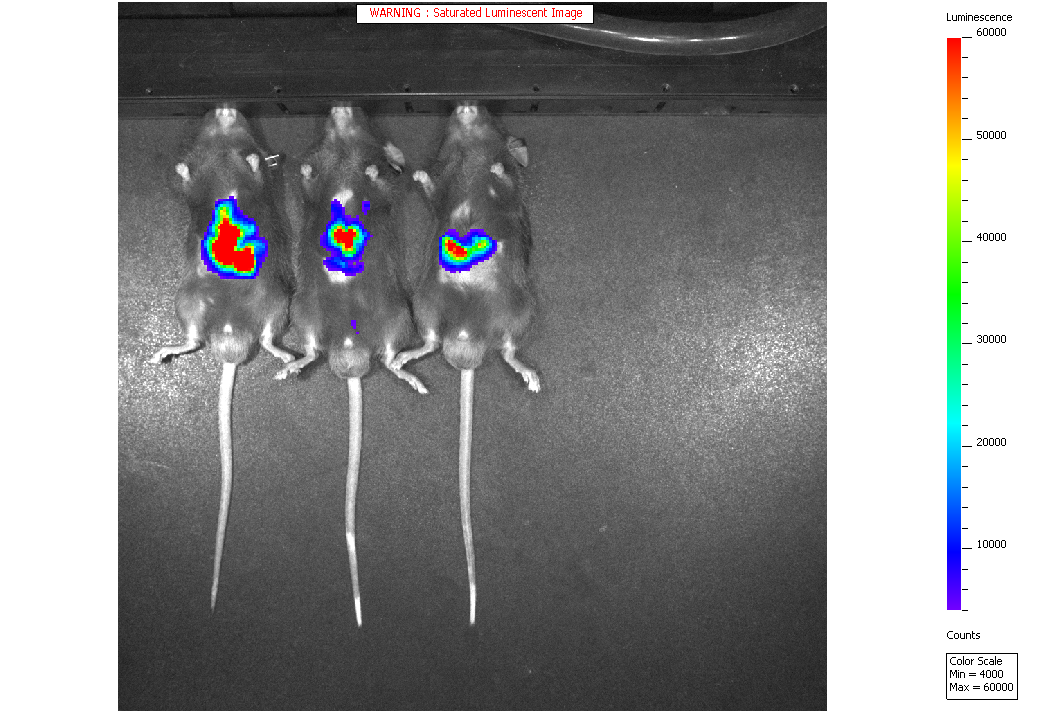

Supplement: Supplementary file 8 — Source data Fig. 7 [file 44321_2026_424_MOESM8_ESM.zip › Figure 7 Source Data/Figure 7B/Day 14/1-PBS-2.tif]

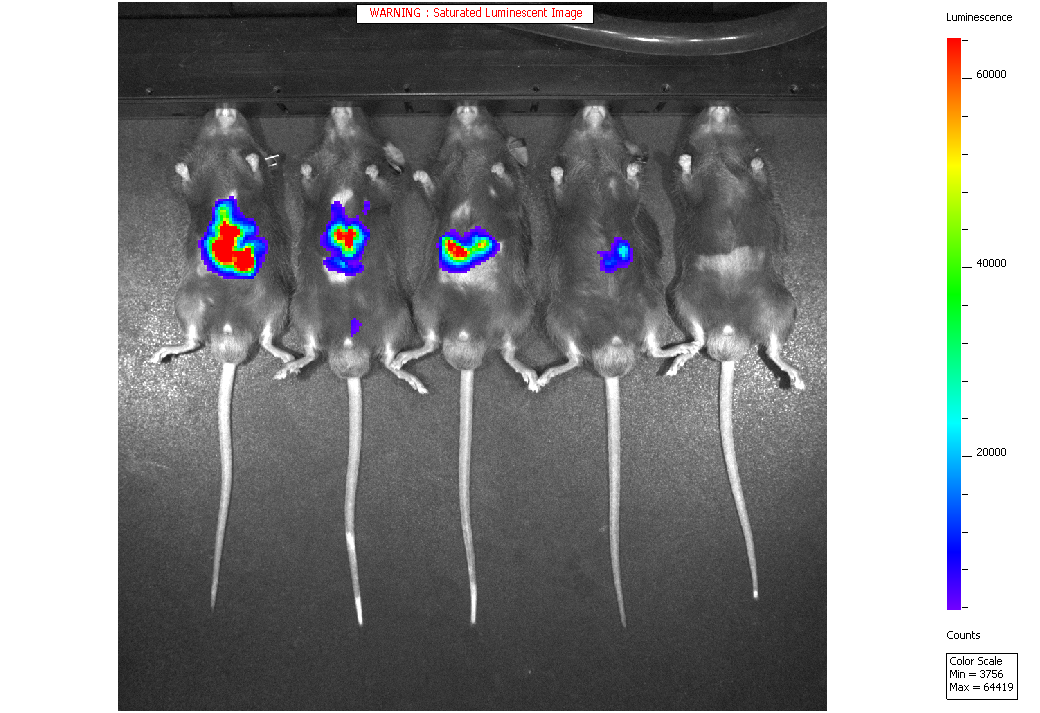

Supplement: Supplementary file 8 — Source data Fig. 7 [file 44321_2026_424_MOESM8_ESM.zip › Figure 7 Source Data/Figure 7B/Day 14/1-PBS-1.tif]

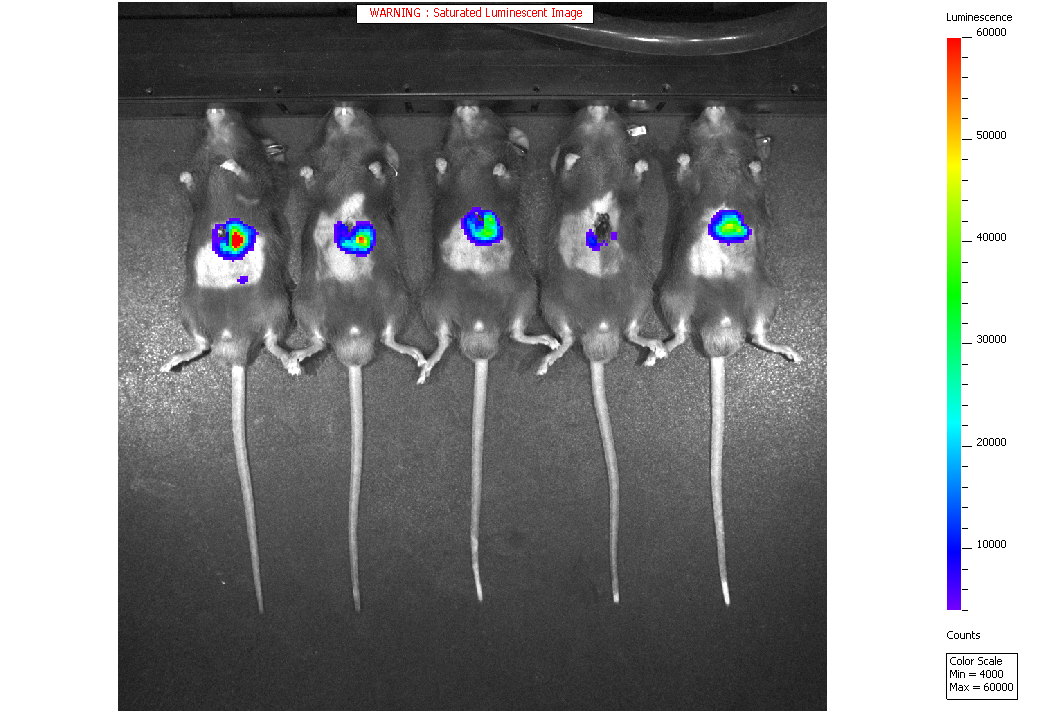

Supplement: Supplementary file 8 — Source data Fig. 7 [file 44321_2026_424_MOESM8_ESM.zip › Figure 7 Source Data/Figure 7B/Day 0/3.tif]

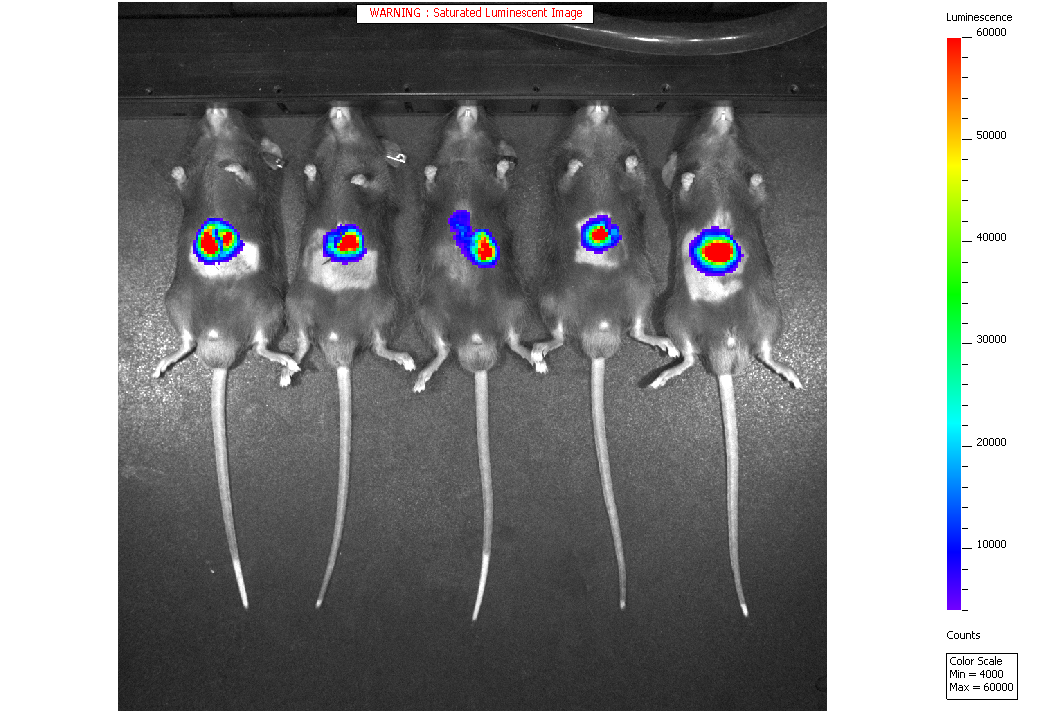

Supplement: Supplementary file 8 — Source data Fig. 7 [file 44321_2026_424_MOESM8_ESM.zip › Figure 7 Source Data/Figure 7B/Day 0/2.tif]

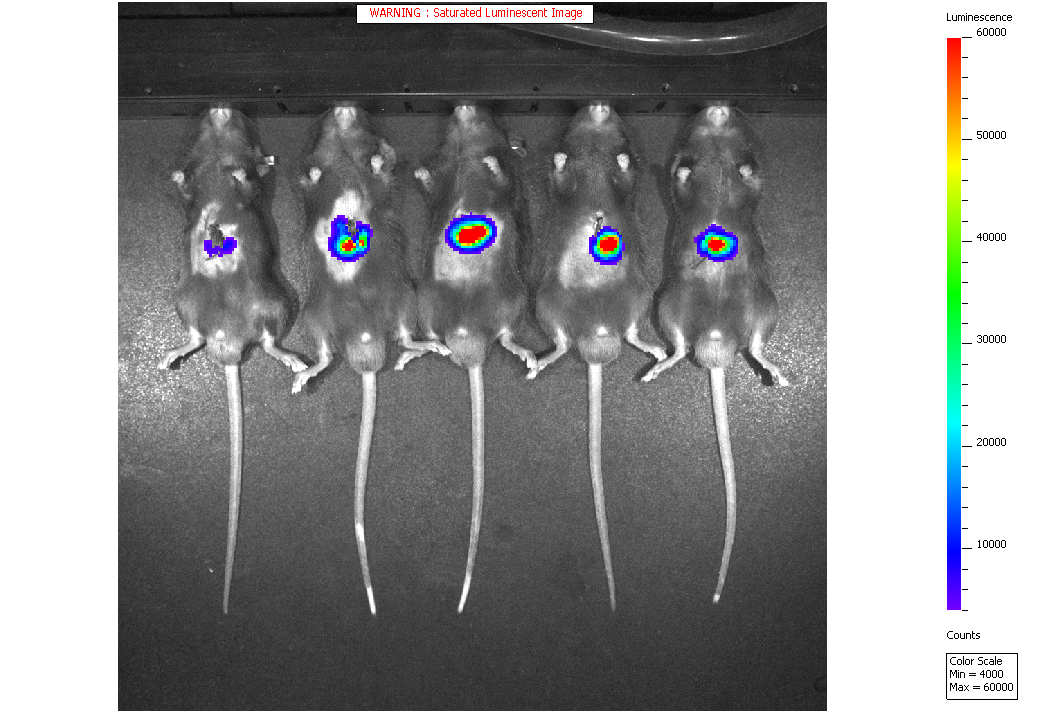

Supplement: Supplementary file 8 — Source data Fig. 7 [file 44321_2026_424_MOESM8_ESM.zip › Figure 7 Source Data/Figure 7B/Day 0/1.tif]

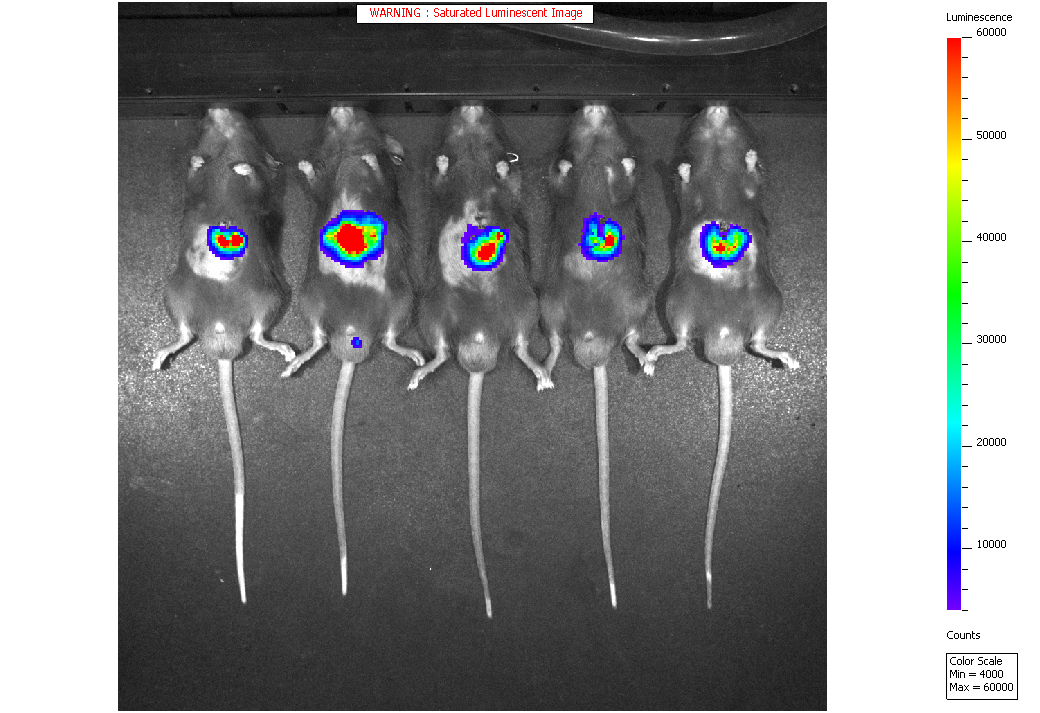

Supplement: Supplementary file 8 — Source data Fig. 7 [file 44321_2026_424_MOESM8_ESM.zip › Figure 7 Source Data/Figure 7B/Day 0/5.tif]

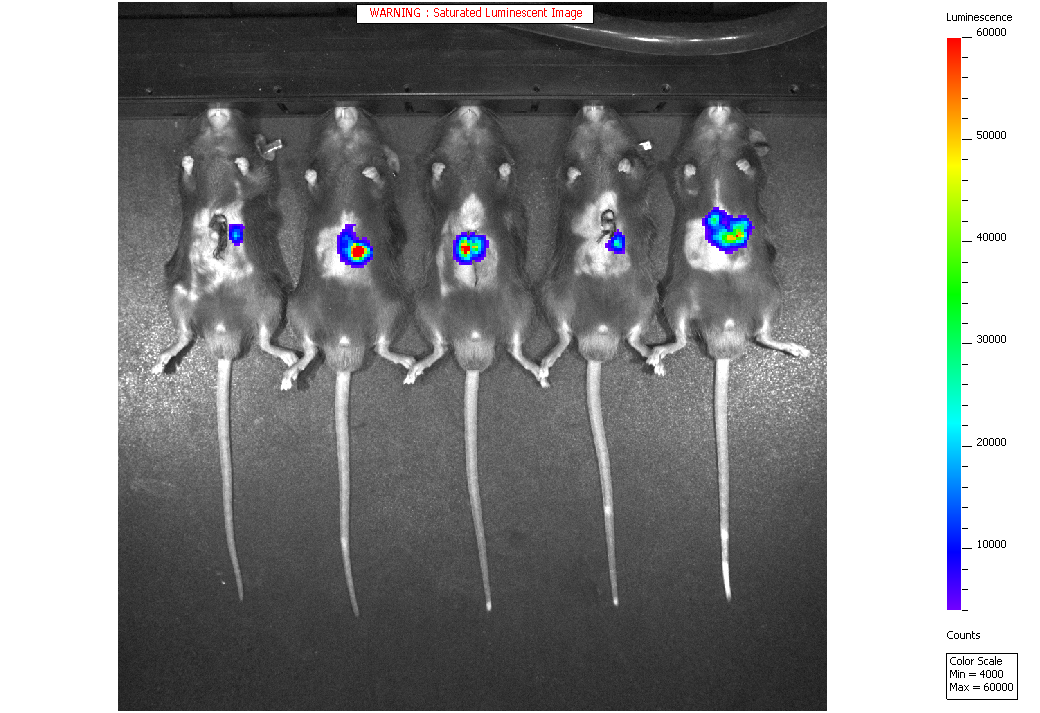

Supplement: Supplementary file 8 — Source data Fig. 7 [file 44321_2026_424_MOESM8_ESM.zip › Figure 7 Source Data/Figure 7B/Day 0/4.tif]

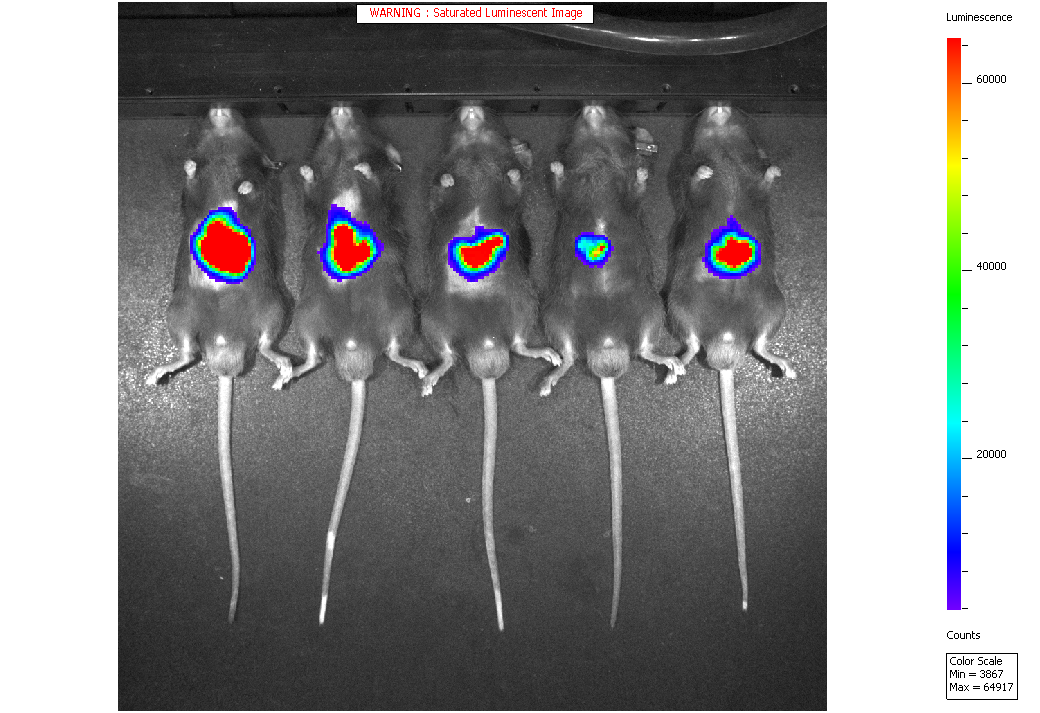

Supplement: Supplementary file 8 — Source data Fig. 7 [file 44321_2026_424_MOESM8_ESM.zip › Figure 7 Source Data/Figure 7B/Day 7/1-pb.tif]

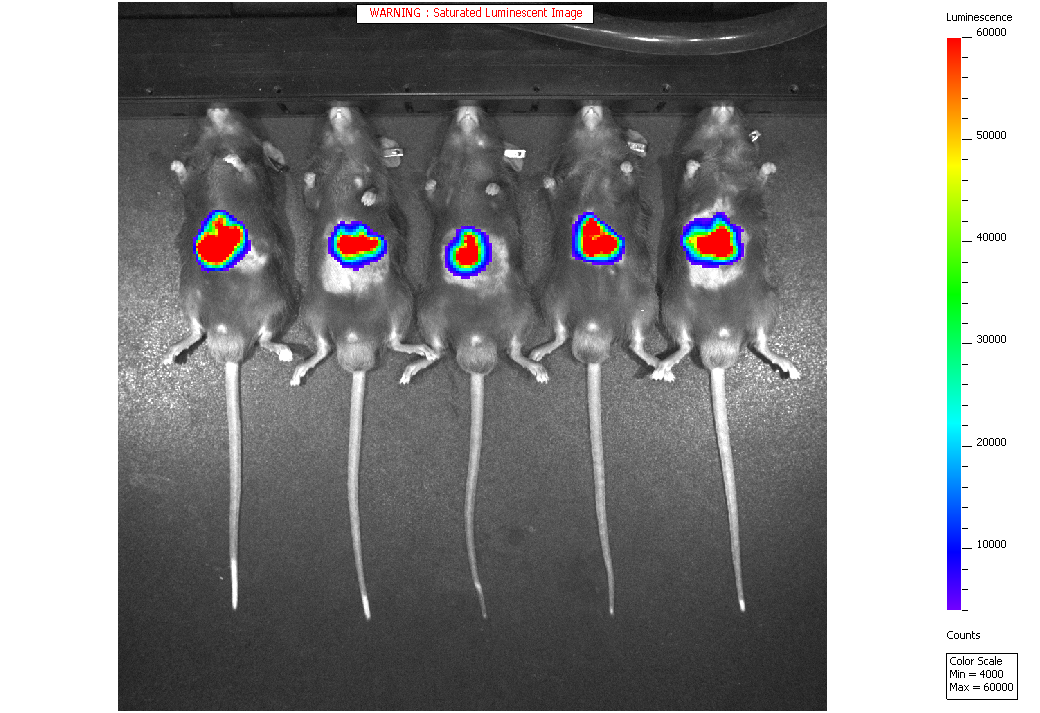

Supplement: Supplementary file 8 — Source data Fig. 7 [file 44321_2026_424_MOESM8_ESM.zip › Figure 7 Source Data/Figure 7B/Day 7/3-combine-IL-15.tif]

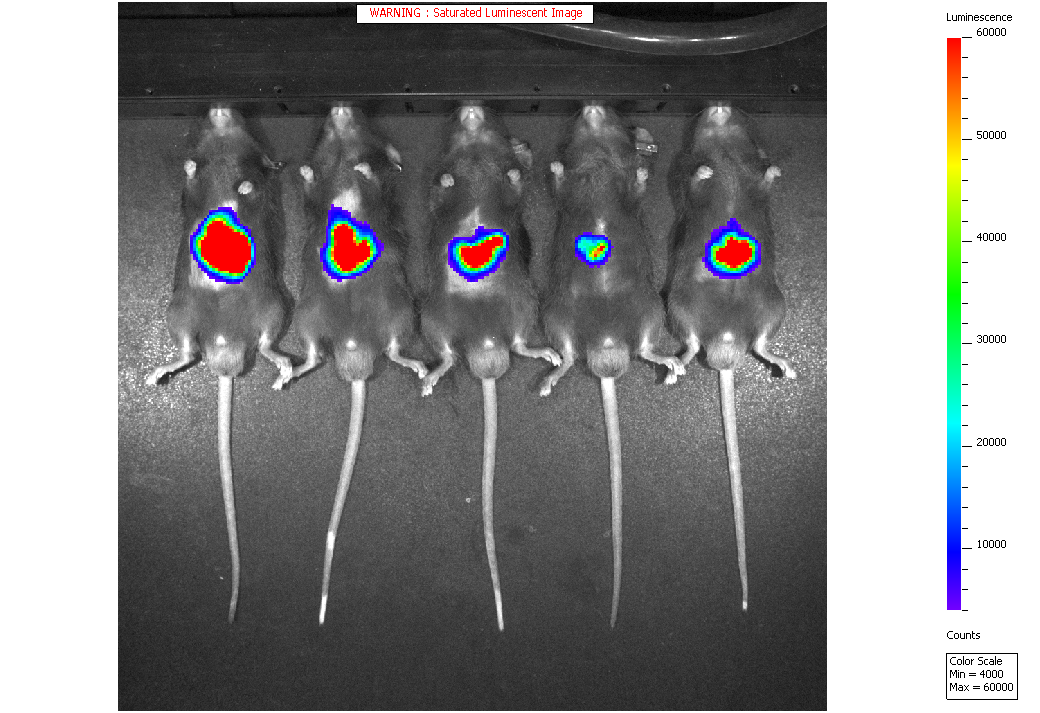

Supplement: Supplementary file 8 — Source data Fig. 7 [file 44321_2026_424_MOESM8_ESM.zip › Figure 7 Source Data/Figure 7B/Day 7/1-pbs.tif]

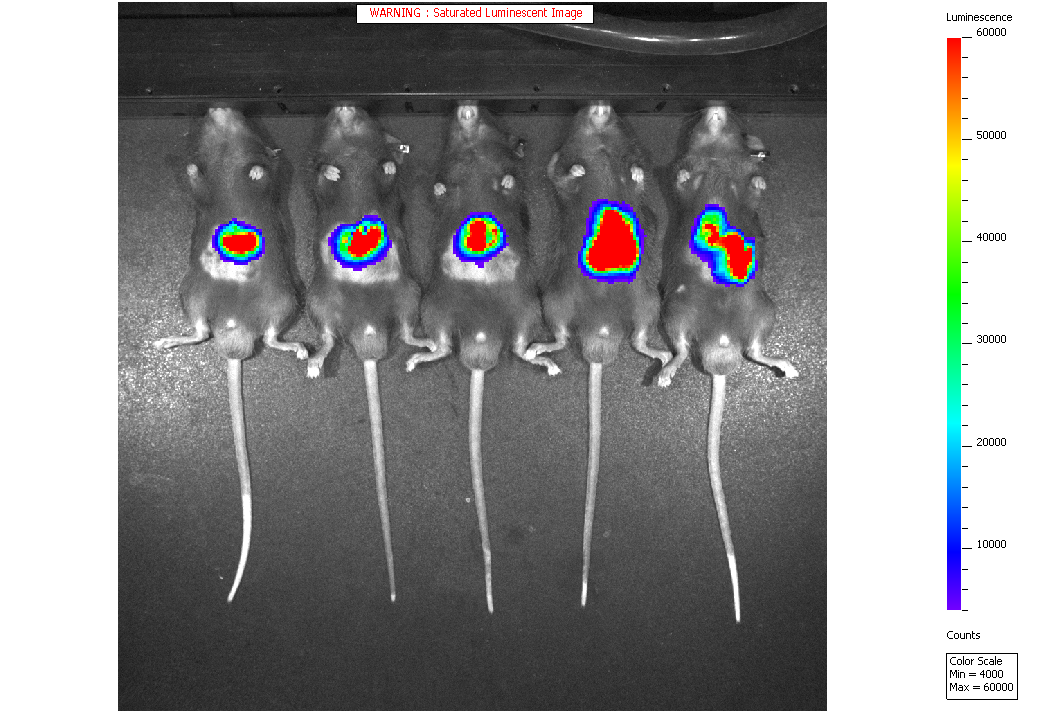

Supplement: Supplementary file 8 — Source data Fig. 7 [file 44321_2026_424_MOESM8_ESM.zip › Figure 7 Source Data/Figure 7B/Day 7/2-IL-15-IN.tif]

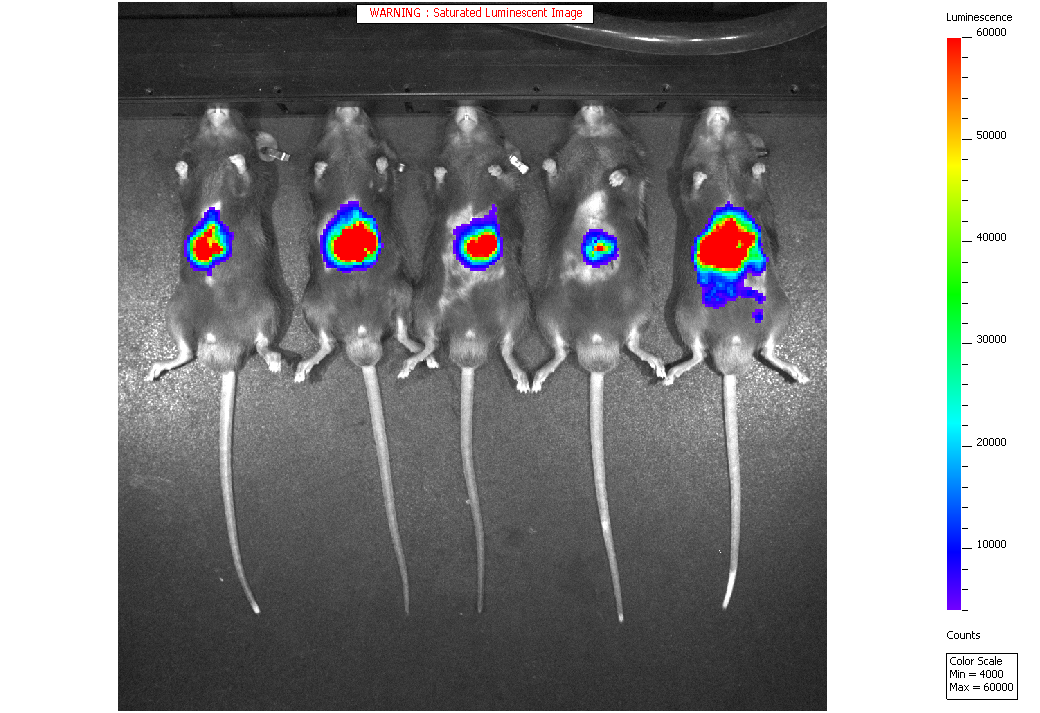

Supplement: Supplementary file 8 — Source data Fig. 7 [file 44321_2026_424_MOESM8_ESM.zip › Figure 7 Source Data/Figure 7B/Day 7/5.tif]

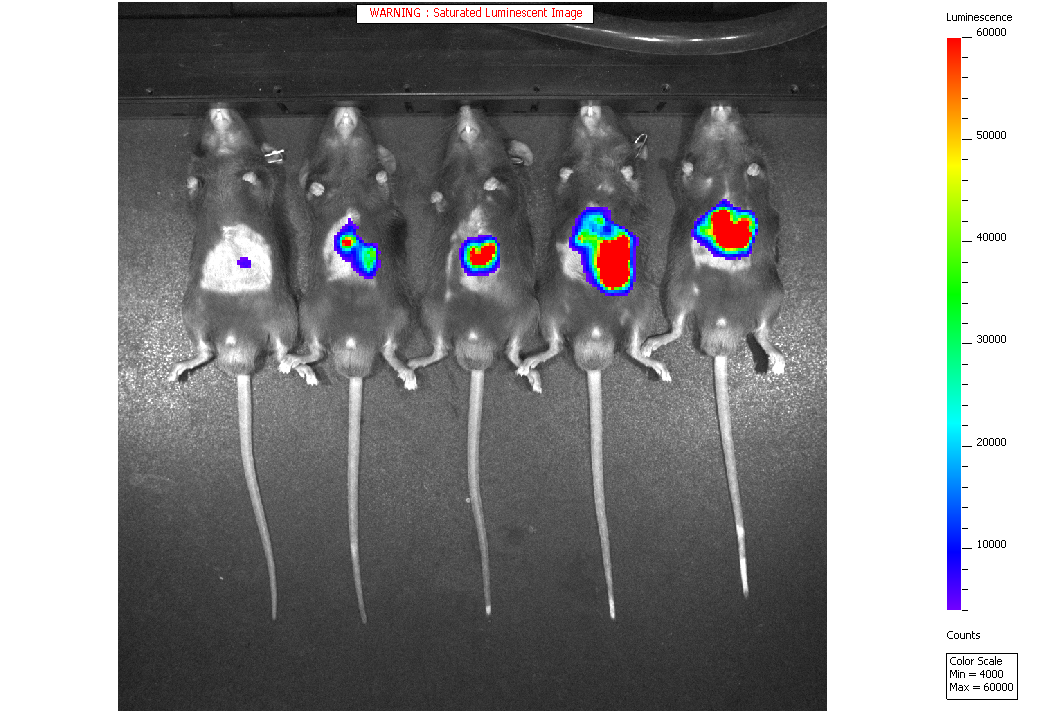

Supplement: Supplementary file 8 — Source data Fig. 7 [file 44321_2026_424_MOESM8_ESM.zip › Figure 7 Source Data/Figure 7B/Day 7/4-combine.tif]
