# Supplementary material for: Neoantigens and shared MICB α3 antigen dual-targeted vaccine generates potent antitumor immunity
Source: EMBO Mol Med. 2026 Apr 17;18(6):2098–123. doi: 10.1038/s44321-026-00424-6 (PMC13269783; doi:10.1038/s44321-026-00424-6)
Supplement: Supplementary file 9 — Expanded View Figures [file 44321_2026_424_MOESM9_ESM.pdf]

Expanded View Figures

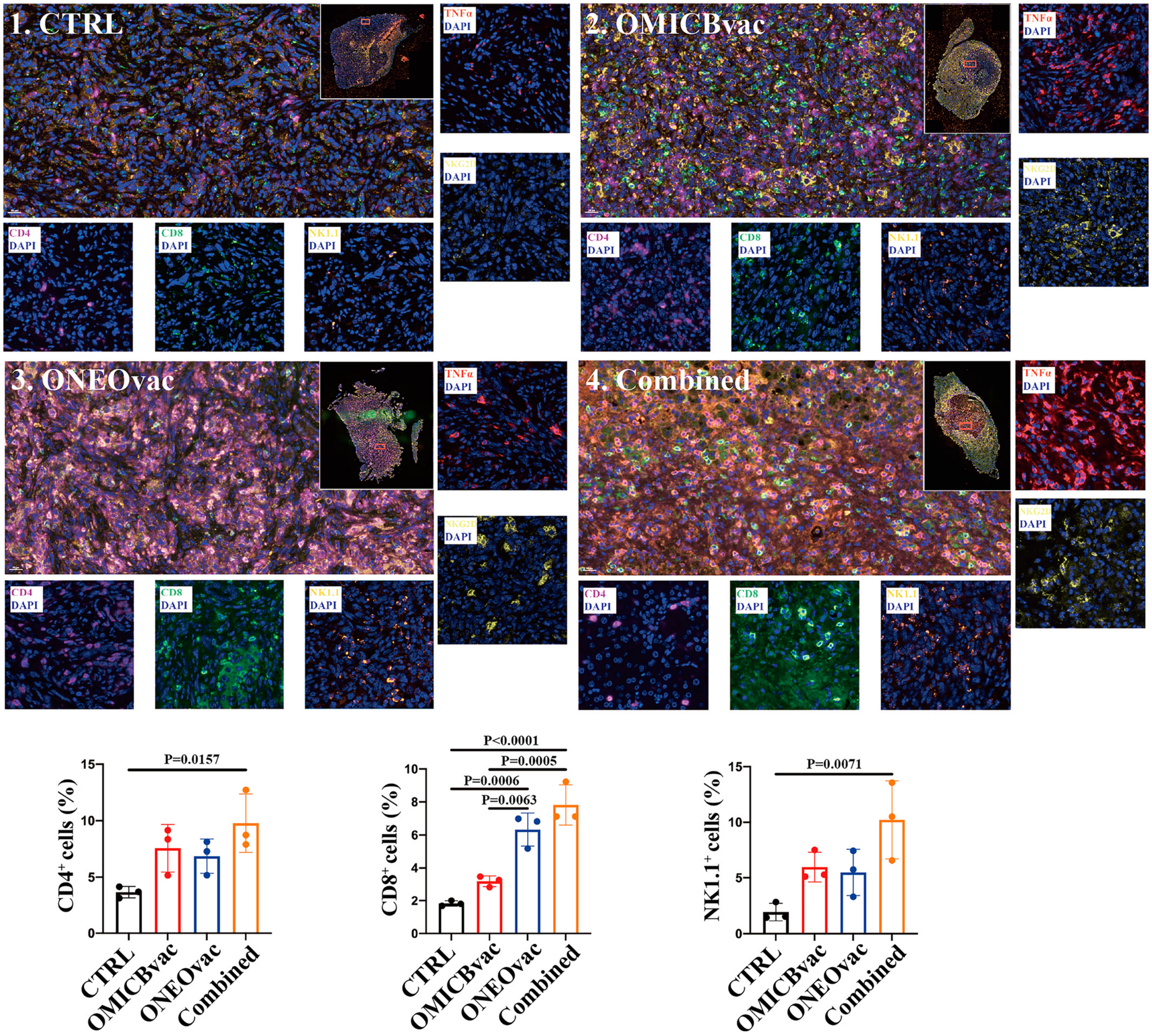

Figure EV1. Immune cell infiltration and effector molecule expression in tumors.

Representative multi-color immunofluorescence images of $CD4^{+}$ T cells, $CD8^{+}$ T cells, and $NK1.1^{+}$ NK cells, as well as the NKG2D and TNF-α expression in tumors across different treatment groups. Statistical scatter plots illustrate the cell density of $CD4^{+}$ T cells, $CD8^{+}$ T cells, and $NK1.1^{+}$ NK cells across entire tumor sections ($n = 3$ slides per group, from different mice; one-way ANOVA). Scale bars, 20 µm. Data are presented as the mean ± SEM. *$P < 0.05$, **$P < 0.01$, ***$P < 0.001$, ****$P < 0.0001$; ns, no significance.

© The Author(s)

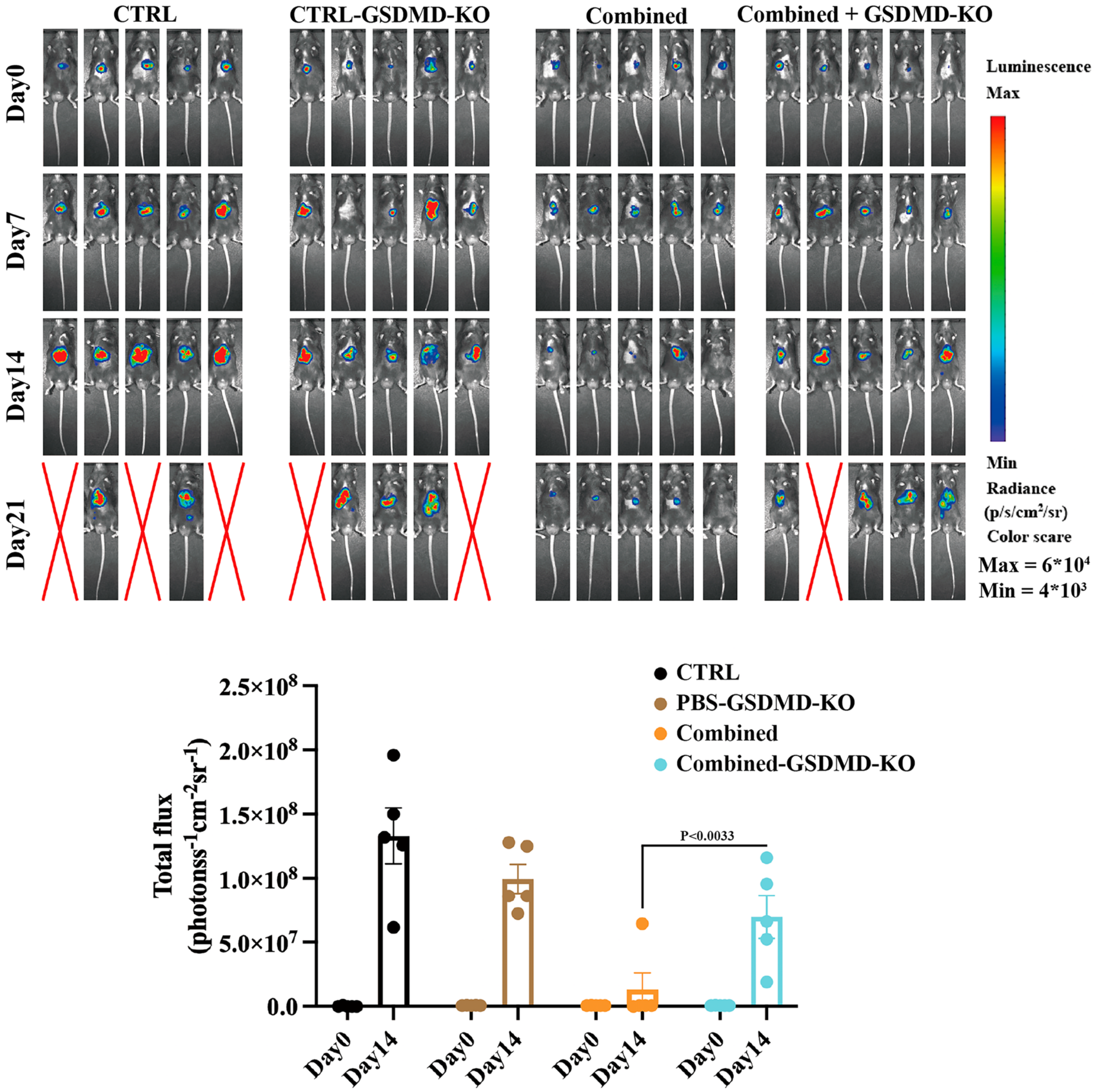

Figure EV2. GSDMD-dependent antitumor efficacy of combined vaccination monitored by bioluminescence imaging.

Tumor burden monitoring by bioluminescence imaging of mice inoculated with Hepa1-6 or Hepa1-6 GSDMD-KO cells and treated with PBS or the combination of ONEOvac and OMICBvac. Each group consisted of 5 mice, which were used to establish tumor models and monitor tumor growth ($n = 5$ mice per group; two-way ANOVA). Data are presented as the mean ± SEM. *$P < 0.05$, **$P < 0.01$, ***$P < 0.001$, ****$P < 0.0001$; ns, no significance.

© The Author(s)

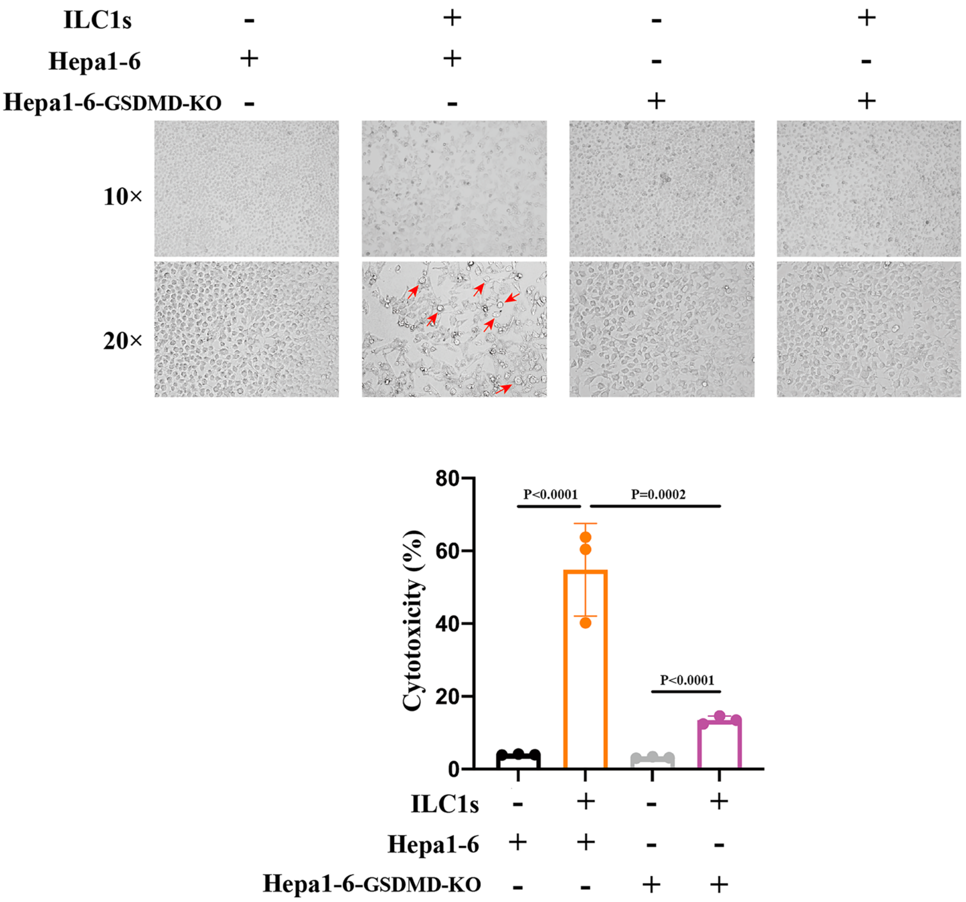

Figure EV3. ILC1-mediated cytotoxicity against tumor cells in vitro.

Microscopic images of ILC1s co-cultured with Hepa1-6-hMICB (or GSDMD KO) cells after 48 h. 12 days after the combined treatment, fresh tumor tissues from mice were collected to prepare single-cell suspensions and stained with CD49a and CD103 for sorting of the ILC1s by flow cytometry. Then ILC1s were co-cultured with Hepa1-6 cells as indicated. LDH analysis of the cell death percentage induced by ILC1s ($n = 3$ independent replicates; one-way ANOVA). 50 µl of the co-culture supernatant from each group was collected for LDH analysis. Data are presented as the mean ± SEM. *$P < 0.05$, **$P < 0.01$, ***$P < 0.001$, ****$P < 0.0001$; ns, no significance.

© The Author(s)
