# Supplementary material for: Neoantigens and shared MICB α3 antigen dual-targeted vaccine generates potent antitumor immunity
Source: EMBO Mol Med. 2026 Apr 17;18(6):2098–123. doi: 10.1038/s44321-026-00424-6 (PMC13269783; doi:10.1038/s44321-026-00424-6)
Supplement: Supplementary file 2 — Peer Review File [file 44321_2026_424_MOESM2_ESM.pdf]

EMBO Molecular Medicine

# Neoantigens and shared MICB α3 antigen dual-targeted vaccine generates potent antitumor immunity

Ruijing Tang, Honghao Ye, Geng Chen, Xiuqing Dong, Zhenli Li, Fangzhou Lin, Tingfeng Huang, Liman Qiu, Gengping Lin, Ming Wu, Haijun Yu, Jianhua Zou, Zhixiong Cai, and Xiaolong Liu

*Corresponding authors: Xiaolong Liu (liuxl@fjirsm.ac.cn) , Zhixiong Cai (zhixiongcai@fjmu.edu.cn), Jianhua Zou (zoujh-93@nus.edu.sg)*

## Review Timeline:

| | |
|---|---|
| Submission Date: | 28th Sep 25 |
| Editorial Decision: | 21st Oct 25 |
| Revision Received: | 26th Jan 26 |
| Editorial Decision: | 12th Feb 26 |
| Revision Received: | 24th Feb 26 |
| Editorial Decision: | 18th Mar 26 |
| Revision Received: | 19th Mar 26 |
| Accepted: | 26th Mar 26 |

*Editor: Lise Roth*

## Transaction Report:

(Note: With the exception of the correction of typographical or spelling errors that could be a source of ambiguity, letters and reports are not edited. Depending on transfer agreements, referee reports obtained elsewhere may or may not be included in this compilation. Referee reports are anonymous unless the Referee chooses to sign their reports.)

21st Oct 2025

Dear Dr. Liu,

Thank you for submitting your manuscript to EMBO Molecular Medicine. We have now received feedback from the two reviewers who agreed to evaluate it. As you will see from the reports below, while they acknowledge the potential interest of the findings, they also raise a number of concerns that must be addressed before the manuscript can be considered further. In particular, the robustness and scientific rigor of the work will need to be improved. If you feel you can address these points satisfactorily, you may wish to submit a revised version of your manuscript.

Fully addressing the reviewers' concerns will be necessary for us to consider the manuscript further, and acceptance will entail a second round of review. EMBO Molecular Medicine only encourages a single round of revisions, so acceptance or rejection of the manuscript will depend on how complete your responses are in the final version. To avoid any frustration later on, I would strongly advise against submitting an incomplete revision.
If you would like to discuss further the points raised by the referees, I am available to do so via email or video. Let me know if you are interested in this option.

We are expecting your revised manuscript within three months, if you anticipate any delay, please contact us.

When submitting your revised manuscript, please carefully review the instructions that follow below. We perform an initial quality control of all revised manuscripts before re-review; failure to include requested items will delay the evaluation of your revision.

We require:

1) A .docx formatted version of the manuscript text (including legends for main figures, EV figures and tables). Please make sure that the changes are highlighted to be clearly visible.

2) Individual production quality figure files as .eps, .tif, .jpg (one file per figure). For guidance, download the 'Figure Guide PDF' (https://www.embopress.org/page/journal/17574684/authorguide#figureformat).

3) At EMBO Press we ask authors to provide source data for the main manuscript figures. You will receive a separate email with instructions for providing source data with your revised manuscript, including how to upload and organize the files.

Additional information on source data and instruction on how to label the files are available

4) A .docx formatted letter INCLUDING the reviewers' reports and your detailed point-by-point responses to their comments. As part of the EMBO Press transparent editorial process, the point-by-point response is part of the Review Process File (RPF), which will be published alongside your paper.

5) A complete author checklist, which you can download from our author guidelines (https://www.embopress.org/page/journal/17574684/authorguide#submissionofrevisions). Please insert information in the checklist that is also reflected in the manuscript. The completed author checklist will also be part of the RPF.

6) All Materials and Methods need to be described in the main text using our 'Structured Methods' format. According to this format, the Methods section includes a Reagents and Tools Table (listing key reagents, experimental models, software and relevant equipment and including their sources and relevant identifiers) followed by a Methods and Protocols section describing the methods, ideally using a step-by-step protocol format. The aim is to facilitate adoption of the methodologies across labs. Please download and fill our Reagents and Tools Table template (.docx), which you can find in our author guidelines: https://www.embopress.org/page/journal/14693178/authorguide#structuredmethods.
When submitting your revised manuscript, please do not include the Reagents and Tools Table in the Methods section of the manuscript but upload it as a separate file choosing the file type "Reagent Table".

7) Please note that all corresponding authors are required to supply an ORCID ID for their name upon submission of a revised manuscript.

8) It is mandatory to include a 'Data Availability' section after the Materials and Methods. Before submitting your revision, primary datasets produced in this study need to be deposited in an appropriate public database, and the accession numbers and database listed under 'Data Availability'. Please remember to provide a reviewer password if the datasets are not yet public (see https://www.embopress.org/page/journal/17574684/authorguide#dataavailability).

In case you have no data that requires deposition in a public database, please state so in this section. Note that the Data Availability Section is restricted to new primary data that are part of this study.

9) For data quantification: please specify the name of the statistical test used to generate error bars and P values, the number (n) of independent experiments (specify technical or biological replicates) underlying each data point and the test used to calculate p-values in each figure legend. The figure legends should contain a basic description of n, P and the test applied. Graphs must include a description of the bars and the error bars (s.d., s.e.m.). Please provide exact p values.

10) Our journal encourages inclusion of *data citations in the reference list* to directly cite datasets that were re-used and obtained from public databases. Data citations in the article text are distinct from normal bibliographical citations and should directly link to the database records from which the data can be accessed. In the main text, data citations are formatted as follows: "Data ref: Smith et al, 2001" or "Data ref: NCBI Sequence Read Archive PRJNA342805, 2017". In the Reference list, data citations must be labeled with "[DATASET]". A data reference must provide the database name, accession number/identifiers and a resolvable link to the landing page from which the data can be accessed at the end of the reference. Further instructions are available at .

11) We replaced Supplementary Information with Expanded View (EV) Figures and Tables that are collapsible/expandable online. EV Figures should be cited as 'Figure EV1, Figure EV2" etc... in the text and their respective legends should be included in the main text after the legends of regular figures.
- For the figures that you do NOT wish to display as Expanded View figures, they should be bundled together with their legends in a single PDF file called *Appendix*, which should start with a short Table of Content. Appendix figures should be referred to in the main text as: "Appendix Figure S1, Appendix Figure S2" etc.
- Additional Tables/Datasets should be labeled and referred to as Table EV1, Dataset EV1, etc. Legends have to be provided in a separate tab in case of .xls files. Alternatively, the legend can be supplied as a separate text file (README) and zipped together with the Table/Dataset file.
See detailed instructions here:

12) The paper explained: EMBO Molecular Medicine articles are accompanied by a summary of the articles to emphasize the major findings in the paper and their medical implications for the non-specialist reader. Please provide a draft summary of your article highlighting
- the medical issue you are addressing,
- the results obtained and
- their clinical impact.
This may be edited to ensure that readers understand the significance and context of the research. Please refer to any of our published articles for an example.

13) Author contributions: CRediT has replaced the traditional author contributions section because it offers a systematic machine readable author contributions format that allows for more effective research assessment. Please remove the Authors Contributions from the manuscript and use the free text boxes beneath each contributing author's name in our system to add specific details on the author's contribution. More information is available in our guide to authors.

14) Disclosure statement and competing interests: We updated our journal's competing interests policy in January 2022 and request authors to consider both actual and perceived competing interests. Please review the policy https://www.embopress.org/competing-interests and update your competing interests if necessary.

15) Every published paper now includes a 'Synopsis' to further enhance discoverability. Synopses are displayed on the journal webpage and are freely accessible to all readers. They include a short stand first (maximum of 300 characters, including space) as well as 2-5 one-sentences bullet points that summarizes the paper. Please write the bullet points to summarize the key NEW findings. They should be designed to be complementary to the abstract - i.e. not repeat the same text. We encourage inclusion of key acronyms and quantitative information (maximum of 30 words / bullet point). Please use the passive voice. Please attach these in a separate file or send them by email, we will incorporate them accordingly.

Please also suggest a visual abstract to illustrate your article as a PNG file 550 px wide x 300-600 px high. A cropped portion of this image will serve as thumbnail for the table of content on our webpage.

16) As part of the EMBO Publications transparent editorial process initiative (see our Editorial at http://embomolmed.embopress.org/content/2/9/329), EMBO Molecular Medicine will publish online a Review Process File (RPF) to accompany accepted manuscripts.
In the event of acceptance, this file will be published in conjunction with your paper and will include the anonymous referee reports, your point-by-point response and all pertinent correspondence relating to the manuscript. Let us know whether you agree with the publication of the RPF and as here, if you want to remove or not any figures from it prior to publication.
Please note that the Authors checklist will be published at the end of the RPF.

EMBO Molecular Medicine has a "scooping protection" policy, whereby similar findings that are published by others during review or revision are not a criterion for rejection. Should you decide to submit a revised version, I do ask that you get in touch

after three months if you have not completed it, to update us on the status.

I look forward to receiving your revised manuscript.

Yours sincerely,

Lise Roth

Lise Roth, PhD
Senior Editor
EMBO Molecular Medicine

***** Reviewer's comments *****

Referee #1 (Comments on Novelty/Model System for Author):

novel combination of vaccine
Many preclinical tumor model some of them are orthotopic
Mechanisms of action analyzed.
Originality about the role of pyroptosis and ILC1

Referee #1 (Remarks for Author):

Although immunization with each vaccine as monotherapy has already been reported, the combination of the two is original as a way of counteracting potential escape mechanisms linked to CD8+T cells or NK cells. The immunogenicity of the vaccine combination is well demonstrated, as has the role of immune subpopulations in the in vivo efficacy of the combined vaccine through well-documented depletion experiments. The vaccine has been evaluated in various preclinical models, including orthotopic models.
A few suggestions to further enhance the quality of this manuscript:

- Can the authors indicate the purity of their OMV vaccine? Have they eliminated the presence of contaminants (LPS) from the bacteria? Is the protein content of the OMVs reproducible from batch to batch?
- the authors insist about the role of ILC1 and pyroptosis to explain the efficacy of the combined vaccine, even though the depletion of CD8 T cells and NK cells also demonstrates their role. Could the ratio of ILC1/tumor cells to CD8 T cells/tumor cells or NK cells/tumor cells be shown to better assess the respective role of each population in the vaccine's effect?
- Could NK cells and CD8 T lymphocytes also play a role in pyroptosis? The experiment with the IL-15 inhibitor is important but does not rule out a role for NK cells.
- The authors show that ILC1s exert cytotoxicity against tumor cells. Is this cytotoxicity dependent on NKG2D? As a control, could the authors show that ILC1s do not exert cytotoxicity against normal hepatocytes?
- Line 345: Isn't it surprising that after 48 hours of co-culture with dendritic cells, neoantigen-specific T lymphocytes have already been primed and are then able to lyse tumor cells?
- Fig. S7C: It is unclear whether the difference between the use of vaccines as monotherapy vs. in combination is significant. Statistical tests are missing.
- Could the authors explain their choice of hyperimmunization at short intervals?
- Some recent articles on vaccines using neoantigens in liver cancer could be mentioned (Yarchoan M Nat Med 2024).

Referee #2 (Remarks for Author):

Immunogenic peptides, known as tumor neoantigens, generated by specific gene variations in tumor cells can be recognized by T cells, inducing specific tumor cell killing. Thus, precise immunotherapy has been developed to target tumors. Nevertheless, hepatocellular carcinoma (HCC) with rapid progression and strong heterogeneity is normally resistant to this immunotherapy. In addition, tumors cells have high expression of MHC class I chain-related polypeptide A/B (MIC A/B) which can be recognized through NKG2D and eliminated by NK cells. However, tumor cells can evade immune recognition pathway through proteolytic shedding of MICA. A previous study has demonstrated that antibody-mediated inhibition of MICA and MICB by blocking their

shedding through binding to the a3 domains can reactivate tumor immunity. In this study, the authors developed a novel synergistic therapeutic vaccine strategy that combines neoantigens and MICB a3 antigen to induce tumor immunity in HCC. They engineered and produced neoantigen protein (ONEOvac) and MICA or B peptides (OMICAvac or OMICBvac). In vitro studies demonstrated that ONEOvac could activate immune cells. In vivo studies revealed that OMICvac and OMICBvac could block the shedding of MICA/B in tumor cells, with the highest efficacy in OMICBvac. Thus, the authors, by using B6 mice with subcutaneous injection of Hep1-6 (a B6 derived liver cancer cell line), evaluated whether vaccination of ONEOvac and OMICBvac could elicit tumor immunity, resulting in tumor regression. Indeed, ONEOvac and OMICBvac both could suppress tumor progression likely through T and NK cell induced tumor cell killing effects. The authors further examined the tumor killing effects of different injection and combination of ONEOvac and OMICBvac. It was found that the combined vaccination of these two has the best tumor killing effects when compared with the single vaccination. Besides, the tumor killing effects of vaccination were likely through pyroptosis as GASMD knockout reversed the effects. The authors also determined that the combined vaccination induced significant ILC1 cell infiltration which could be regulated by IL-15 since IL-15 inhibitor reduced ILC1 cell infiltration and tumor regression in mice treated with the combined vaccination. Overall, the current study provides a novel combinational therapy by combining tumor neoantigen and MICA/B vaccination to enhance tumor killing effects. Several weaknesses identified in its current form should be carefully addressed.

1. In the materials and methods, the authors described that plasmids were transformed into E. coli. They later described that plasmids were transfected into E. coli. Please revise the correct usage accordingly.
2. What is wet experiment? Please describe the manuscript in a professional way.
3. In Fig. 1, some experiments are described with details about the dose of peptides. However, some experiments have no detailed information making data interpretation difficult.
4. In Fig. 2E, did the flowcytometry data show consistent higher CD8+ T cell population in ONEOvac and OMICBvac treated tumor samples when compared with the control or OMVs groups?
5. Authors suggested that these vaccination treatments did not influence normal organ functions by analyzing histological changes and serum markers in the mice with orthotopic liver cancer cell inoculation. It is expected that the treated groups with no tumor cells should have reduced ALT and AST. However, the authors' findings contradicted the complete eradication of liver cancer cells (Fig. S8).
6. In Fig. 3, all mice dead in CTRL group, how did the authors obtain samples for analysis? If tumor samples were collected after mice dead, would this impact results?
7. In Fig. 3, why was data collected with different N numbers?
8. In Fig. 4E, the images are poor quality.
9. In Fig. 4I and J, many mice dead before the authors collected tumor samples. How did the authors get 5 tumor samples for each group?
10. In Fig. 5B, the authors stated T&NK cell increased in the ONEOvac and combined groups compared to the CTRL group. However, hepatocytes decreased substantially. Did the hepatocyte population include HCC?
11. In Fig. 5C, why do the T SNE images have almost no overlap cell types?
12. In Fig. 6C-E, what did you want to compare? In Fig. 6E, the Heatmap data actually suggested many inflammatory cytokines are related to these changes. In Fig. 6F, how did you measure IL-15 in tumor samples by using ng/ml as unit? What samples did you use?
13. In Fig. 7C-H, the authors described N is equal to 5 for all studies. However, the presented data did not support the statement. Besides, how could the authors get identical data in CTRL group for Fig. 7G? It is common that inflammatory cytokines vary significantly between mouse samples.
14. Should the authors repeat the experiments in a humanized mouse model by using human HCC cells?

Mengchao Hepatobiliary Hospital of Fujian Medical University, Fuzhou, 350025, China

E-mail: xiaoloong.liu@gmail.com

**The point by point responses (in black) to the comments (in blue italics) of all reviewers have been listed below for your editorial checking.**

Referee #1

*Although immunization with each vaccine as monotherapy has already been reported, the combination of the two is original as a way of counteracting potential escape mechanisms linked to CD8+T cells or NK cells. The immunogenicity of the vaccine combination is well demonstrated, as has the role of immune subpopulations in the in vivo efficacy of the combined vaccine through well-documented depletion experiments. The vaccine has been evaluated in various preclinical models, including orthotopic models.*

Response: Thanks very much to comment our work as “original” and “well demonstrated”. We sincerely appreciate the efforts and comments from the reviewer to significantly improve the quality of our manuscript. All the arisen issues have been addressed.

*A few suggestions to further enhance the quality of this manuscript:*

*1. Can the authors indicate the purity of their OMV vaccine? Have they eliminated the presence of contaminants (LPS) from the bacteria? Is the protein content of the OMVs reproducible from batch to batch?*

Response: We sincerely thank the reviewer for these critical questions regarding our OMV vaccine quality control. Our OMV preparations demonstrated high structural homogeneity. Transmission electron microscopy revealed uniform spherical vesicles without visible bacterial debris or other particulate contaminants (Figure 1B and S4A). Dynamic light scattering analysis confirmed a monodisperse size distribution (Figure

S1A, S4B), and Western blot analysis showed clear bands corresponding to our engineered ClyA fusion proteins (Figure 1C, S4C).

Regarding the presence of LPS, we wish to clarify that LPS is an intrinsic structural component of the outer membrane of Gram-negative bacteria and their derived OMVs, not a separable contaminant. We have quantified the LPS content in our final vaccine preparations using a sensitive ELISA assay, which yielded consistent and low levels (~100 EU/μg, Figure S13). Based on the comprehensive safety assessment data presented in Figure S8 (including serum biochemistry and histopathology), we can confirm that this LPS concentration is well below the threshold that would cause significant non-specific immune activation or toxicity in our mouse models, indicating a well-controlled and safe formulation.

Batch-to-batch reproducibility was confirmed through BCA assay across three independent production batches (Figure S13, the complete source data for this figure has been provided as part of the manuscript submission). Both total protein content and specific antigen expression remained highly consistent, demonstrating the robustness of our OMV production platform.

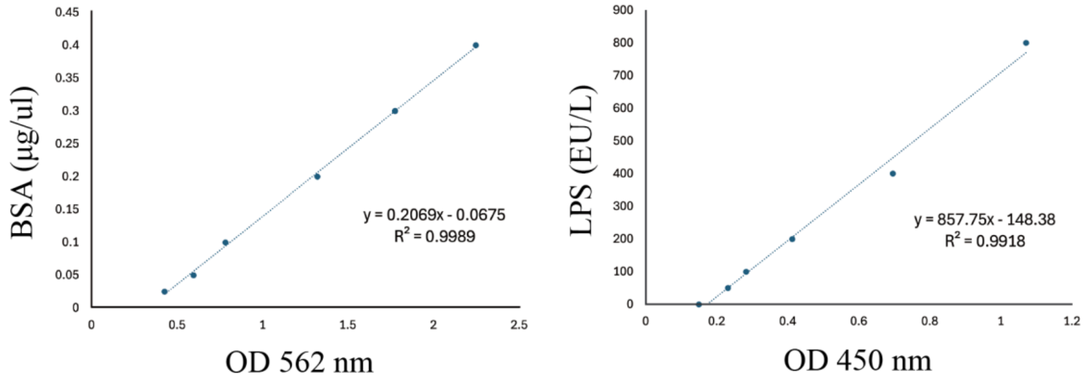


| Sample/100mL *E.coli* | Protein amount (ug) | LPS (EU/ug) |
|---|---|---|
| CTRL (empty plasmid) | 388.5 ± 7.647 | 86.22 ± 1.285 |
| ONEOvac | 523.4 ± 4.930 | 94.79 ± 15.87 |
| OMICBvac | 518.4 ± 5.717 | 92.22 ± 3.021 |

Figure S13. Quality control of OMV-vaccine production and LPS content by ELISA analysis. Data are presented as mean ± SEM (n=3).

*2. the authors insist about the role of ILC1 and pyroptosis to explain the efficacy of the combined vaccine, even though the depletion of CD8 T cells and NK cells also demonstrates their role. Could the ratio of ILC1/tumor cells to CD8 T cells/tumor cells or NK cells/tumor cells be shown to better assess the respective role of each population in the vaccine's effect?*

Response: We sincerely thank the reviewer for this excellent and insightful suggestion. We agree that quantitatively assessing the relative contribution of different immune subsets is crucial for a deeper understanding of the synergy.

In our current data, we find that the efficacy of the combined vaccine is best explained not by the dominance of a single population but by a synergistic interplay and functional amplification among them. As the reviewer noted, depletion studies confirm that both $CD8^+$ T cells and NK cells are essential. Our single-cell data (Figure 6B) further show that in the combined treatment group, both proliferating ($Ki67^+CD8^+$) T cells and ILC1s are coordinately upregulated, suggesting a cooperative expansion rather than a competitive one. When the cell subsets in Figure 6B were normalized to tumor cell number and re-analyzed (Figure S11), the data showed a concomitant increase in multiple immune populations, indicating that no single subset exclusively dominated the response.

The unique and pivotal role of ILC1s, we propose, lies in their ability to amplify the immune response. While T cells and NK cells are critical for initial recognition and killing, the infiltrating ILC1s, characterized by high cytotoxic potential without exhaustion, induce tumor cell pyroptosis. This pyroptotic cell death, as we demonstrated, releases inflammatory mediators (IL-1β, IL-18) that can further stimulate and sustain the overall anti-tumor immunity, creating a positive feedback loop. Therefore, the synergy likely arises from $CD8^+$ T cells and NK cells providing specificity and broad targeting, while ILC1s provide a potent amplification mechanism via pyroptosis.

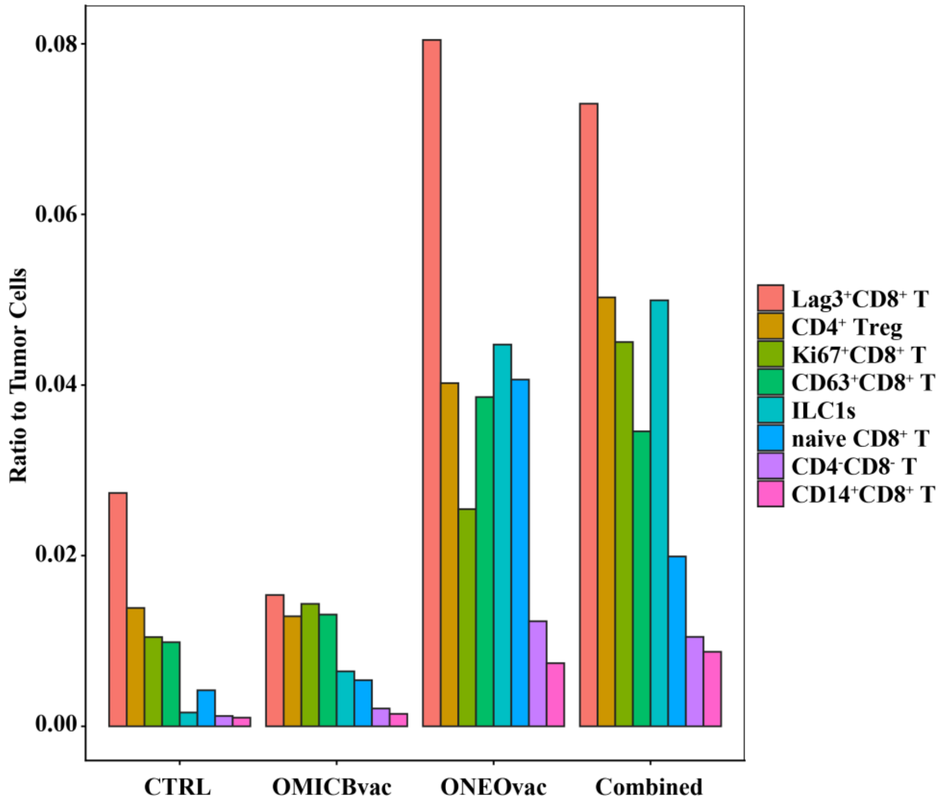


Figure S11. Proportions of various immune cell populations relative to tumor cells across different treatment conditions from Figure 6B. Cell subsets analyzed include Lag3$^+$CD8$^+$ T cells, CD4$^+$ Tregs, Ki67$^+$CD8$^+$ T cells, CD63$^+$CD8$^+$ T cells, ILC1s, naive CD8$^+$ T cells, CD4$^-$CD8$^-$ T cells, and CD14$^+$CD8$^+$ T cells.

*3. Could NK cells and CD8 T lymphocytes also play a role in pyroptosis? The experiment with the IL-15 inhibitor is important but does not rule out a role for NK cells.*

Response: We sincerely thank the reviewer for this insightful comment regarding the potential roles of NK cells and CD8$^+$ T lymphocytes in pyroptosis. The IL-15 inhibitor experiment alone cannot definitively exclude their involvement, as IL-15 is indeed a key survival factor for all three lymphocyte populations. However, our existing data provide strong evidence pointing to ILC1s as the primary direct mediators of pyroptosis in our model (Figure 6A, B). The most direct support comes from our in vitro co-culture experiments, where sorted ILC1s from treated tumors were sufficient to induce GSDMD-dependent pyroptosis in target cells (Figure 7A, B). This demonstrates their intrinsic capability to execute this specific cell death pathway independently of other immune cells in the tumor microenvironment. Furthermore,

the transcriptional profile of $CD8^+$ T cells from our single-cell RNA sequencing data offers important contextual evidence. These cells displayed a distinct exhausted phenotype characterized by high expression of PD-1 and LAG-3, which is typically associated with impaired cytotoxic function (Figure 6C). While they are undoubtedly essential for the overall antitumor response, their functional state makes them less likely to be the main drivers of the robust pyroptosis we observed.

We therefore propose a collaborative model where these immune populations play complementary but distinct roles in Discussion: "Specifically, $CD8^+$ T cells and NK cells are crucial for initial antigen-specific recognition and tumor cell killing, potentially through classical cytotoxic pathways, while the recruited ILC1s act as specialized effectors that amplify the immune response by initiating the pyroptotic cascade." This integrated perspective is supported by our current dataset and provides a coherent explanation for the synergistic efficacy of the combined vaccine.

*4. The authors show that ILC1s exert cytotoxicity against tumor cells. Is this cytotoxicity dependent on NKG2D? As a control, could the authors show that ILC1s do not exert cytotoxicity against normal hepatocytes?*

Response: We sincerely thank the reviewer for these important suggestions. To directly address them, we performed supplementary experiments. Blockade of NKG2D with a specific antibody (anti-NKG2D) partially reduced ILC1-mediated tumor cell killing in vitro (from 62.72±3.953% to 41.79±3.071%, Figure S12A), indicating NKG2D is involved but not the sole mechanism. Furthermore, these same ILC1s showed negligible cytotoxicity against AML12 normal murine hepatocytes (Figure S12B), confirming their tumor-selective activity and safety profile. This high specificity was also corroborated by the absence of liver damage in our comprehensive in vivo safety assessment (Figure S8). These results have been incorporated into the "Results" section under "IL-15 enhances ILC-mediated pyroptosis for antitumor effects" as follows: "In addition, this cytotoxicity partially depends on NKG2D but shows high tumor specificity, as ILC1s exhibited minimal activity against normal AML12 hepatocytes (Figure S12). This selectivity aligns with

the absence of liver damage in our in vivo safety assessment (Figure S8), confirming the therapeutic safety and efficiency of vaccine-induced ILC1s."

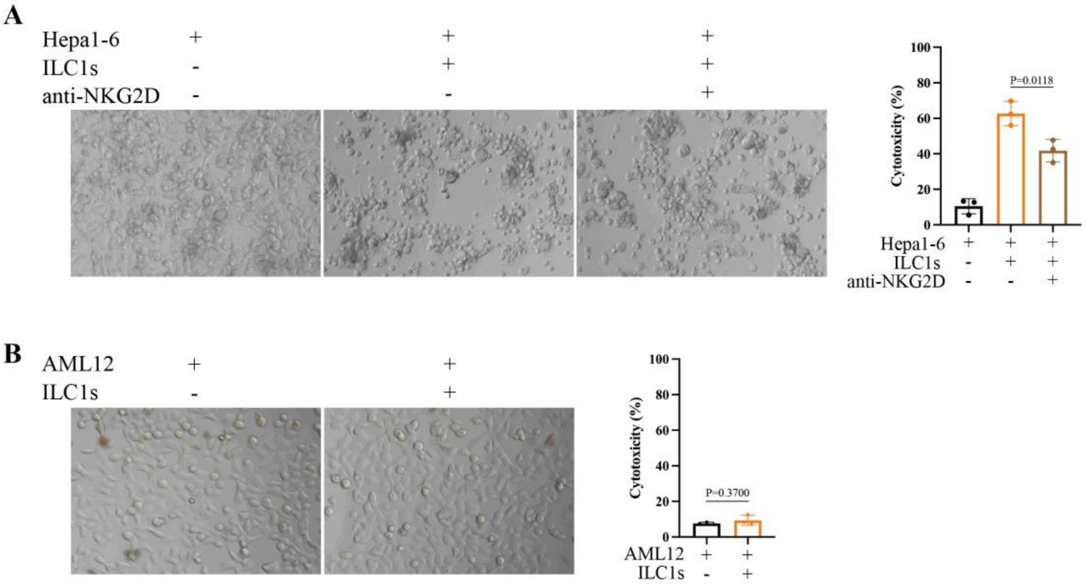


Figure S12. Microscopic images of ILC1s co-cultured with Hepa1-6 (A) and AML12 (B) cells after 48 hours. The cell death percentage induced by ILC1s was analyzed by LDH detection (n=3).

*5. Line 345: Isn't it surprising that after 48 hours of co-culture with dendritic cells, neoantigen-specific T lymphocytes have already been primed and are then able to lyse tumor cells?*

Response: We sincerely thank the reviewer for raising this point. The 48-hour timeline is indeed insufficient for de novo priming of naïve T cells. This assay, however, was designed as a recall response. The splenic T cells were isolated from mice that had already been vaccinated three times with ONEOvac in vivo, meaning they contained a pre-existing pool of neoantigen-experienced T cells. The short in vitro co-culture with antigen-loaded dendritic cells served to re-stimulate these pre-primed, effector-capable T cells, allowing us to assess their functional cytotoxicity. We have clarified this point in the revised manuscript to avoid any misinterpretation.

*6. Fig. S7C: It is unclear whether the difference between the use of vaccines as monotherapy vs. in combination is significant. Statistical tests are missing.*

Response: Thanks very much. We have added the appropriate statistical analysis to Figure S7C.

*7. Could the authors explain their choice of hyperimmunization at short intervals?*

Response: We sincerely thank the reviewer for this question regarding our immunization schedule. The choice of administering three vaccine doses at short intervals (days 0, 4, and 8) was based on two primary considerations aligned with our preclinical therapeutic model.

First, our goal was to rapidly induce a potent immune response capable of controlling an established, fast-growing orthotopic tumor. This condensed prime-boost schedule is a well-established strategy in therapeutic vaccine research to achieve high levels of effector T cells and antibodies in a short timeframe, which is critical for efficacy in aggressive tumor models.

Second, this schedule was pragmatically designed to synchronize the peak of the adaptive immune response with our key analytical time points (e.g., day 12 for immune profiling, day 21 for pyroptosis assessment). It allowed us to clearly capture the dynamic immunological changes induced by the vaccine combination during the critical window of tumor control.

While different schedules may be explored for optimizing long-term memory or clinical translation, the chosen regimen was effective and appropriate for testing the novel mechanistic synergy between neoantigen-specific and MICB-targeted immunity in our study.

*8. Some recent articles on vaccines using neoantigens in liver cancer could be mentioned (Yarchoan M Nat Med 2024).*

Response：We sincerely thank the reviewer for this suggestion. We have added citations to relevant recent work on neoantigen vaccines in liver cancer in the revised manuscript, including the studies by Baretti et al. (Nat Med 2025) and Yarchoan et al. (Nat Med 2024).

Referee #2

*Immunogenic peptides, known as tumor neoantigens, generated by specific gene variations in tumor cells can be recognized by T cells, inducing specific tumor cell killing. Thus, precise immunotherapy has been developed to target tumors. Nevertheless, hepatocellular carcinoma (HCC) with rapid progression and strong heterogeneity is normally resistant to this immunotherapy. In addition, tumors cells have high expression of MHC class I chain-related polypeptide A/B (MIC A/B) which can be recognized through NKG2D and eliminated by NK cells. However, tumor cells can evade immune recognition pathway through proteolytic shedding of MICA. A previous study has demonstrated that antibody-mediated inhibition of MICA and MICB by blocking their shedding through binding to the a3 domains can reactivate tumor immunity. In this study, the authors developed a novel synergistic therapeutic vaccine strategy that combines neoantigens and MICB a3 antigen to induce tumor immunity in HCC. They engineered and produced neoantigen protein (ONEOvac) and MICA or B peptides (OMICAvac or OMICBvac). In vitro studies demonstrated that ONEOvac could activate immune cells. In vivo studies revealed that OMICvac and OMICBvac could block the shedding of MICA/B in tumor cells, with the highest efficacy in OMICBvac. Thus, the authors, by using B6 mice with subcutaneous injection of Hep1-6 (a B6 derived liver cancer cell line), evaluated whether vaccination of ONEOvac and OMICBvac could elicit tumor immunity, resulting in tumor regression. Indeed, ONEOvac and OMICBvac both could suppress tumor progression likely through T and NK cell induced tumor cell killing effects. The authors further examined the tumor killing effects of different injection and combination of ONEOvac and OMICBvac. It was found that the combined vaccination of these two has the best tumor killing effects when compared with the single vaccination. Besides, the tumor killing effects of vaccination were likely through pyroptosis as GASMD knockout reversed the effects. The authors also determined that the combined vaccination induced significant ILC1 cell infiltration which could be regulated by IL-15 since IL-15 inhibitor reduced ILC1 cell infiltration and tumor regression in mice treated with the combined vaccination. Overall, the*

*current study provides a novel combinational therapy by combining tumor neoantigen and MICA/B vaccination to enhance tumor killing effects. Several weaknesses identified in its current form should be carefully addressed.*

Response: Thanks very much to comment our work as "a novel combinational therapy". We sincerely appreciate the efforts and comments from the reviewer to significantly improve the quality of our manuscript. All the arisen issues have been addressed.

*1. In the materials and methods, the authors described that plasmids were transformed into E. coli. They later described that plasmids were transfected into E. coli. Please revise the correct usage accordingly.*

Response: Thanks very much for pointing out this mistake. We have reviewed the entire manuscript and corrected this terminology error. All instances referring to the introduction of plasmids into *E. coli* now use the term "transformed".

*2. What is wet experiment? Please describe the manuscript in a professional way.*

Response: Thanks very much for pointing out this mistake. We have replaced the sentence (This result also verified the previous wet experiment data) with a more precise and professional description: "This finding at the single-cell transcriptome level independently corroborates the enhanced T and NK cell infiltration observed in our experimental validation."

*3. In Fig. 1, some experiments are described with details about the dose of peptides. However, some experiments have no detailed information making data interpretation difficult.*

Response: Thanks very much for the comment. We have thoroughly reviewed the figure legend for Figure 1 and have added the missing experimental details (marked by red) to ensure full clarity.

*4. In Fig. 2E, did the flowcytometry data show consistent higher CD8+ T cell population in ONEOvac and OMICBvac treated tumor samples when compared with the control or OMVs groups?*

Response: Thans very much. Figure 2E specifically quantifies neoantigen-specific $CD8^+$ T cells (identified by Ptpn2-H2kb tetramer staining), not the total $CD8^+$ T cell population. Here, ONEOvac (the neoantigen vaccine) induced a significant increase in Ptpn2-specific $CD8^+$ T cells compared to all other groups (PBS, OMVs, and OMICBvac).

The data for the total $CD8^+$ T cell population are presented in Figure 2D (IHC staining) and Figure 3K (flow cytometry analysis). In those figures, we do observe an increase in total $CD8^+$ T cells in both the ONEOvac and OMICBvac groups compared to the control groups, which aligns with the reviewer's query.

*5. Authors suggested that these vaccination treatments did not influence normal organ functions by analyzing histological changes and serum markers in the mice with orthotopic liver cancer cell inoculation. It is expected that the treated groups with no tumor cells should have reduced ALT and AST. However, the authors' findings contradicted the complete eradication of liver cancer cells (Fig. S8).*

Response: Thanks very much for the comment. We agree that the successful clearance of tumors would ideally be reflected in improved liver function markers. The key to interpreting our data lies in the dynamic interplay between tumor resolution and the immune response within the liver. To provide essential context and prevent any potential misunderstanding, we have explicitly clarified in the legend of Figure S8 that the serum for these analyses was collected on day 12. This specific time point corresponds to the peak of vaccine-induced immune activation. At this juncture, the liver is the site of an intensive immunological process, with substantial infiltration and activity of T cells, NK cells, and ILC1s working to eliminate tumor cells. This robust, localized inflammatory response, while essential for efficacy, can transiently affect the surrounding parenchyma and lead to the release of enzymes from both dying tumor cells and marginally affected hepatocytes. This phenomenon of transient,

immunotherapy-related enzyme elevation is a recognized counterpart to effective anti-tumor immunity. The most critical evidence supporting the safety of our strategy is the absence of any significant histological damage in the H&E-stained liver sections (Fig. S8A). This demonstrates that the observed serological profile reflects a manageable inflammatory state rather than irreversible organ injury. In conclusion, the combined data from histology and serology confirm that our dual-vaccine strategy is well-tolerated and does not cause significant harm to normal liver architecture or function.

*6. In Fig. 3, all mice dead in CTRL group, how did the authors obtain samples for analysis? If tumor samples were collected after mice dead, would this impact results?*

Response: Thanks very much for the comment. We apologize for any confusion caused by the initial description. To clarify, the samples for analysis in Figure 3F-M were not obtained from mice that had died or were moribund. 5 mice in each group were re-performed for analysis at a fixed early time point (day 12 post-treatment initiation) to assess the tumor immune microenvironment. This content has already been described in the article before the description of the results in Figure 3F-M. This time point was chosen specifically because it precedes significant mortality, as evidenced by the bioluminescence imaging and survival curve in Figure 3D and 3E, ensuring that all samples were collected from animals in a stable condition. The survival data (Figure 3D, E) were generated from the remaining 7 mice in each group that were monitored over the long term. We have clarified this experimental design in the legend of Figure 3F to prevent any misunderstanding. This approach ensures that the analytical data accurately reflect the immunologic state induced by the treatment.

*7. In Fig. 3, why was data collected with different N numbers?*

Response: Thanks very much. The different N numbers in Figure 3 stem from our experimental design, which employed separate, pre-defined groups of mice for distinct analytical purposes. For the survival study and longitudinal tumor burden monitoring presented in Figure 3D and 3E (n=7), a group of mice was monitored over

the long term without any intermediate procedures that could affect their survival. Conversely, the data in Figure 3F-M (n=5) were generated from a separate, parallel group of mice that were humanely euthanized at a specific early time point (day 12) to enable a comprehensive analysis of the tumor immune microenvironment, including flow cytometry, ELISA, and other assays that require terminal procedures. We have clarified this experimental design in the legend of Figure 3 to prevent any misunderstanding.

*8. In Fig. 4E, the images are poor quality.*

Response: Thanks very much for the comment. We have improved the quality of Figure 4E in the revised manuscript.

*9. In Fig. 4I and J, many mice dead before the authors collected tumor samples. How did the authors get 5 tumor samples for each group?*

Response: Thanks very much for the comment. To clarify, the tumor samples for Figure 4I and J were collected from mice that were part of a separate experimental group, not from the mice used for survival monitoring. In this separate group, we had 10 mice per treatment condition. From these 10 mice, 5 were randomly selected and euthanized at the predetermined time point of day 21 to collect fresh tumor samples for the IL-1β and IL-18 analyses. This approach ensured that all samples were obtained from healthy, non-moribund animals at a consistent time point, guaranteeing data quality and reliability. We have added this explanation to the Figure 4 legend in the revised manuscript to provide this clarity.

*10. In Fig. 5B, the authors stated T&NK cell increased in the ONEOvac and combined groups compared to the CTRL group. However, hepatocytes decreased substantially. Did the hepatocyte population include HCC?*

Response: We sincerely thank the reviewer for raising this critical point. In our initial cell type annotation (Figure 5B), the "Hepatocytes" cluster was defined by the expression of canonical hepatocyte markers (e.g., Alb, Afp). As expected, this

population indeed contained a mixture of both normal hepatocytes and tumor cells, since Hepa1-6 is a hepatocellular carcinoma cell line. The key subsequent step, as shown in Figure 5C, was to apply copy number variation (CNV) analysis using SCEVAN specifically to this “Hepatocytes” cluster. This allowed us to definitively distinguish the CNV-aberrant tumor cells from the CNV-normal hepatocytes. Therefore, while the broad “Hepatocytes” annotation in Figure 5B includes HCC cells, our downstream analyses (Figure 5C-G) focus specifically on the CNV-defined malignant cells that were identified within that parent population. The decrease of the “Hepatocytes” population in Figure 5B thus reflects a combination of tumor cell killing and immune cell infiltration, and the precise quantification of tumor cell elimination is provided by the SCEVAN-based analysis in the following panels.

*11. In Fig. 5C, why do the T SNE images have almost no overlap cell types?*

Response: We sincerely thank the reviewer for this pertinent observation. The distinct separation in the t-SNE plot (Figure 5C) is indeed by design and reflects the specific purpose of this particular analysis. This t-SNE visualization is not based on standard gene expression for cell type clustering. Instead, it is generated from the output of SCEVAN, an algorithm that infers copy number variations (CNVs) by analyzing the expression of genes within contiguous genomic regions. This plot, therefore, visualizes cells based on their inferred CNV profiles. Since CNV patterns differ drastically between malignant tumor cells and diploid normal cells, they form two well-separated clusters in this dedicated analysis. This clear separation is a positive result, confirming the successful computational identification of tumor cells (as shown in the associated panels) for our downstream, neoantigen-specific analyses.

*12. In Fig. 6C-E, what did you want to compare? In Fig. 6E, the Heatmap data actually suggested many inflammatory cytokines are related to these changes. In Fig. 6F, how did you measure IL-15 in tumor samples by using ng/ml as unit? What samples did you use?*

Response: We sincerely thank the reviewer for these questions. For Figures 6C-E, these analyses aimed to functionally compare the immune cell subsets identified by scRNA-seq. Figure 6C directly compares the cytotoxic and exhausted state of $CD8^+$ T cells versus the highly cytotoxic but non-exhausted profile of ILC1s. Figure 6D defines the unique surface marker signature of ILC1s. Figure 6E specifically compares the expression of core genes within the IL-15 signaling pathway across all subsets, visually confirming its predominant activity in ILC1s, which aligns with their biology and our functional data.

For Figure 6F, the use of “ng/mL” reflects the concentration of IL-15 in the standardized tissue lysate supernatant that was directly loaded into the ELISA plate. To clarify the methodology, we have re-described in detail in the revised “Supplementary Methods” section under “Antigen-Specific IgG analysis and ELISA” as follows: “Additionally, to assess cytokine levels in the tumors, the collected tumors of equal weight were lysed in the same volume of RIPA lysis buffer containing 1% phenylmethylsulfonyl fluoride (PMSF) and 2% protease inhibitor cocktail. The samples were then homogenized with grinding beads using a tissue homogenizer at low temperature for 6 minutes. The resulting suspension was centrifuged at 12, 000 g at 4°C for 5 minutes. The supernatants of equal volume were subsequently analyzed using ELISA kits (Boster Biological Technology, Wuhan, China) to measure IL-1β, IL-18 and IL-15, following the standard protocols.” Since the entire process, from tissue weight to the volume of lysate analyzed, was consistently standardized across all samples, the concentration obtained in "ng/mL" is directly proportional to the total IL-15 content per mass of tissue and is therefore valid for cross-group comparison.

*13. In Fig. 7C-H, the authors described N is equal to 5 for all studies. However, the presented data did not support the statement. Besides, how could the authors get identical data in CTRL group for Fig. 7G? It is common that inflammatory cytokines vary significantly between mouse samples.*

Response: We sincerely apologize for the error in our initial figure legend and thank the reviewer for the careful attention to this detail. In our initial analysis, we randomly

selected 3 mice from each group of 5 for the cytokine detection to perform assessment. To make our findings more robust and reliable, we proceeded to analyze the remaining 2 samples from all groups.

We also sincerely thank the reviewer for the close examination of the data in Figure 7G. In the control group for the IL-1β measurement, the values we obtained were 0.622, 0.619, and 0.611 pg/mL. While these values are indeed not mathematically identical, they are highly consistent, resulting in an extremely small error bar that may give the visual impression of being identical in the figure.

This remarkable consistency within the control group likely stems from the homogeneous nature of the tumor model at this specific early time point and the highly standardized procedures we employed for tissue collection, processing, and ELISA analysis. The low baseline variation strengthens the reliability of the observed significant increases in the treatment groups. We have verified the raw data, and these values are accurate. The complete source data for this figure has been provided as part of the manuscript submission.

*14. Should the authors repeat the experiments in a humanized mouse model by using human HCC cells?*

Response: Thanks very much for the suggestion. Our study focuses on the synergistic antitumor effects achieved by combining T cell-mediated and humoral immune responses. Although it is technically possible to simulate the activation of human B cells by MICA/B-vaccination through transplantation of $CD34^+$ cells derived from human umbilical cord blood, but the T cells differentiated from $CD34^+$ cells will undergo thymic selection in the murine thymus. As a result, their TCRs fail to recognize human HCC antigens presented by human MHC molecules, making it impossible to reproduce the combined antitumor mechanism in such models. Furthermore, while human T cell transplantation into tumor-bearing mice could partially restore T cell immunity, $CD34^+$-derived cells cannot differentiate into innate lymphoid cells (ILC1s). Therefore, the critical interplay between T cell- and ILC1-mediated immunity, as well as the associated humoral immune activation,

cannot be faithfully modeled in humanized mice. For these mechanistic and technical reasons, a humanized mouse model is not suitable for validating our dual immune activation strategy at this stage. We will explicitly acknowledge this limitation in the revised Discussion and emphases that future studies will aim to validate the findings in humanized systems once suitable models become available.

12th Feb 2026

Dear Prof. Liu,

Thank you for submitting your revised study. We have now received the reports from the two initial referees. As you will see below, they are overall satisfied with the revisions. I will thus be able to accept your manuscript once the following matters are addressed:

1/ Please address referee #2's concerns by measuring ALT and AST if you have samples available. If it is not the case, please discuss this limitation in the text.

2/ Manuscript text:
- Please remove the colored text and only keep in track changes mode any new modification to the text.
- Please correct the order of the sections in the manuscript text to: Abstract / The Paper Explained / Introduction / Results / Discussion / Methods / Data Availability / Acknowledgements / Disclosure and Competing Interests Statement / References / Figure Legends.
- Please note that all corresponding authors are required to supply an ORCID ID for their name upon submission of a revised manuscript. An ORCID identifier is currently missing for Xiaolong Liu.
- "Material and Methods" should be renamed "Methods". Please provide antibodies dilutions. In the statistics section, please include a statement on sample size, blinding and inclusion/exclusion criteria.
- Data availability: please remove "All data generated or analyzed during this study are included in this article. Inquiries about further material documents can be directed to the corresponding author."
- Please remove the section 'Author contributions' and provide instead CRediT (Contributor Role Taxonomy) terms in the submission system.
- A "Disclosure and Competing Interests Statement" should be provided in the main text.

3/ Figures and Appendix:
- Figure check: please upload your responses to the enquiry of our data integrity analyst as part of your point by point letter, modify your figures and legends accordingly, and clearly indicate the changes that have been made and why. Please also update the associated Source Data. Please indicate which mice (rows and columns) are identical in Figure 3D and Appendix Fig S5B.
- Appendix: Please move the supplementary methods to the main manuscript file. We would also encourage you to replace some of your Appendix figures by Expanded View (EV) Figures and Tables that are collapsible/expandable online. EV Figures should be cited as 'Figure EV1, Figure EV2" etc... in the text and their respective legends should be included in the main text after the legends of regular figures. For the figures that you do NOT wish to display as Expanded View figures, they should be bundled together with their legends in a single PDF file called *Appendix*, which should start with a short Table of Content (including page numbers). Appendix figures should be referred to in the main text as: "Appendix Figure S1, Appendix Figure S2" etc.
- Several figures have many panels. Please consider whether you want to split your figures to make them more accessible to the readers.
- Please note that a callout for Appendix Table S3 is missing in the text.
- Please address the query from our data editors in the figure legends:
1. Please indicate the statistical test used for data analysis in the legends of figures 1F, G, H, I, N, O; 2B, D, E, F; 3A, B, E, G, H, I, J, K, L, M; 4B, C, D, E, H, I, J; 5F, G; 6F, G; 7B, E, F, G, H"
2. Please note that the box plots need to be defined in terms of minima, maxima, centre, bounds of box and whiskers, and percentile in the legends of figures 5F, G
3. Please note that information related to n is missing in the legends of figures 1F, G, N, O; 3G, 4E, 5F, G; 6C, D; 7F
4. Please note that the error bars are not defined in the legends of figures 1F, G, H, I, L, M, N, O; 2B, D, E, F; 3A, B, F-M; 4B, C, D, E, H, I, J; 6F, 7B, E, F, G, H

4/ Checklist:
- please provide information for Experimental study design and statistics - inclusion/exclusion criteria
- please fill in the section Data availability/ primary dataset deposition

5/ Thank you for providing Source Data. Please make sure that all panel and subpanel folders are clearly labeled. Please also provide source data for FACS experiments.

6/ The paper explained: I have shortened your text, please let me know if you agree or amend as you see fit:
Problem
Neoantigen-based vaccines hold great promise for personalized cancer immunotherapy, yet their efficacy is often compromised by tumor immune evasion mechanisms. Moreover, even when neoantigen-specific T cells are successfully primed, they often become exhausted in the immunosuppressive tumor microenvironment. Therefore, there is an urgent need for integrated

vaccine platforms that can simultaneously enhance antigen presentation, prevent immune escape, and sustain potent antitumor immunity across diverse tumor types.
Results
We developed a novel bacterial outer membrane vesicle (OMV)-based vaccine platform that co-delivers personalized neoantigens and the shared MICB α3 domain. This dual-targeted approach simultaneously activates neoantigen-specific $CD8^+$ T cells and induces high-titer antibodies that block MICB shedding, thereby preserving NKG2D-mediated tumor recognition. In multiple orthotopic and metastatic mouse models of hepatocellular and pancreatic cancers, the combined vaccine elicited superior antitumor efficacy and prolonged survival compared to either component alone. Mechanistically, single-cell RNA sequencing revealed that the therapy recruited and activated a population of IL-15-armed ILC1s, which mediated tumor pyroptosis via GZMA/GZMB-induced cleavage of GSDMD. This pyroptotic cell death amplified the inflammatory tumor microenvironment and enhanced systemic antitumor immunity without inducing significant toxicity.
Impact
This study introduces a versatile and synergistic vaccination strategy that merges personalized and shared antigen targeting to overcome key limitations of current neoantigen immunotherapies. Importantly, the OMV-based delivery system is scalable, clinically tractable, and adaptable to various cancer types, offering a translational pathway toward more effective and broadly applicable cancer vaccines.

7/ Please upload and fill a Reagents and Tools Table, which can be downloaded from our author guidelines.

8/ As part of the EMBO Publications transparent editorial process initiative (see our Editorial at http://embomolmed.embopress.org/content/2/9/329), EMBO Molecular Medicine will publish online a Review Process File (RPF) to accompany accepted manuscripts.
This file will be published in conjunction with your paper and will include the anonymous referee reports, your point-by-point response and all pertinent correspondence relating to the manuscript. Let us know whether you agree with the publication of the RPF and as here, if you want to remove or not any figures from it prior to publication.
Please note that the Authors checklist will be published at the end of the RPF.

I look forward to receiving your revised manuscript.

Yours sincerely,

Lise Roth

Lise Roth, PhD
Senior Editor
EMBO Molecular Medicine

To submit your manuscript, please follow this link:
https://embomolmed.msubmit.net/cgi-bin/main.plex

***** Reviewer's comments *****

Referee #1 (Remarks for Author):

The authors have satisfactorily addressed my various concerns

Referee #2 (Remarks for Author):

Thanks for the authors' clarifications. I still have one suggestion regarding the AST and ALT studies.

Original comment:
5.Authors suggested that these vaccination treatments did not influence normal organ functions by analyzing histological changes and serum markers in the mice with orthotopic liver cancer cell inoculation. It is expected that the treated groups with no tumor cells should have reduced ALT and AST. However, the authors' findings contradicted the complete eradication of liver cancer cells (Fig. S8).
Authors' Response: Thanks very much for the comment. We agree that the successful clearance of tumors would ideally be reflected in improved liver function markers. The key to interpreting our data lies in the dynamic interplay between tumor resolution and the immune response within the liver. To provide essential context and prevent any potential misunderstanding,

we have explicitly clarified in the legend of Figure S8 that the serum for these analyses was collected on day 12. This specific time point corresponds to the peak of vaccine-induced immune activation. At this juncture, the liver is the site of an intensive immunological process, with substantial infiltration and activity of T cells, NK cells, and ILC1s working to eliminate tumor cells. This robust, localized inflammatory response, while essential for efficacy, can transiently affect the surrounding parenchyma and lead to the release of enzymes from both dying tumor cells and marginally affected hepatocytes. This phenomenon of transient, immunotherapy-related enzyme elevation is a recognized counterpart to effective anti-tumor immunity. The most critical evidence supporting the safety of our strategy is the absence of any significant histological damage in the H&E-stained liver sections (Fig. S8A). This demonstrates that the observed serological profile reflects a manageable inflammatory state rather than irreversible organ injury. In conclusion, the combined data from histology and serology confirm that our dual-vaccine strategy is well-tolerated and does not cause significant harm to normal liver architecture or function.

New comment: Thanks for your response. In this case, can you measure ALT and AST after you euthanize mice? This result will significantly strengthen your conclusion.

The authors addressed the editorial issues.

18th Mar 2026

Dear Prof. Liu,

Thank you for providing your revised manuscript. There are still a few issues that need addressing before I can proceed with acceptance:

1. An ORCID identifier is currently missing for Xiaolong Liu, as well as institutional email address.
2. Methods/Antibodies: please also provide dilutions for WB/FACS.
3. We note that you have not changed the composition of your figures. However, the figures with panels showing bioluminescence in tumor-bearing mice are very small and difficult to read. In particular for Figure 3, please either move panel D to the Appendix, or make it an EV figure. Alternatively, you could split Figure 3 in 2 figures. I would encourage you to also consider figures 4 and 7.
4. Checklist: you indicated completing the checklist, however I'm afraid I could not find the following information:
- Experimental study design and statistics - inclusion/exclusion criteria
- Data availability/ primary dataset deposition
5. Figure legends: please make sure that n is provided for each figure panel. Please define the error bars in all figure legends.
6. Figure check: thank you for addressing the figure issues raised. Please provide the detailed response from your email dated January 28 in your point-by-point rebuttal letter. Please indicate in the legend of Appendix Figure S2 that the marker is the same, and provide source data for this Figure.

I look forward to receiving your revised manuscript.

Yours sincerely,

Lise Roth

Lise Roth, PhD
Senior Editor
EMBO Molecular Medicine

The authors addressed the remaining editorial issues.

26th Mar 2026

Dear Dr. Liu,

Thank you for submitting your revised files. We are pleased to inform you that your manuscript is accepted for publication and is now being sent to our publisher to be included in the next available issue of EMBO Molecular Medicine.

Your manuscript will be processed for publication by EMBO Press. It will be copy edited and you will receive page proofs prior to publication. Please note that you will be contacted by Springer Nature Author Services to complete licensing and payment information.

You may qualify for financial assistance for your publication charges - either via a Springer Nature fully open access agreement or an EMBO initiative. Check your eligibility: https://link.springer.com/journal/44321/how-to-publish-with-us

Should you be planning a Press Release on your article, please get in contact with embo_production@springernature.com as early as possible in order to coordinate publication and release dates.

If you have any questions, please do not hesitate to contact the Editorial Office. Thank you for your contribution to EMBO Molecular Medicine.

Yours sincerely,

Lise Roth, Ph.D
Senior Editor
EMBO Molecular Medicine

------------------------------------------------

>>> Please note that it is EMBO Molecular Medicine policy for the transcript of the editorial process (containing referee reports and your response letter) to be published as an online supplement to each paper. If you do NOT want this, you will need to inform the Editorial Office via email immediately. More information is available here: https://link.springer.com/partners/embo-press/editorial-policies#Peer%20review

## EMBO Press Author Checklist

| | |
|---|---|
| Corresponding Author Name: Jianhua Zou, Xiaolong Liu, Zhixiong Cai | |
| Journal Submitted to: EMBO MOLECULAR MEDICINE | |
| Manuscript Number: EMM-2025-22700 | |

**USEFUL LINKS FOR COMPLETING THIS FORM**
The EMBO Journal - Author Guidelines
EMBO Reports - Author Guidelines
Molecular Systems Biology - Author Guidelines
EMBO Molecular Medicine - Author Guidelines

**Reporting Checklist for Life Science Articles (updated January**
This checklist is adapted from Materials Design Analysis Reporting (MDAR) Checklist for Authors. MDAR establishes a minimum set of requirements in transparent reporting in the life sciences (see Statement of Task: 10.31222/osf.io/9sm4x). Please follow the journal's guidelines in preparing your
**Please note that a copy of this checklist will be published alongside your article.**

**Abridged guidelines for figures**
**1. Data**
The data shown in figures should satisfy the following conditions:
- the data were obtained and processed according to the field's best practice and are presented to reflect the results of the experiments in an accurate and unbiased manner.
- ideally, figure panels should include only measurements that are directly comparable to each other and obtained with the same assay.
- plots include clearly labeled error bars for independent experiments and sample sizes. Unless justified, error bars should not be shown for technical
- if n<5, the individual data points from each experiment should be plotted.  Any statistical test employed should be justified.
- Source Data should be included to report the data underlying figures according to the guidelines set out in the authorship guidelines on Data

**2. Captions**
Each figure caption should contain the following information, for each panel where they are relevant:
- a specification of the experimental system investigated (eg cell line, species name).
- the assay(s) and method(s) used to carry out the reported observations and measurements.
- an explicit mention of the biological and chemical entity(ies) that are being measured.
- an explicit mention of the biological and chemical entity(ies) that are altered/varied/perturbed in a controlled manner.
- the exact sample size (n) for each experimental group/condition, given as a number, not a range;
- a description of the sample collection allowing the reader to understand whether the samples represent technical or biological replicates (including how many animals, litters, cultures, etc.).
- a statement of how many times the experiment shown was independently replicated in the laboratory.
- definitions of statistical methods and measures:
  - common tests, such as t-test (please specify whether paired vs. unpaired), simple $\chi 2$ tests, Wilcoxon and Mann-Whitney tests, can be unambiguously identified by name only, but more complex techniques should be described in the methods section;
  - are tests one-sided or two-sided?
  - are there adjustments for multiple comparisons?
  - exact statistical test results, e.g., P values = x but not P values < x;
  - definition of 'center values' as median or average;
  - definition of error bars as s.d. or s.e.m.

**Please complete ALL of the questions below.**
**Select "Not Applicable" only when the requested information is not relevant for your study.**

**Materials**

| Newly Created Materials | Information included in the manuscript? | In which section is the information available? (Reagents and Tools Table, Materials and Methods, Figures, Data Availability Section) |
|---|---|---|
| New materials and reagents need to be available; do any restrictions apply? | Not Applicable | |

| Antibodies | Information included in the manuscript? | In which section is the information available? (Reagents and Tools Table, Materials and Methods, Figures, Data Availability Section) |
|---|---|---|
| For **antibodies** provide the following information:<br>- Commercial antibodies: RRID (if possible) or supplier name, catalogue number and or/clone number<br>- Non-commercial: RRID or citation | Yes | Materials and methods |

| DNA and RNA sequences | Information included in the manuscript? | In which section is the information available? (Reagents and Tools Table, Materials and Methods, Figures, Data Availability Section) |
|---|---|---|
| **Short novel DNA or RNA including primers, probes**: provide the sequences. | Yes | Supplementary Methods |

| Cell materials | Information included in the manuscript? | In which section is the information available? (Reagents and Tools Table, Materials and Methods, Figures, Data Availability Section) |
|---|---|---|
| **Cell lines:** Provide species information, strain. Provide accession number in repository **OR** supplier name, catalog number, clone number, and/**OR** RRID. | Yes | Materials and methods |
| **Primary cultures:** Provide species, strain, sex of origin, genetic modification status. | Not Applicable | |
| Report if the cell lines were recently **authenticated** (e.g., by STR profiling) and tested for mycoplasma contamination. | Yes | Materials and methods |

| Experimental animals | Information included in the manuscript? | In which section is the information available? (Reagents and Tools Table, Materials and Methods, Figures, Data Availability Section) |
|---|---|---|
| **Laboratory animals or Model organisms:** Provide species, strain, sex, age, genetic modification status. Provide accession number in repository **OR** supplier name, catalog number, clone number, **OR** RRID. | Yes | Materials and methods |
| **Animal observed in or captured from the field:** Provide species, sex, and age where possible. | Not Applicable | |
| Please detail **housing and husbandry conditions**. | Yes | Materials and methods |

| Plants and microbes | Information included in the manuscript? | In which section is the information available? (Reagents and Tools Table, Materials and Methods, Figures, Data Availability Section) |
|---|---|---|
| **Plants:** provide species and strain, ecotype and cultivar where relevant, unique accession number if available, and source (including location for collected wild specimens). | Not Applicable | |
| **Microbes:** provide species and strain, unique accession number if available, and source. | Not Applicable | |

| Human research participants | Information included in the manuscript? | In which section is the information available? (Reagents and Tools Table, Materials and Methods, Figures, Data Availability Section) |
|---|---|---|
| If collected and within the bounds of privacy constraints report on age, sex and gender or ethnicity for all study participants. | Not Applicable | |

| Core facilities | Information included in the manuscript? | In which section is the information available? (Reagents and Tools Table, Materials and Methods, Figures, Data Availability Section) |
|---|---|---|
| If your work benefited from core facilities, was their service mentioned in the acknowledgments section? | Not Applicable | |

**Design**

| Study protocol | Information included in the manuscript? | In which section is the information available? (Reagents and Tools Table, Materials and Methods, Figures, Data Availability Section) |
|---|---|---|
| If study protocol has been **pre-registered, provide DOI in the manuscript**. For clinical trials, provide the trial registration number **OR** cite DOI. | Not Applicable | |
| Report the **clinical trial registration number** (at ClinicalTrials.gov or equivalent), where applicable. | Not Applicable | |

| Laboratory protocol | Information included in the manuscript? | In which section is the information available? (Reagents and Tools Table, Materials and Methods, Figures, Data Availability Section) |
|---|---|---|
| Provide DOI OR other citation details if **external detailed step-by-step protocols** are available. | Not Applicable | |

| Experimental study design and statistics | Information included in the manuscript? | In which section is the information available? (Reagents and Tools Table, Materials and Methods, Figures, Data Availability Section) |
|---|---|---|
| Include a statement about **sample size** estimate even if no statistical methods were used. | Yes | Materials and methods |
| Were any steps taken to minimize the effects of subjective bias when allocating animals/samples to treatment (e.g. **randomization procedure**)? If yes, have they been described? | Yes | Materials and methods |
| Include a statement about **blinding** even if no blinding was done. | Yes | Materials and methods |
| Describe **inclusion/exclusion criteria** if samples or animals were excluded from the analysis. Were the criteria pre-established?<br><br>If sample or data points were omitted from analysis, report if this was due to attrition or intentional exclusion and provide justification. | Not Applicable | |
| For every figure, are **statistical tests** justified as appropriate? Do the data meet the assumptions of the tests (e.g., normal distribution)? Describe any methods used to assess it. Is there an estimate of variation within each group of data? Is the variance similar between the groups that are being statistically compared? | Yes | Materials and methods |

| Sample definition and in-laboratory replication | Information included in the manuscript? | In which section is the information available? (Reagents and Tools Table, Materials and Methods, Figures, Data Availability Section) |
|---|---|---|
| In the figure legends: state number of times the experiment was **replicated** in laboratory. | Yes | Figure legends |
| In the figure legends: define whether data describe **technical or biological replicates**. | Yes | Figure legends |

## Ethics

| Ethics | Information included in the manuscript? | In which section is the information available? (Reagents and Tools Table, Materials and Methods, Figures, Data Availability Section) |
|---|---|---|
| Studies involving **human participants**: State details of **authority granting ethics approval** (IRB or equivalent committee(s), provide reference number for approval. | Not Applicable | |
| Studies involving **human participants**: Include a statement confirming that **informed consent** was obtained from all subjects and that the experiments conformed to the principles set out in the WMA Declaration of Helsinki and the Department of Health and Human Services Belmont Report. | Not Applicable | |
| Studies involving **human participants: For** publication of **patient photos**, include a statement confirming that consent to publish was obtained. | Not Applicable | |
| Studies involving experimental **animals**: State details of **authority granting ethics approval** (IRB or equivalent committee(s), provide reference number for approval. Include a statement of compliance with ethical regulations. | Yes | Materials and methods |
| Studies involving **specimen and field samples:** State if relevant **permits** obtained, provide details of authority approving study; if none were required, explain why. | Not Applicable | |

| Dual Use Research of Concern (DURC) | Information included in the manuscript? | In which section is the information available? (Reagents and Tools Table, Materials and Methods, Figures, Data Availability Section) |
|---|---|---|
| Could your study fall under dual use research restrictions? Please check biosecurity documents and list of **select agents and toxins** (CDC): https://www.selectagents.gov/sat/list.htm | Not Applicable | |
| If you used a select agent, is the security level of the lab appropriate and reported in the manuscript? | Not Applicable | |
| If a study is subject to dual use research of concern regulations, is the name of the **authority granting approval and reference number** for the regulatory approval provided in the manuscript? | Not Applicable | |

## Reporting

The MDAR framework recommends adoption of discipline-specific guidelines, established and endorsed through community initiatives. Journals have their own policy about requiring specific guidelines and recommendations to complement MDAR.

| Adherence to community standards | Information included in the manuscript? | In which section is the information available? (Reagents and Tools Table, Materials and Methods, Figures, Data Availability Section) |
|---|---|---|
| State if relevant guidelines or checklists (e.g., **ICMJE, MIBBI, ARRIVE, PRISMA**) have been followed or provided. | Not Applicable | |
| For **tumor marker prognostic studies,** we recommend that you follow the **REMARK** reporting guidelines (see link list at top right). See author guidelines, under 'Reporting Guidelines'. Please confirm you have followed these guidelines. | Not Applicable | |
| For **phase II and III randomized controlled trials**, please refer to the **CONSORT** flow diagram (see link list at top right) and submit the CONSORT checklist (see link list at top right) with your submission. See author guidelines, under 'Reporting Guidelines'. Please confirm you have submitted this list. | Not Applicable | |

## Data Availability

| Data availability | Information included in the manuscript? | In which section is the information available? (Reagents and Tools Table, Materials and Methods, Figures, Data Availability Section) |
|---|---|---|
| Have **primary datasets** been deposited according to the journal's guidelines (see 'Data Deposition' section) and the respective accession numbers provided in the Data Availability Section? | Not Applicable | |
| Were **human clinical and genomic datasets** deposited in a public access-controlled repository in accordance to ethical obligations to the patients and to the applicable consent agreement? | Not Applicable | |
| Are **computational models** that are central and integral to a study available without restrictions in a machine-readable form? Were the relevant accession numbers or links provided? | Not Applicable | |
| If publicly available data were reused, provide the respective **data citations in the reference list**. | Not Applicable | |
